# Discovery of novel and potent CDK8 inhibitors for the treatment of acute myeloid leukaemia

**DOI:** 10.1080/14756366.2024.2305852

**Published:** 2024-01-23

**Authors:** Zhuoying Chen, Quan Wang, Yao Yao Yan, Dalong Jin, Yumeng Wang, Xing Xing Zhang, Xin Hua Liu

**Affiliations:** a School of Pharmacy, Anhui Medical University, Hefei, P. R. China; b School of Biology, Food and Environment, Hefei University, Hefei, China

**Keywords:** CDK8 inhibitor, STAT-1, STAT-5, AML

## Abstract

It has been reported that CDK8 plays a key role in acute myeloid leukaemia. Here, a total of 40 compounds were rational designed and synthesised based on the previous SAR. Among them, compound **12** (*3-(3-(furan-3-yl)-1H-pyrrolo[2,3-b]pyridin-5-yl)benzamide*) showed the most potent inhibiting activity against CDK8 with an IC_50_ value of 39.2 ± 6.3 nM and anti AML cell proliferation activity (molm-13 GC_50_ = 0.02 ± 0.01 *μ*M, MV4-11 GC_50_ = 0.03 ± 0.01 *μ*M). Mechanistic studies revealed that this compound **12** could inhibit the phosphorylation of STAT-1 and STAT-5. Importantly, compound **12** showed relative good bioavailability (*F* = 38.80%) and low toxicity *in vivo*. This study has great significance for the discovery of more efficient CDK8 inhibitors and the development of drugs for treating AML in the future.

## Introduction

Acute myeloid leukaemia (AML), a highly heterogeneous disease derived from the malignant clonal proliferation of abnormally differentiated myeloid lineage cells, is very difficult to be cured in young adults^1^^,^^2^. In the recent 40 years, daunorubicin or idarubicin and cytarabine as the standard induction (initial) chemotherapy were selected for the treatment of AML with only 70-80% of the complete response due to the intrinsic disease resistance^1^. In recent years, more and more targeted inhibitors have been used to treat AML with better therapeutic effects, such as IDH1/IDH2 inhibitors^3^^,^^4^, FLT3 inhibitors^3^^,^^5^, BCL-2 inhibitors^3^^,^^4^ *etc*. Therefore, to discover novel targeted inhibitors for treatment of AML is of great significance, especially for patients who have been resistant to drugs.

**Figure 1.**
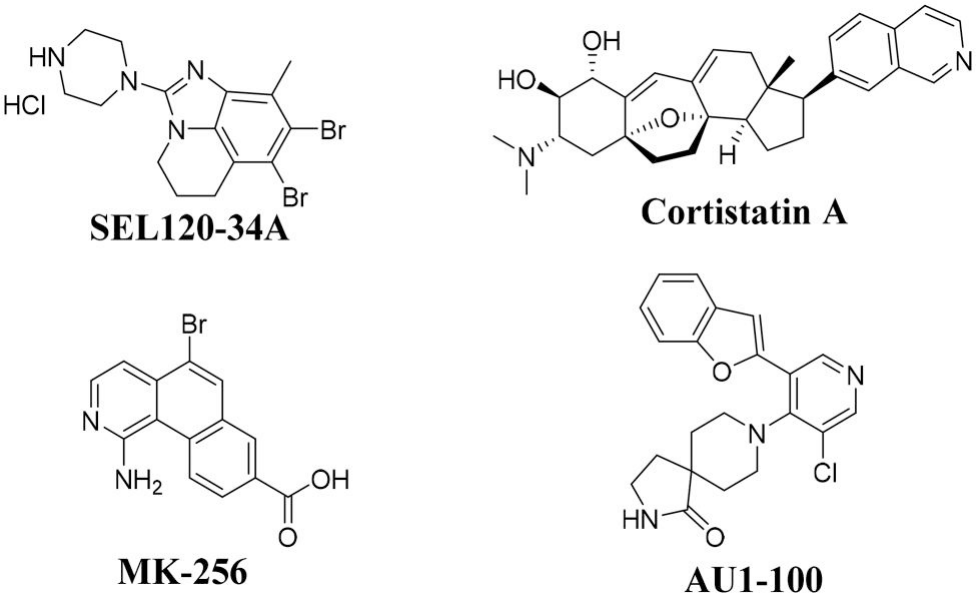
Chemical structures of some CDK8 inhibitors.

Signal transducer and activator of transcription 5 (STAT-5) had been found to exist in AML cells and exhibited sustained high activity. Importantly, STAT-5 also played a key role in mediating the relationship between several malignant diseases in AML cells^5–9^. In the recent ten years, many CDK8 inhibitors have been reported to exhibited good anti-tumour activity, such as cortistainA^10^, AU1-100^11^, MK-256^12^, SEL120-34A^7^, CCT251545^13^, CCT251591^14^, MSC2530818^15^, BI-1347^16^^,^^17^, *etc*. It has been reported compound SEL120-34A (Figure 1) as the potent CDK8 inhibitor could inhibit the phosphorylation of STAT1 S727 and STAT5 S726 in AML cells^7^. More importantly, in 2019, SEL120-34A was approved for clinical trials for treatment of advanced AML (NCT04021368)^18^. In addition, many CDK8 inhibitors were also reported to have good anti proliferative activity on AML cells^10–12^^,^^19–22^, such as cortistatinA^10^, AU1-100^11^, MK-256^12^, *etc (*Figure 1). Although, three CDK8 inhibitors, such as BCD-115 (NCT03065010), SEL120-34A (NCT04021368, NCT05052255) and TSN-084 (NCT05300438) have been approved for clinical trials, however, due to toxicity and drug PK efficacy, there are no commercially available drugs on the market. So, we think it is great significance to discover novel potent CDK8 inhibitors for treatment of AML through inhibiting the phosphorylation of STAT1 S727 and STAT5 S726.

In this work, based on rational design and structure-activity relationship discussion, compound **12** (*3–(3-(furan-3-yl)-1H-pyrrolo[2,3-b]pyridin-5-yl)benzamide*) as a potent CDK8 inhibitor was found. Further mechanistic studies showed that compound **12** could inhibit the phosphorylation of STAT5 S726 and showed good antiproliferative activity for acute myeloid leukaemia cell lines (molm-13 GC_50_ = 0.02 ± 0.01 *μ*M, MV4-11 GC_50_ = 0.03 ± 0.01 *μ*M). Importantly, compound **12** showed relatively good bioavailability (*F* = 38.80%, which is better than our previous report) and low toxicity *in vivo.* It is of great significance for the discovery of more efficient CDK8 inhibitors and the development of drugs for treating AML.

## Results and discussions

### Design and optimisation

**Figure 2.**
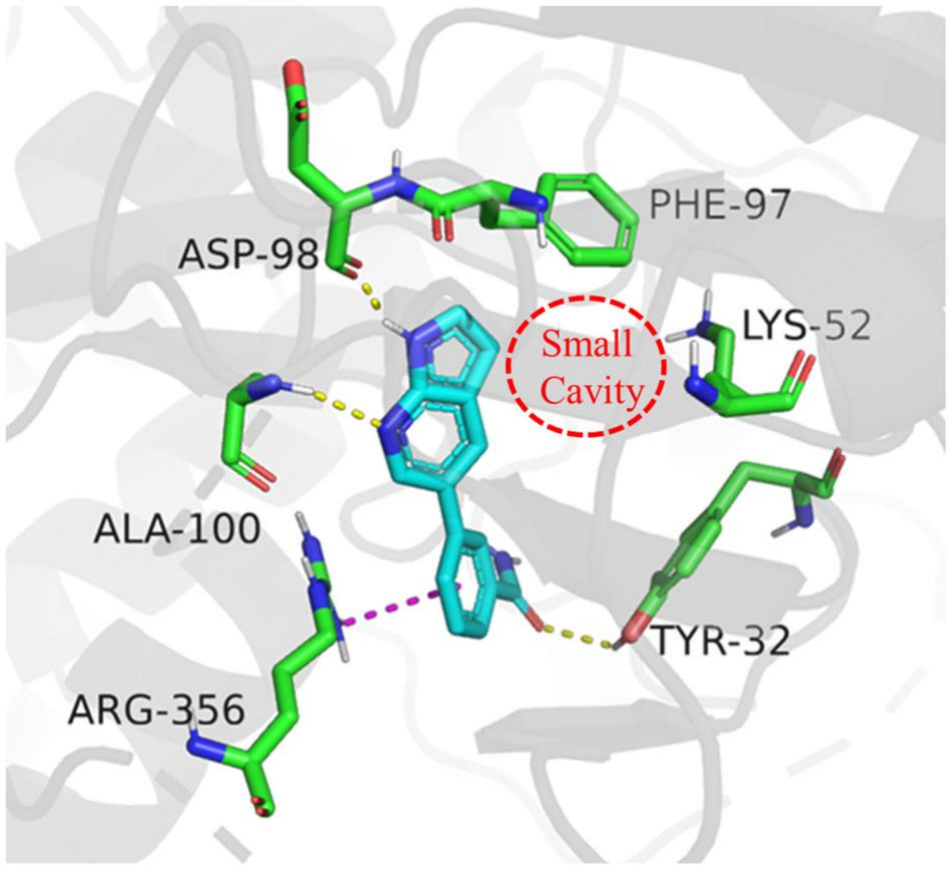
Binding mode of compound **C43** with active site of CDK8 (PDB: 5IDN). CDK8 is shown in gray ribbons with selected residues coloured green. Hydrogen bonds are drawn as yellow dashed lines, and pi-pi stacking is drawn as magenta dashed lines. Compound **43** is shown with blue stick. The illustration was generated using PyMOL.

In order to further optimise on the hit compound **C43**, we analysed the docking model of it with CDK8. As shown in Figure 2, we noticed that the azaindole of compound **C43** forms hydrogen-bonding interactions with amino acid residues Asp98 and Ala100. The benzamide fragment forms cation-pi stacking with amino acid residue Arg356 and hydrogen-bonding interaction with the hydroxyl group of Tyr32. Importantly, there is a small cavity near the 3-position of the azaindole ring, which consists of the phenyl ring of the amino acid residue Phe97 and the amino group of Lys52 (Figure 2). In addition, based on the previous SAR^21^, we found that the activity of compounds that were introduced into other groups in the small cavity was significantly reduced, only compounds **C5** and **C8** which with 3-furyl and phenyl still had good activity, with IC_50_ values of 85.1 ± 5.0 nM and 60.3 ± 1.2 nM, respectively. Therefore, we believe that the introduction of aromatic heterocycle or benzene ring derivatives at the 3-position of the azaindole ring can enhance the activity by forming hydrophobic interactions, pi-pi stacking interactions or hydrogen bonding interactions.

### 2.2. Chemistry

**Scheme 1.**
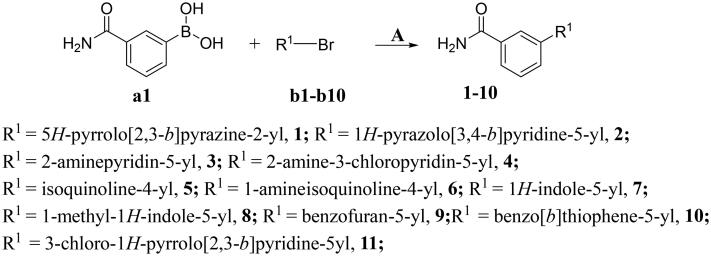
Synthesis of compounds **1-11**^a^ **^a^Reagents and conditions**: **A**. K_2_CO_3_, Pd(dppf)Cl_2_, 1,4-dioxne, H_2_O, 85 °C, 14 h.

**Scheme 2.**
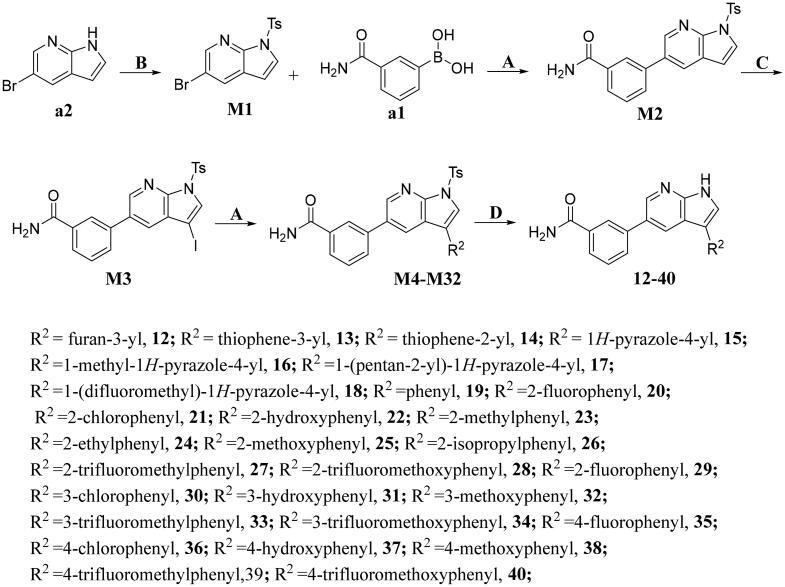
Synthesis of compounds **12-40**^a^ **^a^Reagents and conditions**: **A**. K_2_CO_3_, Pd(dppf)Cl_2_, 1,4-dioxne, H_2_O, 85 °C, 14 h; **B**. NaOH, THF, H_2_O, 35 °C, 6 h; **C**. DMF, NIS, 85 °C, 12 h; **D**. NaOH, CH_3_CH_2_OH, H_2_O, 75 °C, 2 h.

The preparation of title compounds **1-40** was described in Schemes 1–2. As shown in Scheme 1, compounds **1-11** were obtained through Suzuki Reaction. As shown in Scheme 2, compounds **12-40** were obtained through 5-step reaction. (1) The nitrogen atom of 5-bromo-7-azaindole was protected by *p*-methylbenzenesulfonyl to obtain compound **M1**; (2) The key intermediate **M2** was synthesised by Suzuki Reaction; (3) Compound **M2** went through Halogenated Reaction to obtain compound **M3**; (4) The key intermediate **M4** was prepared through Suzuki-Miyaura reaction and the synthesis method of compounds **M5-M32** was the same as compound **M4**; (5) *p*-methylbenzenesulfonyl of compound **M4** was removed under the condition of sodium hydroxide at 75 °C to obtain compounds **12**, the synthesis method of compounds **13-40** was the same as compound **12**.

### SAR study

A total of 40 compounds were designed and synthesised, and their CDK8 inhibition rates at 200 nM were screened. Some of them with high inhibition rates were further measured for their IC_50_ values. Compound SEL-120 34 A was selected as a positive control.

First, in order to determine whether the previous 7-azaindole is still a good skeleton after the introduction of the amide group, compounds **1-10** were designed and synthesised. As shown in Table 1, their activity was significantly reduced, except for compound **6**, with the IC_50_ value of 64.5 ± 3.8 nM, showing that 7-azaindole is still a good skeleton.

**Table 1.** The evaluation of the activity of compounds **1-10** on CDK8.

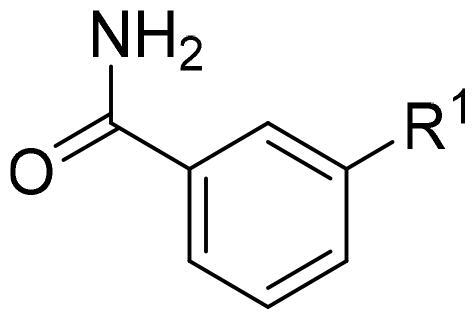			
Compounds	R^1^	Inhibition rate @ 200 nM^*a*^ (%)^*a*^	CDK8 IC_50_ (nM)^*b*^
1	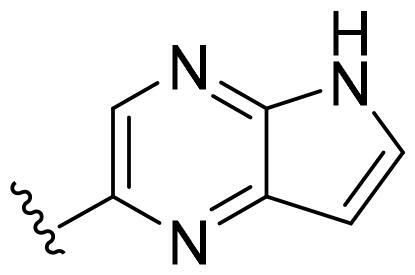	63.0 ± 4.5	105.9 ± 4.6
2	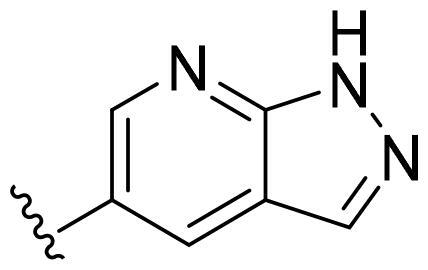	35.2 ± 2.8	NT*^c^*
3	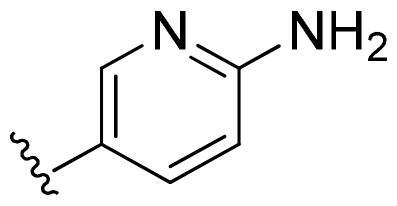	50.8 ± 2.3	176.5 ± 7.2
4	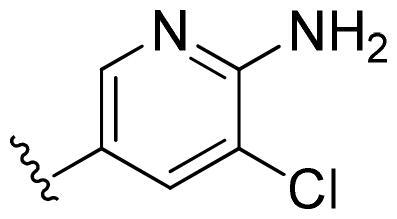	45.6 ± 4.2	NT
5	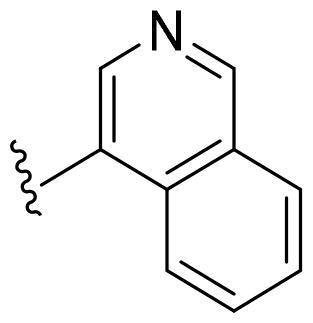	55.7 ± 3.8	186.3 ± 4.5
6	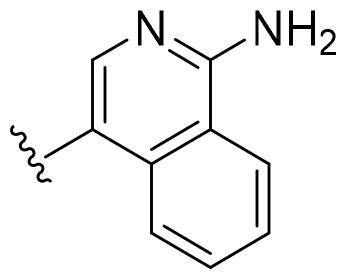	75.8 ± 5.9	64.5 ± 3.8
7	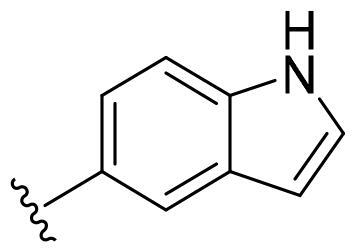	59.1 ± 2.1	212.3 ± 6.1
8	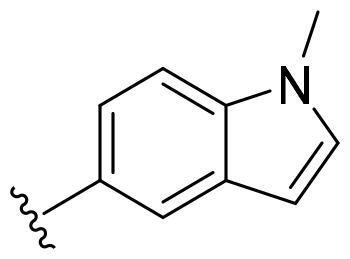	51.7 ± 5.4	255.3 ± 4.5
9	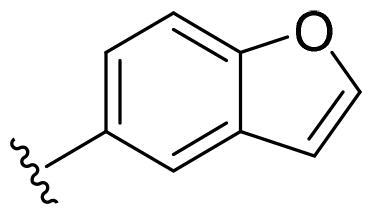	60.4 ± 4.3	189.6 ± 4.9
10	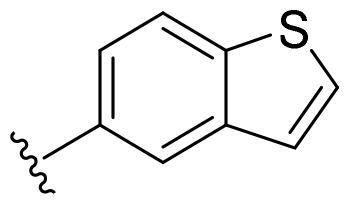	38.3 ± 2.2	NT
SEL-120-34A	/	82.5 ± 5.8	37.2 ± 2.5
C43	/	73.2 ± 4.3	52.6 ± 3.5

^a^ CDK8 inhibition rate at 200 nM was determined by CDK8 enzyme activity assay. Values from two experiments and results were shown as means ± SD.

^b^ IC_50_ values were determined by CDK8 enzyme activity assay. Values from three dependent experiments and results were shown as means ± SD. ^c^NT: Not Test.

Based on these, compounds **11-41** were designed and synthesised through introducing chlorine atom, aromatic heterocycle or benzene ring derivatives. As shown **in** Table 2, the activity of compound **12** with 3-furyl is the most active and significantly higher than that of **C43**, with an IC_50_ value of 39.2 ± 6.3 nM. When 3-furyl was replaced by 3-thienyl to give compound **13**, its activity decreased a little, with an IC_50_ value of 55.7 ± 3.0 nM. The activity of other compounds decreased, especially compound **17**, its activity decreased significantly, due to the introduction of 3-methylimidazolyl group, it has a large steric hindrance, which is the reason for the decreased activity. There is a significant difference in activity between compounds **20-41**, showing that the size of the functional group and the electron cloud density of the aromatic ring have a significant impact on the activity.

**Table 2.** The evaluation of the activity of compounds **11-40** on CDK8.

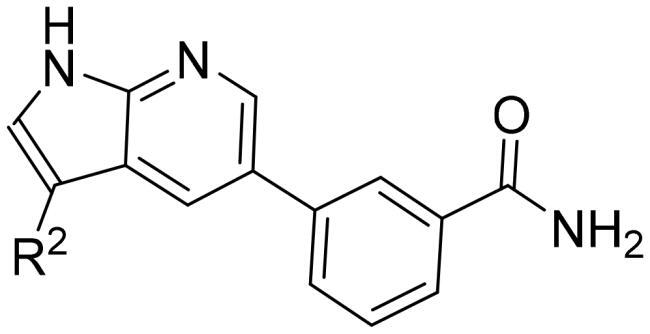			
Compounds	R^2^	Inhibition rate @ 200 nM (%)^*a*^	CDK8 IC_50_ (nM)^*b*^
11	Cl	51.8 ± 4.3	NT*^c^*
12	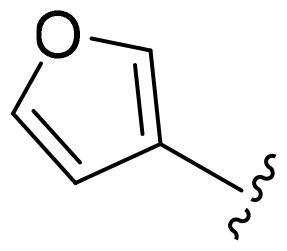	80.9 ± 3.5	39.2 ± 6.3
13	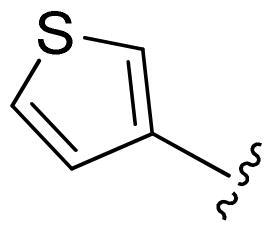	70.4 ± 3.6	65.7 ± 3.1
14	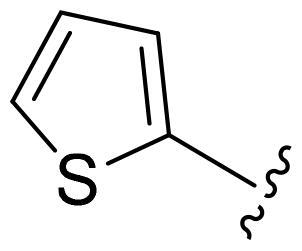	52.5 ± 2.8	185.7 ± 4.6
15	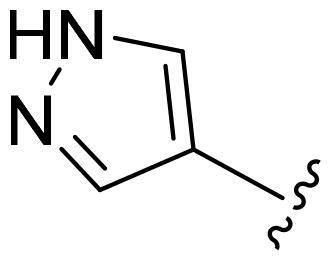	49.1 ± 3.4	NT
16	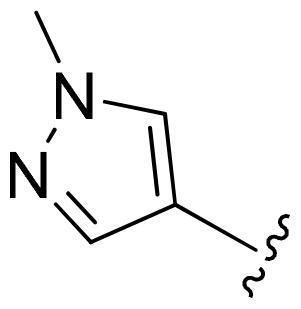	29.5 ± 2.3	NT
17	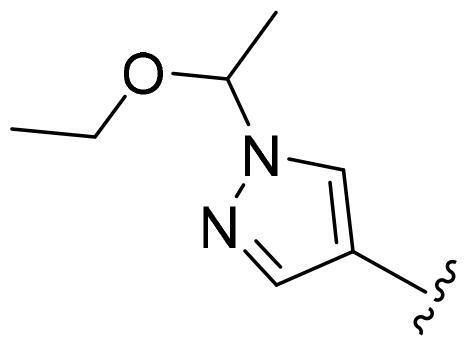	46.5 ± 1.8	NT
18	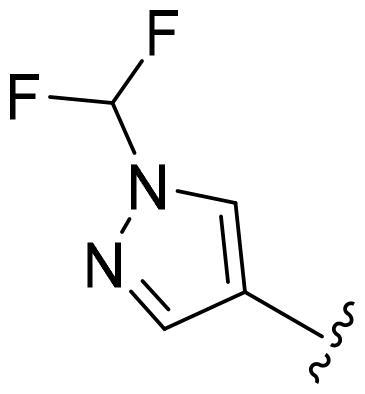	32.5 ± 4.9	NT
19	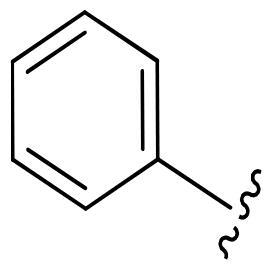	57.4 ± 1.4	157.7 ± 4.3
20	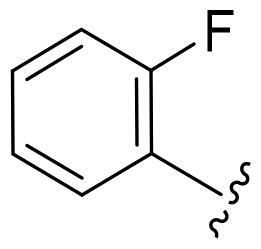	58.7 ± 3.3	160.5 ± 3.1
21	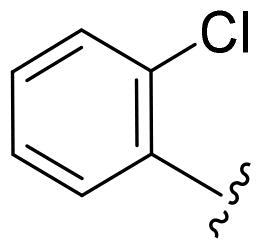	23.6 ± 2.6	NT
22	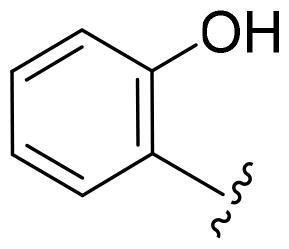	36.2 ± 2.7	NT
23	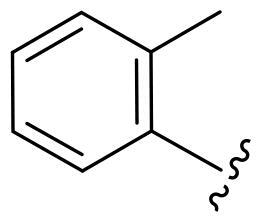	51.6 ± 2.6	192.6 ± 6.6
24	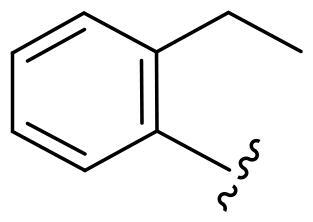	24.6 ± 2.4	NT
25	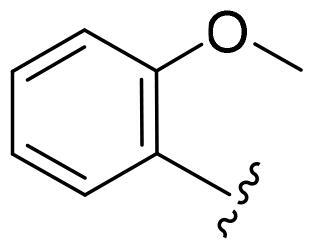	61.98 ± 4.6	135.7 ± 5.3
26	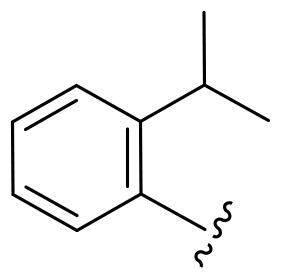	33.1 ± 4.2	NT
27	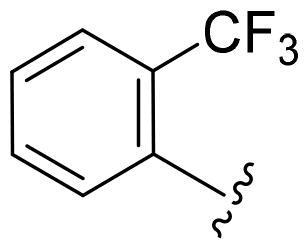	15.9 ± 5.2	NT
28	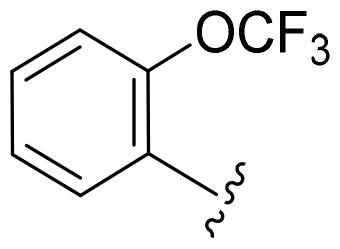	16.9 ± 2.2	NT
29	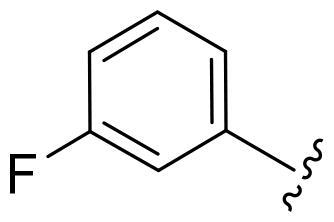	23.1 ± 2.5	NT
30	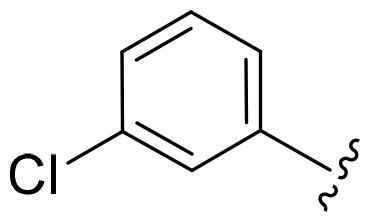	65.5 ± 3.8	86.7 ± 6.5
31	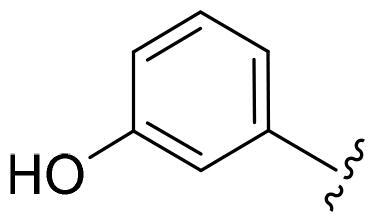	30.5 ± 2.3	NT
32	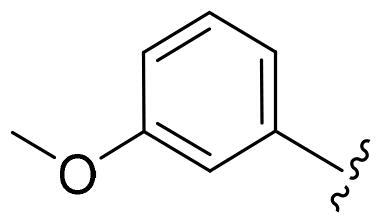	43.97 ± 3.8	NT
33	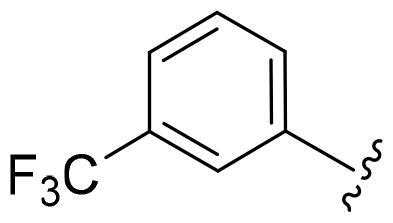	34.6 ± 4.1	NT
34	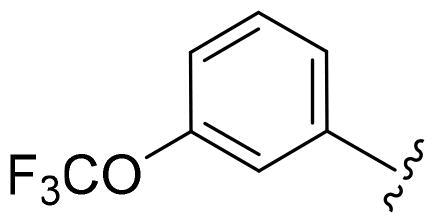	23.4 ± 3.3	NT
35	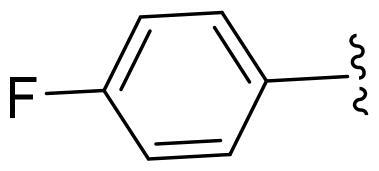	55.8 ± 4.6	NT
36	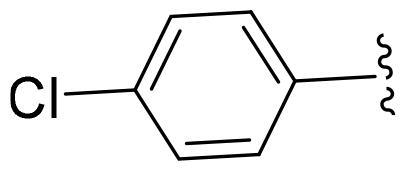	61.6 ± 5.4	127.9 ± 3.8
37	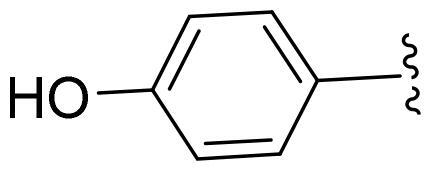	28.3 ± 3.1	NT
38	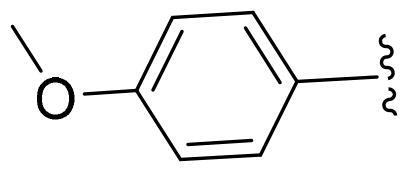	26.42 ± 1.6	NT
39	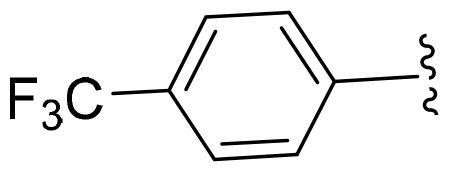	22.3 ± 3.7	NT
40	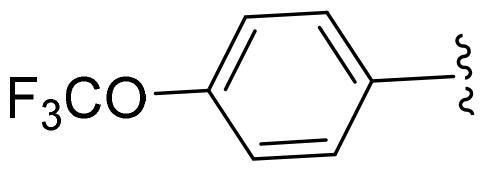	46.3 ± 1.9	NT
SEL-120-34A	/	82.5 ± 5.8	37.2 ± 2.5
C43	/	73.2 ± 4.3	52.6 ± 3.5

^a^ CDK8 inhibition rate at 200 nM was determined by CDK8 enzyme activity assay. Values from two experiments and results were shown as means ± SD.

^b^ IC_50_ values were determined by CDK8 enzyme activity assay. Values from three dependent experiments and results were shown as means ± SD. ^c^NT = Not Test.

**Figure 3.**
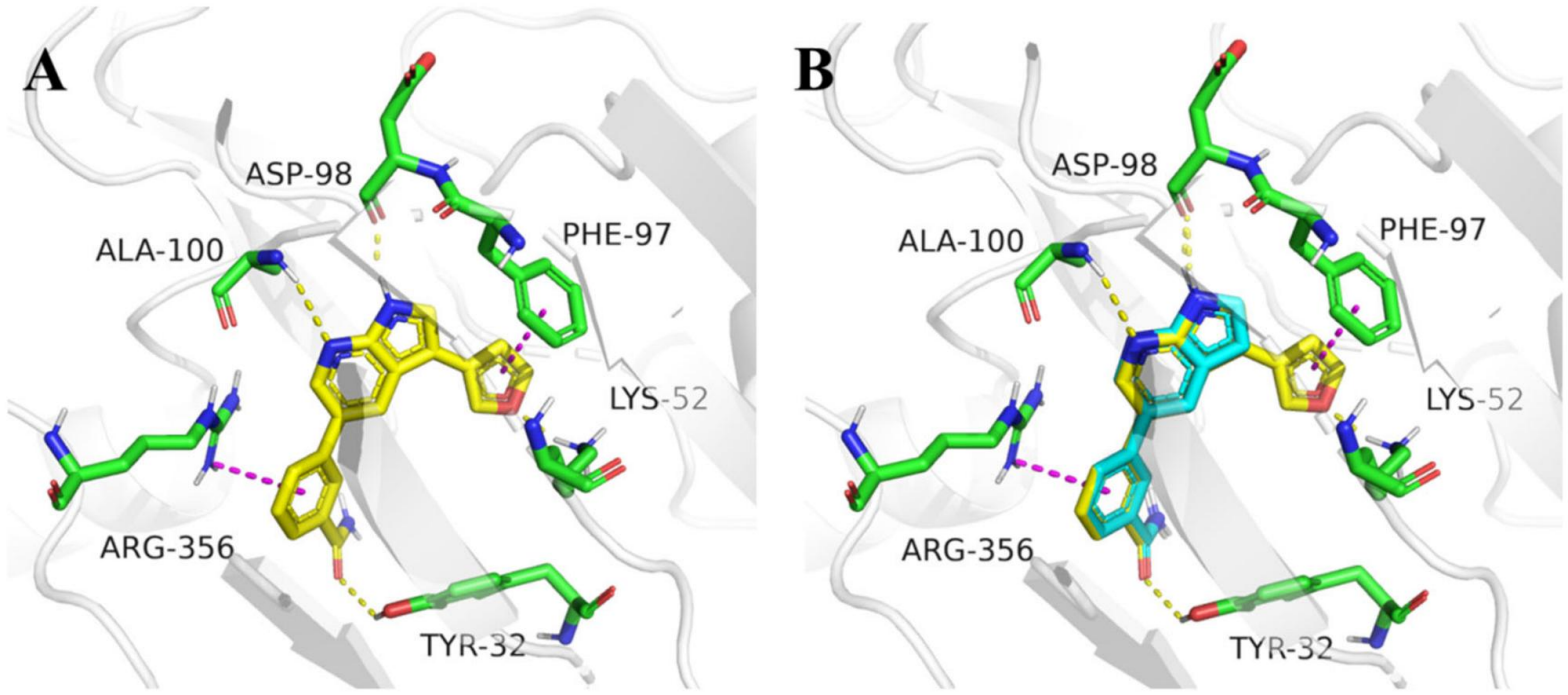
Molecular docking of target compounds. (**A**) The docking model of compound **12** with CDK8 (**B**) Superposition of spatial structures of compound **C43** and **12** within active site of CDK8 (PDB: 5IDN). CDK8 is shown in gray ribbons with selected residues coloured green. Hydrogen bonds are drawn as yellow dashed lines, and pi-pi stacking is drawn as magenta dashed lines. Compound **12** is shown with yellow stick and compound **C43** is shown with blue stick. The illustration was generated using PyMOL.

Finally, for a better overall structure-activity relationship discussion, we analyse and compare the molecular docking of compounds **C43** and **12** (Figure 3B). It is obvious that compound **12** forms more pi-pi stacking interactions and one hydrogen bond interaction than compound **C43**, which is also the reason why compound **12** (CDK8 IC_50_ =39.2 ± 6.3 nM) is more active than compound **C43** (CDK8 IC_50_ =52.6 ± 3.5 nM), but the activity is not greatly improved. The reason was analysed: on the one hand, it may be that the furan ring has a certain steric hindrance, which is not conducive to improving the activity; The significant difference in activity between compounds **17** and **20-41** indirectly proved large spatial hindrance could weaken the activity. On the other hand, it is important that the furan ring is connected to 7-azaindole, and the two aromatic systems may form a large delocalised π bond due to coplanarity, especially affecting the charge distribution of the pyrrole ring results in decreased activity. The significantly lower activity of compound **2** than that of **C43** may be due to the charge change of the pyrrole ring. Therefore, these results are instructive to design novel and highly active CDK8 inhibitors in the future.

### The evaluation of anti-proliferation activity and cytotoxicity of compounds

Compounds**12**, **13** and **30** were selected for further cell activity assessments based on the enzymatic activity assessment results. molm-13 and MV4-11(acute myeloid lineage leukaemia cells), MGC-803 (gastric cancer cell), MDA-MB-231(breast cancer cell), A375 (melanoma cell), A549 (lung cancer cell), HCT-116, SW-480 and HT-29 (colorectal cancer cells) were selected to evaluate antitumor activity *in vitro*, GES-1 (gastric mucosal epithelial cell) was selected to evaluate toxicity *in vitro*. As shown in Table 3, compound **12** and **13** exhibited potent antiproliferative activity on selected tumour cells, especially on AML cells. However, compound **30** did no**t** exhibited potent antiproliferative activity on selected tumour cells.

**Table 3.** Antiproliferative activity and preliminary safety of selected compounds.

Compounds		12	13	30	SEL120-34A
CDK8 IC_50_ (nM) *^a^*	/	39.2 ± 6.3	65.7 ± 3.1	86.7 ± 6.5	37.2 ± 2.5
GC_50_ (*μ*M) *^b^*	molm-13	0.02 ± 0.01	0.16 ± 0.02	>50	0.02 ± 0.01
	MV4-11	0.03 ± 0.01	0.36 ± 0.12	>50	0.007 ± 0.001
	MGC-803	11.72 ± 1.93	9.19 ± 2.42	>50	16.26 ± 1.86
	MDA-MB-231	>50	31.47 ± 1.27	>50	9.60 ± 0.81
	A375	1.00 ± 0.23	0.95 ± 0.52	>50	5.90 ± 1.43
	A549	15.49 ± 2.78	21.75 ± 1.66	>50	12.06 ± 0.37
	HCT-116	3.66 ± 1.94	2.48 ± 0.99	>50	11.3 ± 1.9
	SW480	6.05 ± 2.26	5.45 ± 2.82	>50	17.7 ± 2.1
	HT-29	8.26 ± 0.65	9.31 ± 1.67	>50	33.8 ± 1.4
	GES-1	25.70 ± 4.97	24.15 ± 3.62	>100	55.3 ± 3.2

^a^ IC_50_ values were determined by CDK8 enzyme activity assay. Values from three dependent experiments and results were shown as means ± SD.

^b^ GI_50_ values were determined by CCK-8 assay. Values from three dependent experiments and results were shown as means ± SD.

### CDKs Selectivity of compound 12

CDK7 and CDK9 with the same transcription function and CDK2 and CDK6 were selected to evaluate the selectivity of compound **12**. As shown in Table 4, compound **12** showed good family protein selectivity.

**Table 4.** CDKs Selectivity of Compound 12.

Compound	CDK2 (nM)	CDK6 (nM)	CDK7 (nM)	CDK9 (nM)	CDK8 (nM)
12	>1000	351.9 ± 36.5	301.5 ± 27.8	473.6 ± 25.3	39.2 ± 6.3

### Cellular thermal shift assay

**Figure 4.**
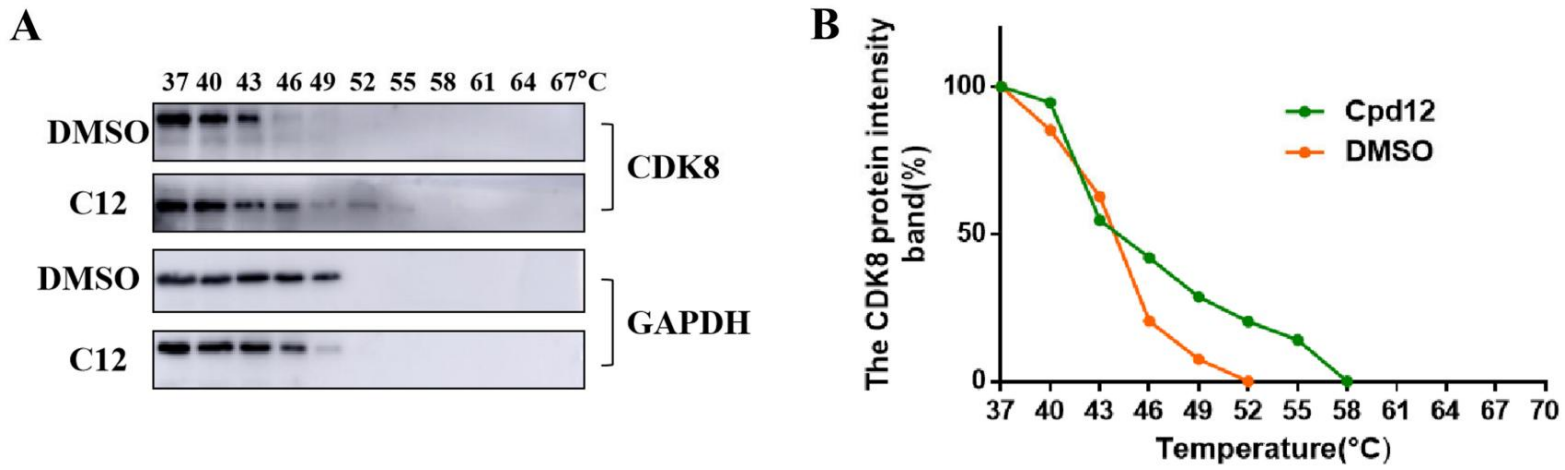
Compound **12** enhanced the thermal stability of intracellular CDK8 protein. (A-B)The CDK8 protein intensity at different temperature.

Cellular thermal shift assay has been recognised as a means of demonstrating the binding of small molecules to target proteins. Here, in order to identify compound **12** could bind CDK8, HCT-116 cells, a CDK8 high expression cell line, were treated with 5 *μ*M compound **12** and DMSO for 6 h, respectively. Cell suspension was divided into 11 PCR tubes equally, then the samples were heated from 37 to 67 °C at intervals of 3 °C and the results were analysed through western blot. As shown in Figure 4, CDK8 was degraded almost completely at 49 °C after treated with DMSO. However, CDK8 was degraded almost completely at 55 °C after treated with compound **12**, which indicated compound **12** enhanced the thermal stability of CDK8 and compound **12** could bind CDK8.

### Compound 12 and biotinylated compound bind to CDK8 competitively

**Figure 5.**
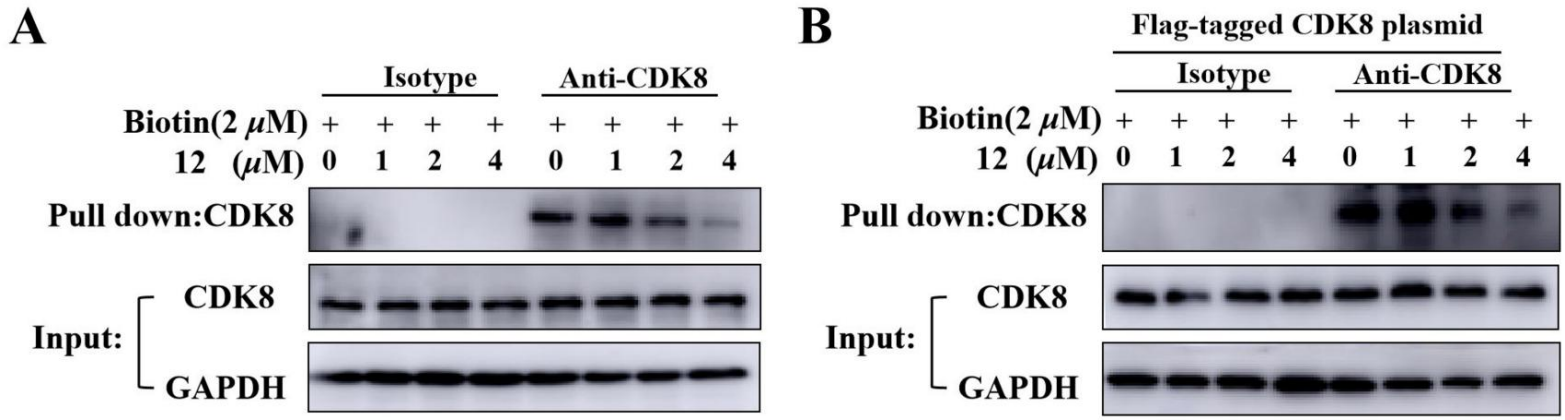
Compound **12** and biotin bind to CDK8 protein competitively. (A) Compound **12** binds to CDK8 protein in HCT-116 cells. (B)Compound **12** binds to CDK8 protein in HEK293T cells.

In previous study, a biotinylated compound with favourable CDK8 inhibition activity was designed and synthesized^23^. Here, the pulldown assay was performed to determine compound **12** could bind CDK8. HCT-116 cells or HEK293T cells transfected with CDK8 plasmid labelled were treated with 2 *μ*M biotinylated compound and compounds **12** with different concentrations of 1 *μ*M, 2 *μ*M, 4 *μ*M separately. As shown in Figure 5, the amount of protein pulled down by biotinylated compound decreased as the concentration of compound **12** increased, which indicated that compound **12** could inhibit the binding of biotinylated compound to CDK8, indirectly proving that the compound **12** could bind CDK8.

### Effect of compound 12 on phosphorylation of STAT1 S727 and STAT5 S726

**Figure 6.**
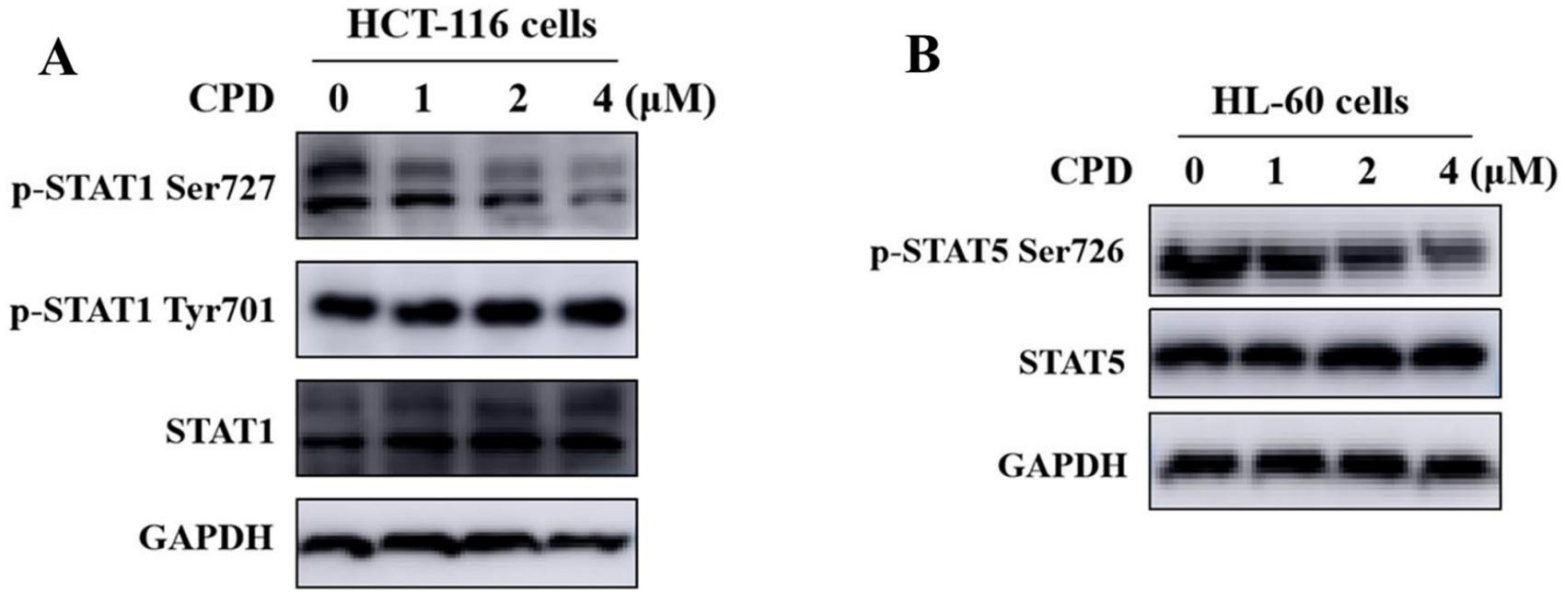
Compound **12** inhibited phosphorylation of STAT1 S727 and STAT5 S726. (A) Compound **12** suppressed the phosphorylation of STAT1 S727 in a dose-dependent manner. HCT-116 cells were treated with compound for 12 h. (B) Compound **12** suppressed the phosphorylation of STAT5 S726 in a dose dependent manner. HL-60 cells were treated with or without compound for 12 h. The samples were analysed by Western blot.

It has been reported that CDK8 could specifically target the transactivation domain of STAT1 at Ser727 phosphorylation^24^. HCT-116 cells were treated with compounds **12** with different concentrations of 1 *μ*M, 2 *μ*M, 4 *μ*M separately to verify compound **12** could inhibit the phosphorylation of STAT1 Ser727. As shown in Figure 6(A), after treating cells with compound **12**, the phosphorylation of STAT1 S727 was significantly inhibited in a dose-dependent manner, while JAK-mediated the phosphorylation of STAT1 Tyr701 was not inhibited. Above results indicated that compound **12** could affect the biological function of CDK8. It’s reported that CDK8 was also involved in S726 phosphorylation of STAT5 in AML cells^7^. As shown in Figure 6(B), compound **12** could inhibit phosphorylation of STAT5 S726 in a dose-dependent manner.

### In vivo pharmacokinetic evaluation

**Figure 7.**
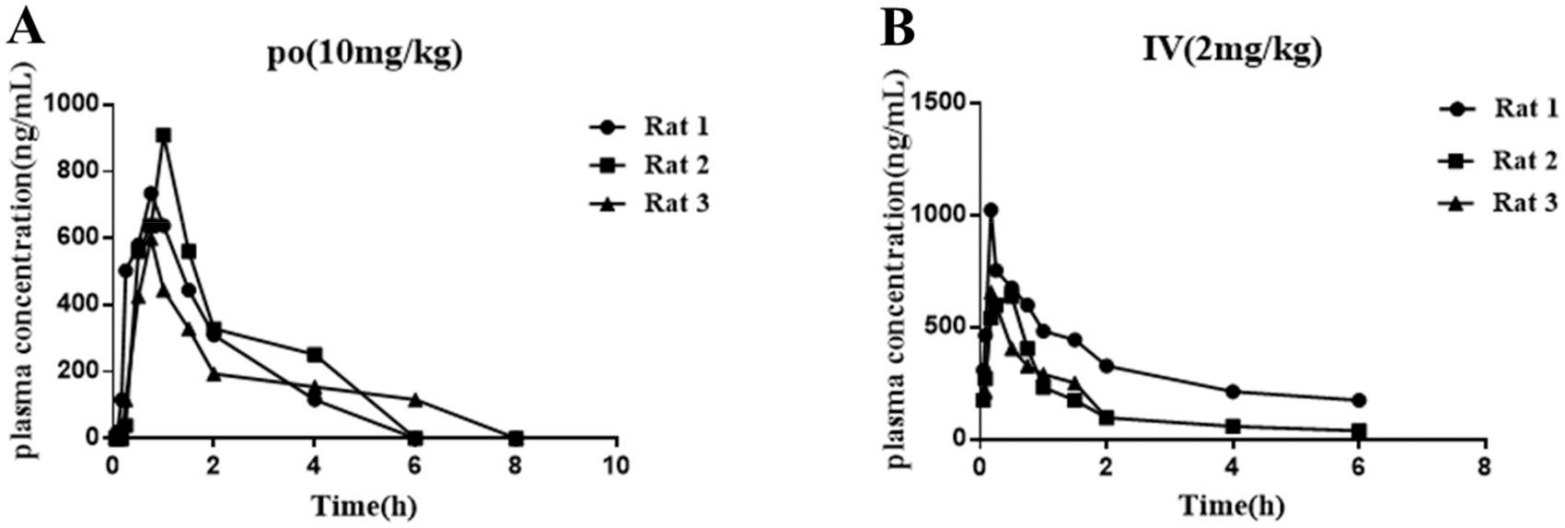
**(A-B)** The concentrations versus time curve of compound **12**.

Pharmacokinetic data is an important parameter for small molecule drugs. Here, the pharmacokinetic data of compound **12** was performed. As shown in Table 5, after oral administration of compound **12** at 10 mg/kg, there was a good blood drug concentration (AUC_0−∞_ = 1611.6 *μ*g/L × h). In addition, we noticed that the C_max_ was 821.8 *μ*g/L at 0.88 h and t_1/2_ was 0.95 h, which indicated that compound **12** exhibietd acceptable pharmacokinetic properties. After *iv* administration of compound **12** at 2 mg/kg, the C_max_ was 647.7 *μ*g/L, the half-time (t_1/2_) was about 1.12 h and the area under the concentration time curve (AUC_0−∞_) was 829.7 *μ*g/L × h. Importantly, compound **12** exhibited relatively satisfied bioavailability (*F*%= 38.8%). In addition, compound **12** showed high permeability and no obvious inhibition activity against five cytochrome p450 isoenzymes (Tables 6 and 7), which indicated that compound **12** may be suitable for co-administering with other drugs. Based on the above data analysis, we believed it is possible that compound **12** could be used as an oral medication (Figure 7).

**Table 5.** *In vivo* PK properties of compound 12.

Dose/routes	t_1/2_ (h)	T_max_ (h)	MRT (h)	*C*_max_ (*μ*g/L)	AUC _0-∞_ (*μ*g/L × h)	CL(L/h/kg)	*F*(%)
10 mg/kg (*po*)	0.95	0.88	2.24	821.82	1611.67	6.21	38.80

2 mg/kg (iv)	1.12	0.33	1.64	647.79	829.75	2.45	/


**Table 6.** Caco-2 permeability determination.

Compound	P_app_ (10 ^−6^ cm/s)		Efflux ratio	Recovery (%)	
	A→B	B→A		A→B	B→A
12	27.0	35.1	1.3	107.0	108.1
Nadolol	0.2	0.5	–	99.0	110.9
Propranolol	40.2	34.1	–	99.9	107.0
Digoxin	0.9	5.6	6.2	92.5	99.2

**Table 7.** CYP inhibition activity.

Compound	IC_50_ (*μ*M)				
	1A2	2C9	2C19	2D6	3A4/5 (M)
12	>50	>50	>50	>50	>50
Positive control	0.209	0.569	9.30	0.0356	0.0118

### Acute toxicity study

**Figure 8.**
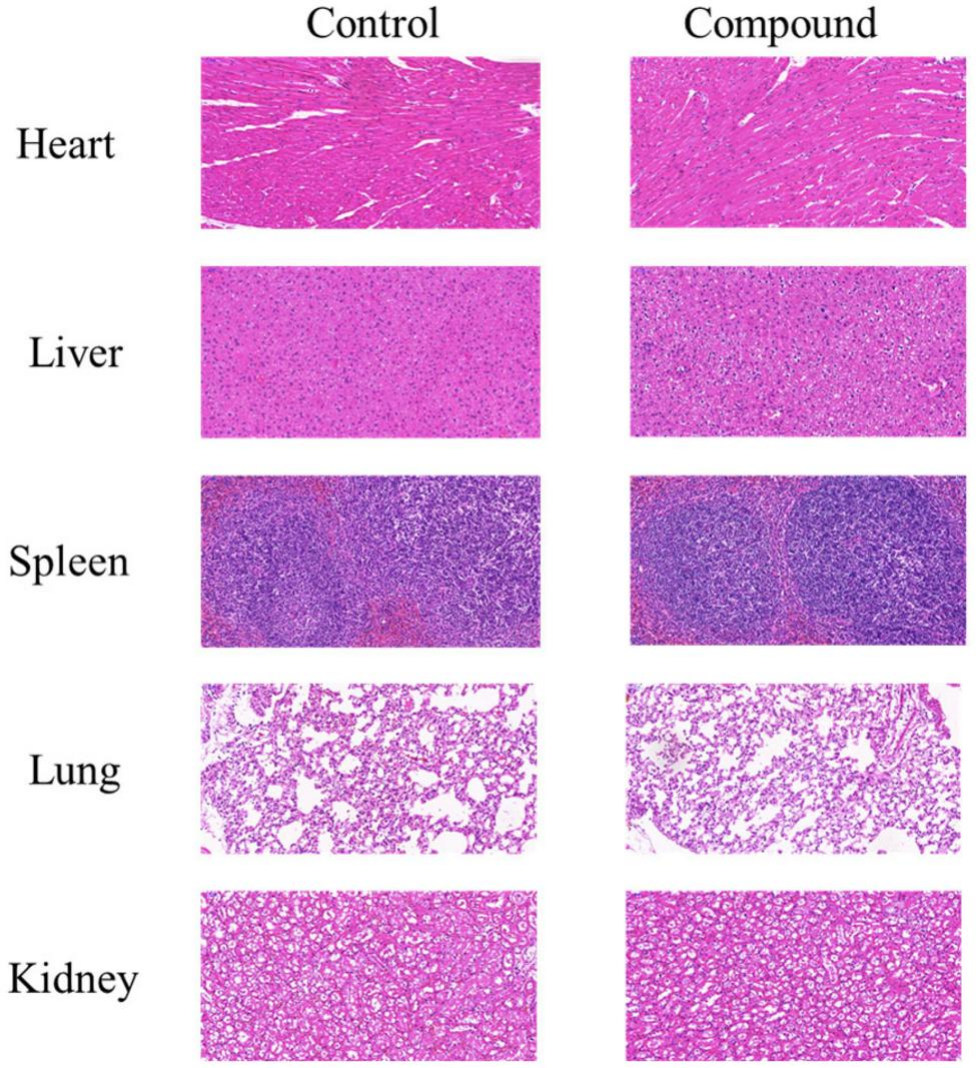
Compound **12** had low toxicity in *vivo*. The HE staining of tissues after mice were treated with 1000 mg/kg compound **12**. The scan bar: 100 *μ*M.

The safety of compound **12** *in vivo* was evaluated through an acute toxicity study on C57BL/6 mice. The compound **12** was administered by gastric tube at a dose of 1000 mg/kg. The normal group was given the same amount of normal saline. During the whole 7 days, there is no obvious change in physiological phenomena such as anorexia, drowsiness, seizures and hyperactivity. Finally, the mice were anaesthetised and sacrificed, and the main organs such as heart, liver, spleen, lung and kidney were detected through pathological section. As shown in Figure 8, there were no diseased tissue, indicating good safety of compound **12** *in vivo*.

## Conclusion

STAT1, STAT3 and STAT5 are constitutively activated in AML cell lines. CDK8 can positively regulate phosphorylation of S727 in STAT1 and S726 in STAT5 in AML cells. SEL120-34A (SEL120), a clinical trial phase I CDK8 inhibitor, has been shown to downregulate phosphorylation level of STAT1 and STAT5 in AML cells lines. Thus, the synergistic effects of STAT inhibitor with CDK8 inhibitor could provide new therapeutic opportunities for AML, rather than reverse the effects of the CDK8 inhibitor. Based on the previous work, a total of 40 compounds were designed and synthesised through rational design and discussion on structure-activity relationships. Through comprehensive evaluation, compound **12** (*3–(3-(furan-3-yl)-1H-pyrrolo[2,3-b]pyridin-5-yl)benzamide*) was selected as potent CDK8 inhibitor (CDK8 IC_50_ = 39.2 ± 6.3 nM) for further research. The studies showed that compound **12** could bind the CDK8 and inhibit the phosphorylation of the STAT 1 Ser 727 and STAT 5 Ser 726. compound **12** showed good antiproliferative activity against AML cells at nanomolar concentrations. Importantly, compound **12** exhibited acceptable pharmacokinetic properties that could be taken orally. This study has guiding significance for the development of novel and efficient CDK8 inhibitors.

## Supporting information

^1^H NMR,^13^C NMR and HR-MS spectra.

## Supplementary Material

Supplemental Material

## References

[1] Yogarajah M, Stone RM. A concise review of BCL-2 inhibition in acute myeloid leukemia. Expert Rev Hematol. 2018;11(2):145–154. 29264938 10.1080/17474086.2018.1420474

[2] Cao Z, Shu Y, Wang J, Wang C, Feng T, Yang L, Shao J, Zou L. Super enhancers: Pathogenic roles and potential therapeutic targets for acute myeloid leukemia (AML). Genes Dis. 2022;9(6):1466–1477. 36157504 10.1016/j.gendis.2022.01.006 PMC9485276

[3] Kayser S, Levis MJ. Updates on targeted therapies for acute myeloid leukaemia. Br J Haematol. 2022;196(2):316–328. 34350585 10.1111/bjh.17746

[4] Thol F, Ganser A. Treatment of relapsed acute myeloid leukemia. Curr Treat Options Oncol. 2020;21(8):66. 32601974 10.1007/s11864-020-00765-5 PMC7324428

[5] Hosono N, Yokoyama H, Aotsuka N, Ando K, Iida H, Ishikawa T, Usuki K, Onozawa M, Kizaki M, Kubo K, et al. Gilteritinib versus chemotherapy in Japanese patients with FLT3-mutated relapsed/refractory acute myeloid leukemia. Int J Clin Oncol. 2021;26(11):2131–2141. 34363558 10.1007/s10147-021-02006-7 PMC8522999

[6] Brachet-Botineau M, Deynoux M, Vallet N, Polomski M, Juen L, Hérault O, Mazurier F, Viaud-Massuard MC, Prié G, Gouilleux F. A novel inhibitor of STAT5 signaling overcomes chemotherapy resistance in myeloid leukemia cells. Cancers. 2019;11(12):2043. 31861239 10.3390/cancers11122043 PMC6966442

[7] Rzymski T, Mikula M, Żyłkiewicz E, Dreas A, Wiklik K, Gołas A, Wójcik K, Masiejczyk M, Wróbel A, Dolata I, et al. SEL120-34A is a novel CDK8 inhibitor active in AML cells with high levels of serine phosphorylation of STAT1 and STAT5 transactivation domains. Oncotarget. 2017;8(20):33779–33795. 28422713 10.18632/oncotarget.16810 PMC5464911

[8] Wingelhofer B, Maurer B, Heyes EC, Cumaraswamy AA, Berger-Becvar A, de Araujo ED, Orlova A, Freund P, Ruge F, Park J, et al. Pharmacologic inhibition of STAT5 in acute myeloid leukemia. Leukemia. 2018;32(5):1135–1146. 29472718 10.1038/s41375-017-0005-9 PMC5940656

[9] Yoshimoto G, Miyamoto T, Jabbarzadeh-Tabrizi S, Iino T, Rocnik JL, Kikushige Y, Mori Y, Shima T, Iwasaki H, Takenaka K, et al. FLT3-ITD up-regulates MCL-1 to promote survival of stem cells in acute myeloid leukemia via FLT3-ITD-specific STAT5 activation. Blood. 2009;114(24):5034–5043. 19808698 10.1182/blood-2008-12-196055 PMC2788977

[10] Cee VJ, Chen DY, Lee MR, Nicolaou KC. Cortistatin A is a high-affinity ligand of protein kinases ROCK, CDK8, and CDK11. Angew Chem Int Ed Engl. 2009;48(47):8952–8957. 19844931 10.1002/anie.200904778

[11] Yu M, Long Y, Yang Y, Li M, Teo T, Noll B, Philip S, Wang S. Discovery of a potent, highly selective, and orally bioavailable inhibitor of CDK8 through a structure-based optimisation. Eur J Med Chem. 2021;218:113391. 33823391 10.1016/j.ejmech.2021.113391

[12] Lee JC, Liu S, Wang Y, Liang Y, Jablons DM. MK256 is a novel CDK8 inhibitor with potent antitumor activity in AML through downregulation of the STAT pathway. Oncotarget. 2022;13(1):1217–1236. 36342456 10.18632/oncotarget.28305 PMC9629815

[13] Dale T, Clarke PA, Esdar C, Waalboer D, Adeniji-Popoola O, Ortiz-Ruiz MJ, Mallinger A, Samant RS, Czodrowski P, Musil D, et al. A selective chemical probe for exploring the role of CDK8 and CDK19 in human disease. Nat Chem Biol. 2015;11(12):973–980. 26502155 10.1038/nchembio.1952 PMC4677459

[14] Mallinger A, Schiemann K, Rink C, Stieber F, Calderini M, Crumpler S, Stubbs M, Adeniji-Popoola O, Poeschke O, Busch M, et al. Discovery of potent, selective, and orally bioavailable small-molecule modulators of the mediator complex-associated kinases CDK8 and CDK19. J Med Chem. 2016;59(3):1078–1101. 26796641 10.1021/acs.jmedchem.5b01685 PMC5362750

[15] Czodrowski P, Mallinger A, Wienke D, Esdar C, Pöschke O, Busch M, Rohdich F, Eccles SA, Ortiz-Ruiz MJ, Schneider R, et al. Structure-based optimization of potent, selective, and orally bioavailable CDK8 inhibitors discovered by high-throughput screening. J Med Chem. 2016;59(20):9337–9349. 27490956 10.1021/acs.jmedchem.6b00597

[16] Hofmann MH, Mani R, Engelhardt H, Impagnatiello MA, Carotta S, Kerenyi M, Lorenzo-Herrero S, Böttcher J, Scharn D, Arnhof H, et al. Selective and potent CDK8/19 inhibitors enhance NK-cell activity and promote tumor surveillance. Mol Cancer Ther. 2020;19(4):1018–1030. 32024684 10.1158/1535-7163.MCT-19-0789 PMC7661742

[17] Boehringer Ingelheim GmbH. New phenyl pyrazolyl acetamide compounds and derivatives as CDK8/CDK19 inhibitors [P]. WO2017202719A1, 2017.11.30.

[18] NIH. U.S. National Library of Medicine. https://clinicaltrials.gov. (accessed May 25, 2023).

[19] Yu M, Teo T, Yang Y, Li M, Long Y, Philip S, Noll B, Heinemann GK, Diab S, Eldi P, et al. Potent and orally bioavailable CDK8 inhibitors: design, synthesis, structure-activity relationship analysis and biological evaluation. Eur J Med Chem. 2021;214:113248. 33571827 10.1016/j.ejmech.2021.113248

[20] Solum E, Hansen TV, Aesoy R, Herfindal L. New CDK8 inhibitors as potential anti-leukemic agents - design, synthesis and biological evaluation. Bioorg Med Chem. 2020;28(10):115461. 32245563 10.1016/j.bmc.2020.115461

[21] Zhang XX, Yan YY, Ma X, Xiao Y, Lei CC, Wang YM, Liu C, Wang Q, Zhang XT, Cheng WD, et al. Discovery of a novel oral type I CDK8 inhibitor against acute myeloid leukemia. Eur J Med Chem. 2023;251:115214. 36889252 10.1016/j.ejmech.2023.115214

[22] Xie Z, Hou S, Yang X, Duan Y, Han J, Wang Q, Liao C. Lessons learned from past cyclin-dependent kinase drug discovery efforts. J Med Chem. 2022;65(9):6356–6389. 35235745 10.1021/acs.jmedchem.1c02190

[23] Yan YY, Zhang XX, Xiao Y, Shen XB, Jian YJ, Wang YM, She ZH, Liu MM, Liu XH. Design and synthesis of a 2-amino-pyridine derivative as a potent CDK8 inhibitor for anti-colorectal cancer therapy. J Med Chem. 2022;65(19):13216–13239. 36126227 10.1021/acs.jmedchem.2c01042

[24] Bancerek J, Poss ZC, Steinparzer I, Sedlyarov V, Pfaffenwimmer T, Mikulic I, Dölken L, Strobl B, Müller M, Taatjes DJ, et al. CDK8 kinase phosphorylates transcription factor STAT1 to selectively regulate the interferon response. Immunity. 2013;38(2):250–262. 23352233 10.1016/j.immuni.2012.10.017 PMC3580287

