# Supplementary material for: Discovery of novel and potent CDK8 inhibitors for the treatment of acute myeloid leukaemia
Source: J Enzyme Inhib Med Chem. 2024 Jan 23;39(1):2305852. doi: 10.1080/14756366.2024.2305852 (PMC10810651; doi:10.1080/14756366.2024.2305852)

Discovery of novel and potent CDK8 inhibitors for the treatment of acute myeloid leukemia

Zhuoying Chen[§, ‡], Quan Wang[§, ‡], Yao Yao Yan[‡], Dalong Jin[‡], Yumeng Wang[‡],
Xing Xing Zhang*[,†], Xin Hua Liu*[,‡]

[‡] *School of Pharmacy, Anhui Medical University, Hefei, 230032, P. R. China*

[†]*School of Biology, Food and Environment, Hefei University, Hefei, 230601, China*

Contents

1. **Experimental section ……………………………………………………..…S1**

2. **^{1}H NMR, ^{13}C NMR, HRMS…………………………………………………S2**

1. Experimental section

1.1 General methods for chemistry

Reactions were monitored by thin layer chromatography (TLC) on pre-coated silica GF_{254} plates. ^{1}H and ^{13}C NMR spectra are obtained by Agilent DD2 500 MHz (^{1}H, 500 MHz; ^{13}C, 126 MHz) spectrometer with DMSO-d_6 as the solvents and TMS as the internal standard. High-resolution mass spectrometry (HRMS) was recorded on an Agilent Technologies LC-TOF instrument.

General procedure for synthesis of compound **1**

Compound **a1** (165 mg, 1.0 mmol), compound **b1** (238 mg, 1.2 mmol), K_2CO_3 (552 mg, 4.00 mmol), Pd(dppf)Cl_2 (72 mg, 0.1 mmol), 1,4-dioxne (15 mL) and H_2O (4 mL) were added into flask, then stirred at 80 °C for 20 h under nitrogen. After the reaction was completed, it was added ethyl acetate and H_2O, then the mixture was extracted with ethyl acetate (60 mL×2), washed with saturated brine solution, dried over anhydrous Na_2SO_4, and concentrated to give the yellow solid, then purified by chromatography (gradient elution of PE/EA 10/90, v/v) to obtain compound **1.**

3-(5H-pyrrolo[2,3-b]pyrazin-2-yl)benzamide **(1)**

^{1}H NMR (500 MHz, DMSO-d_6) δ 12.14 (s, 1H), 8.95 (s, 1H), 8.66 (s, 1H), 8.30 (d, J = 6.7 Hz, 1H), 8.17 (s, 1H), 7.95 (s, 2H), 7.62 (t, J = 7.0 Hz, 1H), 7.47 (s, 1H), 6.74 (s, 1H). ^{13}C NMR (126 MHz, DMSO-d_6) δ 168.31, 145.38, 141.11, 139.01, 138.43, 135.40, 135.21, 132.61, 129.59, 129.30, 127.95, 125.98, 101.16. HRMS (ESI): *m/z* $[M+H]^+$ calcd for $C_{13}H_{10}N_4O$:239.0927; found:239.0927.

General procedure for synthesis of compounds **2-11**

Compounds **2-11** were obtained according to the similar procedures with compound **1.**

3-(1H-pyrazolo[3,4-b]pyridin-5-yl)benzamide **(2)**

^{1}H NMR (400 MHz, DMSO-d_6) δ 13.76 (s, 1H), 8.92 (d, J = 2.2 Hz, 1H), 8.56 (d, J = 2.1 Hz, 1H), 8.28 – 8.20 (m, 2H), 8.13 (s, 1H), 7.91 (td, J = 8.2, 1.5 Hz, 2H), 7.59 (t, J = 7.7 Hz, 1H), 7.47 (s, 1H). ^{13}C NMR (101 MHz, DMSO-d_6) δ 168.15, 151.80, 148.61, 138.42, 135.54, 134.16, 130.24, 129.60, 129.23, 128.29, 127.07, 126.45, 115.03. HRMS (ESI): *m/z* $[M+H]^+$ calcd for $C_{13}H_{10}N_4O$:239.0927; found:239.0929.

3-(6-aminopyridin-3-yl)benzamide **(3)**

^{1}H NMR (400 MHz, DMSO-*d*$_6$) δ 8.33 (d, *J* = 2.2 Hz, 1H), 8.09 (s, 1H), 8.07 (s, 1H), 7.77 (dd, *J* = 8.6, 2.5 Hz, 2H), 7.72 (d, *J* = 8.2 Hz, 1H), 7.48 (t, *J* = 7.7 Hz, 1H), 7.44 (s, 1H), 6.55 (d, *J* = 8.6 Hz, 1H), 6.15 (s, 2H). ^{13}C NMR (101 MHz, DMSO-*d*$_6$) δ 168.39, 159.86, 146.42, 138.55, 135.91, 135.31, 129.34, 128.45, 125.91, 124.65, 123.70, 108.42. HRMS (ESI): *m/z* $[M+H]^+$ calcd for $C_{12}H_{11}N_3O$:214.0975; found:214.0974.

3-(6-amino-5-chloropyridin-3-yl)benzamide **(4)**

^{1}H NMR (400 MHz, DMSO-*d*$_6$) δ 8.36 (d, *J* = 2.1 Hz, 1H), 8.11 (s, 2H), 8.02 (d, *J* = 2.2 Hz, 1H), 7.80 (ddd, *J* = 6.6, 4.1, 1.6 Hz, 2H), 7.50 (t, *J* = 7.8 Hz, 1H), 7.46 (s, 1H), 6.51 (s, 2H). ^{13}C NMR (101 MHz, DMSO-*d*$_6$) δ 168.20, 155.53, 144.90, 136.99, 135.35, 135.10, 129.45, 128.75, 126.58, 125.32, 124.87, 114.08. HRMS (ESI): *m/z* $[M+H]^+$ calcd for $C_{12}H_{10}N_3OCl$:239.0927; found:248.0583.

3-(isoquinolin-4-yl)benzamide **(5)**

^{1}H NMR (500 MHz, DMSO-*d6*) δ 9.39 (s, 1H), 8.51 (s, 1H), 8.25 (d, *J* = 8.1 Hz, 1H), 8.12 (s, 1H), 8.08 (s, 1H), 8.05 (d, *J* = 7.6 Hz, 1H), 7.83 (q, *J* = 8.4 Hz, 2H), 7.76 (t, *J* = 7.1 Hz, 1H), 7.73 (d, *J* = 7.5 Hz, 1H), 7.68 (t, *J* = 7.6 Hz, 1H), 7.49 (s, 1H). ^{13}C NMR (126 MHz, DMSO-*d6*) δ 168.05, 152.75, 143.09, 136.87, 135.25, 133.58, 133.12, 132.41, 131.74, 129.31, 129.25, 128.62, 128.47, 128.07, 127.70, 124.40. HRMS (ESI): *m/z* $[M+H]^+$ calcd for $C_{16}H_{12}N_2O$:249.1022; found:249.1022.

3-(1-aminoisoquinolin-4-yl)benzamide **(6)**

^{1}H NMR (500 MHz, DMSO-*d*$_6$) δ 8.33 (d, *J* = 6.0 Hz, 1H), 8.10 (s, 1H), 7.96 (s, 1H), 7.92 (d, *J* = 6.5 Hz, 1H), 7.81 (s, 1H), 7.71 – 7.62 (m, 2H), 7.59 (dd, *J* = 9.5, 4.2 Hz, 2H), 7.54 (ddd, *J* = 8.1, 5.8, 2.5 Hz, 1H), 7.41 (s, 1H), 6.97 (s, 2H). ^{13}C NMR (126 MHz, DMSO-*d*$_6$) δ 168.38, 157.69, 142.31, 138.40, 135.23, 135.07, 133.09, 130.78, 129.25, 128.97, 126.49, 125.98, 124.94, 124.23, 121.97, 117.10. HRMS (ESI): *m/z* $[M+H]^+$ calcd for $C_{16}H_{13}N_3O$:264.1131; found:264.1131.

3-(1H-indol-5-yl)benzamide **(7)**

^{1}H NMR (400 MHz, DMSO-*d*$_6$) δ 11.21 (s, 1H), 8.21 (s, 1H), 8.15 (s, 1H), 7.91 (s, 1H), 7.87 – 7.75 (m, 2H), 7.53 (d, *J* = 7.6 Hz, 1H), 7.50 (s, 1H), 7.48 (d, *J* = 1.4 Hz,

1H), 7.46 (s, 1H), 7.41 (t, *J* = 2.7 Hz, 1H), 6.52 (s, 1H). ^{13}C NMR (101 MHz, DMSO-d_6) δ 184.70, 168.55, 142.30, 136.08, 135.25, 131.16, 129.82, 129.21, 128.73, 126.67, 126.11, 125.83, 120.88, 118.83, 112.32, 102.05. HRMS (ESI): *m/z* $[M+H]^+$ calcd for $C_{15}H_{11}N_2O$:237.1022; found:237.1019.

3-(1-methyl-1H-indol-5-yl)benzamide **(8)**

^{1}H NMR (500 MHz, DMSO-d_6) δ 8.21 (d, *J* = 1.5 Hz, 1H), 8.11 (s, 1H), 7.91 (s, 1H), 7.82 (td, *J* = 7.8, 1.5 Hz, 2H), 7.52 (dd, *J* = 13.8, 4.3 Hz, 3H), 7.39 (s, 1H), 7.37 (d, *J* = 3.0 Hz, 1H), 6.50 (d, *J* = 3.0 Hz, 1H), 3.82 (s, 3H). ^{13}C NMR (126 MHz, DMSO-d_6) δ 168.53, 142.11, 136.63, 135.31, 131.25, 130.94, 129.83, 129.22, 129.09, 126.11, 125.91, 120.90, 119.09, 110.62, 101.33, 33.04. HRMS (ESI): *m/z* $[M+H]^+$ calcd for $C_{16}H_{14}N_2O$:251.1179; found:251.1182.

3-(benzofuran-5-yl)benzamide **(9)**

^{1}H NMR (500 MHz, DMSO-d_6) δ 8.22 (s, 1H), 8.13 (s, 1H), 8.06 (d, J = 2.1 Hz, 1H), 8.01 (d, *J* = 1.4 Hz, 1H), 7.86 (dd, *J* = 14.1, 7.8 Hz, 2H), 7.69 (dt, *J* = 8.6, 5.2 Hz, 2H), 7.56 (t, *J* = 7.7 Hz, 1H), 7.44 (s, 1H), 7.06 – 7.00 (m, 1H). ^{13}C NMR (126 MHz, DMSO-d_6) δ 168.34, 154.57, 147.25, 141.02, 135.41, 135.39, 130.13, 129.37, 128.42, 126.66, 126.44, 124.13, 120.07, 112.10, 107.48. HRMS (ESI): *m/z* $[M+H]^+$ calcd for $C_{15}H_{11}NO_2$:238.0863; found:238.0861.

3-(benzo[b]thiophen-5-yl)benzamide **(10)**

^{1}H NMR (500 MHz, DMSO-d_6) δ 8.27 (d, *J* = 4.7 Hz, 2H), 8.19 (s, 1H), 8.12 (d, *J* = 8.4 Hz, 1H), 7.91 (d, *J* = 1.7 Hz, 1H), 7.89 (d, *J* = 1.7 Hz, 1H), 7.83 (d, *J* = 5.4 Hz, 1H), 7.76 (dd, *J* = 8.4, 1.7 Hz, 1H), 7.58 (t, *J* = 7.7 Hz, 1H), 7.54 (d, *J* = 5.4 Hz, 1H), 7.45 (s, 1H). ^{13}C NMR (126 MHz, DMSO-d_6) δ 168.31, 140.70, 140.69, 139.05, 136.49, 135.47, 130.07, 129.44, 128.79, 126.94, 126.40, 124.73, 123.87, 123.57, 122.23. HRMS (ESI): *m/z* $[M+H]^+$ calcd for $C_{15}H_{11}NOS$:254.0634; found: 254.0639.

3-(3-chloro-1H-pyrrolo[2,3-b]pyridin-5-yl)benzamide **(11)**

^{1}H NMR (500 MHz, DMSO-d_6) δ 12.24 (s, 1H), 8.73 (d, *J* = 2.1 Hz, 1H), 8.55 (d, *J* = 2.1 Hz, 1H), 8.23 (s, 1H), 8.15 (s, 1H), 7.93 (t, *J* = 7.3 Hz, 2H), 7.59 (t, *J* = 7.7 Hz, 1H), 7.49 (s, 1H).^{13}C NMR (126 MHz, DMSO-d_6) δ 169.37, 168.04, 153.38, 149.72, 136.46, 135.62, 132.02, 131.90, 129.74, 129.65, 127.80, 125.89, 124.15, 74.50.

HRMS (ESI): *m/z* $[M+H]^+$ calcd for $C_{14}H_{10}N_3OCl$:272.0585; found:272.0580.

General procedure for synthesis of compounds **12-40**

Compound **a2** (3 g, 15.23 mmol) was dissolved in THF (60 mL), then NaOH (1.22 g, 30.46 mmol) and H_2O (6 mL) were added into the solution. Then, the mixture was stirred at 30°C for 10 min. *p*-toluenesulfonyl chloride under was added under ice bath. Then, the mixture was stirred at 30 °C for 6 h. The reaction was concentrated to obtain yellow oil, then EA was added and the mixture was washed with saturated brine solution, dried over anhydrous Na_2SO_4, then purified by column chromatography (gradient elution of PE/EA 85/15, v/v) to obtain compound **M1.**

Compound **M2** was obtained according to the similar procedures with compound **1**. Compound **M2** (2 g, 5.71 mmol) was dissolved in DMF (60 mL), then NIS (1.67 g, 7.41 mmol) was added into the solution under ice bath. Then, the mixture was stirred at 65 °C for 12 h. After the reaction was completed, the reaction was quenched with aqueous sodium thiosulfate solution, then extracted with EA (200 mL×2). The combined organic layer was washed with saturated brine solution, dried over anhydrous Na_2SO_4, then purified by column chromatography (gradient elution of PE/ EA 30/70, v/v) to obtain compound **M3.**

Compounds **M4-M32** were obtained according to the similar procedures with compound **1**. Compound **M4** (200 mg), NaOH (60 mg) and H_2O (2 mL) and CH_3CH_2OH (20 mL) were added into flask. Then, the mixture was stirred at 75 °C for 2 h. The reaction was concentrated, then EA was added and the mixture was washed with saturated brine solution, dried over anhydrous Na_2SO_4, then purified by column chromatography (gradient elution of PE/EA 10/90, v/v) to obtain compound **12.**

3-(3-(furan-3-yl)-1H-pyrrolo[2,3-b]pyridin-5-yl)benzamide **(12)**

^{1}H NMR (400 MHz, DMSO-*d*$_6$) δ 11.91 (s, 1H), 8.64 (d, *J* = 2.0 Hz, 1H), 8.47 (d, *J* = 1.9 Hz, 1H), 8.38 (s, 1H), 8.26 (s, 1H), 8.16 (s, 1H), 7.98 (d, *J* = 7.8 Hz, 1H), 7.90 – 7.85 (m, 2H), 7.75 (t, *J* = 1.6 Hz, 1H), 7.59 (t, *J* = 7.7 Hz, 1H), 7.49 (s, 1H), 7.02 (d, *J* = 1.0 Hz, 1H). ^{13}C NMR (101 MHz, DMSO-*d*$_6$) δ 168.35, 148.97, 143.80, 142.53, 139.33, 138.25, 135.42, 130.44, 129.42, 128.45, 126.69, 126.37 (2C), 124.80, 119.74, 117.62, 109.81, 107.05. HRMS (ESI): *m/z* $[M+H]^+$ calcd for $C_{18}H_{13}N_3O_2$:239.0927;

Compounds **13-40** were obtained according to the similar procedures with compound **12.**

found:304.1084. *3-(3-(thiophen-3-yl)-1H-pyrrolo[2,3-b]pyridin-5-yl)benzamide* **(13)**

^{1}H NMR (400 MHz, DMSO-*d6*) δ 11.98 (s, 1H), 8.62 (d, *J* = 22.3 Hz, 2H), 8.22 (d, *J* = 36.9 Hz, 2H), 7.97 (t, *J* = 10.3 Hz, 3H), 7.88 (d, *J* = 7.5 Hz, 1H), 7.65 (s, 2H), 7.59 (t, *J* = 7.6 Hz, 1H), 7.50 (s, 1H). ^{13}C NMR (101 MHz, DMSO-*d6*) δ 168.31, 148.98, 142.59, 139.41, 135.70, 135.42, 130.43, 129.44, 128.66, 127.26, 126.71, 126.61, 126.38, 126.27, 125.24, 118.71, 117.69, 111.16. HRMS (ESI): *m/z* $[M+H]^+$ calcd for $C_{18}H_{13}N_3OS$: 320.0852; found:320.0853.

3-(3-(thiophen-2-yl)-1H-pyrrolo[2,3-b]pyridin-5-yl)benzamide (**14**)

^{1}H NMR (500 MHz, DMSO-d_6) δ 12.10 (s, 1H), 8.68 (d, *J* = 1.7 Hz, 1H), 8.54 (d, *J* = 1.5 Hz, 1H), 8.27 (s, 1H), 8.17 (s, 1H), 7.96 (d, *J* = 7.7 Hz, 1H), 7.91 (d, *J* = 7.6 Hz, 2H), 7.71 (s, 1H), 7.60 (t, *J* = 7.7 Hz, 1H), 7.48 (s, 1H), 6.88 (d, *J* = 3.1 Hz, 1H), 6.61 (d, *J* = 1.7 Hz, 1H). ^{13}C NMR (126 MHz, DMSO-d_6) δ 168.32, 150.02, 148.69, 143.04, 141.25, 139.24, 135.50, 130.37, 129.49, 128.92, 126.80, 126.40, 126.36, 123.94, 116.52, 112.00, 106.89, 104.14. HRMS (ESI): *m/z* $[M+H]^+$ calcd for $C_{18}H_{13}N_3OS$:320.0852; found: 320.0855.

3-(3-(1H-pyrazol-4-yl)-1H-pyrrolo[2,3-b]pyridin-5-yl)benzamide (**15**)

^{1}H NMR (500 MHz, DMSO-d_6) δ 13.52 (s, 1H), 8.63 (d, *J* = 2.1 Hz, 1H), 8.45 (d, *J* = 8.4 Hz, 1H), 8.29 (d, *J* = 2.0 Hz, 1H), 8.12 (s, 1H), 8.09 (s, 1H), 8.06 – 7.95 (m, 1H), 7.82 (d, *J* = 7.7 Hz, 2H), 7.53 (t, *J* = 7.7 Hz, 1H), 7.41 (s, 3H).^{13}C NMR (126 MHz, DMSO-d_6) δ 189.51, 168.24, 158.82, 151.64, 141.24, 138.65, 137.65, 135.41, 133.27, 129.45, 129.03, 126.50, 125.08, 123.81, 122.29, 122.12, 114.10. HRMS (ESI): *m/z* $[M+H]^+$ calcd for $C_{17}H_{13}N_5O$:304.1193; found: 304.1169

3-(3-(1-methyl-1H-pyrazol-4-yl)-1H-pyrrolo[2,3-b]pyridin-5-yl)benzamide (**16**)

^{1}H NMR (400 MHz, DMSO-d_6) δ 11.83 (s, 1H), 8.62 (d, *J* = 1.9 Hz, 1H), 8.45 (d, *J* = 1.7 Hz, 1H), 8.27 (d, *J* = 3.4 Hz, 2H), 8.20 (s, 1H), 7.96 (d, *J* = 7.8 Hz, 1H), 7.92 (s, 1H), 7.89 (d, *J* = 7.7 Hz, 1H), 7.77 (d, *J* = 2.3 Hz, 1H), 7.59 (t, *J* = 7.7 Hz, 1H), 7.51 (s, 1H), 3.91 (s, 3H). ^{13}C NMR (101 MHz, DMSO-d_6) δ 168.35, 148.81, 142.40, 139.52, 136.65, 135.41, 130.41, 129.41, 128.29, 127.56, 126.63, 126.40, 125.99,

123.26, 117.85, 115.62, 107.36, 60.23. HRMS (ESI): *m/z* $[M+H]^+$ calcd for $C_{18}H_{15}N_5O$:318.1349; found:318.1346.

3-(3-(1-(1-ethoxyethyl)-1H-pyrazol-4-yl)-1H-pyrrolo[2,3-b]pyridin-5-yl)benzamide (**17**)

^{1}H NMR (500 MHz, DMSO-*d*$_6$) δ 11.82 (s, 1H), 8.62 (d, *J* = 1.6 Hz, 1H), 8.49 (s, 1H), 8.44 (s, 1H), 8.26 (s, 1H), 8.14 (s, 1H), 8.02 (s, 1H), 7.97 (d, *J* = 7.7 Hz, 1H), 7.89 (d, *J* = 7.7 Hz, 1H), 7.83 (d, *J* = 2.2 Hz, 1H), 7.59 (t, *J* = 7.7 Hz, 1H), 7.45 (s, 1H), 5.60 (t, *J* = 6.0 Hz, 1H), 3.47 (dq, *J* = 14.1, 7.0 Hz, 1H), 3.28 (dq, *J* = 14.2, 7.1 Hz, 1H), 1.68 (d, *J* = 6.0 Hz, 3H), 1.07 (t, *J* = 7.0 Hz, 3H). ^{13}C NMR (126 MHz, DMSO-*d*$_6$) δ 168.35, 148.85, 142.48, 139.55, 136.98, 135.43, 130.48, 129.39, 128.39, 126.62, 126.48, 126.10, 124.52, 123.66, 117.77, 116.24, 107.19, 86.75, 63.42, 21.70, 15.25. HRMS (ESI): *m/z* $[M+H]^+$ calcd for $C_{21}H_{21}N_5O_2$:376.1768; found: 376.1771.

3-(3-(1-(difluoromethyl)-1H-pyrazol-4-yl)-1H-pyrrolo[2,3-b]pyridin-5-yl)benzamide (**18**)

^{1}H NMR (500 MHz, DMSO-*d*$_6$) δ 11.96 (s, 1H), 8.81 (s, 1H), 8.65 (d, *J* = 1.5 Hz, 1H), 8.54 (s, 1H), 8.35 (s, 1H), 8.27 (s, 1H), 8.13 (s,1H), 8.00-7.97 (m, 2.25H), 7.89 (d, *J* = 7.7 Hz, 14H), 7.84 (d, *J* = 10.4 Hz, 0.5H), 7.73 (s, 0.25H), 7.60 (t, *J* = 7.7 Hz, 1H), 7.46 (s,1H). ^{13}C NMR (126 MHz, DMSO-*d*$_6$) δ 168.35, 148.86, 142.74, 140.90, 139.41, 135.43, 130.53, 129.37, 128.70, 126.68, 126.52, 126.12, 124.85, 124.43, 118.51, 117.61, 113.13, 111.16, 109.20, 105.63. HRMS (ESI): *m/z* $[M+H]^+$ calcd for $C_{18}H_{13}N_5OF_2$:354.1161; found: 354.1136.

3-(3-phenyl-1H-pyrrolo[2,3-b]pyridin-5-yl)benzamide **(19)**

^{1}H NMR (400 MHz, DMSO-*d6*) δ 12.08 (s, 1H), 8.65 (d, *J* = 1.5 Hz, 1H), 8.53 (s, 1H), 8.26 (s, 1H), 8.18 (s, 1H), 7.95 (d, *J* = 6.4 Hz, 2H), 7.89 (d, *J* = 7.7 Hz, 1H), 7.81 (d, *J* = 7.5 Hz, 2H), 7.58 (t, *J* = 7.7 Hz, 1H), 7.47 (t, *J* = 7.5 Hz, 3H), 7.28 (t, *J* = 7.3 Hz, 1H).^{13}C NMR (101 MHz, DMSO-*d6*) δ 168.29, 149.24, 142.61, 139.49, 135.42, 135.37, 130.39, 129.48, 129.42 (2C), 128.75, 126.95 (2C), 126.72, 126.38, 126.25, 126.09, 125.24, 117.78, 115.26. HRMS (ESI): *m/z* $[M+H]^+$ calcd for $C_{20}H_{15}N_3O$:314.1288; found:314.1286.

3-(3-(2-fluorophenyl)-1H-pyrrolo[2,3-b]pyridin-5-yl)benzamide (**20**)

^{1}H NMR (500 MHz, DMSO-d_6) δ 12.19 (s, 1H), 8.68 (d, J = 1.8 Hz, 1H), 8.35 (s, 1H), 8.23 (s, 1H), 8.14 (s, 1H), 7.87 (ddd, J = 24.7, 14.3, 7.5 Hz, 4H), 7.58 (t, J = 7.7 Hz, 1H), 7.41 (d, J = 39.8 Hz, 2H), 7.34 (dd, J = 16.0, 8.7 Hz, 2H). ^{13}C NMR (126 MHz, DMSO-d_6) δ 168.27, 160.49, 158.54, 148.77, 142.76, 139.41, 135.47, 130.59, 130.55, 130.32, 129.50, 128.78, 128.50, 128.33, 128.27, 127.21, 127.17, 126.73, 126.45, 126.42, 126.36, 125.99, 125.39, 125.37, 122.70, 122.58, 118.30, 116.58, 116.40, 108.94. HRMS (ESI): *m/z* $[M+H]^+$ calcd for $C_{20}H_{14}N_3OF$:332.1194; found: 332.1196.

3-(3-(2-chlorophenyl)-1H-pyrrolo[2,3-b]pyridin-5-yl)benzamide （**21**）

^{1}H NMR (500 MHz, DMSO-d_6) δ 12.17 (s, 3H), 8.66 (d, J = 1.8 Hz, 3H), 8.52 (d, J = 1.8 Hz, 3H), 8.26 (s, 3H), 8.15 (s, 4H), 8.07 (s, 2H), 7.95 (d, J = 7.7 Hz, 3H), 7.89 (d, J = 7.7 Hz, 3H), 7.86 – 7.79 (m, 6H), 7.59 (t, J = 7.7 Hz, 3H), 7.49 (dd, J = 16.2, 8.1 Hz, 6H), 7.32 (d, J = 7.8 Hz, 3H). ^{13}C NMR (126 MHz, DMSO-d_6) δ 168.25, 149.21, 142.84, 139.41, 137.64, 135.42, 134.16, 131.23, 130.47, 129.48, 129.02, 126.76, 126.46, 126.27(2C), 126.08, 125.98, 125.48, 117.55, 113.79. HRMS (ESI): *m/z* $[M+H]^+$ calcd for $C_{20}H_{14}N_3OCl$:348.0898; found: 348.0898.

3-(3-(2-hydroxyphenyl)-1H-pyrrolo[2,3-b]pyridin-5-yl)benzamide (**22**)

^{1}H NMR (500 MHz, DMSO-d_6) δ 10.13 (s, 1H), 8.67 (d, J = 2.4 Hz, 1H), 8.08 (s, 1H), 8.02 (s, 1H), 7.84 (dd, J = 16.4, 5.0 Hz, 4H), 7.60 (d, J = 7.7 Hz, 1H), 7.52 (t, J = 7.7 Hz, 1H), 7.48 – 7.38 (m, 2H), 7.38 – 7.32 (m, 1H), 6.99 (dd, J = 12.8, 7.8 Hz, 2H). ^{13}C NMR (126 MHz, DMSO-d_6) δ 198.13, 168.16, 158.88, 155.36, 152.85, 141.07, 137.61, 135.53, 132.53, 129.85, 129.54, 128.67, 127.10, 126.42, 124.98, 123.69, 119.69, 116.80, 112.68. HRMS (ESI): *m/z* $[M+H]^+$ calcd for $C_{20}H_{15}N_3O_2$:330.1237; found: 330.3334.

3-(3-(o-tolyl)-1H-pyrrolo[2,3-b]pyridin-5-yl)benzamide (**23**)

^{1}H NMR (500 MHz, DMSO-d_6) δ 11.99 (s, 1H), 8.65 (d, J = 1.9 Hz, 1H), 8.18 (s, 1H), 8.12 (s, 1H), 8.08 (d, J = 1.6 Hz, 1H), 7.92 – 7.82 (m, 2H), 7.65 (d, J = 2.1 Hz, 1H), 7.54 (t, J = 7.7 Hz, 1H), 7.48 – 7.43 (m, 1H), 7.41 (s, 1H), 7.36 (d, J = 7.0 Hz, 1H), 7.32 – 7.21 (m, 2H), 2.35 (s, 3H). ^{13}C NMR (126 MHz, DMSO-d_6) δ 168.28, 148.63, 142.45, 139.44, 136.34, 135.46, 134.17, 131.01, 130.75, 130.20, 129.46, 128.44, 127.08, 126.64, 126.40, 126.32, 126.23, 125.77, 119.21, 114.71, 21.05. HRMS (ESI):

m/z [M+H]$^+$ calcd for $C_{21}H_{17}N_3O$:328.1444; found: 328.1449.

3-(3-(2-ethylphenyl)-1H-pyrrolo[2,3-b]pyridin-5-yl)benzamide (**24**)

^{1}H NMR (500 MHz, DMSO-*d6*) δ 11.97 (s, 1H), 8.65 (s, 1H), 8.17 (s, 1H), 8.12 (s, 1H), 8.01 (s, 1H), 7.85 (d, *J* = 7.7 Hz, 2H), 7.57 (d, *J* = 21.5 Hz, 1H), 7.53 (d, *J* = 7.7 Hz, 1H), 7.48 – 7.35 (m, 3H), 7.31 (dt, *J* = 19.4, 7.1 Hz, 2H), 2.68 (q, *J* = 7.4 Hz, 2H), 1.07 (t, *J* = 7.5 Hz, 3H). ^{13}C NMR (126 MHz, DMSO-*d6*) δ 168.28, 148.58, 142.81, 142.46, 139.39, 135.47, 133.51, 131.33, 130.15, 129.46, 129.10, 128.40, 127.56, 126.63, 126.29, 126.20, 125.84, 125.48, 119.57, 114.67, 26.39, 15.98. HRMS (ESI): *m/z* [M+H]$^+$ calcd for $C_{22}H_{19}N_3O$:342.1601; found: 342.1606.

3-(3-(2-methoxyphenyl)-1H-pyrrolo[2,3-b]pyridin-5-yl)benzamide (**25**)

^{1}H NMR (500 MHz, DMSO-*d6*) δ 11.95 (s, 1H), 8.62 (d, *J* = 1.5 Hz, 1H), 8.26 (s, 1H), 8.21 (s, 1H), 8.13 (s, 1H), 7.88 (t, *J* = 8.5 Hz, 2H), 7.76 (d, *J* = 2.2 Hz, 1H), 7.62 (d, *J* = 7.4 Hz, 1H), 7.57 (t, *J* = 7.7 Hz, 1H), 7.42 (s, 1H), 7.31 (t, *J* = 7.8 Hz, 1H), 7.15 (d, *J* = 8.2 Hz, 1H), 7.07 (t, *J* = 7.4 Hz, 1H), 3.84 (s, 3H). ^{13}C NMR (126 MHz, DMSO-*d6*) δ 168.31, 156.70, 148.70, 142.21, 139.65, 135.48, 130.19, 130.18, 129.49, 128.29, 127.89, 126.84, 126.59, 126.31, 123.77, 121.24, 118.93, 112.13, 111.71, 55.78. HRMS (ESI): *m/z* [M+H]$^+$ calcd for $C_{21}H_{17}N_3O_2$:344.1394; found: 344.1394.

3-(3-(2-isopropylphenyl)-1H-pyrrolo[2,3-b]pyridin-5-yl)benzamide (**26**)

^{1}H NMR (500 MHz, DMSO-*d6*) δ 11.96 (s, 1H), 8.66 (d, *J* = 1.6 Hz, 1H), 8.16 (s, 1H), 8.11 (s, 1H), 7.98 (s, 1H), 7.89 – 7.79 (m, 2H), 7.59 – 7.50 (m, 2H), 7.48 (d, *J* = 7.8 Hz, 1H), 7.42 – 7.32 (m, 3H), 7.27 (t, *J* = 7.4 Hz, 1H), 3.18 (dq, *J* = 13.4, 6.6 Hz, 1H), 1.15 (d, *J* = 6.8 Hz, 6H). ^{13}C NMR (126 MHz, DMSO-*d6*) δ 168.30, 148.53, 147.84, 142.47, 139.35, 135.52, 132.79, 131.50, 130.06, 129.48, 128.38, 127.93, 126.61, 126.18, 126.09, 126.04, 125.82, 125.23, 119.94, 114.72, 29.65, 24.63(2C). HRMS (ESI): *m/z* [M+H]$^+$ calcd for $C_{23}H_{21}N_3O$:356.1757; found: 356.1761.

3-(3-(2-(trifluoromethyl)phenyl)-1H-pyrrolo[2,3-b]pyridin-5-yl)benzamide (**27**)

^{1}H NMR (500 MHz, DMSO-*d6*) δ 12.10 (s, 1H), 8.68 (s, 1H), 8.16 (s, 1H), 8.11 (s, 1H), 8.04 (s, 1H), 7.89 (d, *J* = 7.9 Hz, 1H), 7.86 (d, *J* = 7.7 Hz, 2H), 7.76 (d, *J* = 7.5 Hz, 1H), 7.67 (d, *J* = 7.6 Hz, 1H), 7.64 – 7.57 (m, 2H), 7.54 (t, *J* = 7.7 Hz, 1H), 7.41 (s, 1H). ^{13}C NMR (126 MHz, DMSO-*d6*) δ 168.26, 148.29, 142.77, 139.25, 135.47,

133.85, 133.56, 132.89, 130.19, 129.45, 128.71, 128.48, 128.25, 127.96, 126.90, 126.86, 126.69, 126.43, 126.23, 125.92, 125.33, 123.75, 119.81, 112.22. HRMS (ESI): *m/z* $[M+H]^+$ calcd for $C_{21}H_{14}N_3OF_3$:382.1162; found: 382.1168.

3-(3-(2-(trifluoromethoxy)phenyl)-1H-pyrrolo[2,3-b]pyridin-5-yl)benzamide (**28**)

^{1}H NMR (500 MHz, DMSO-d_6) δ 12.19 (s, 1H), 8.68 (s, 1H), 8.29 (s, 1H), 8.22 (s, 1H), 8.13 (s, 1H), 7.89 (t, *J* = 7.5 Hz, 2H), 7.86 – 7.80 (m, 2H), 7.58 (t, *J* = 7.7 Hz, 1H), 7.55 – 7.50 (m, 2H), 7.46 (dd, *J* = 15.9, 7.1 Hz, 2H). ^{13}C NMR (126 MHz, DMSO-d_6) δ 168.26, 148.71, 145.88, 142.79, 139.37, 135.50, 131.75, 130.23, 129.50, 128.79, 128.57, 128.47, 128.37, 127.18, 126.72, 126.32, 126.30, 122.15, 121.60, 119.56, 118.35, 109.87. HRMS (ESI): *m/z* $[M+H]^+$ calcd for $C_{21}H_{14}N_3O_2F_3$:398.1111; found: 398.1113.

3-(3-(3-fluorophenyl)-1H-pyrrolo[2,3-b]pyridin-5-yl)benzamide (**29**)

^{1}H NMR (500 MHz, DMSO-d_6) δ 12.15 (s, 1H), 8.66 (d, *J* = 1.7 Hz, 1H), 8.55 (d, *J* = 1.7 Hz, 1H), 8.26 (s, 1H), 8.15 (s, 1H), 8.05 (s, 1H), 7.97 (d, *J* = 7.7 Hz, 1H), 7.90 (d, *J* = 7.7 Hz, 1H), 7.70 (d, *J* = 7.8 Hz, 1H), 7.68 – 7.56 (m, 2H), 7.56 – 7.38 (m, 2H), 7.14 – 7.04 (m, 1H). ^{13}C NMR (126 MHz, DMSO-d_6) δ 168.28, 164.24, 162.31, 149.23, 142.80, 139.41, 137.93, 137.86, 135.44, 131.31, 131.24, 130.46, 129.46, 129.00, 126.76, 126.45, 126.18, 126.14, 122.88, 117.57, 114.10, 114.08, 113.33, 113.15, 112.90, 112.73. HRMS (ESI): *m/z* $[M+H]^+$ calcd for $C_{20}H_{14}N_3OF$:332.1194; found: 332.1195.

3-(3-(3-chlorophenyl)-1H-pyrrolo[2,3-b]pyridin-5-yl)benzamide （**30**）

^{1}H NMR (500 MHz, DMSO-d_6) δ 12.17 (s, 1H), 8.66 (d, *J* = 1.8 Hz, 1H), 8.52 (d, *J* = 1.8 Hz, 1H), 8.26 (s, 1H), 8.16 (s, 1H), 8.07 (s, 1H), 7.96 (d, *J* = 7.7 Hz, 1H), 7.90 (d, *J* = 7.7 Hz, 1H), 7.83 (d, *J* = 6.8 Hz, 2H), 7.59 (t, *J* = 7.7 Hz, 1H), 7.49 (dd, *J* = 17.5, 9.4 Hz, 2H), 7.32 (d, *J* = 7.8 Hz, 1H). ^{13}C NMR (126 MHz, DMSO-d_6) δ 168.27, 149.22, 142.84, 139.42, 137.64, 135.44, 134.17, 131.23, 130.46, 129.48, 129.03, 126.76, 126.47, 126.28, 126.25, 126.07, 125.98, 125.48, 117.56, 113.80. HRMS (ESI): *m/z* $[M+H]^+$ calcd for $C_{20}H_{14}N_3OCl$:348.0898; found: 348.0896.

3-(3-(3-hydroxyphenyl)-1H-pyrrolo[2,3-b]pyridin-5-yl)benzamide (**31**)

^{1}H NMR (500 MHz, DMSO-d_6) δ 12.01 (s, 1H), 9.41 (s, 1H), 8.64 (d, *J* = 1.9 Hz, 1H),

8.47 (d, *J* = 1.7 Hz, 1H), 8.24 (s, 1H), 8.16 (s, 1H), 7.96 – 7.84 (m, 3H), 7.59 (t, *J* = 7.7 Hz, 1H), 7.45 (s, 1H), 7.32 – 7.17 (m, 3H), 6.69 (dd, *J* = 7.8, 1.1 Hz, 1H). ^{13}C NMR (126 MHz, DMSO-d_6) *δ* 170.80, 168.26, 158.26, 149.23, 142.56, 139.57, 136.54, 135.47, 130.40, 130.34, 129.50, 128.72, 126.70, 126.39, 126.06, 125.07, 117.81, 115.38, 113.71, 113.38. HRMS (ESI): *m/z* $[M+H]^+$ calcd for $C_{20}H_{15}N_3O_2$:330.1237; found: 330.1238.

3-(3-(3-methoxyphenyl)-1H-pyrrolo[2,3-b]pyridin-5-yl)benzamide (**32**)

^{1}H NMR (500 MHz, DMSO-d_6) *δ* 12.05 (s, 1H), 8.65 (d, *J* = 1.5 Hz, 1H), 8.50 (s, 1H), 8.25 (s, 1H), 8.15 (s, 1H), 8.00 – 7.83 (m, 3H), 7.58 (t, *J* = 7.7 Hz, 1H), 7.44 (s, 1H), 7.39 (d, *J* = 5.9 Hz, 2H), 7.31 (s, 1H), 6.93 – 6.81 (m, 1H), 3.84 (s, 3H). ^{13}C NMR (126 MHz, DMSO-d_6) *δ* 168.28, 160.26, 149.24, 142.62, 139.50, 136.74, 135.46, 130.46, 130.34, 129.49, 128.76, 126.71, 126.38, 126.06, 125.50, 119.44, 117.83, 115.22, 112.45, 111.89, 55.53. HRMS (ESI): *m/z* $[M+H]^+$ calcd for $C_{21}H_{17}N_3O_2$:344.1394; found: 344.1392.

3-(3-(3-(trifluoromethyl)phenyl)-1H-pyrrolo[2,3-b]pyridin-5-yl)benzamide (**33**)

^{1}H NMR (500 MHz, DMSO-d_6) *δ* 12.22 (s, 1H), 8.67 (d, *J* = 1.7 Hz, 1H), 8.53 (d, *J* = 1.8 Hz, 1H), 8.26 (s, 1H), 8.23 – 8.04 (m, 4H), 7.93 (dd, *J* = 25.6, 7.7 Hz, 2H), 7.72 (t, *J* = 7.8 Hz, 1H), 7.60 (dd, *J* = 14.8, 7.3 Hz, 2H), 7.45 (s, 1H). ^{13}C NMR (126 MHz, DMSO-d_6) *δ* 168.25, 149.25, 142.92, 139.41, 136.54, 135.46, 130.78, 130.50, 130.42, 130.16, 129.49, 129.10, 126.77, 126.48, 125.98, 125.92, 123.76, 123.07, 123.04, 122.60, 122.57, 117.57, 113.75. HRMS (ESI): *m/z* $[M+H]^+$ calcd for $C_{21}H_{14}N_3OF_3$:382.1162; found: 382.1157.

3-(3-(3-(trifluoromethoxy)phenyl)-1H-pyrrolo[2,3-b]pyridin-5-yl)benzamide (**34**)

^{1}H NMR (500 MHz, DMSO-d_6) *δ* 12.20 (s, 1H), 8.67 (d, *J* = 1.8 Hz, 1H), 8.53 (d, *J* = 1.8 Hz, 1H), 8.26 (s, 1H), 8.12 (d, *J* = 20.6 Hz, 2H), 7.95 (d, *J* = 7.7 Hz, 1H), 7.90 (d, *J* = 7.8 Hz, 2H), 7.75 (s, 1H), 7.60 (td, *J* = 7.8, 5.3 Hz, 2H), 7.45 (s, 1H), 7.26 (d, *J* = 8.1 Hz, 1H). ^{13}C NMR (126 MHz, DMSO-d_6) *δ* 168.25, 149.54, 149.24, 142.89, 139.40, 137.83, 135.45, 131.31, 130.42, 129.48, 129.07, 126.77, 126.47, 126.39, 126.03, 125.88, 121.70, 119.67, 119.12, 118.33, 117.53, 113.70. HRMS (ESI): *m/z* $[M+H]^+$ calcd for $C_{21}H_{14}N_3O_2F_3$:330.1237; found: 330.1238.

3-(3-(4-fluorophenyl)-1H-pyrrolo[2,3-b]pyridin-5-yl)benzamide **(35)**

^{1}H NMR (400 MHz, DMSO-*d*$_6$) δ 12.11 (s, 1H), 8.66 (d, *J* = 1.7 Hz, 1H), 8.50 (d, *J* = 1.5 Hz, 1H), 8.23 (d, *J* = 24.2 Hz, 2H), 7.88 (ddd, *J* = 15.1, 14.0, 6.7 Hz, 5H), 7.57 (dd, *J* = 22.0, 14.3 Hz, 2H), 7.30 (t, *J* = 8.8 Hz, 2H). ^{13}C NMR (101 MHz, DMSO-*d*$_6$) δ 168.29, 162.32, 159.91, 149.14, 142.66, 139.46, 135.40, 131.83, 130.41, 129.47, 128.77, 128.70, 126.73, 126.40, 125.98, 125.23, 117.68, 116.28, 116.07, 114.27. HRMS (ESI): *m/z* [M+H]$^+$ calcd for $C_{20}H_{14}N_3OF$:332.1194; found:332.1198.

3-(3-(4-chlorophenyl)-1H-pyrrolo[2,3-b]pyridin-5-yl)benzamide **(36)**

^{1}H NMR (400 MHz, DMSO-*d*$_6$) δ 12.17 (s, 1H), 8.66 (d, *J* = 1.4 Hz, 1H), 8.53 (s, 1H), 8.26 (s, 1H), 8.18 (s, 1H), 8.01 (d, *J* = 1.8 Hz, 1H), 7.95 (d, *J* = 7.6 Hz, 1H), 7.89 (d, *J* = 7.7 Hz, 1H), 7.85 (d, *J* = 8.4 Hz, 2H), 7.59 (t, *J* = 7.7 Hz, 1H), 7.51 (d, *J* = 8.3 Hz, 3H). ^{13}C NMR (101 MHz, DMSO-*d*$_6$) δ 168.27, 149.21, 142.76, 139.41, 135.41, 134.30, 130.59, 130.44, 129.47, 129.32 (2C), 128.90, 128.52 (2C), 126.76, 126.42, 126.09, 125.74, 117.58, 113.97. HRMS (ESI): *m/z* [M+H]$^+$ calcd for $C_{20}H_{14}N_3OCl$:348.0898; found:348.0893.

3-(3-(4-hydroxyphenyl)-1H-pyrrolo[2,3-b]pyridin-5-yl)benzamide (**37**)

^{1}H NMR (500 MHz, DMSO-*d*$_6$) δ 10.35 (s, 1H), 8.63 (d, *J* = 2.2 Hz, 1H), 8.06 (d, *J* = 5.3 Hz, 2H), 7.97 (d, *J* = 2.2 Hz, 1H), 7.80 (d, *J* = 7.7 Hz, 1H), 7.72 (d, *J* = 7.6 Hz, 1H), 7.63 (d, *J* = 8.6 Hz, 2H), 7.50 (t, *J* = 7.7 Hz, 1H), 7.41 (s, 1H), 7.29 (s, 2H), 6.91 (d, *J* = 8.6 Hz, 2H). ^{13}C NMR (126 MHz, DMSO-*d*$_6$) δ 195.67, 168.18, 162.02, 159.02, 151.40, 139.36, 137.59, 135.45, 132.53, 129.70, 129.50, 128.84, 126.45, 124.99, 123.29, 115.74, 113.26. HRMS (ESI): *m/z* [M+H]$^+$ calcd for $C_{20}H_{15}N_3O_2$:330.1237; found: 330.1240.

3-(3-(4-methoxyphenyl)-1H-pyrrolo[2,3-b]pyridin-5-yl)benzamide (**38**)

^{1}H NMR (500 MHz, DMSO-*d*$_6$) δ 11.93 (s, 1H), 8.64 (s, 1H), 8.47 (s, 1H), 8.25 (s, 1H), 8.15 (s, 1H), 7.91 (dd, *J* = 23.5, 7.7 Hz, 2H), 7.81 (d, *J* = 1.8 Hz, 1H), 7.72 (d, *J* = 8.6 Hz, 2H), 7.58 (t, *J* = 7.7 Hz, 1H), 7.45 (s, 1H), 7.05 (d, *J* = 8.5 Hz, 2H), 3.81 (s, 3H). ^{13}C NMR (126 MHz, DMSO-*d*$_6$) δ 168.30, 158.13, 149.16, 142.46, 139.56, 135.45, 130.32, 129.46, 128.53, 128.18(2C), 127.84, 126.66, 126.34, 125.96, 124.27, 117.86, 115.12, 114.92(2C), 55.59. HRMS (ESI): *m/z* [M+H]$^+$ calcd for

$C_{21}H_{17}N_3O_2$:344.1394; found: 344.1395.

3-(3-(4-(trifluoromethyl)phenyl)-1H-pyrrolo[2,3-b]pyridin-5-yl)benzamide (**39**)

^{1}H NMR (500 MHz, DMSO-*d*$_6$) δ 12.26 (s, 1H), 8.69 (d, *J* = 1.6 Hz, 1H), 8.60 (s, 1H), 8.27 (s, 1H), 8.14 (s, 2H), 8.06 (d, *J* = 8.1 Hz, 2H), 7.97 (d, *J* = 7.7 Hz, 1H), 7.90 (d, *J* = 7.7 Hz, 1H), 7.79 (d, *J* = 8.2 Hz, 2H), 7.60 (t, *J* = 7.7 Hz, 1H), 7.46 (s, 1H). ^{13}C NMR (126 MHz, DMSO-*d*$_6$) δ 168.27, 149.33, 142.96, 139.67, 139.33, 135.47, 130.46, 129.46, 129.19, 127.17, 126.81, 126.46, 126.23, 126.19, 126.12, 123.96, 117.59, 113.77. HRMS (ESI): *m/z* $[M+H]^+$ calcd for $C_{21}H_{14}N_3OF_3$:382.1162; found: 382.1163.

3-(3-(4-(trifluoromethoxy)phenyl)-1H-pyrrolo[2,3-b]pyridin-5-yl)benzamide (**40**)

^{1}H NMR (500 MHz, DMSO-*d*$_6$) δ 12.14 (s, 1H), 8.67 (d, *J* = 1.8 Hz, 1H), 8.54 (d, *J* = 1.8 Hz, 1H), 8.26 (s, 1H), 8.14 (s, 1H), 8.01 (s, 1H), 7.99 – 7.86 (m, 4H), 7.59 (t, *J* = 7.7 Hz, 1H), 7.45 (d, *J* = 8.5 Hz, 3H). ^{13}C NMR (126 MHz, DMSO-*d*$_6$) δ 168.27, 149.22, 146.81, 142.79, 139.40, 135.45, 134.88, 130.43, 129.45, 128.97, 128.48(2C), 126.76, 126.42, 126.04, 125.90, 122.07(2C), 121.71, 119.67, 117.61, 113.86. HRMS (ESI): *m/z* $[M+H]^+$ calcd for $C_{21}H_{14}N_3O_2F_3$398.1111; found: 398.1115.

1. 2 Cell Culture

All the cell lines were purchased from the Shanghai Cell Resource Bank. SW-480, HT-29, MV4-11, Molm-13, MGC-803, MDA-MB-231, A375, A549, and GES-1 cells were cultured in RPMI-1640 with 10% FBS (*Shanghai Shuangru Biology Science & Technology Co.,Ltd*, LONSERA) and 1% penicillin-streptomycin (Beyotime). HCT-116 cells were cultured in McCoy's 5A contained 10%FBS and 1% P/S. Human Umbilical Vein Endothelial cells (HUVEC) were cultured in DMEM contained 10% FBS and 1% P/S. Cells were cultured within a humidified incubator at 37 °C in the presence of 5% CO_2.

1.3 MTT Assay

The cell viability of the compound was assessed by CCK-8 (Biofroxx) assay. Cells were seeded in a 96-well plate at 8000 cells/well and cultured in a 37 °C and 5%

CO_2 atmosphere incubator for 24 h. Then, medium with compound at the concentrations of 100, 20, 4, 0.8, 0.016 μM was added into each well for another 48 h and incubated with CCK-8 (10 μL) for 2 h at 37 °C. The absorbance value at 450 nm was measured by the microplate reader (PerkinElmer Envision).

1.4 Kinase Assay

The kinase inhibition was detected by ADP-Glo™ Kinase Assay (Promega) using a black 384-well plate. The active CDK8 kinase was diluted in mixture (5 ng enzyme, 0.5 *μ*g substrate, 50 *μ*M DTT, 1 *μ*L reaction buffer and added ddH2O to 3 *μ*L each well) and the final ATP concentrations of was 50 *μ*M. After incubation at room temperature, ADP-Glo™ solution and kinase detection reagent was added. The data was collected by the microplate reader.

1.5 Cellular thermal shift assay

HCT-116 cells were cultured in cell culture dishes and treated with 5 *μ*M compound 12 for 4 h. HCT-116 cells were collected, washed by PBS and cell suspensions were divided into 12 PCR tubes equally. Then,11 tubes were heated at 37, 40, 43, 46, 49, 52, 55, 58, 61, 64, and 67 °C for 4 min, respectively. After that, cells were broken by ultrasonic treatment and centrifuged at 12000 rpm for 20 min. The supernatant was analyzed by western blot.

1.6 Biotin-pulldown assay

After co-treated with 2 *μ*M biotinylated compound and compound **12** for 12 h, HCT-116 cells were lysed with NP-40 containing PMSF and centrifuged at 14000 r for 30 min at 4 °C. Then, 40 *μ*L supernatant was used as the input by adding 10 μL 5×SDS loading buffer, and the remains were mixed with Streptavidin Agarose (Novex, Carlsbad, CA, USA). After 6 h incubation at 4 °C, the beads were washed three times using lysis buffer and resuspended in 2×SDS loading buffer. HEK-293T cells overexpressed flag-tagged CDK8 were treated using above method. The samples were analyzed by western blot.

1.7 The effect of compound on phosphorylation of STAT1 S727

HCT-116 cells were seeded into 60 mm cultural dishes at a density of 1.5×10^6 cells per dishes. After incubated for 12 h, cells were treated with DMSO or 10ng/mL IFN-γ.1 hour later, cells were treated with or without different concentrations of compound for 12 h. Cells were lysed using RIPA containing PMSF and phosphatase inhibitor cocktail A. Then cell samples were treated by western blot. Levels of total STAT1, phosphorylated STAT1 S727, Y701 and GAPDH were analyzed.

1.8 The effect of compound on phosphorylation of STAT5 S726

HL-60 cells were seeded into 60 mm cultural dishes at a density of 2×10^6 cells per dishes. After incubated for 12 h, cells were treated with or without different concentrations of compound for 12 h. Cells were lysed using RIPA containing PMSF and phosphatase inhibitor cocktail A. Then cell samples were treated by western blot. Levels of total STAT5, phosphorylated STAT5 S726 and GAPDH were analyzed.

Statistical Analyses

The results were expressed as the means ± SEMs (SPSS software), and the differences between groups were evaluated using Turkey's method (GraphPad Software). Differences between data were considered significant when the P value was <0.05.

3-(5H-pyrrolo[2,3-b]pyrazin-2-yl)benzamide (**1**)

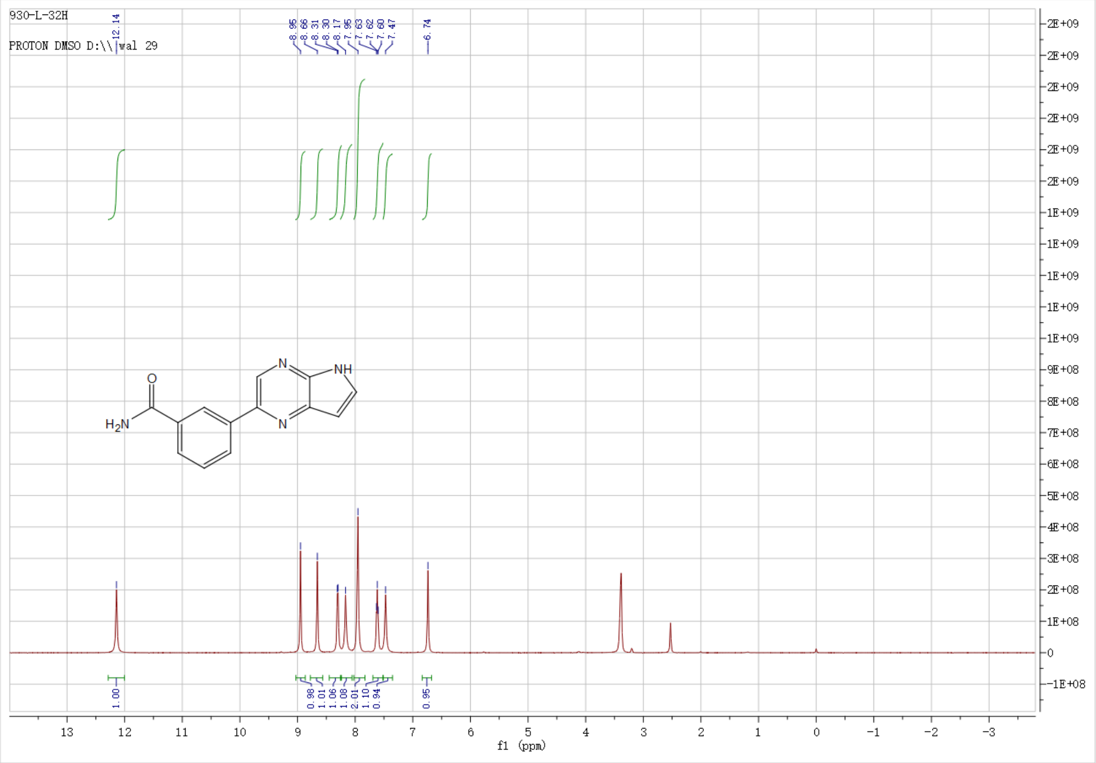

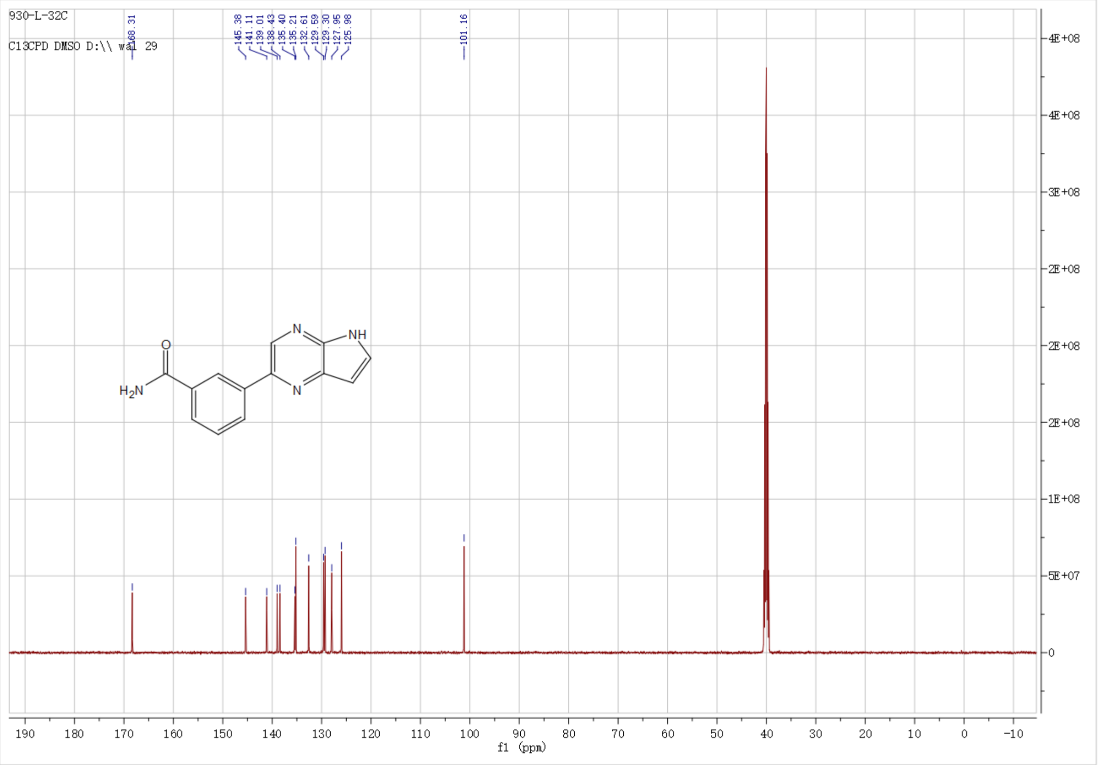

$C_{13}H_{10}N_4O$

[illegible]: 239.0927

Sample Name	ZXX-0719-3	Position	Vial 42	Instrument Name	Instrument 1	User Name	
Inj Vol	2	InjPosition		SampleType	Sample	IRM Calibration Status	All Ions Missed
Data Filename	ZXX-0719-3.d	ACQ Method	2020LXSpo.m	Comment		Acquired Time	7/19/2021 4:40:34 PM

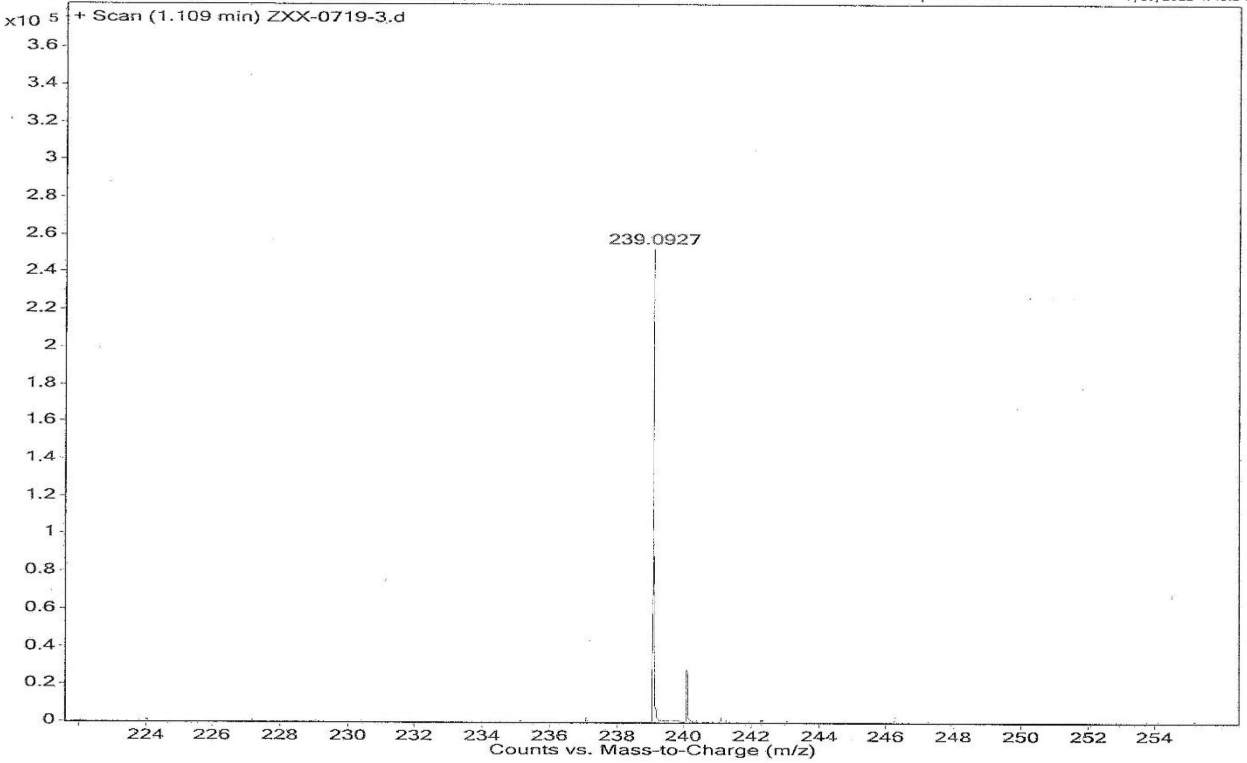

0718-1

3-(1H-pyrazolo[3,4-b]pyridin-5-yl)benzamide (**2**)

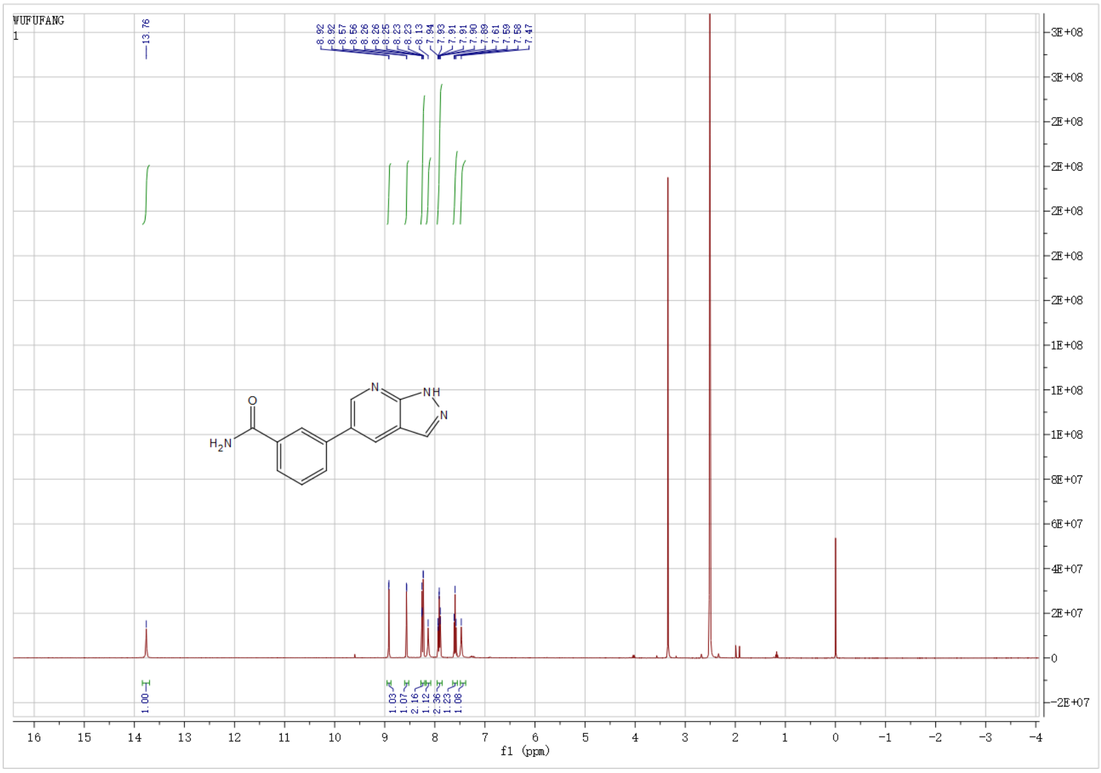

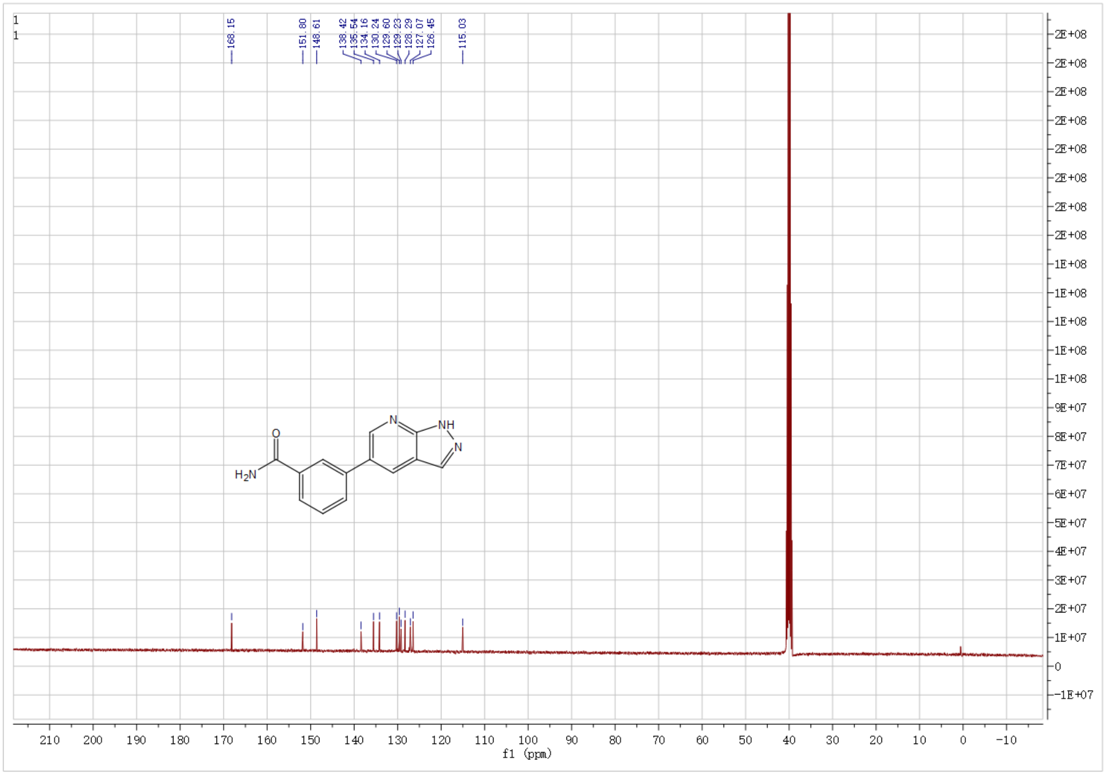

cal[M+H]+: 239.0927

**Sample Name**	ZXX-20220315-21	**Position**	Vial 1	**Instrument Name**	Instrument 1	**User Name**	
**Inj Vol**	2	**InjPosition**		**SampleType**	Sample	**IRM Calibration Status**	Success
**Data Filename**	ZXX-20220315-21.d	**ACQ Method**	2020LXSpo.m	**Comment**		**Acquired Time**	3/15/2022 9:53:07 PM

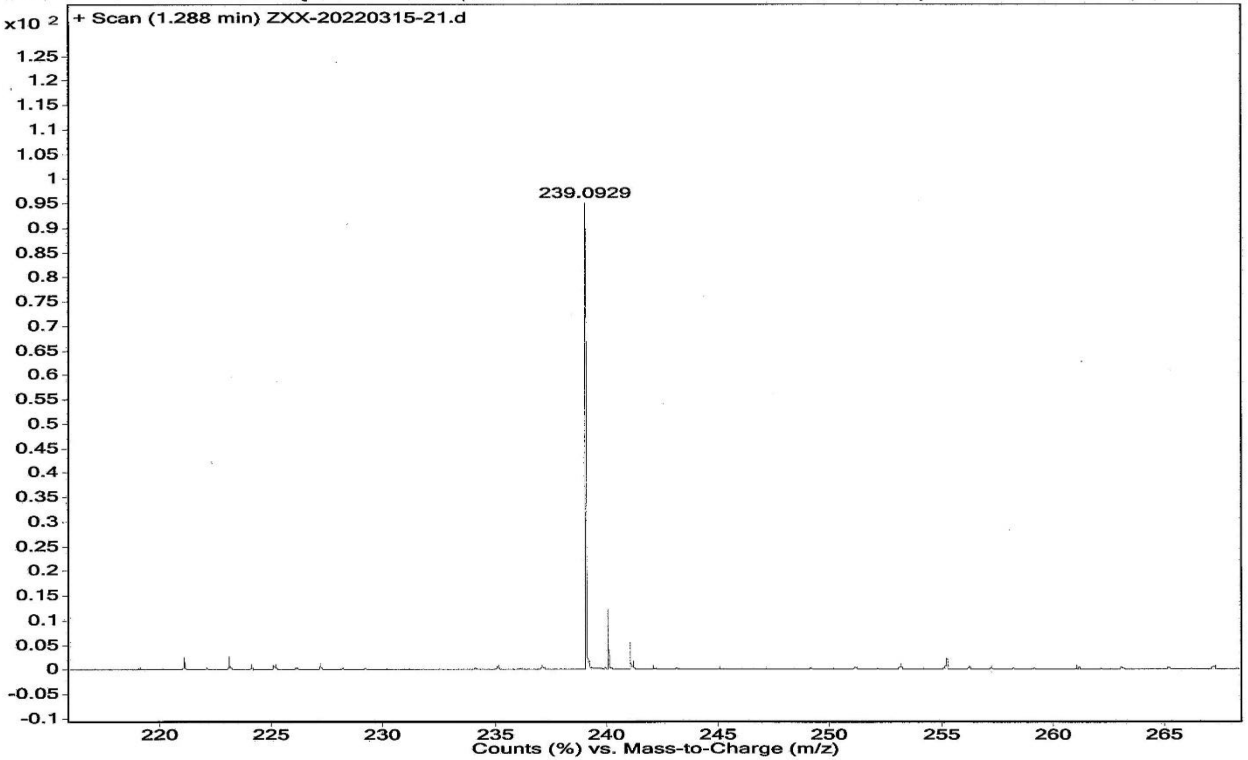

0304-1

3-(6-aminopyridin-3-yl)benzamide (**3**)

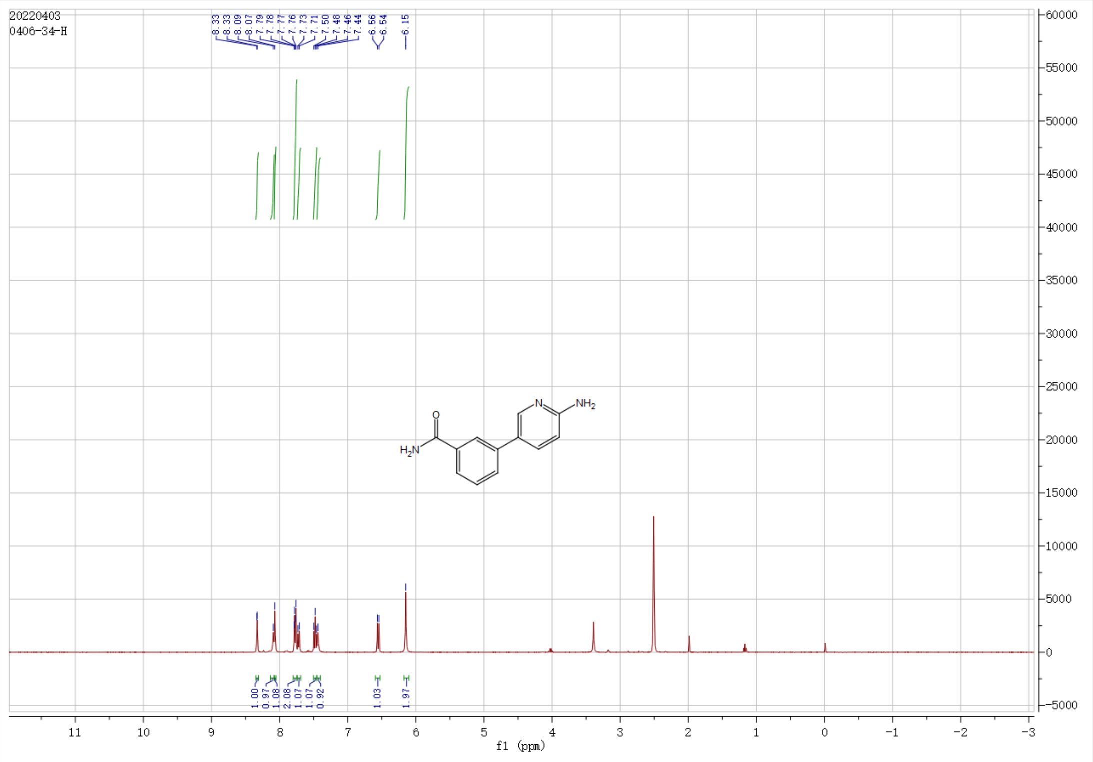

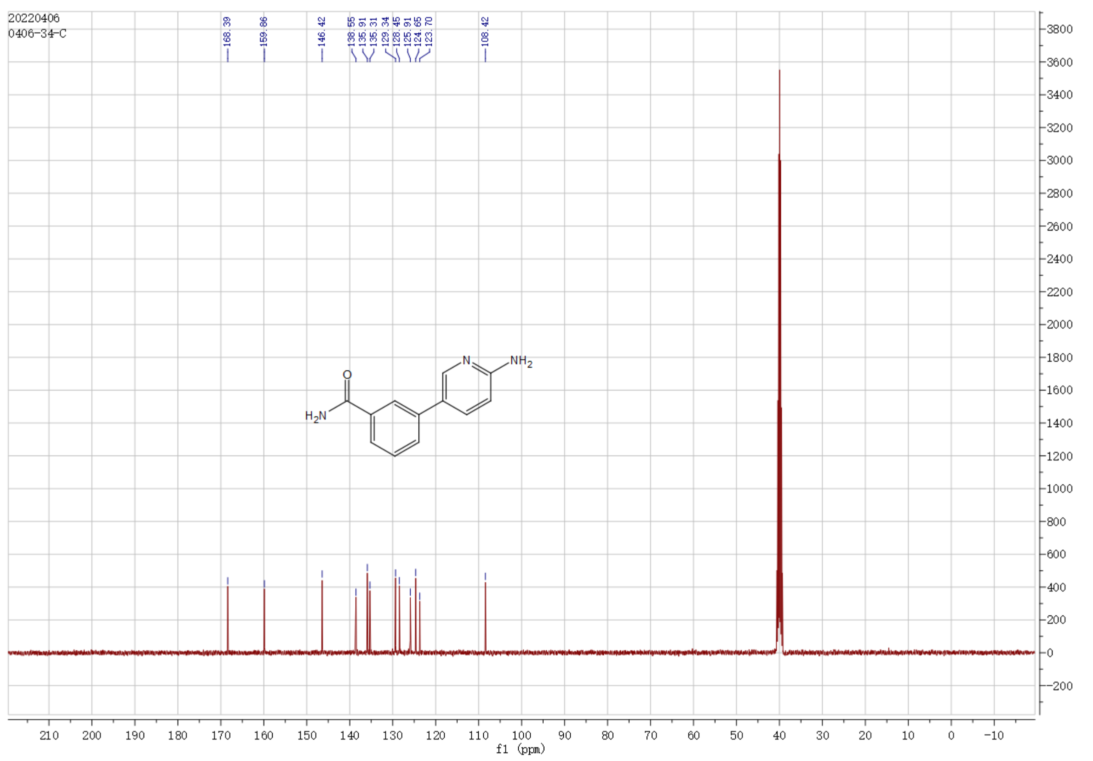

**Sample Name**	ZXX-2022-0321-115	**Position**	Vial 15	**Instrument Name**	Instrument 1	**User Name**	
**Inj Vol**	2	**InjPosition**		**SampleType**	Sample	**IRM Calibration Status**	All Ions Missed
**Data Filename**	ZXX-2022-0321-115.d	**ACQ Method**	2020LXSpo.m	**Comment**		**Acquired Time**	3/21/2022 9:33:38 PM

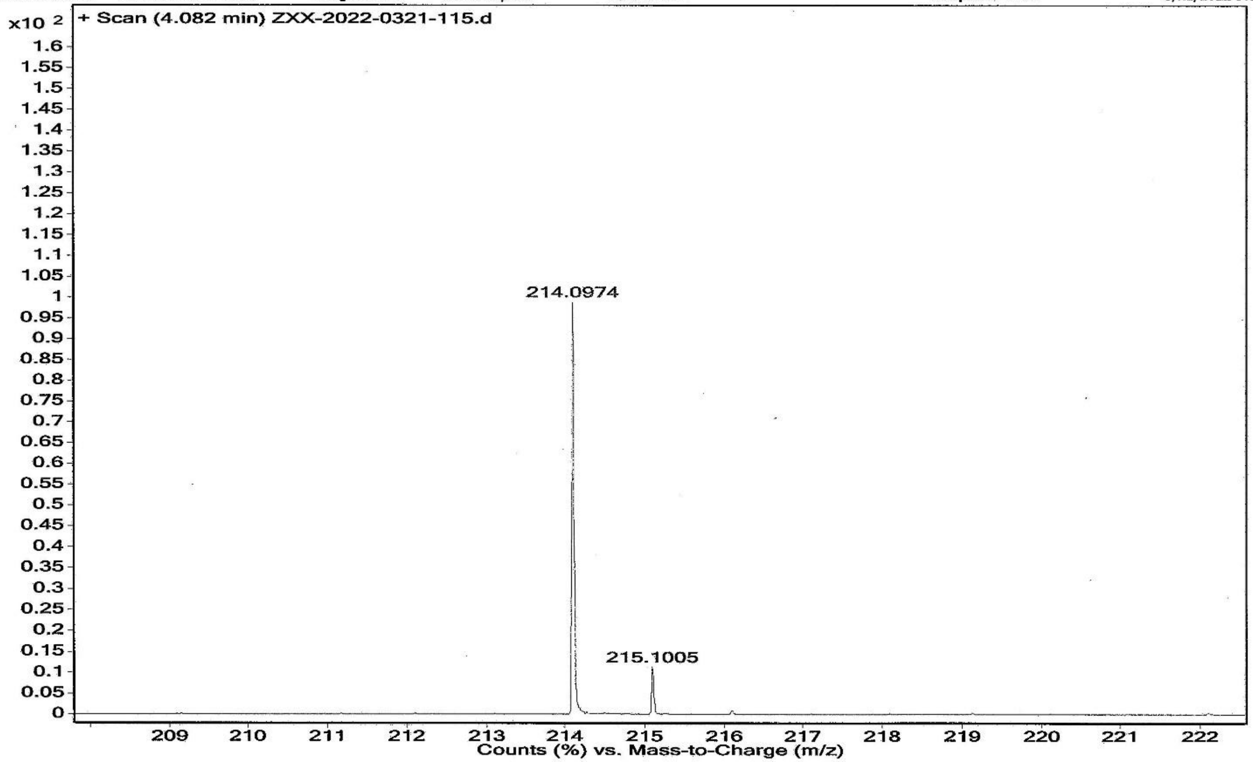

0321-3

3-(6-amino-5-chloropyridin-3-yl)benzamide (**4**)

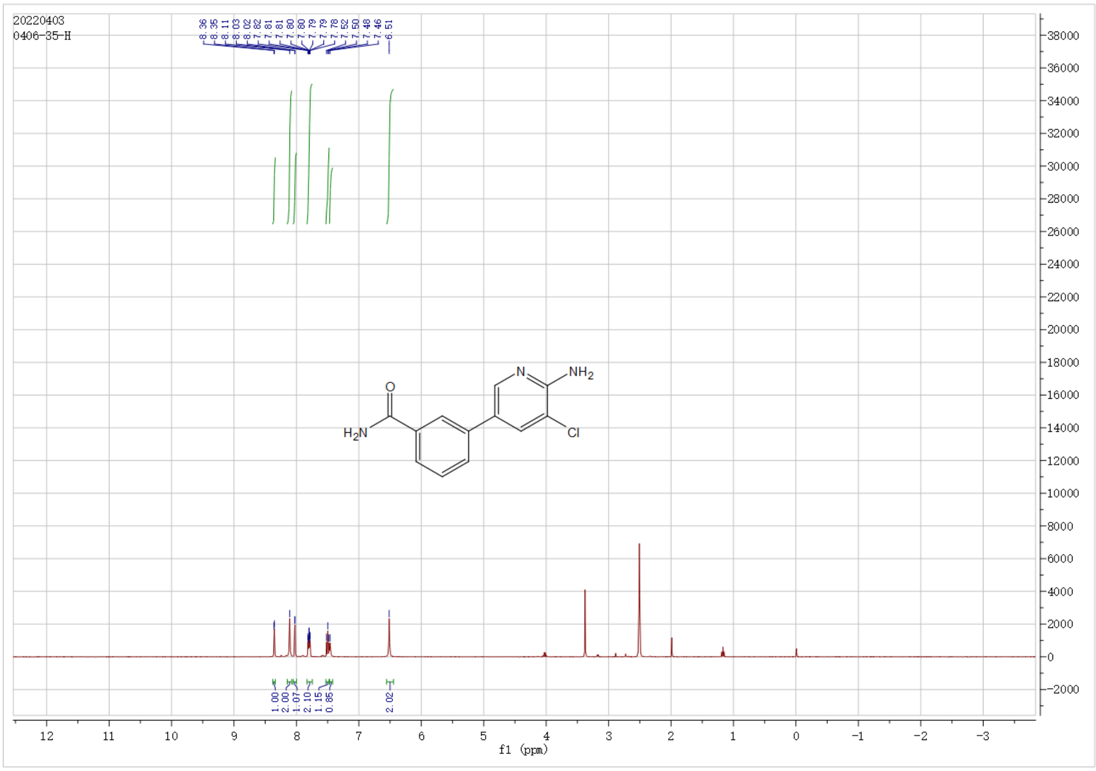

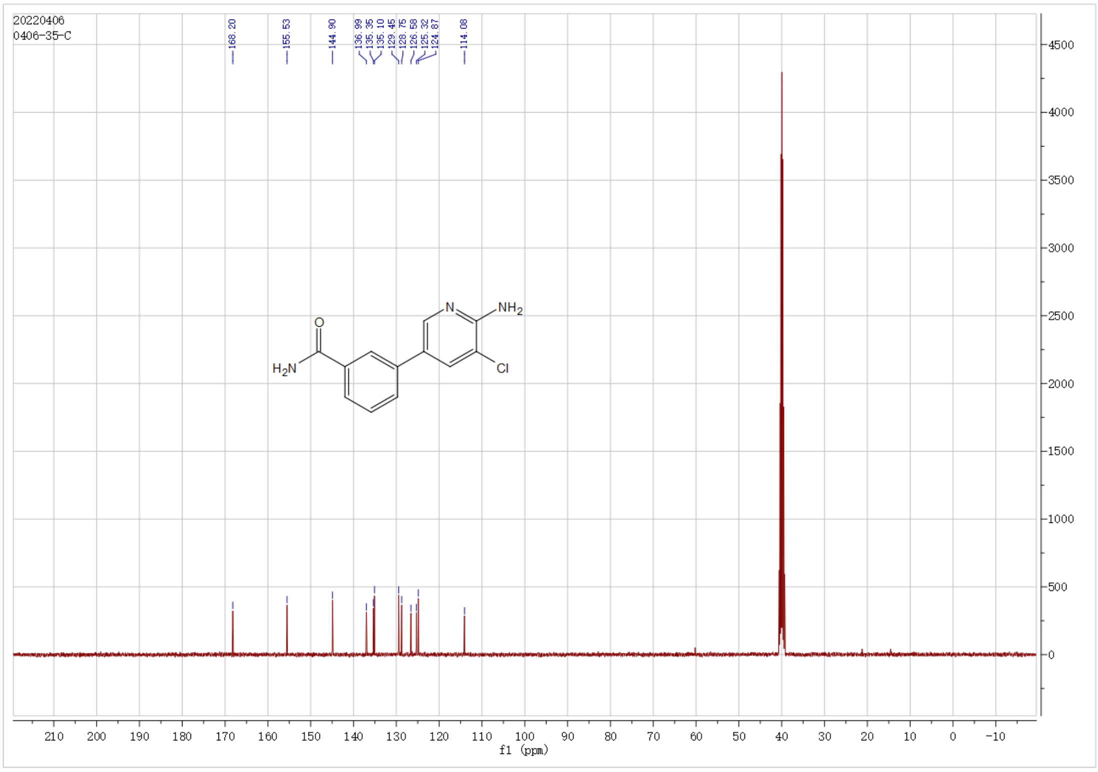

**Sample Name**	ZXX-2022-0321-116	**Position**	Vial 16	**Instrument Name**	Instrument 1	**User Name**	
**Inj Vol**	2	**InjPosition**		**SampleType**	Sample	**IRM Calibration Status**	All Ions Missed
**Data Filename**	ZXX-2022-0321-116.d	**ACQ Method**	2020LXSpo.m	**Comment**		**Acquired Time**	3/21/2022 9:40:25 PM

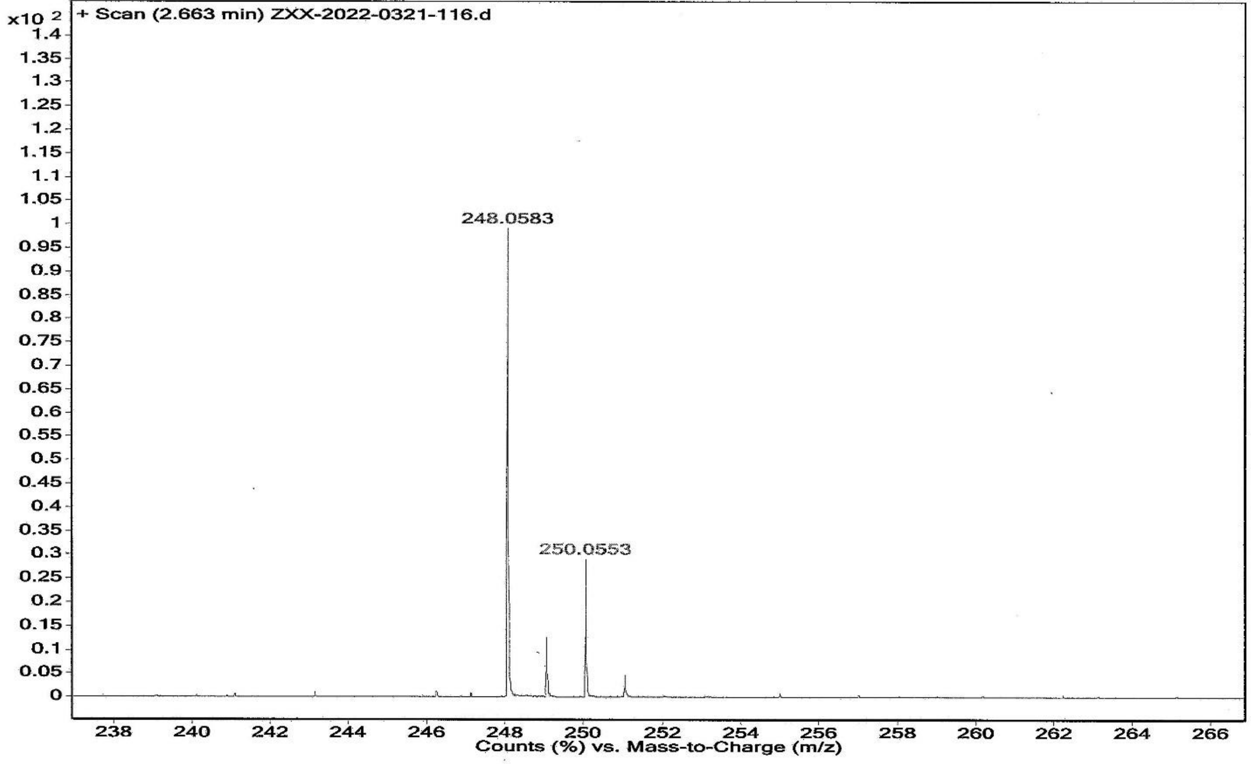

0321-4

3-(isoquinolin-4-yl)benzamide (**5**)

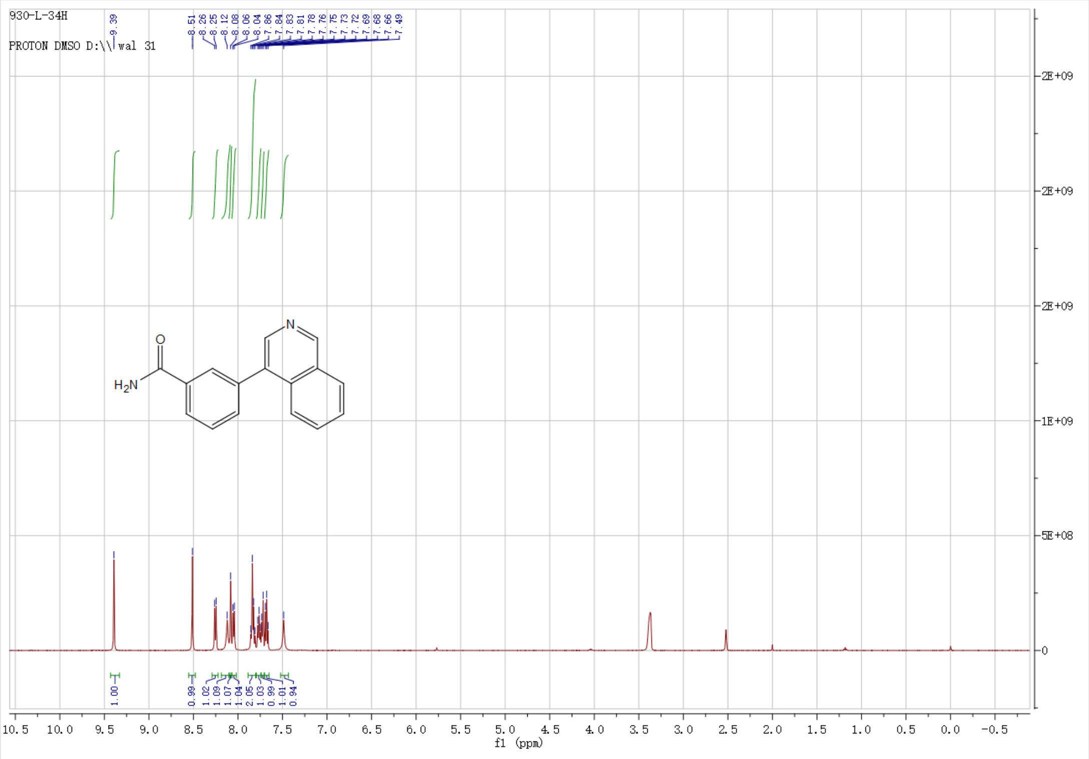

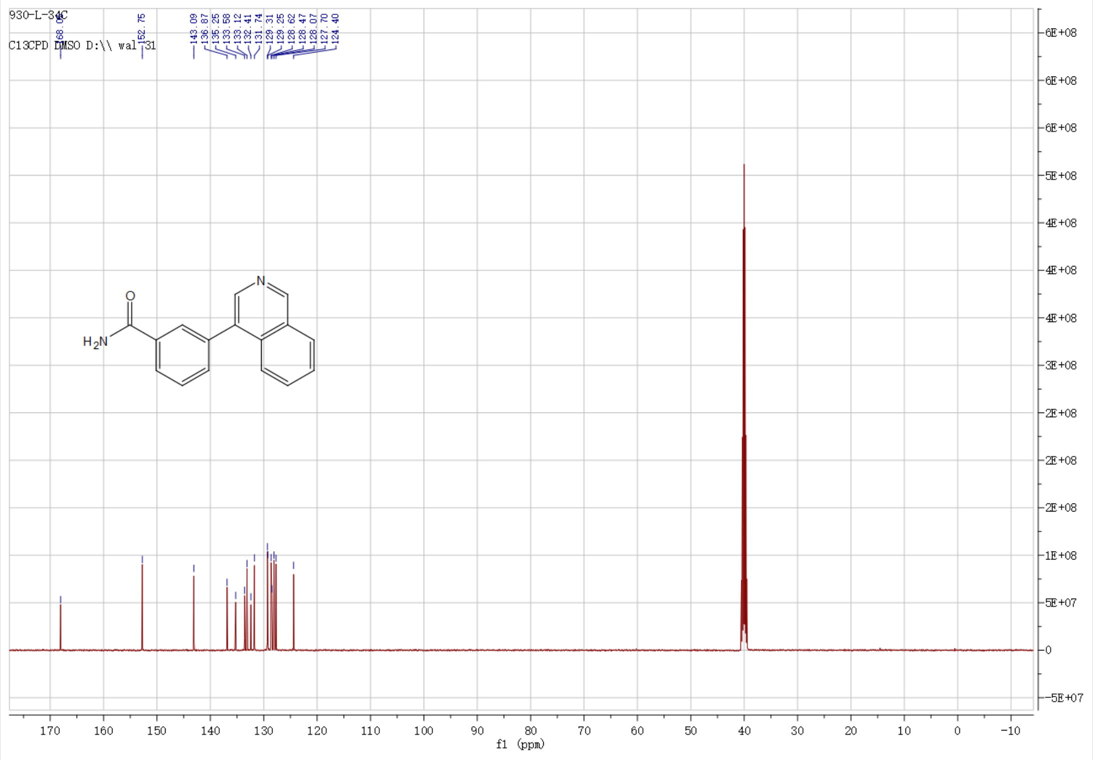

$C_{16}H_{12}N_2O$
[illegible]: 249.1022

Sample Name	ZXX-0722-4	Position	Vial 34	Instrument Name	Instrument 1	User Name	
Inj Vol	2	InjPosition		SampleType	Sample	IRM Calibration Status	All Ions Missed
Data Filename	ZXX-0722-4.d	ACQ Method	2020LXSpo.m	Comment		Acquired Time	7/22/2021 11:04:01 AM

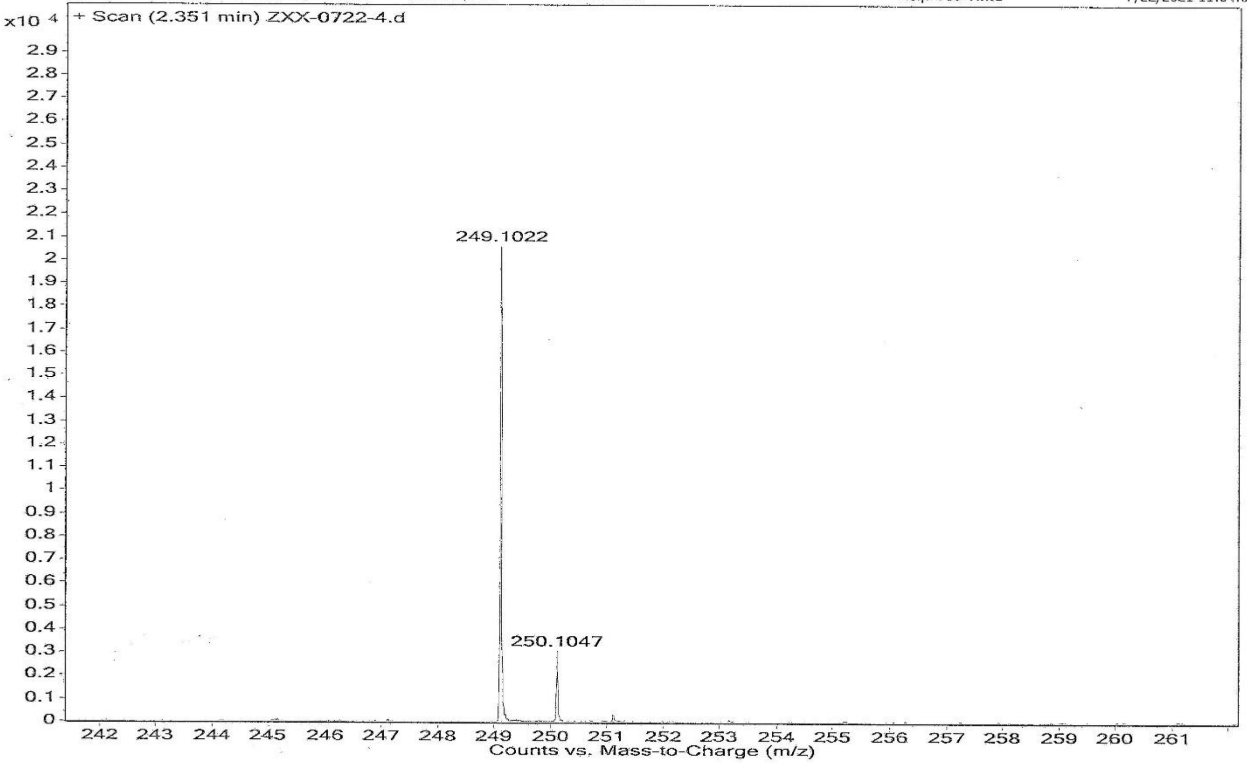

0719-2
305

3-(1-aminoisoquinolin-4-yl)benzamide (**6**)

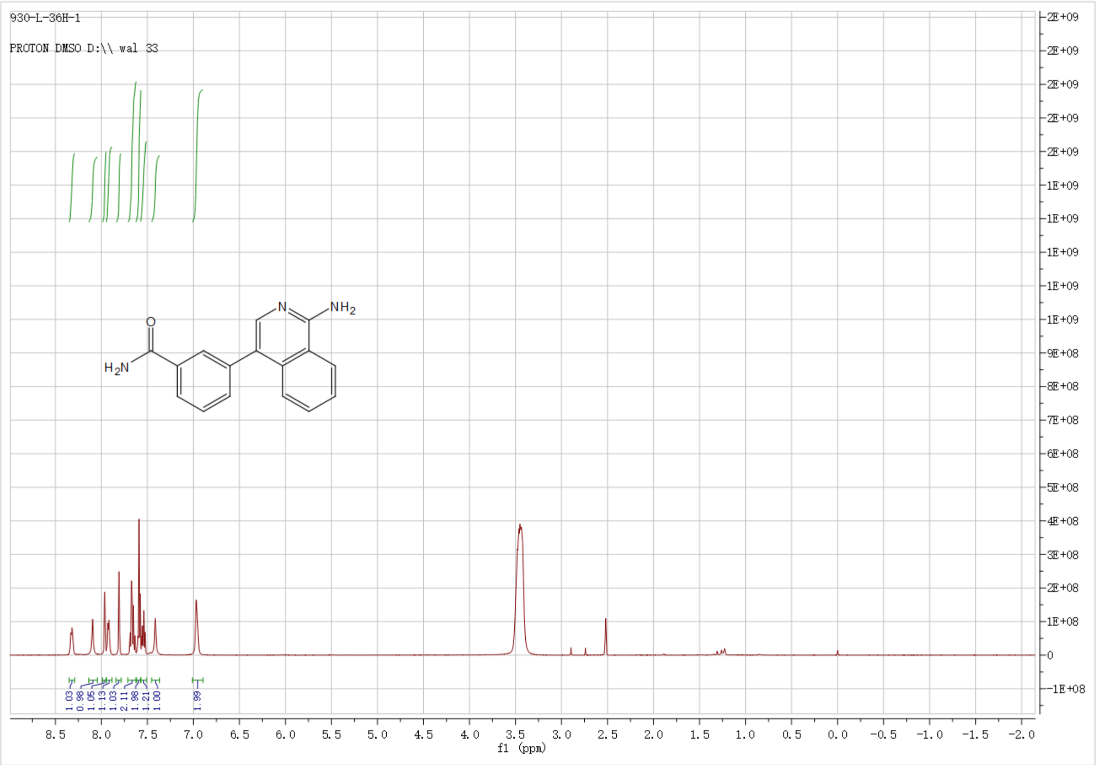

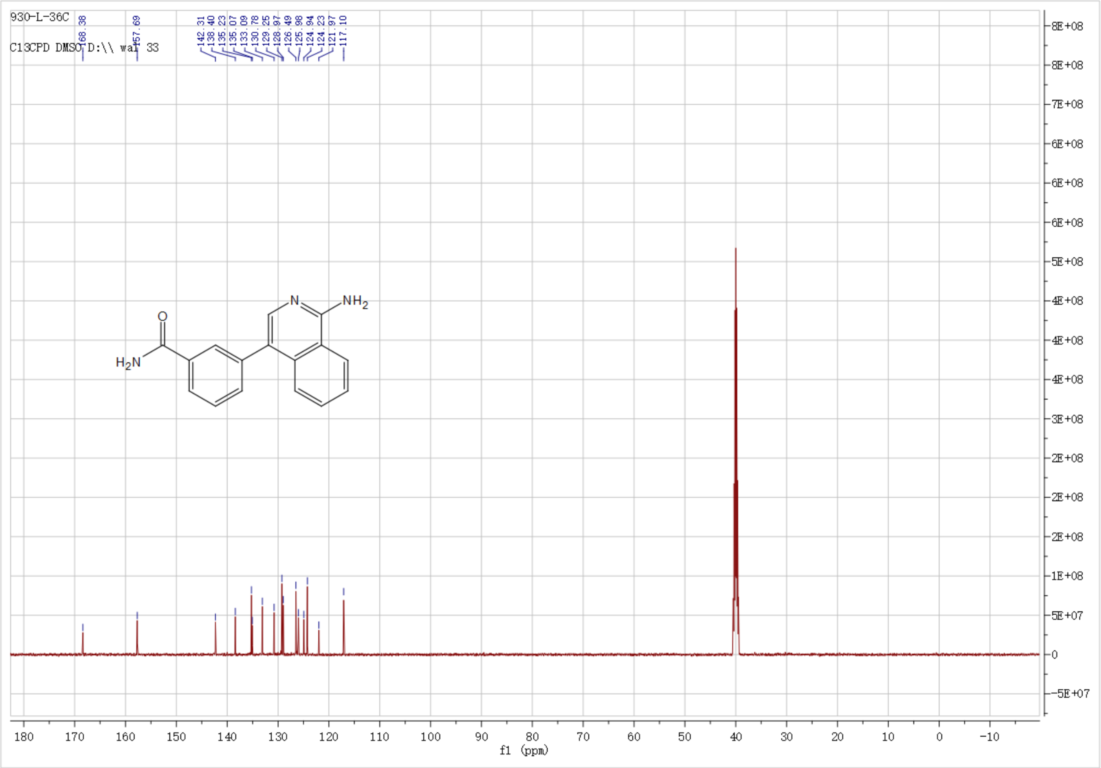

Sample Name	ZXX-0722-3	Position	Vial 33	Instrument Name	Instrument 1	User Name	
Inj Vol	2	InjPosition		SampleType	Sample	IRM Calibration Status	All Ions Missed
Data Filename	ZXX-0722-3.d	ACQ Method	2020LXSpo.m	Comment		Acquired Time	7/22/2021 10:57:13 AM

$C_{16}H_{13}N_3O$

[illegible]: 264.1131

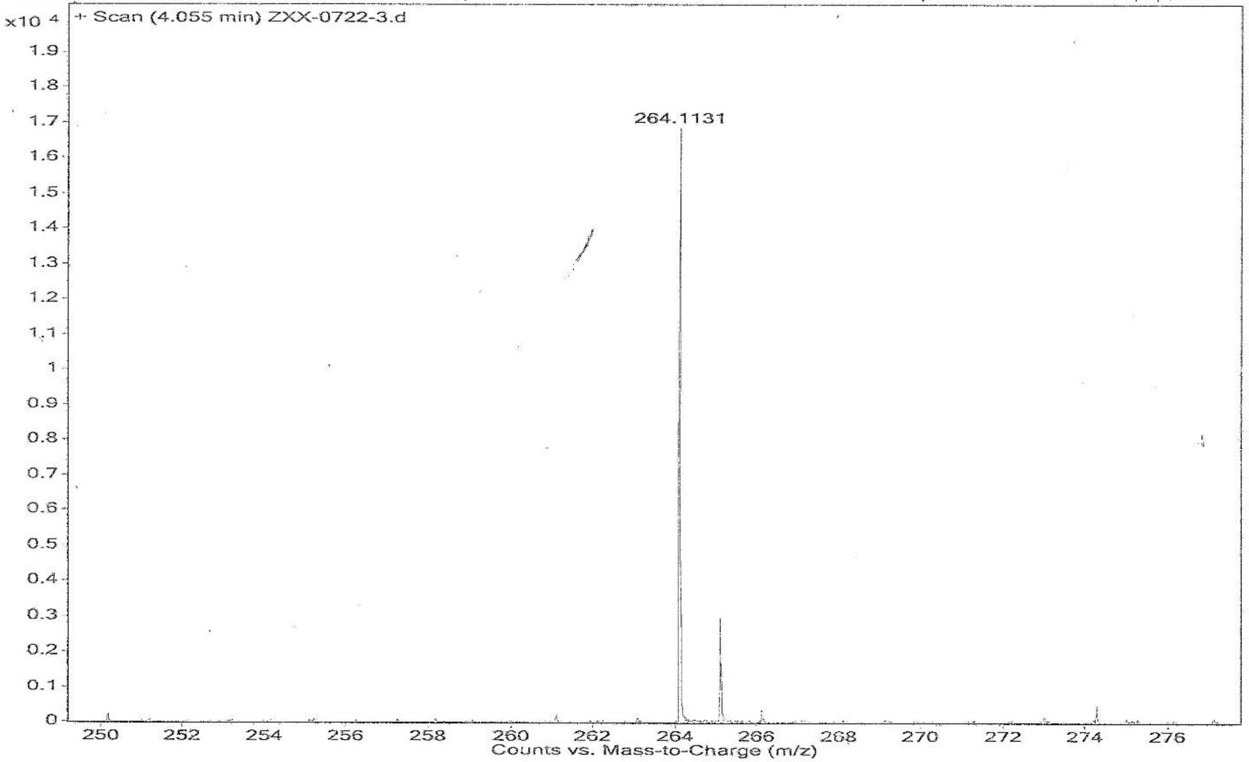

3-(1H-indol-5-yl)benzamide (**7**)

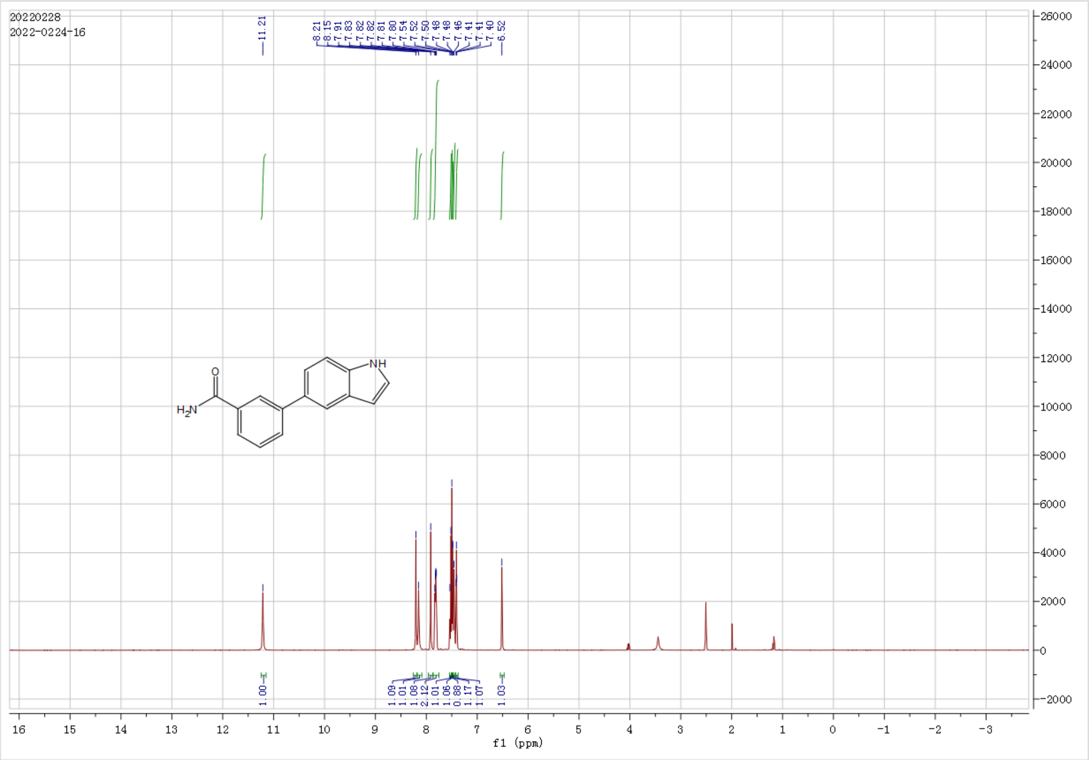

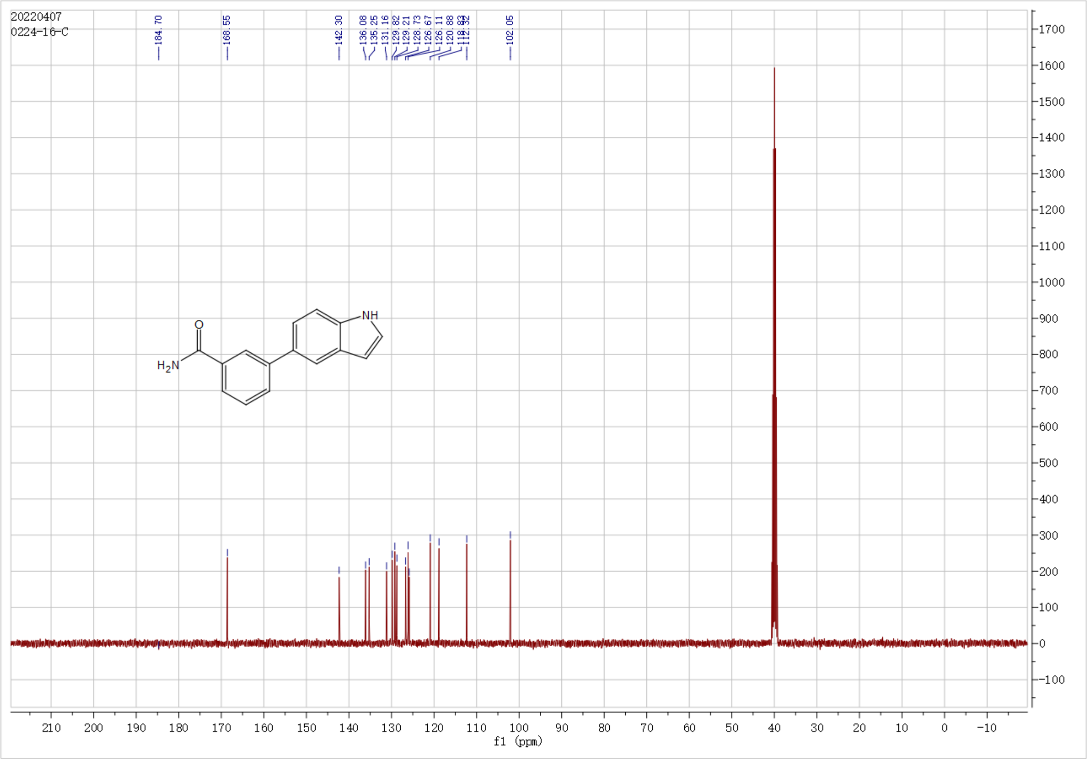

**Sample Name**	ZXX-2022-0224-116	**Position**	Vial 16	**Instrument Name**	Instrument 1	**User Name**	
**Inj Vol**	2	**InjPosition**		**SampleType**	Sample	**IRM Calibration Status**	All Ions Missed
**Data Filename**	ZXX-2022-0224-116.d	**ACQ Method**	2020LXSpo.m	**Comment**		**Acquired Time**	2/24/2022 11:57:43 PM

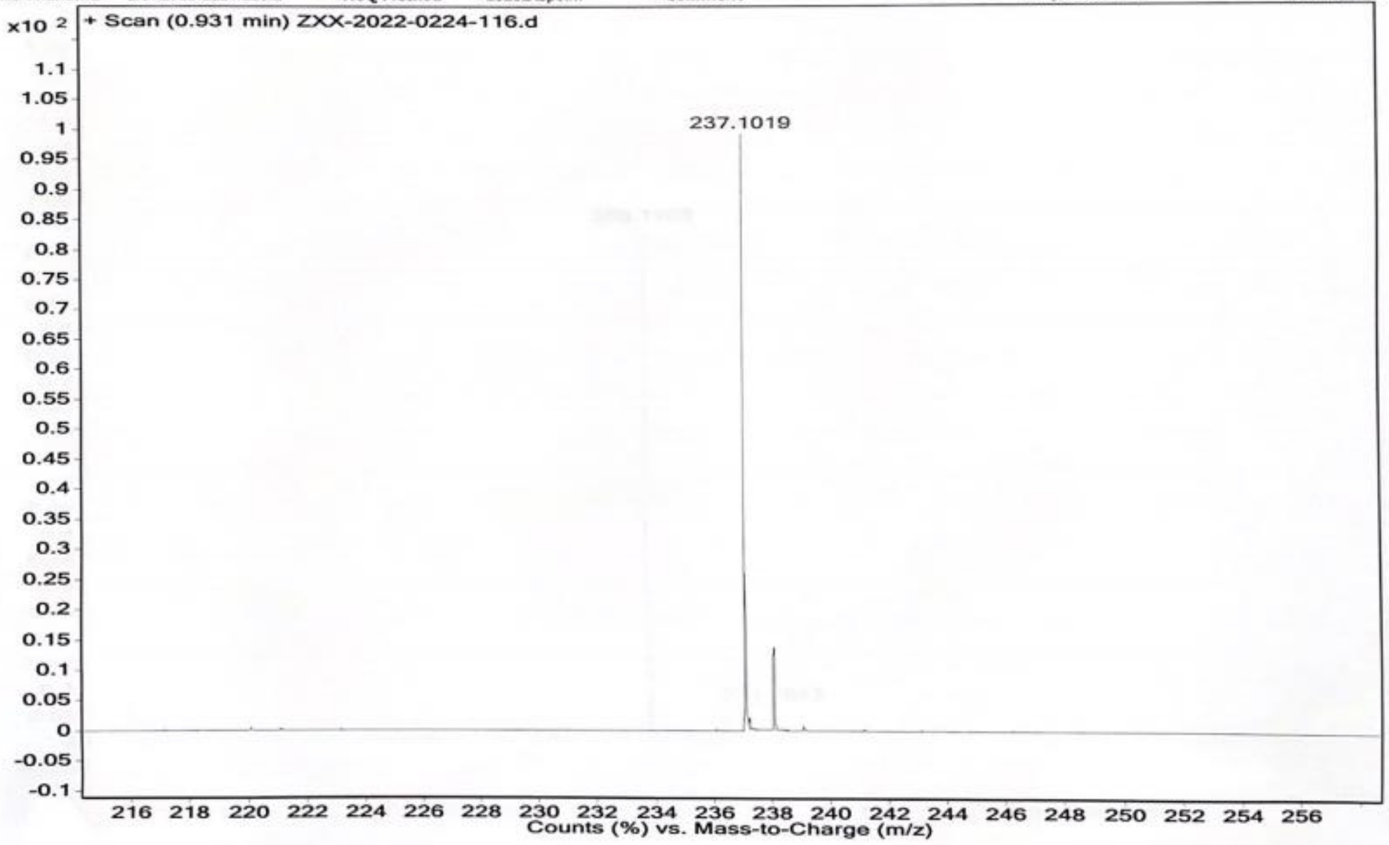

3-(1-methyl-1H-indol-5-yl)benzamide (**8**)

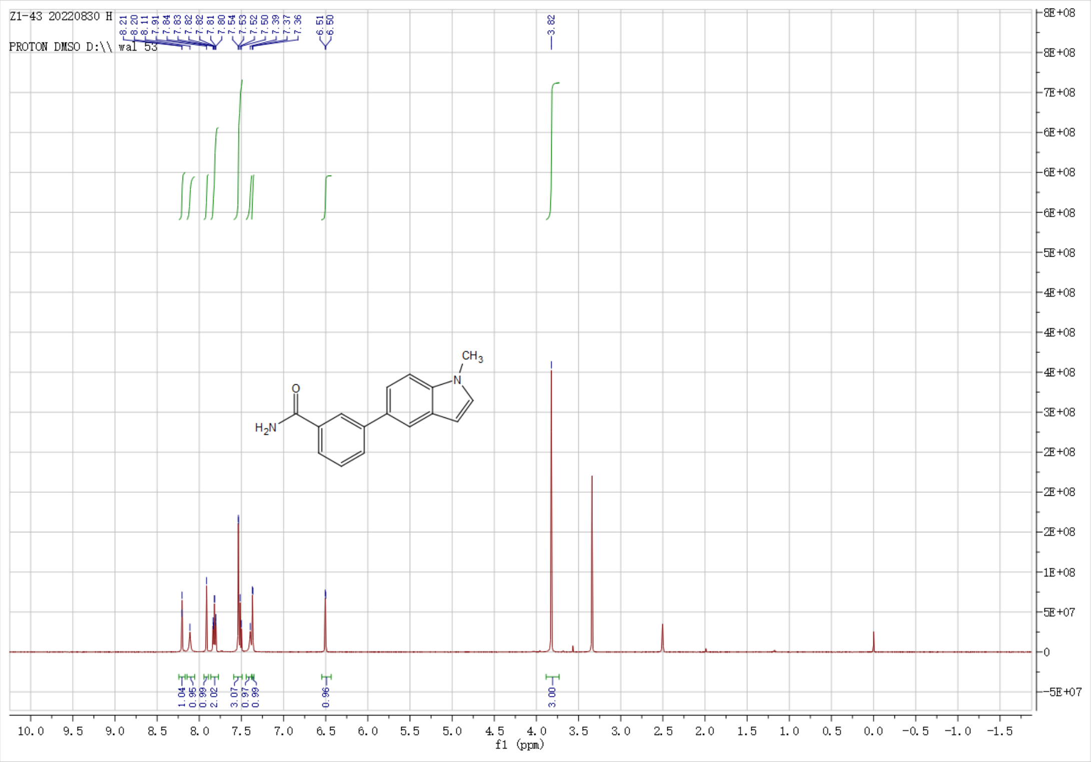

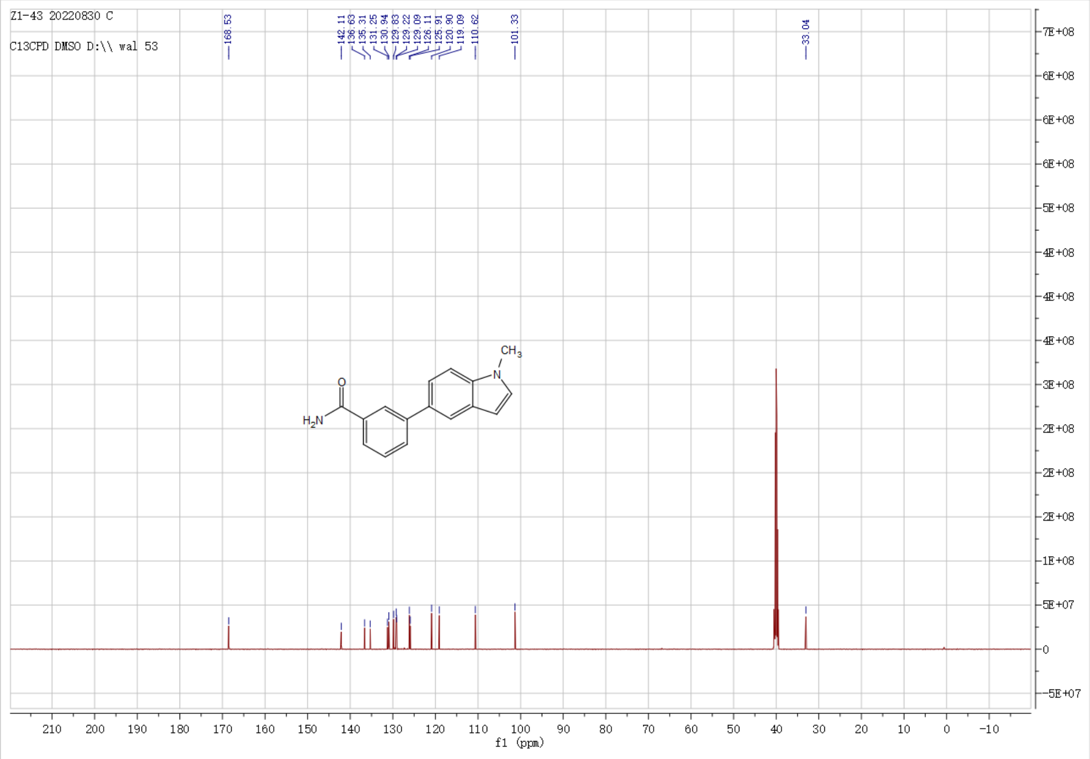

X-2022-0623-116	**Position**	Vial 16	**Instrument Name**	Instrument 1	**User Name**	
	**InjPosition**		**SampleType**	Sample	**IRM Calibration Status**	
X-2022-0623-116.d	**ACQ Method**	2020LXSpo.m	**Comment**		**Acquired Time**	

n (0.874 min) ZXX-2022-0623-116.d

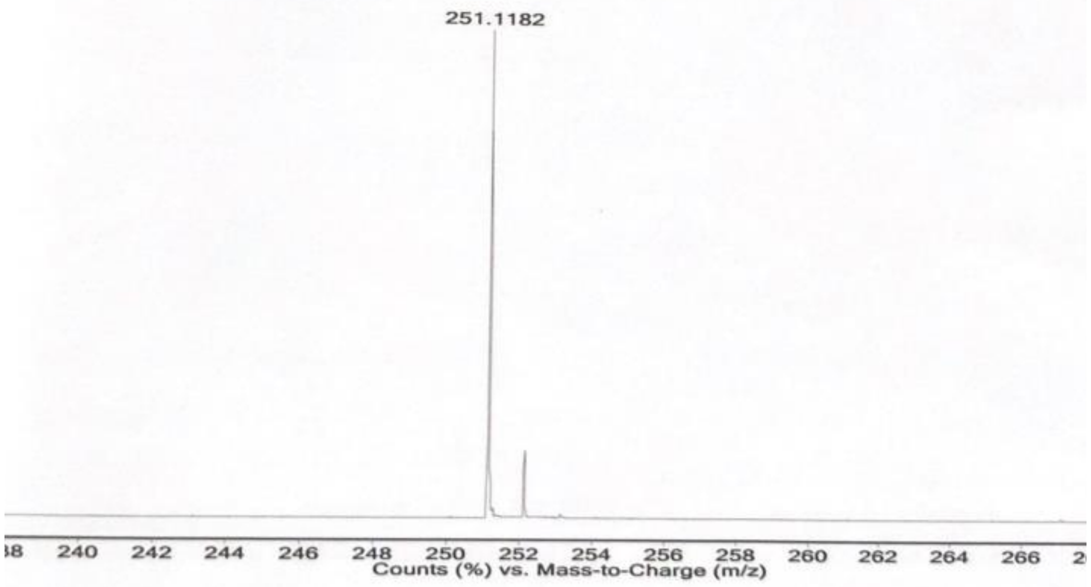

3-(benzofuran-5-yl)benzamide (**9**)

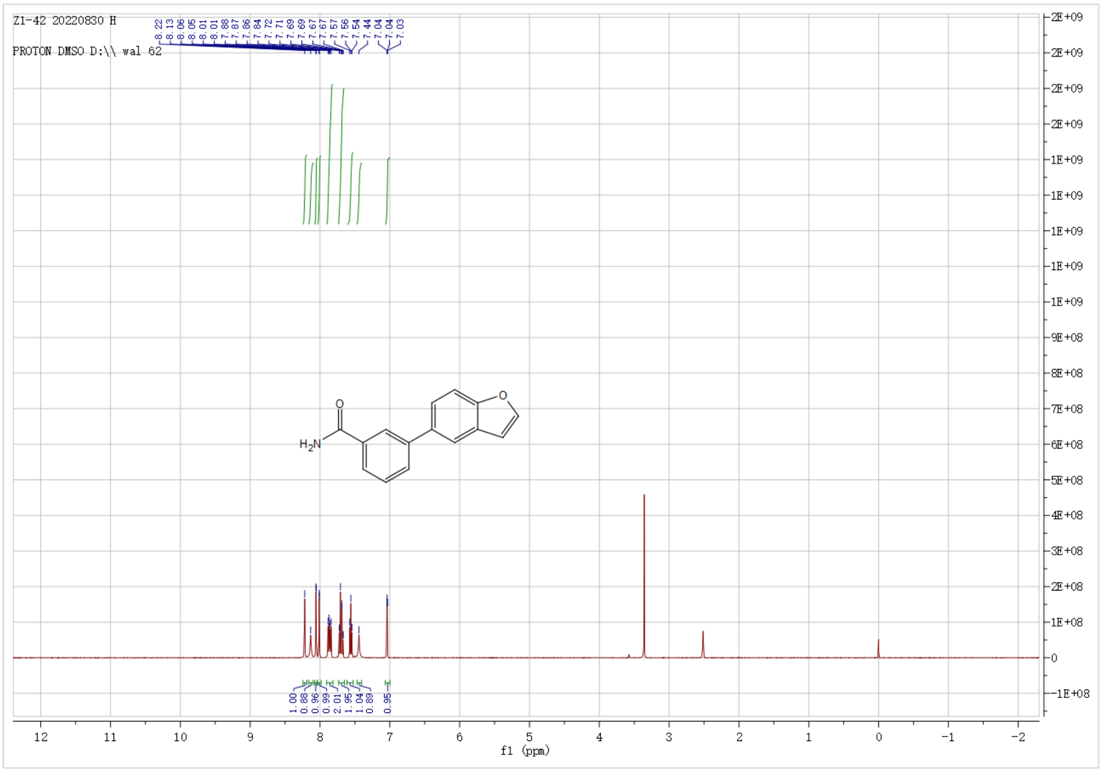

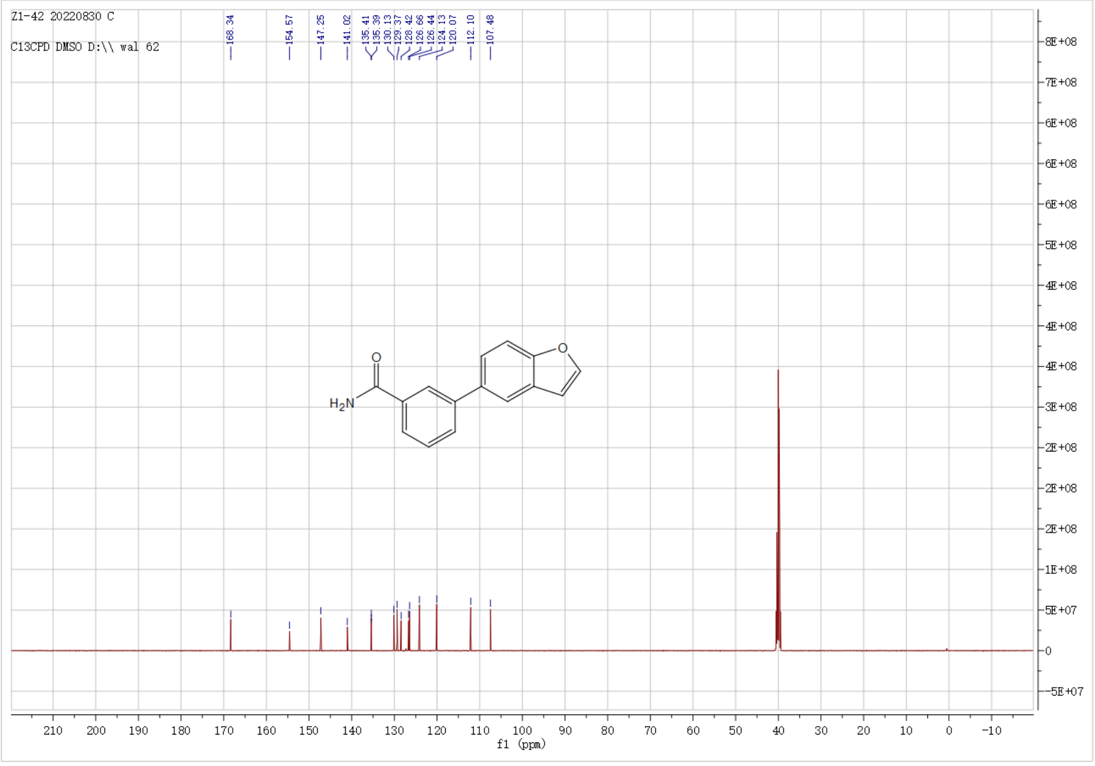

**Sample Name**	ZXX-2022-0623-115	**Position**	Vial 15	**Instrument Name**	Instrument 1	**User Name**	
**Inj Vol**	2	**InjPosition**		**SampleType**	Sample	**IRM Calibration Status**	Some Ions Missed
**Data Filename**	ZXX-2022-0623-115.d	**ACQ Method**	2020LXSpo.m	**Comment**		**Acquired Time**	6/23/2022 9:22:46 PM

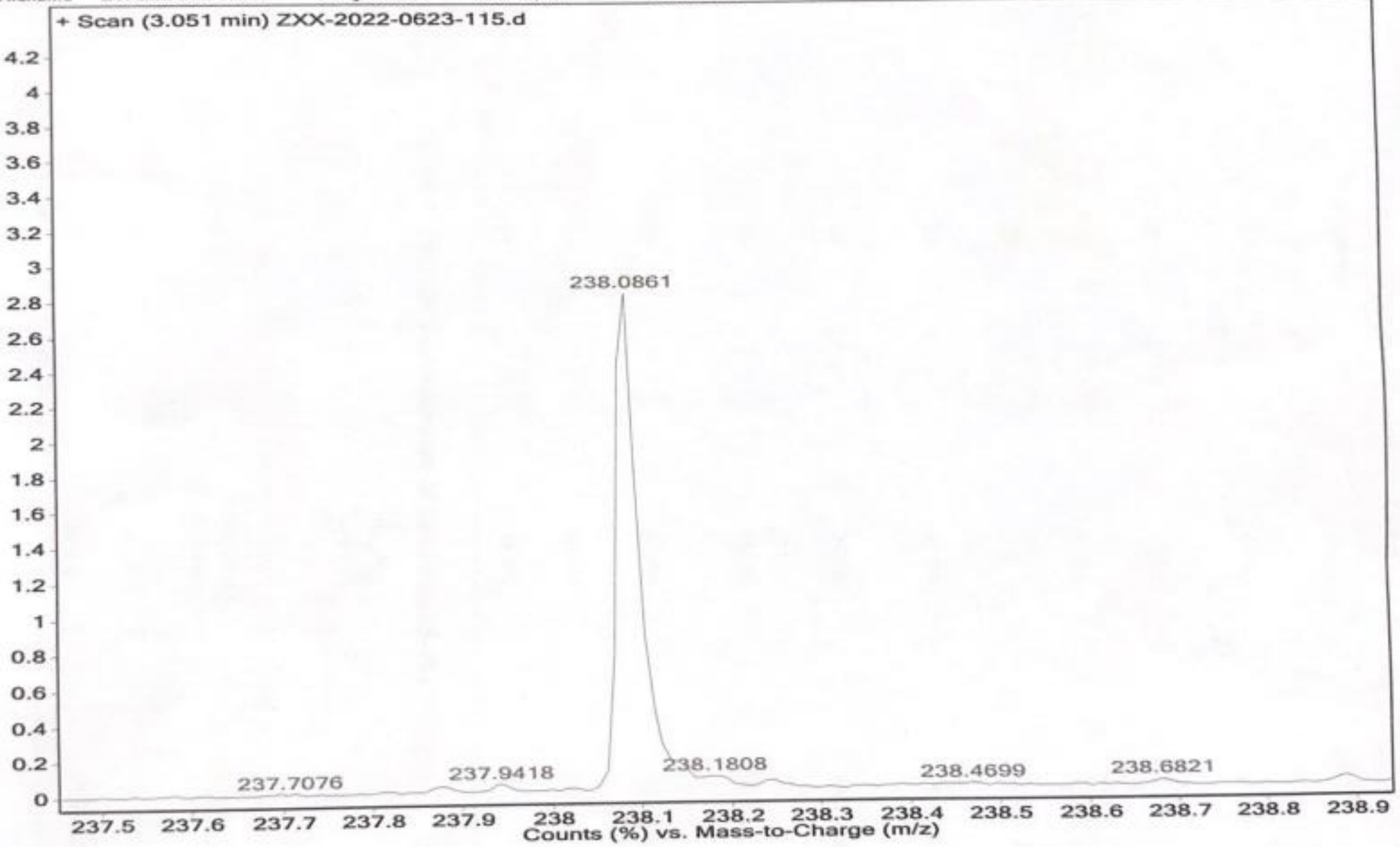

3-(benzo[b]thiophen-5-yl)benzamide (**10**)

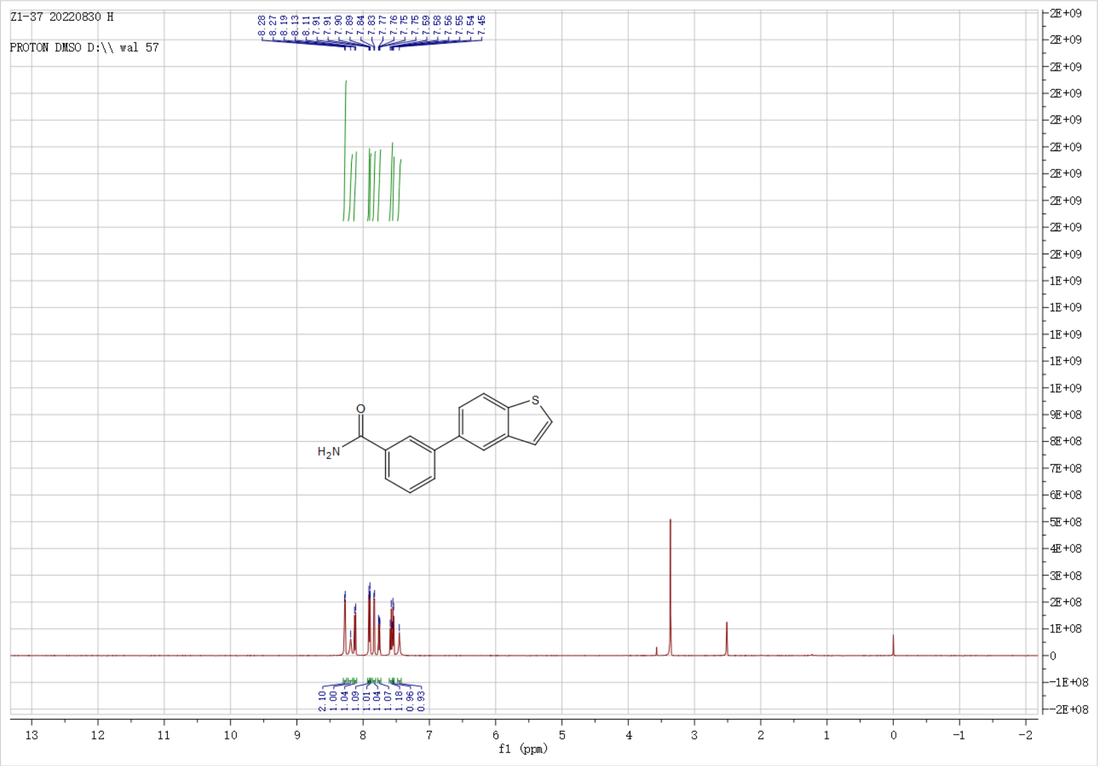

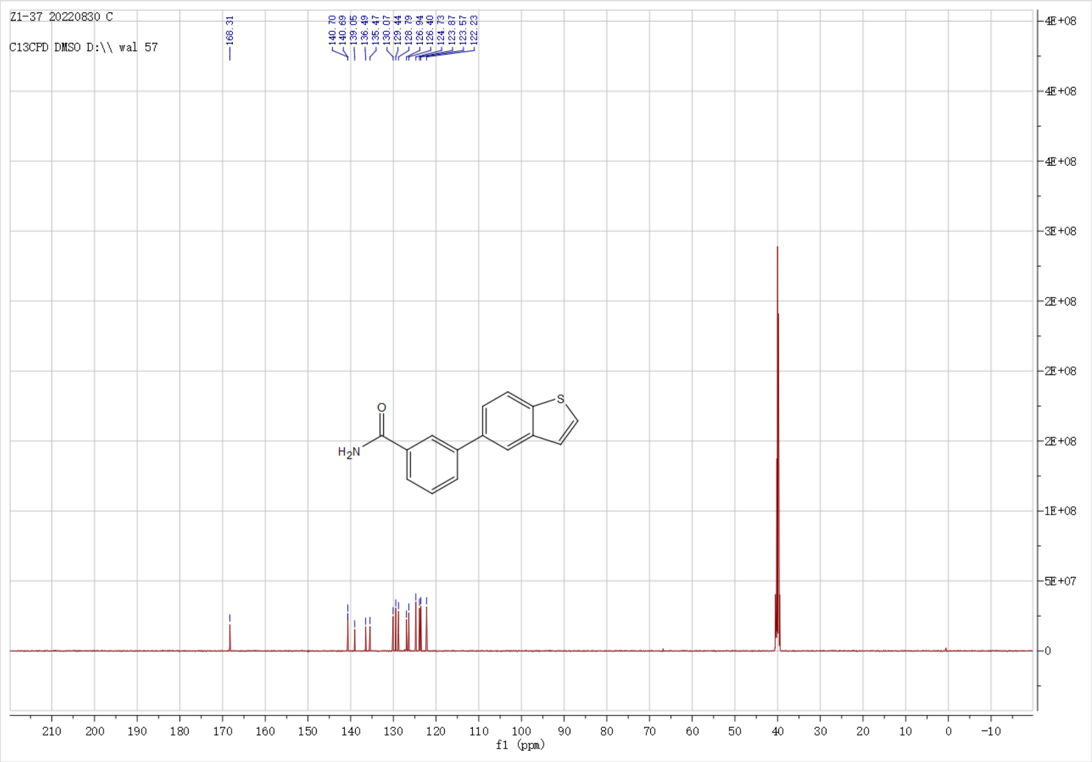

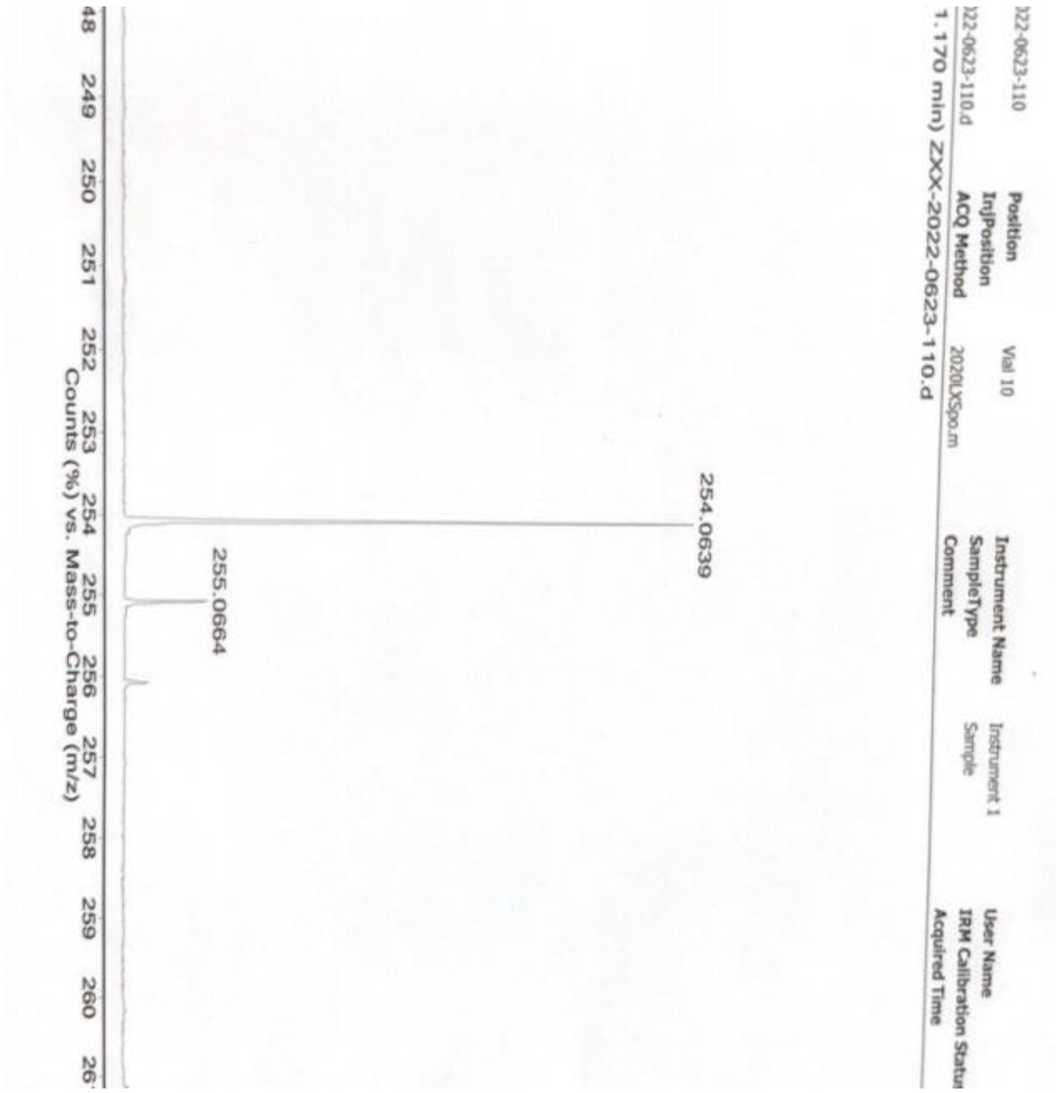

3-(3-chloro-1H-pyrrolo[2,3-b]pyridin-5-yl)benzamide (**11**)

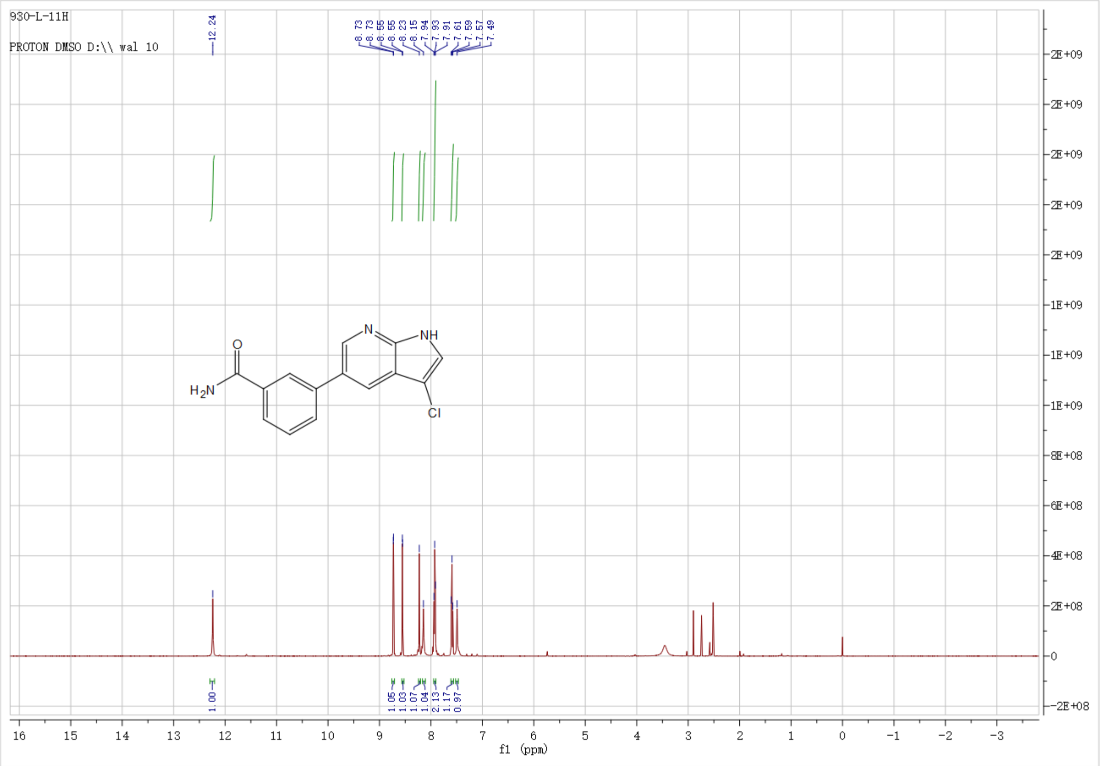
930-L-11H
PROTON DMSO D:\\ wal 10
12.24
8.73
8.73
8.55
8.55
8.23
8.15
7.94
7.93
7.91
7.61
7.59
7.57
7.49
H2N
O
N
NH
Cl
1.00
1.05
1.03
1.07
1.04
2.13
1.17
0.97
f1 (ppm)

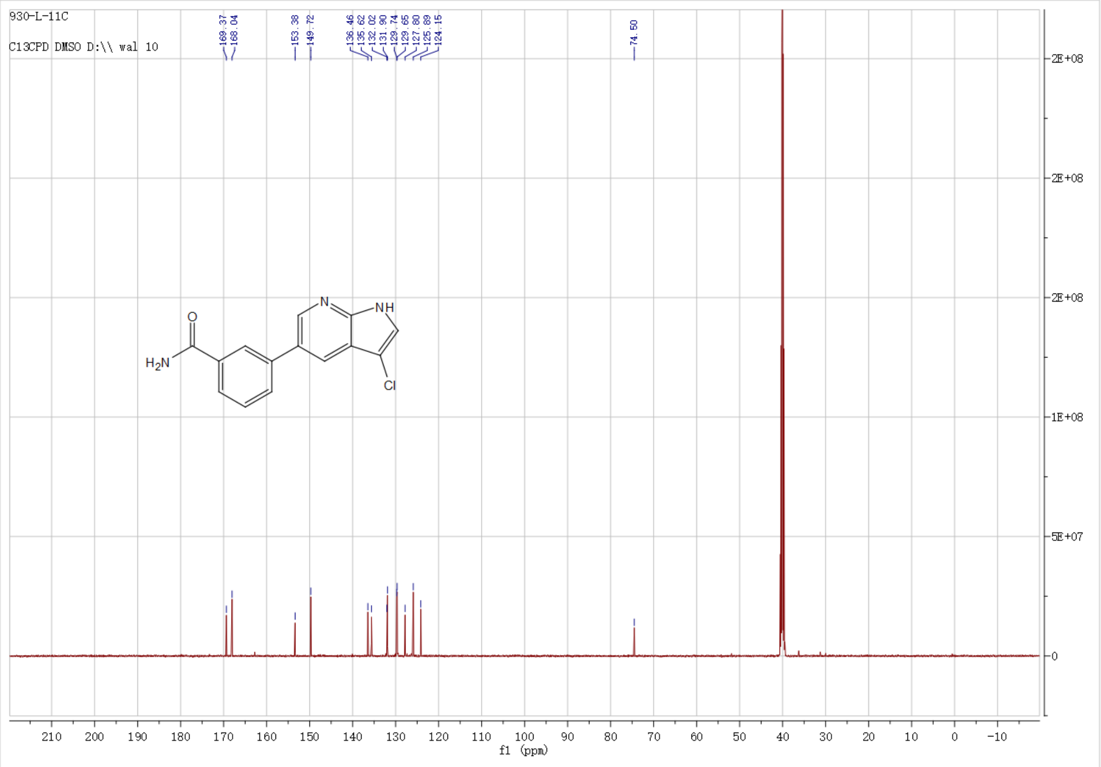
930-L-11C
C13CPD DMSO D:\\ wal 10
169.37
168.04
153.38
149.72
136.46
135.62
132.02
131.90
129.74
129.65
127.80
125.89
124.15
74.50
H2N
O
N
NH
Cl
f1 (ppm)

C14H10N3OCl
Calcd = 272.0585

Sample Name	ZXX-0725-10	Position	Vial 10	Instrument Name	Instrument 1	User Name	
Inj Vol	2	InjPosition		SampleType	Sample	IRM Calibration Status	All Ions Missed
Data Filename	ZXX-0725-10.d	ACQ Method	2020LXSpo.m	Comment		Acquired Time	7/25/2021 9:03:04 AM

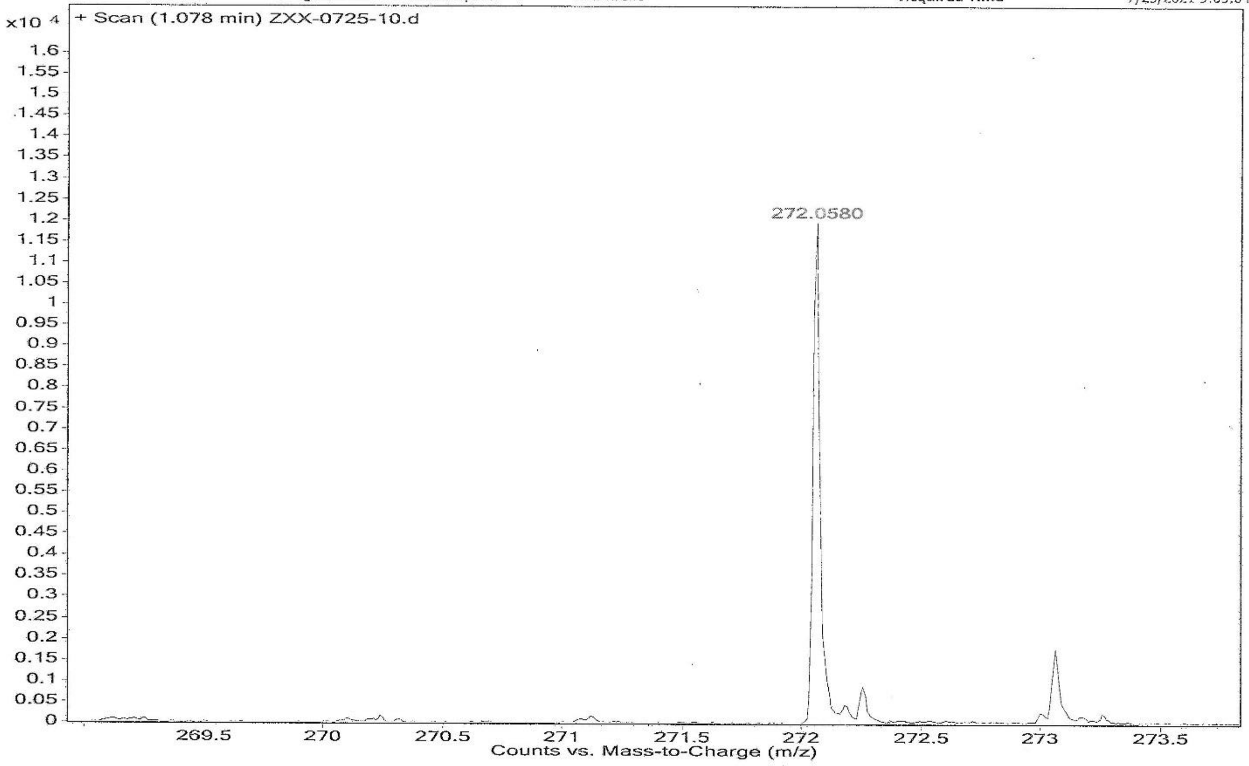

0529-2

3-(3-(furan-3-yl)-1H-pyrrolo[2,3-b]pyridin-5-yl)benzamide (**12**)

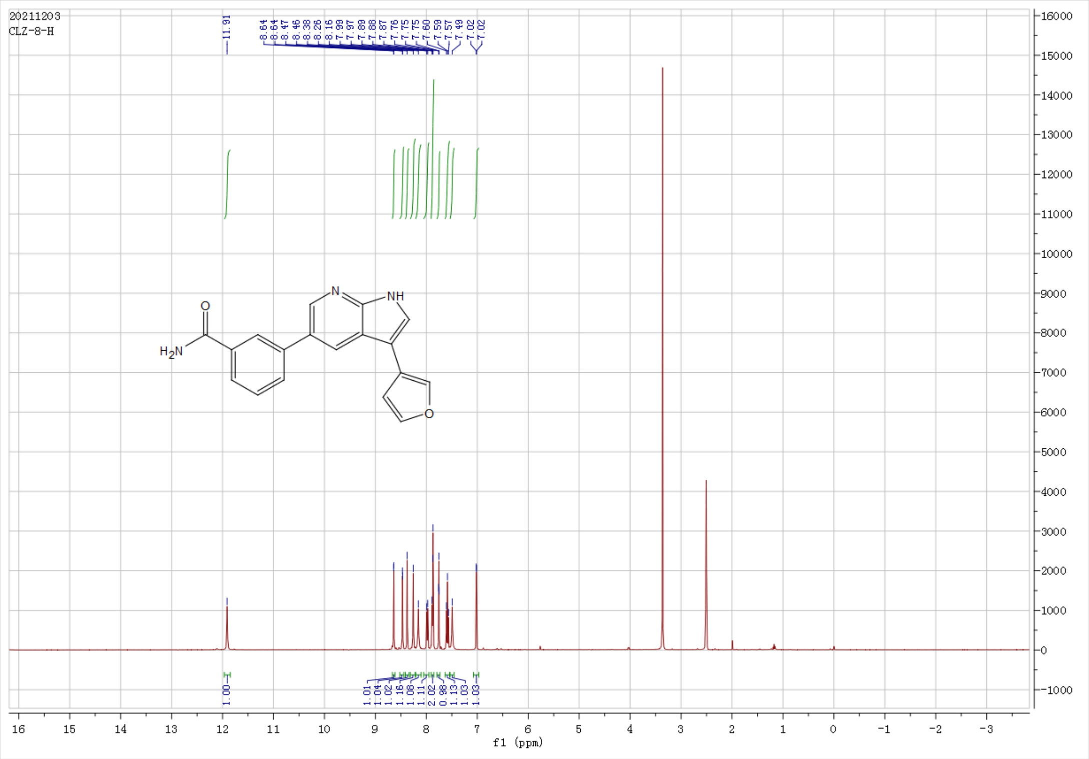

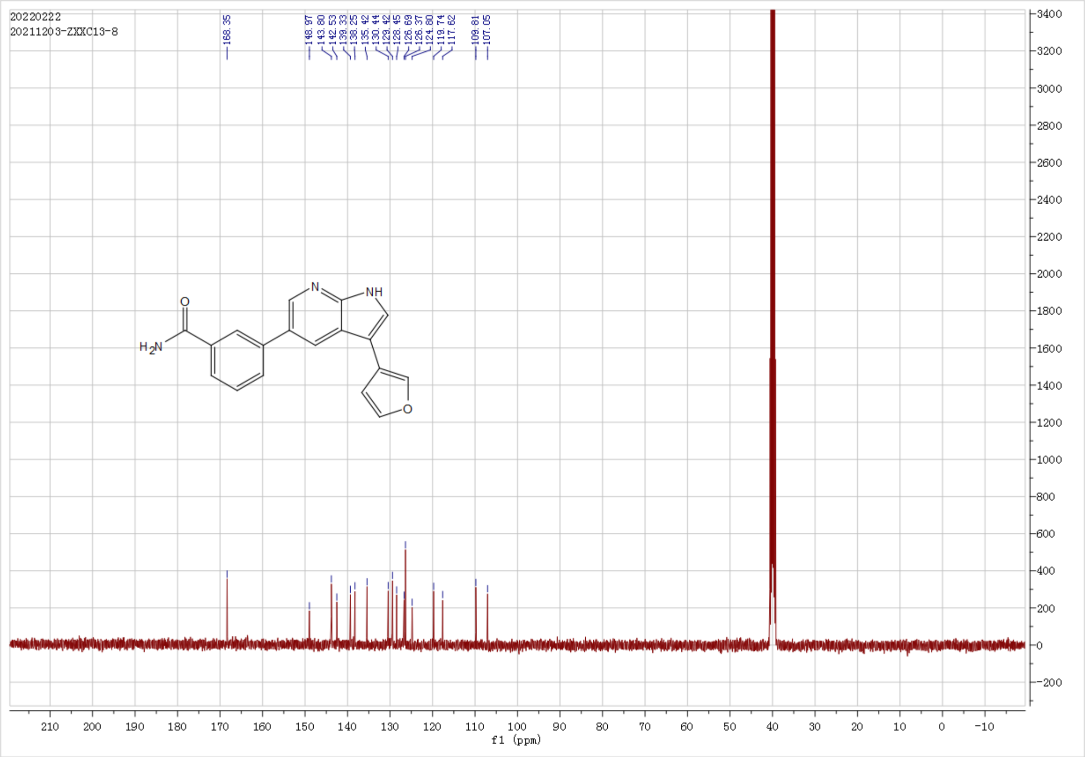

**Sample Name**	ZXX-2022-0301-127	**Position**	Vial 27	**Instrument Name**	Instrument 1	**User Name**	
**Inj Vol**	2	**InjPosition**		**SampleType**	Sample	**IRM Calibration Status**	All Ions Missed
**Data Filename**	ZXX-2022-0301-127.d	**ACQ Method**	2020LXSpo.m	**Comment**		**Acquired Time**	3/1/2022 2:34:56 PM

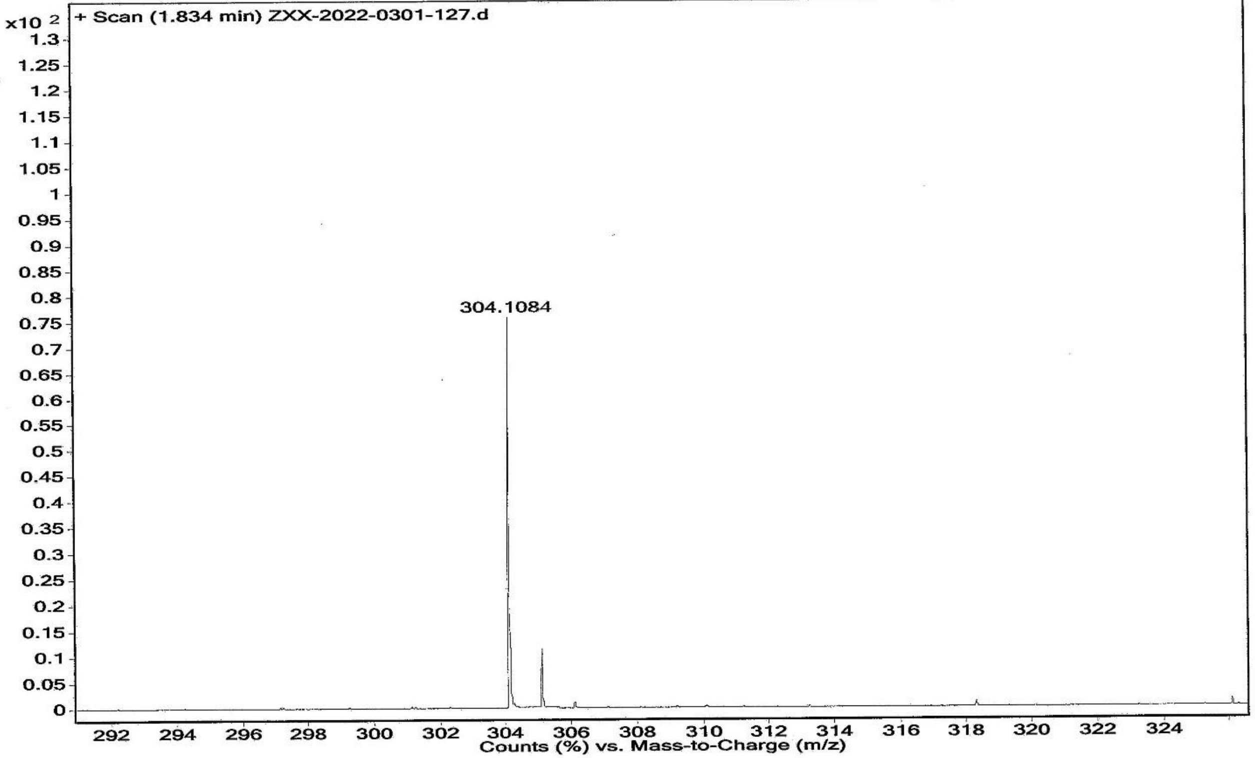

0926-1

3-(3-(thiophen-3-yl)-1H-pyrrolo[2,3-b]pyridin-5-yl)benzamide (**13**)

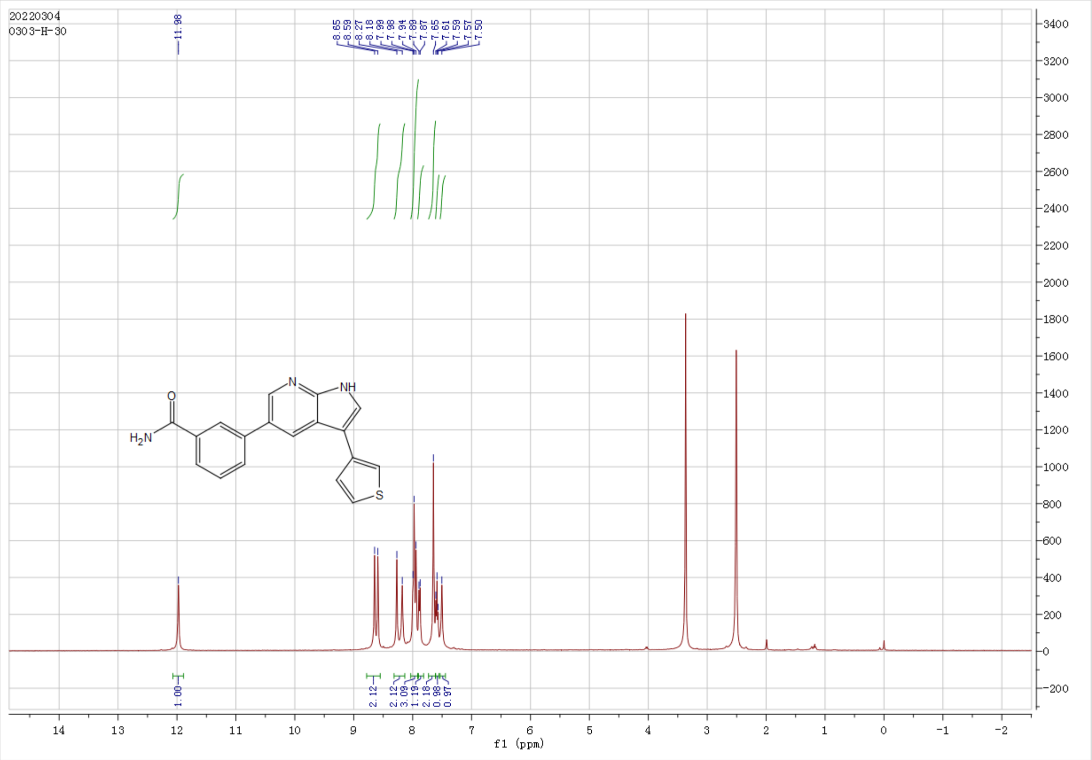

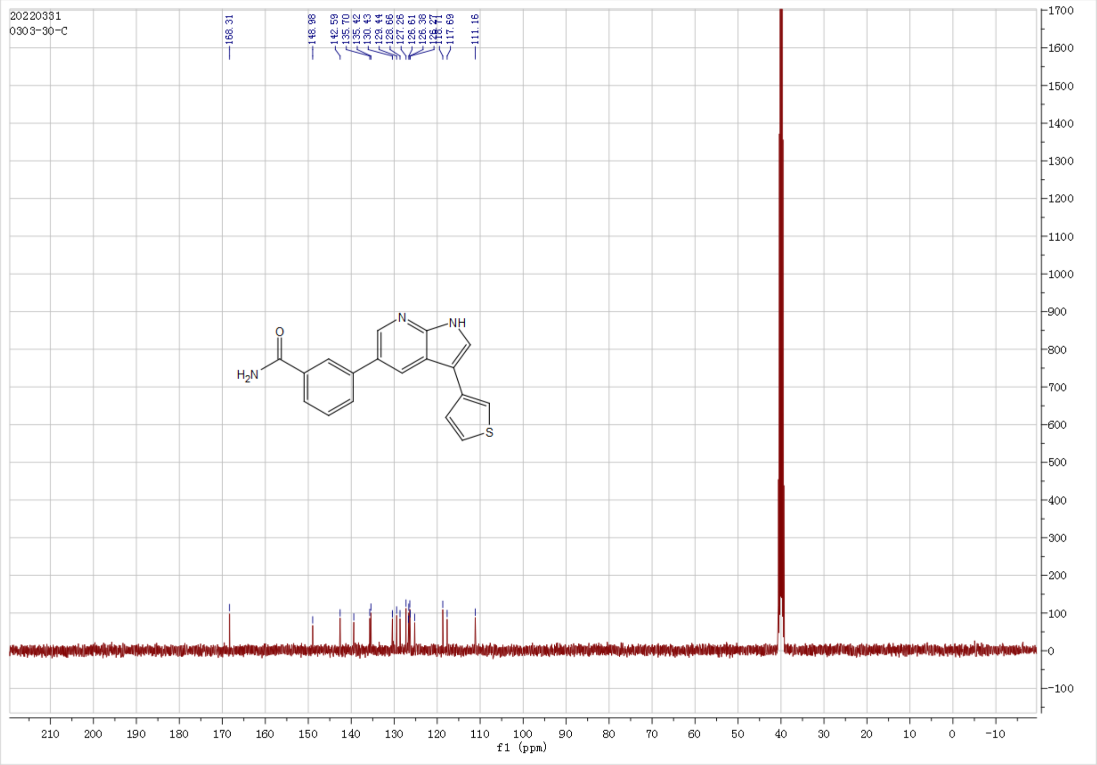

**Sample Name**	ZXX-2022-0301-138	**Position**	Vial 38	**Instrument Name**	Instrument 1	**User Name**	
**Inj Vol**	2	**InjPosition**		**SampleType**	Sample	**IRM Calibration Status**	All Ions Missed
**Data Filename**	ZXX-2022-0301-138.d	**ACQ Method**	2020LXSpo.m	**Comment**		**Acquired Time**	3/1/2022 3:49:40 PM

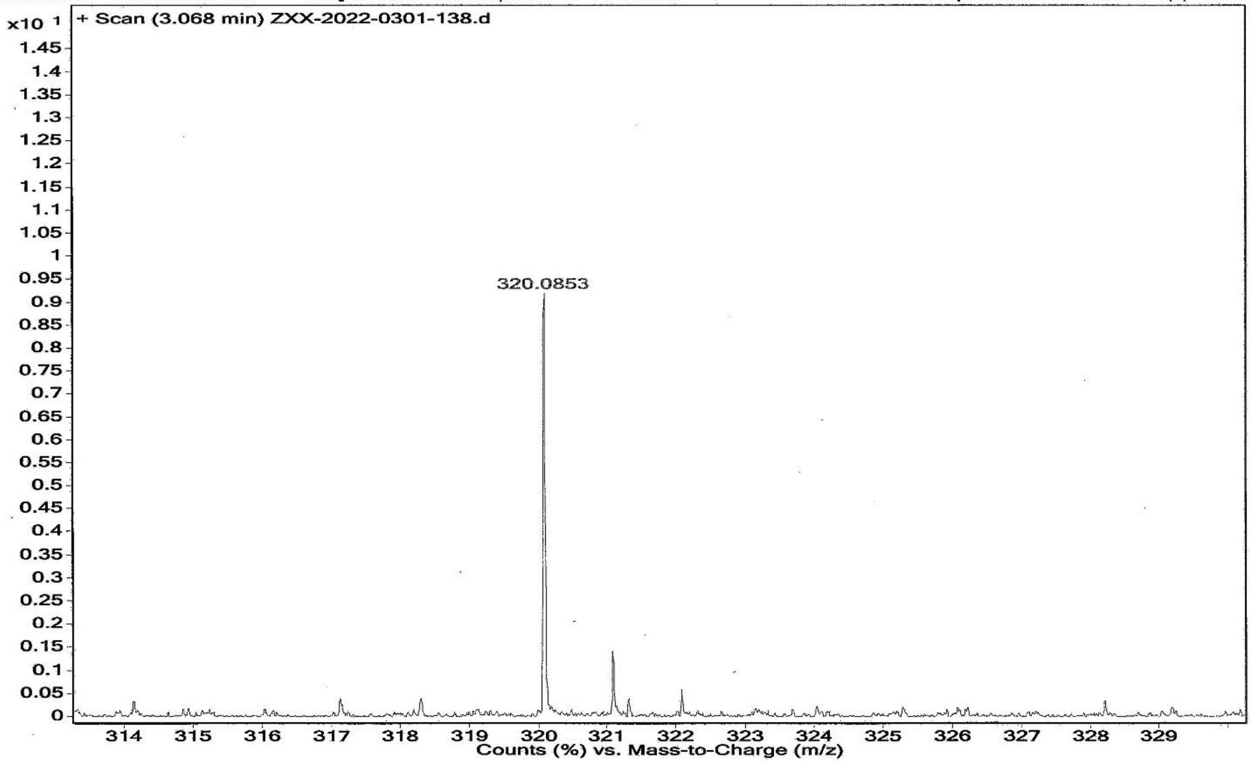

bls 7

3-(3-(thiophen-2-yl)-1H-pyrrolo[2,3-b]pyridin-5-yl)benzamide (**14**)

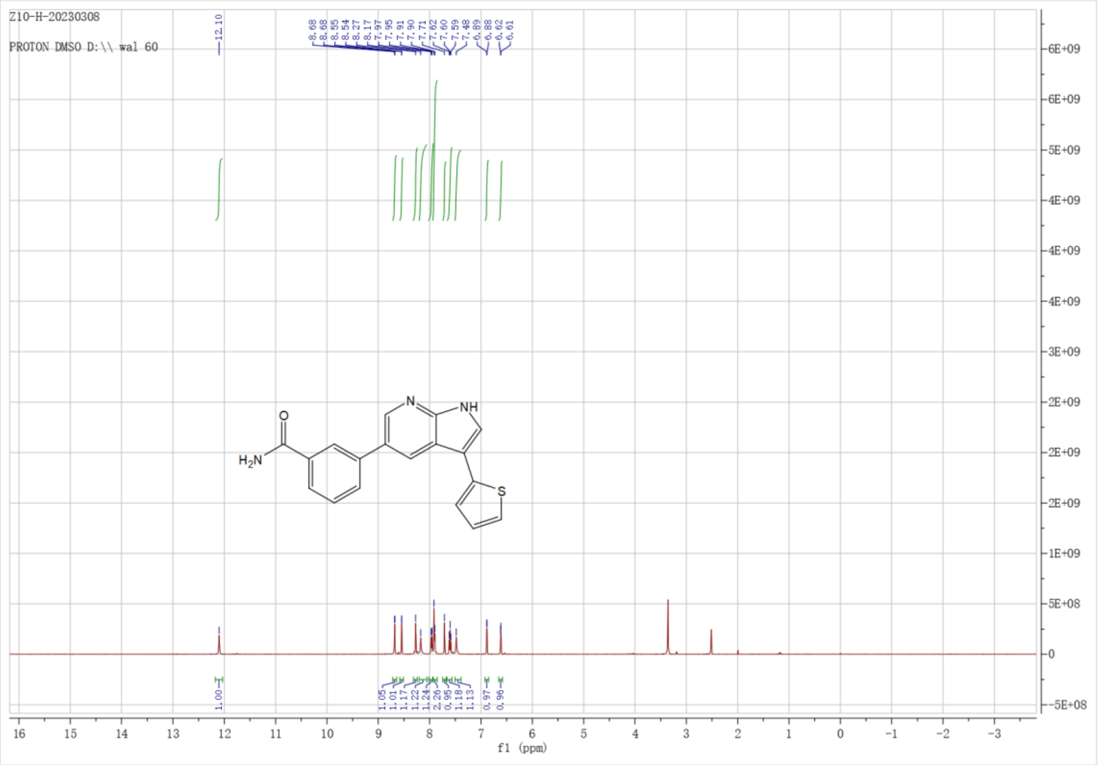

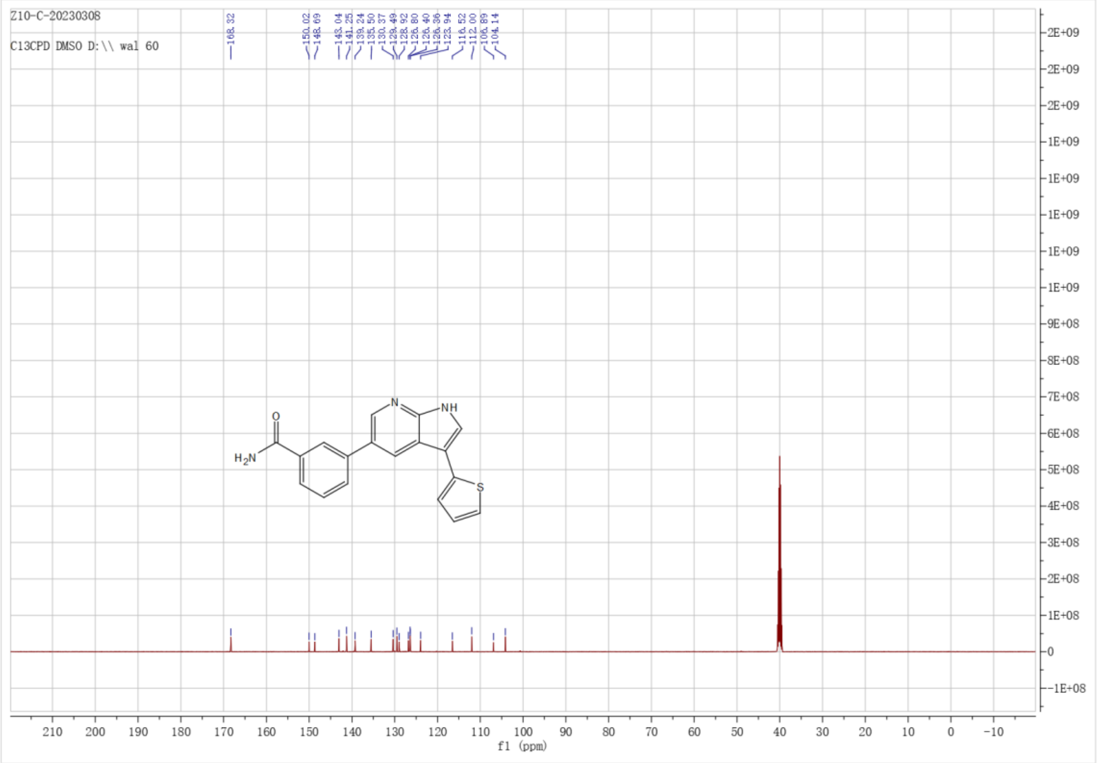

Sample Name	Position	Instrument Name	User Name
Inj Vol	InjPosition	SampleType	IRM Calibration Status
Data Filename	ACQ Method	Comment	Acquired Time

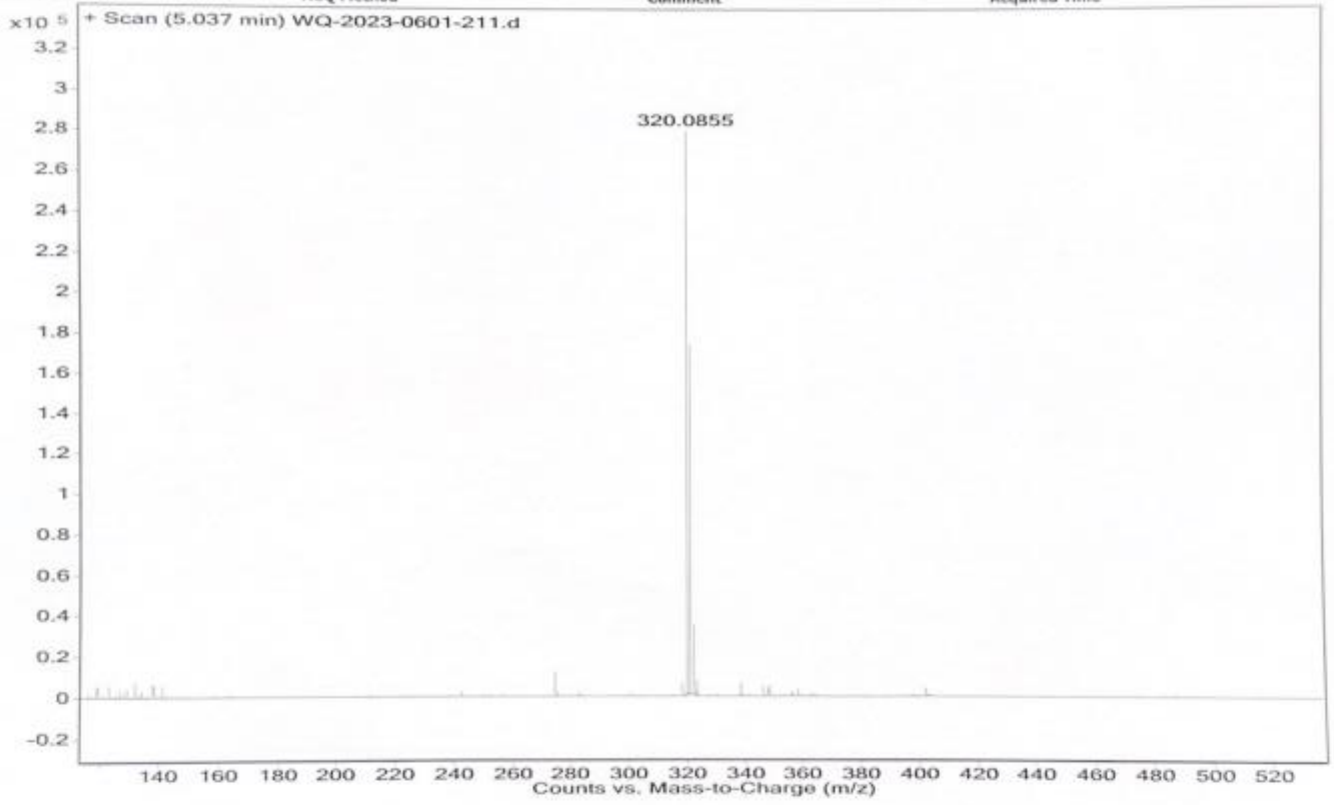

3-(3-(1H-pyrazol-4-yl)-1H-pyrrolo[2,3-b]pyridin-5-yl)benzamide (**15**)

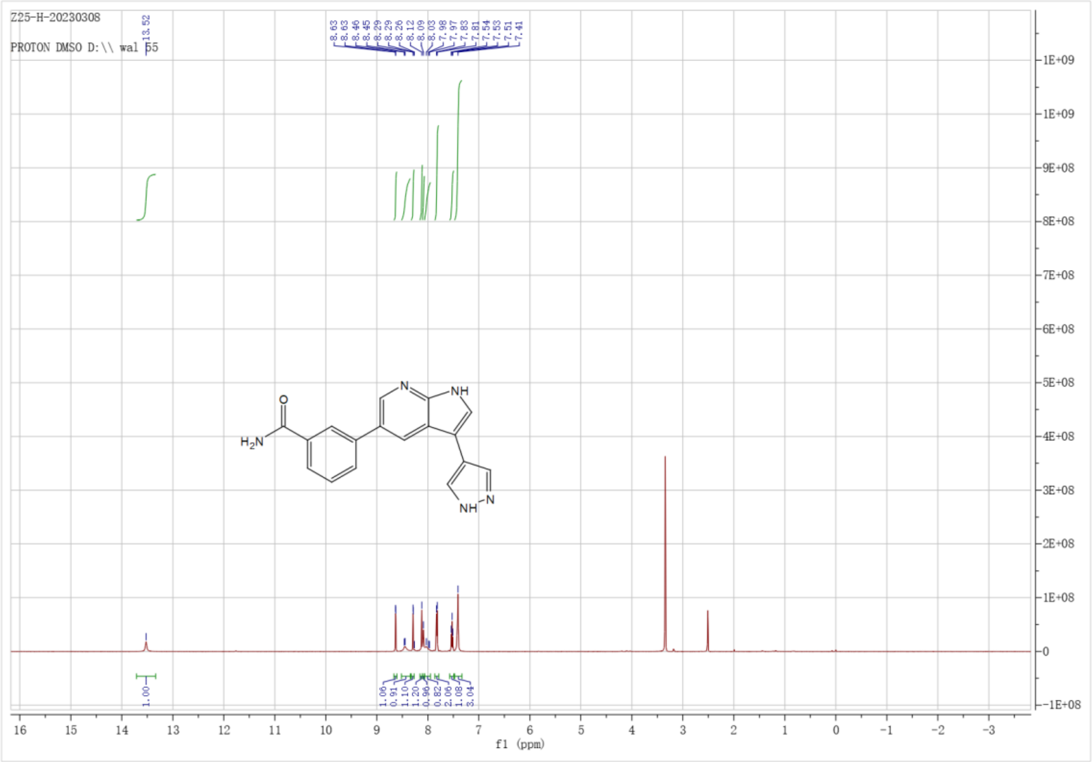

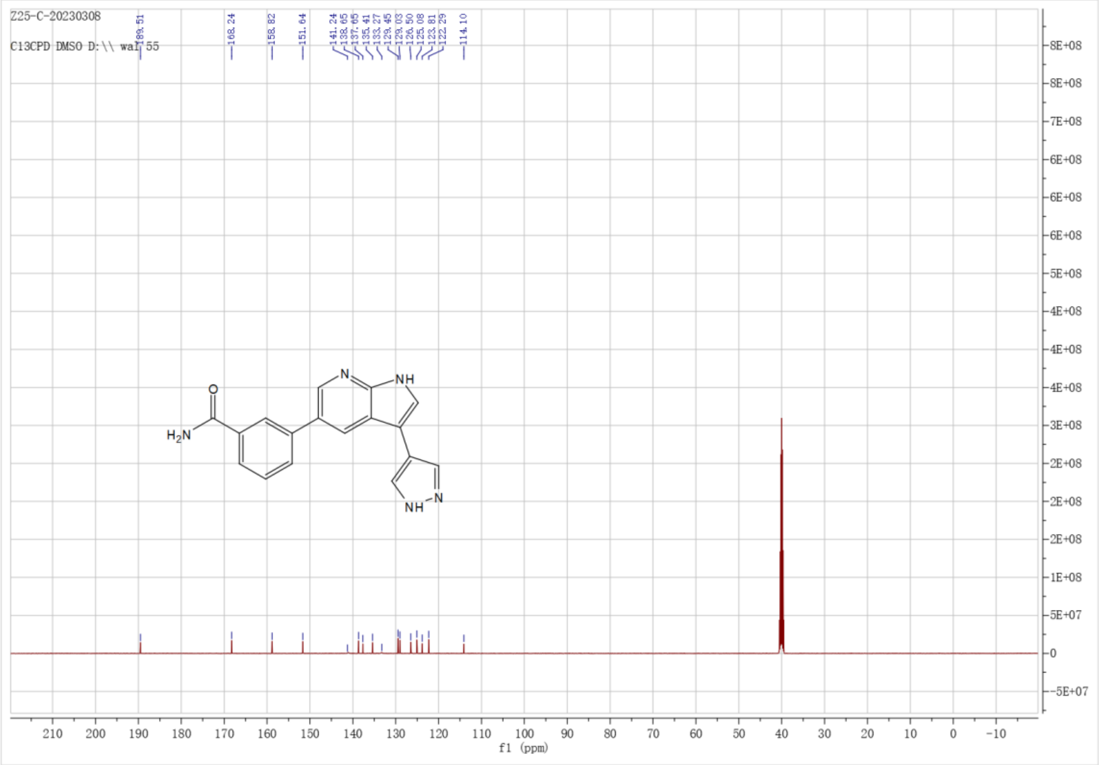

Sample Name	Position	Instrument Name	User Name
Inj Vol	InjPosition	SampleType	IRM Calibration Status
Data Filename	ACQ Method	Comment	Acquired Time

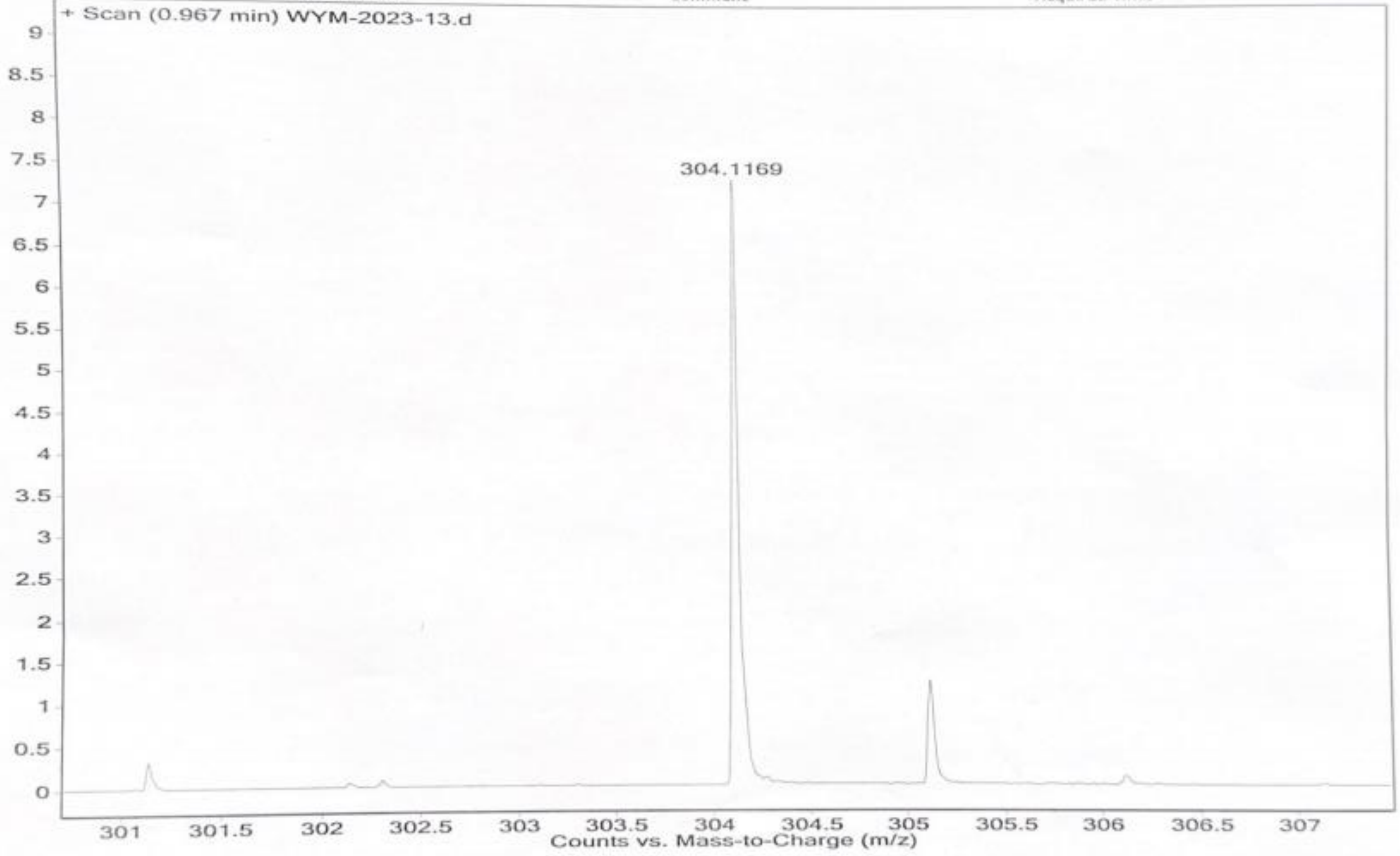

3-(3-(1-methyl-1H-pyrazol-4-yl)-1H-pyrrolo[2,3-b]pyridin-5-yl)benzamide (**16**)

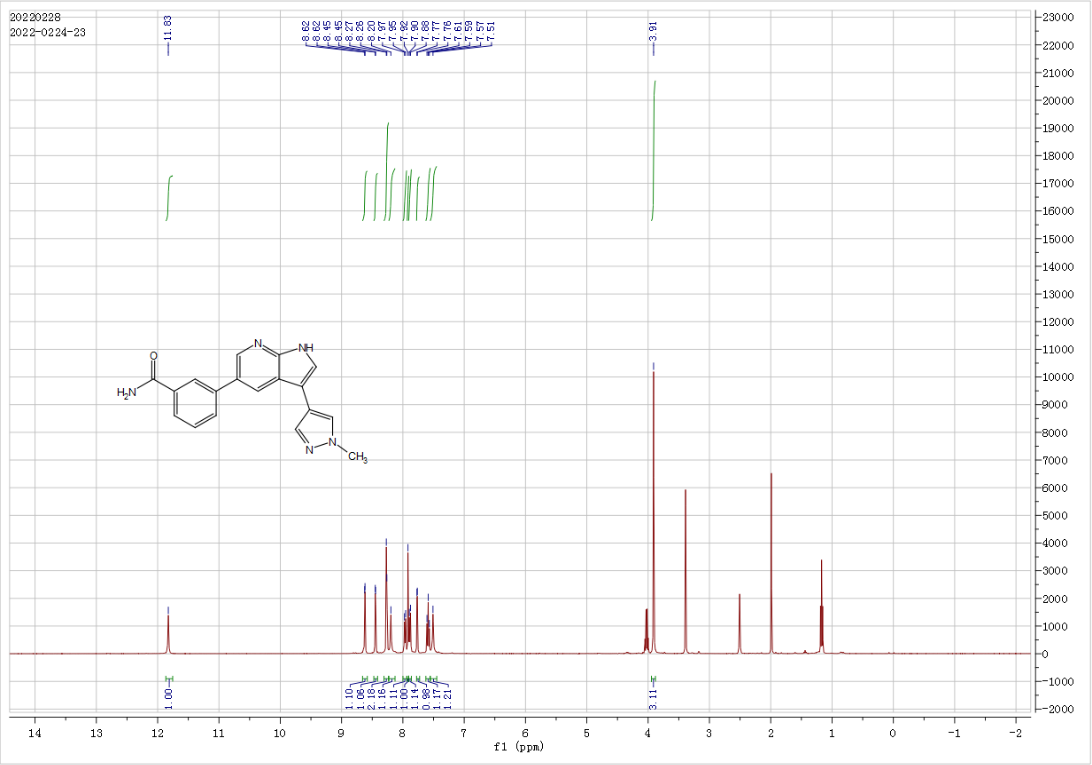

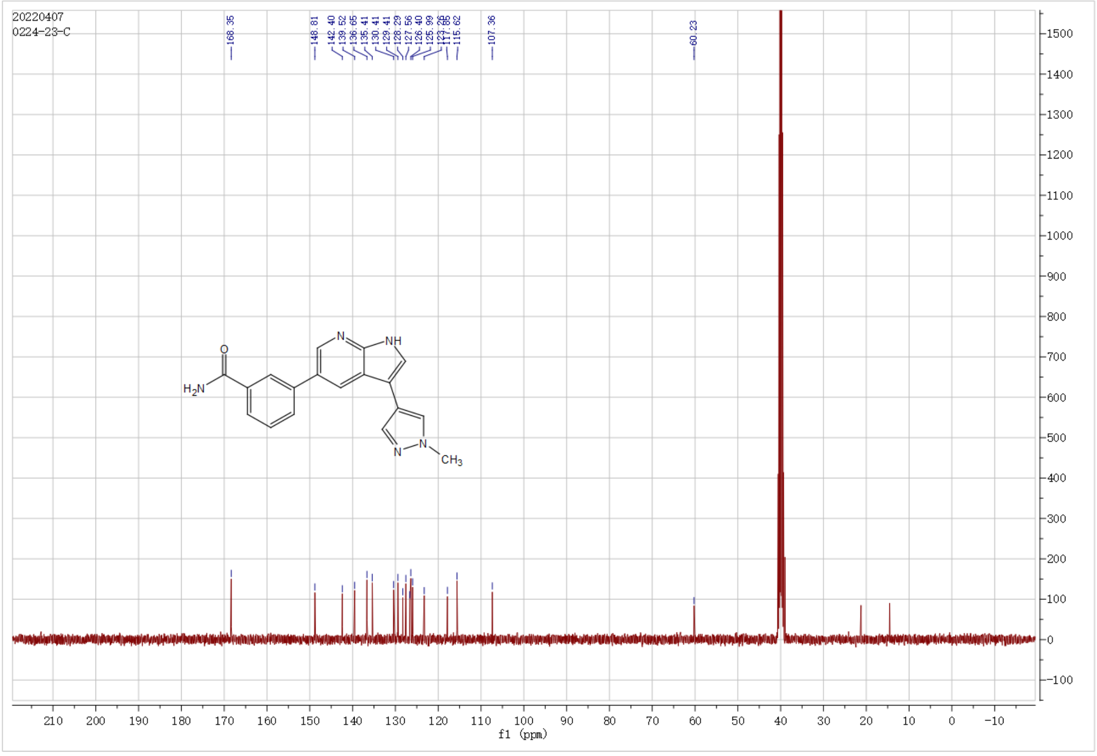

**Sample Name**	ZXX-2022-0224-123	**Position**	Vial 23	**Instrument Name**	Instrument 1	**User Name**	
**Inj Vol**	2	**InjPosition**		**SampleType**	Sample	**IRM Calibration Status**	All Ions Missed
**Data Filename**	ZXX-2022-0224-123.d	**ACQ Method**	2020LXSpo.m	**Comment**		**Acquired Time**	2/25/2022 12:45:19 AM

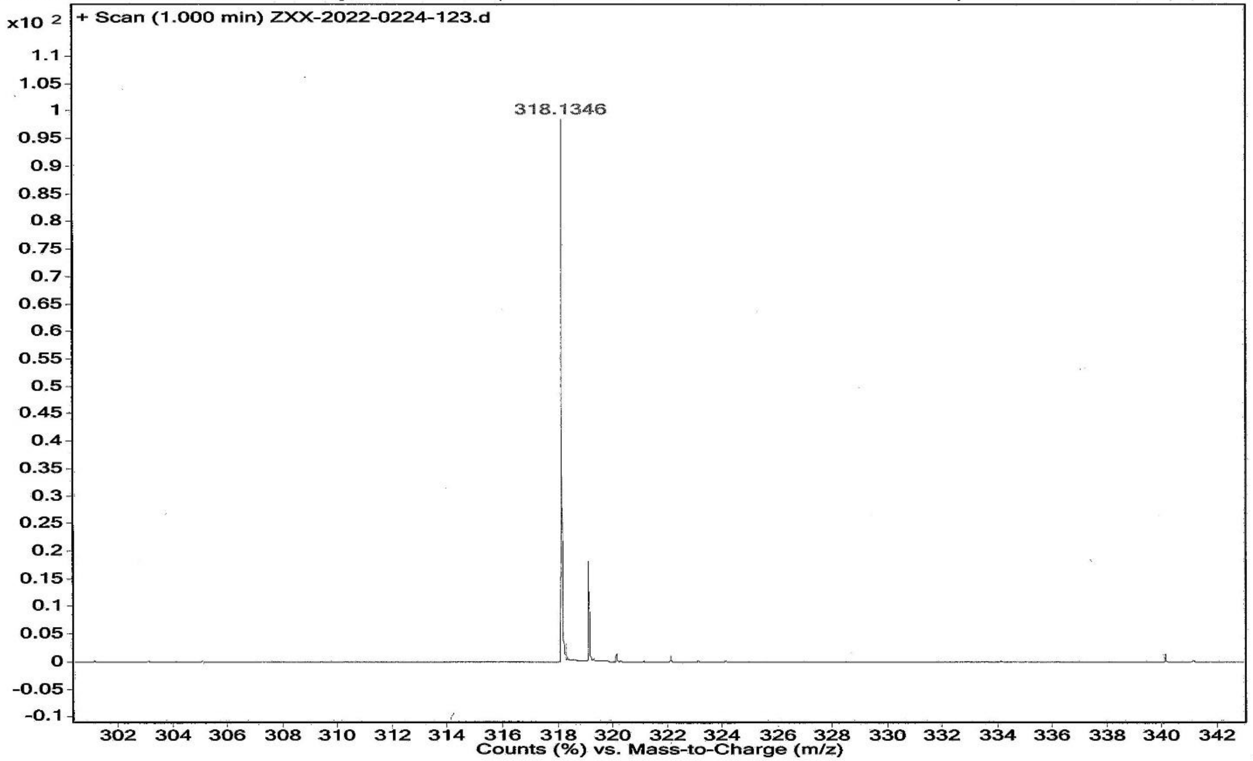

092-1

3-(3-(1-(1-ethoxyethyl)-1H-pyrazol-4-yl)-1H-pyrrolo[2,3-b]pyridin-5-yl)benzamide (**17**)

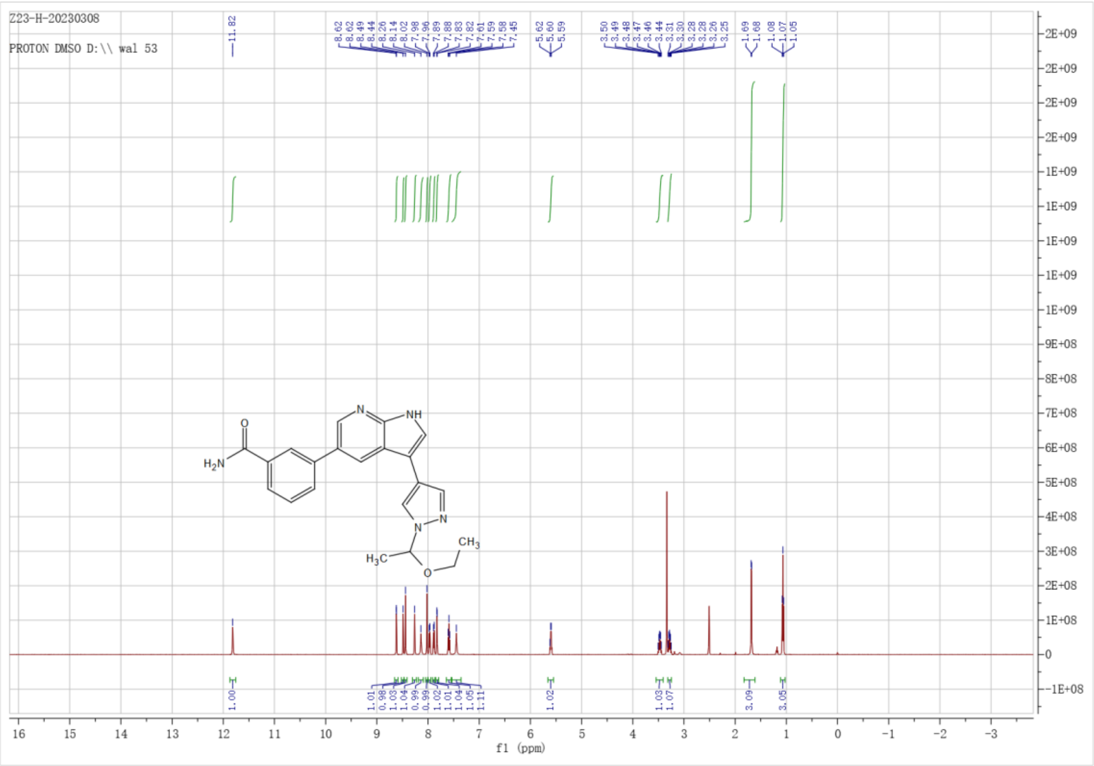

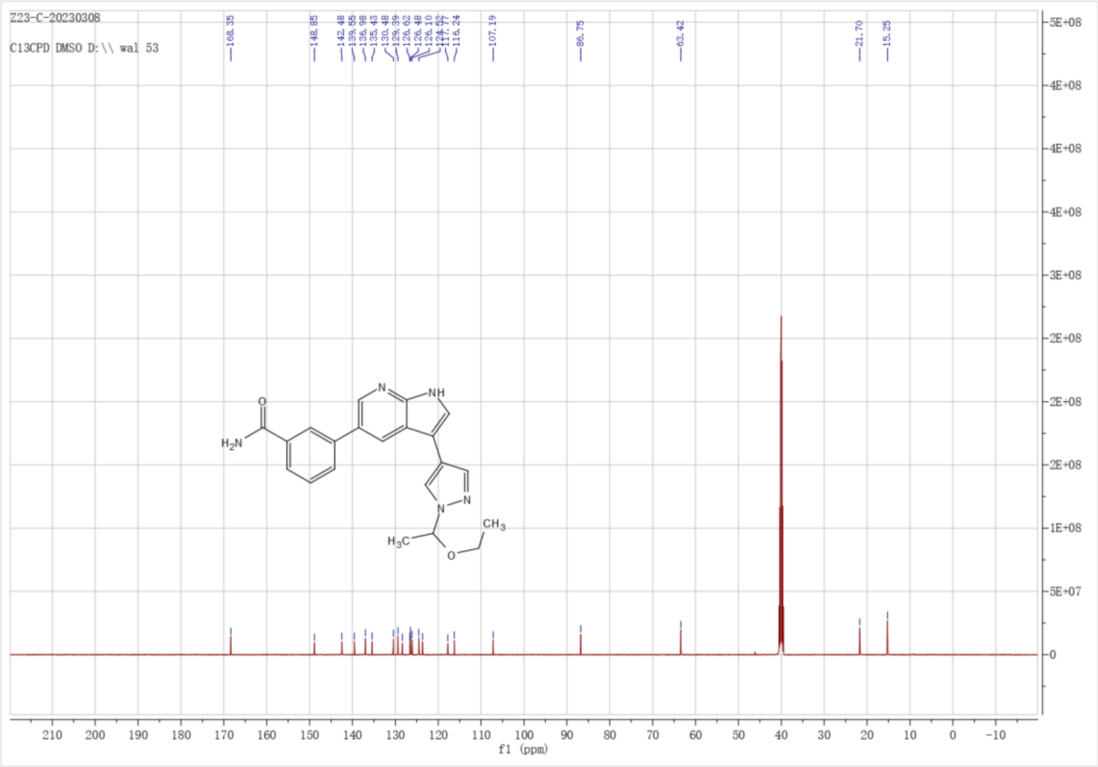

Sample Name	Position	Instrument Name	User Name
Inj Vol	InjPosition	SampleType	IRM Calibration Status
Data Filename	ACQ Method	Comment	Acquired Time

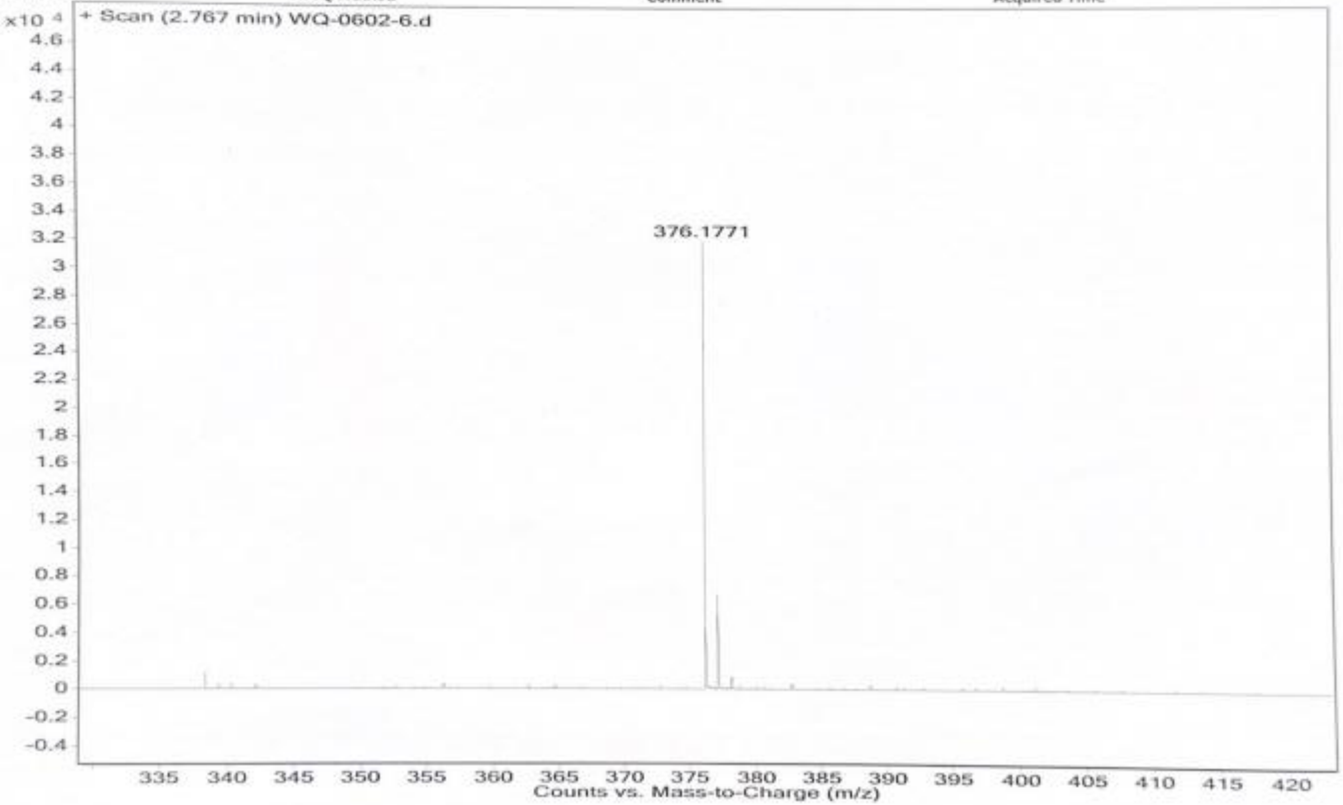

3-(3-(1-(difluoromethyl)-1H-pyrazol-4-yl)-1H-pyrrolo[2,3-b]pyridin-5-yl)benzamide (**18**)

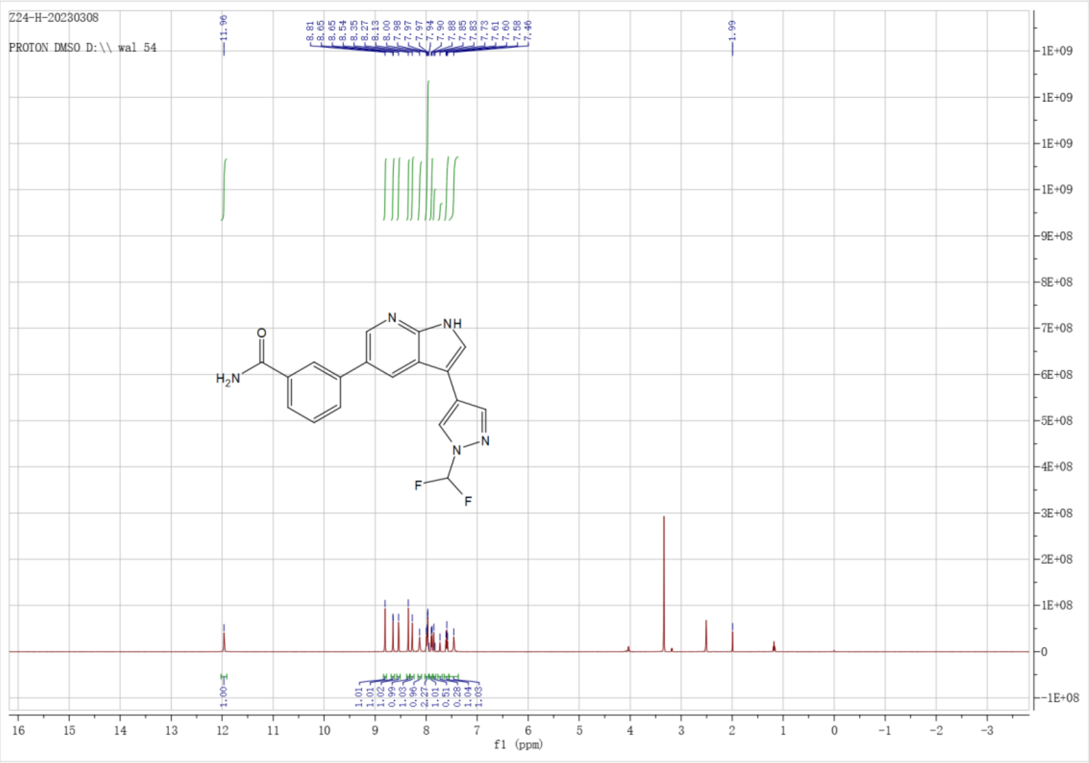

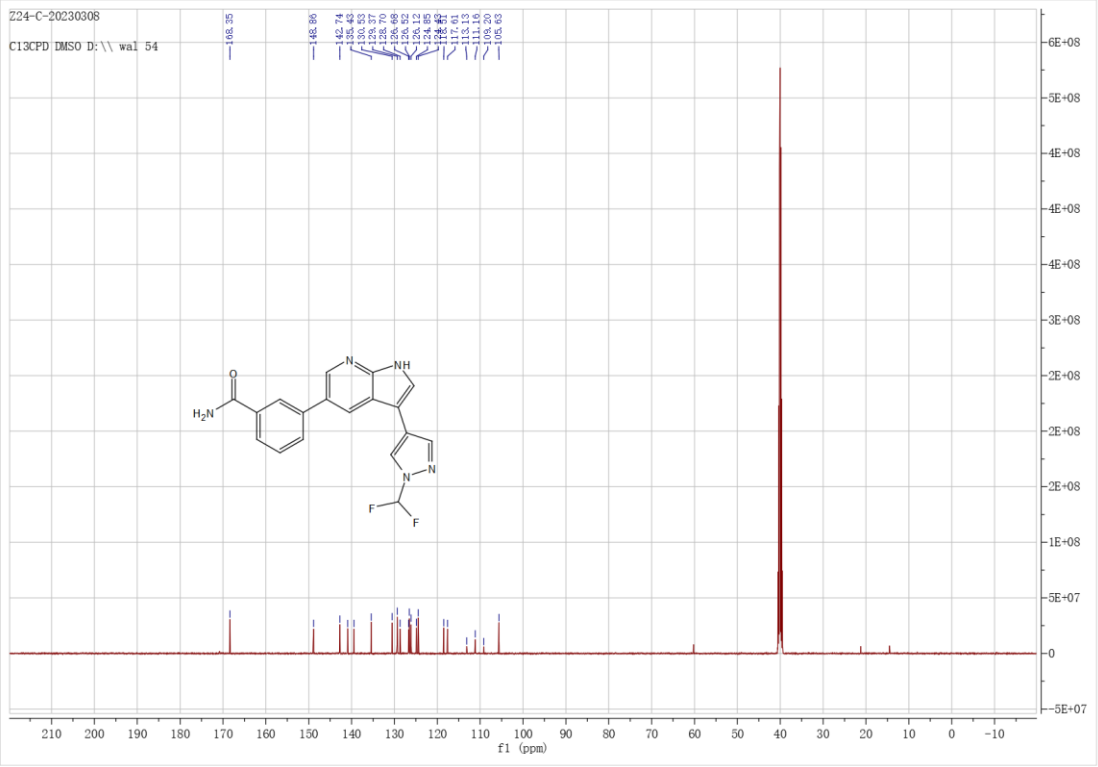

Sample Name	Position	Instrument Name	User Name
Inj Vol	InjPosition	SampleType	IRM Calibration Status
Data Filename	ACQ Method	Comment	Acquired Time

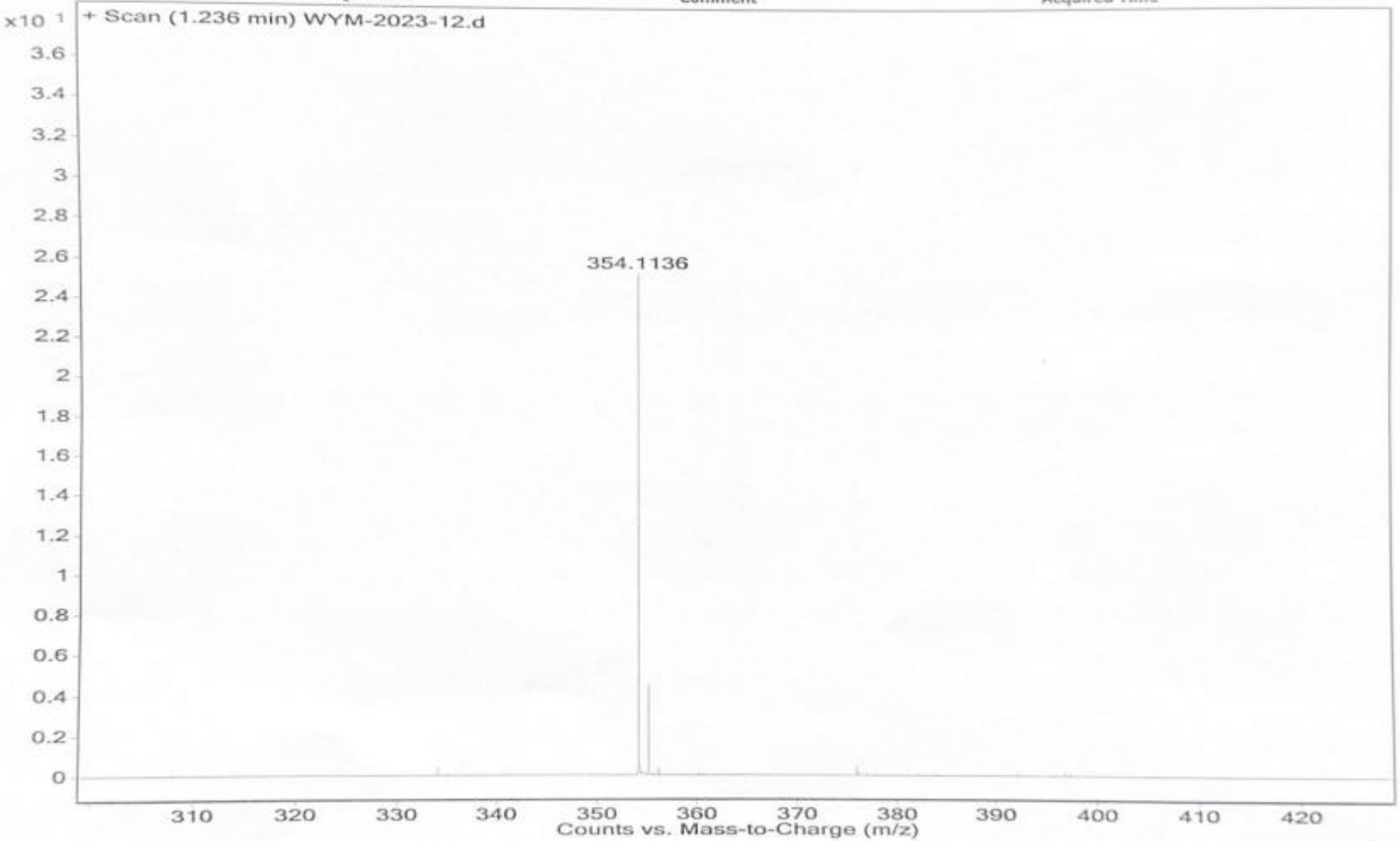

3-(3-phenyl-1H-pyrrolo[2,3-b]pyridin-5-yl)benzamide (19)

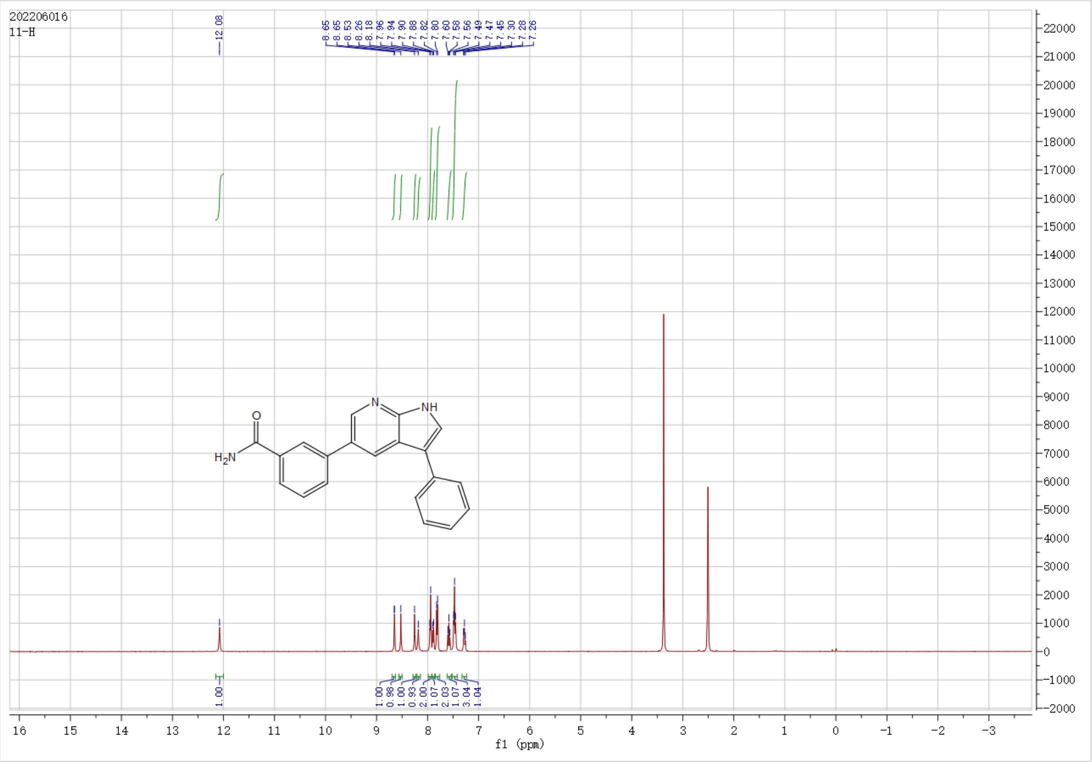

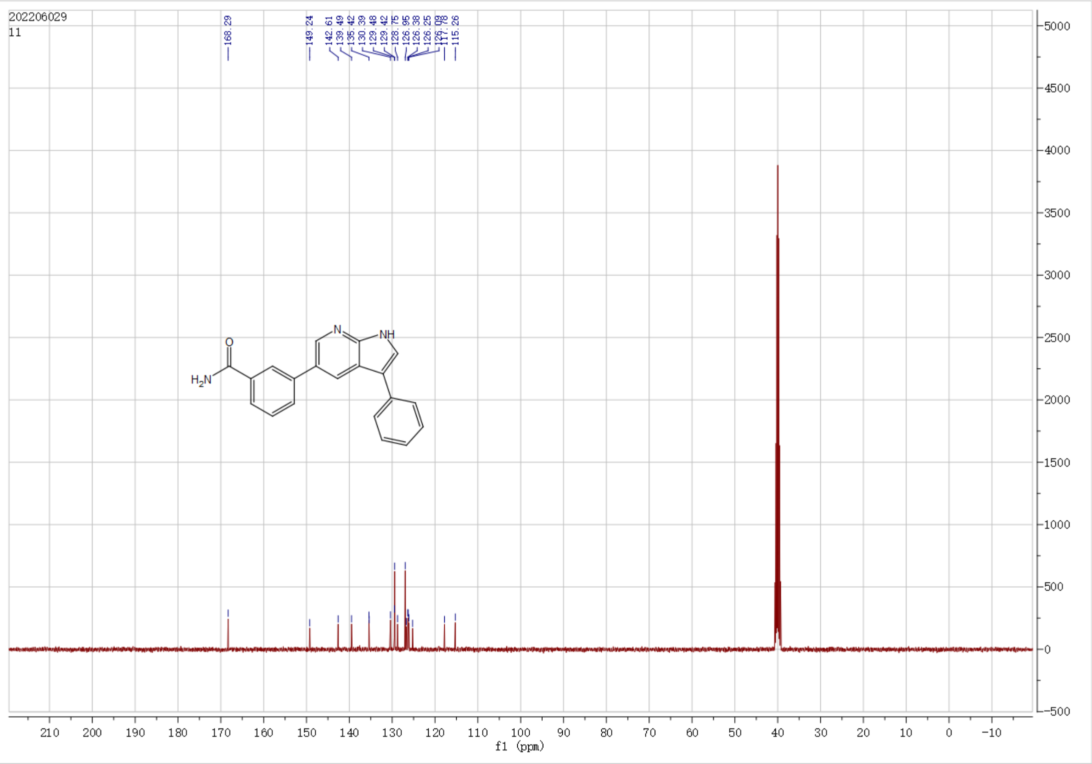

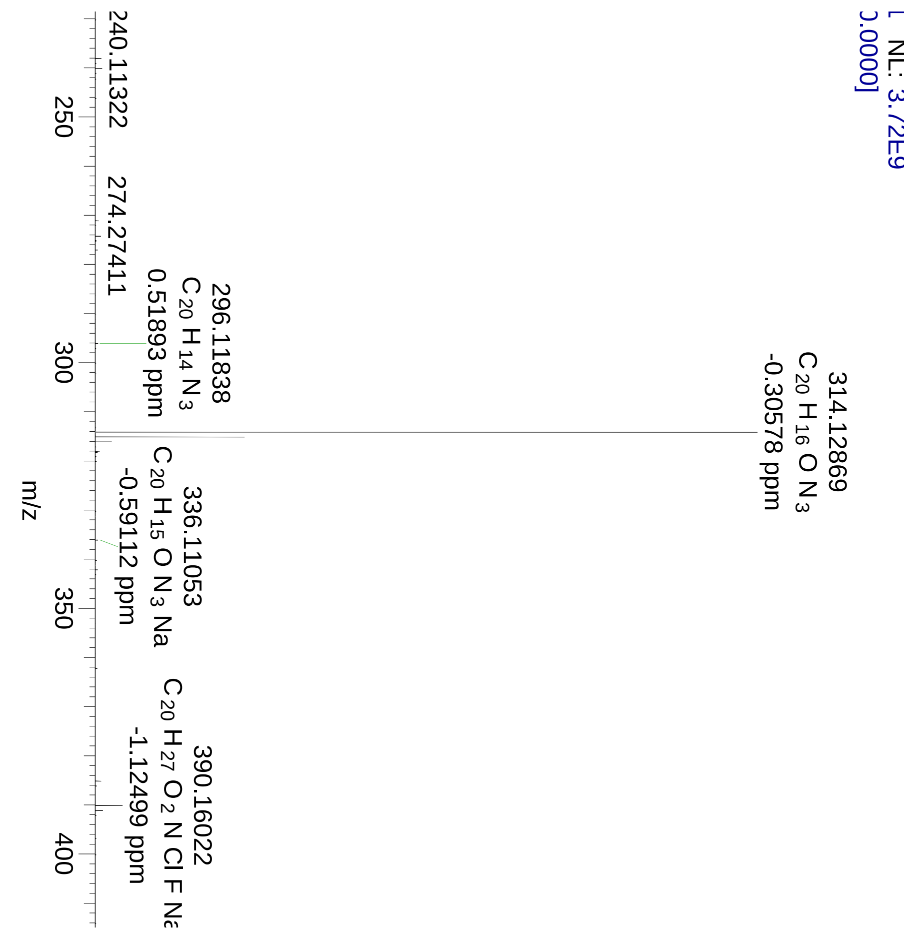

3-(3-(2-fluorophenyl)-1H-pyrrolo[2,3-b]pyridin-5-yl)benzamide (**20**)

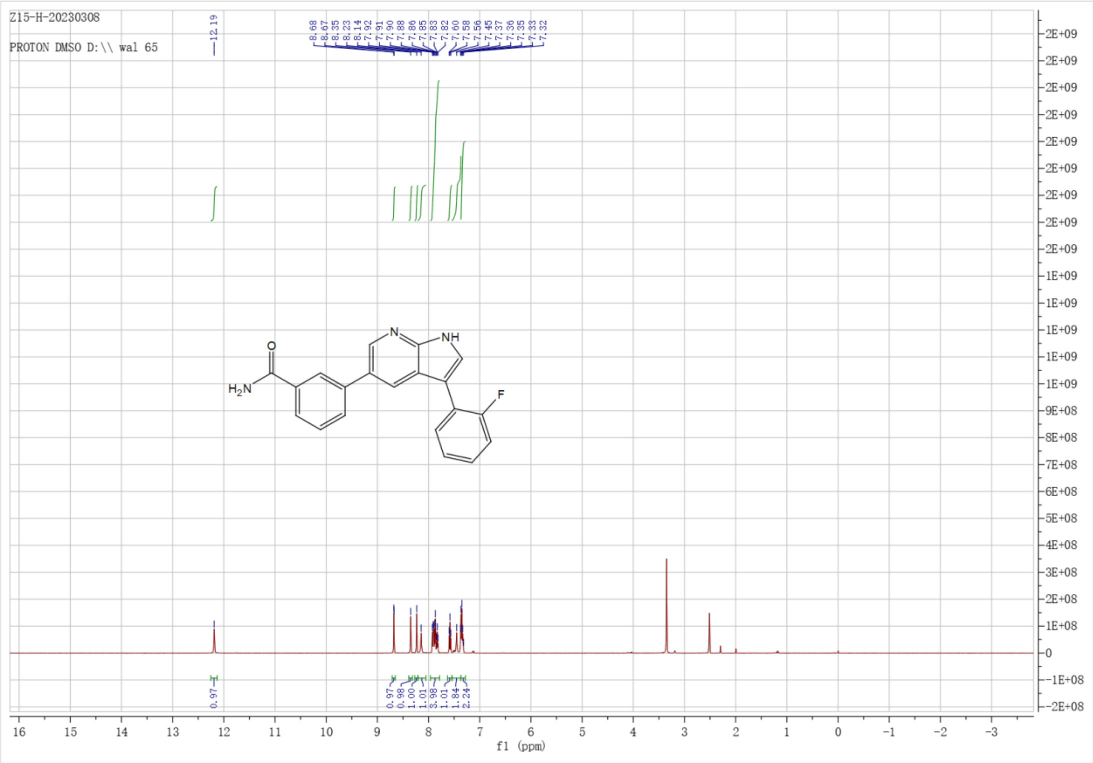

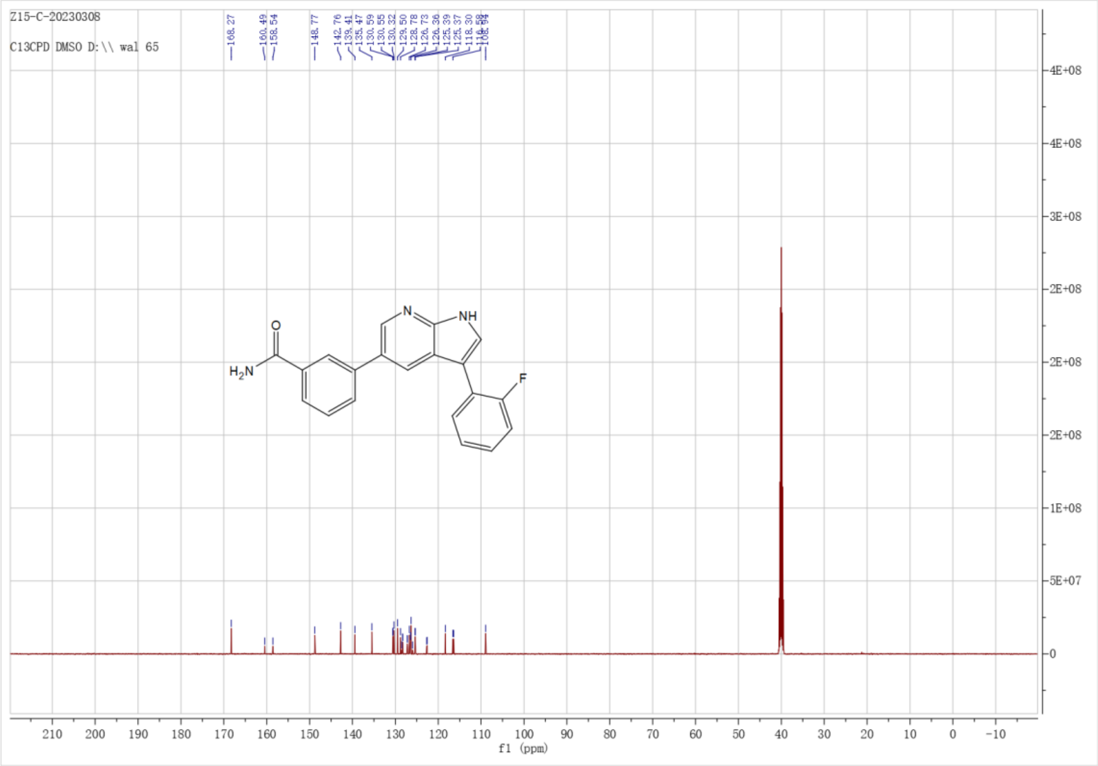
Z15-C-20230308
C13CPD DMSO D:\\ wal 65
168.27
160.49
158.54
148.77
142.76
139.41
135.47
130.59
130.55
130.32
129.50
128.78
126.73
126.36
125.39
125.37
118.30
108.94
O
H2N
N
NH
F
4E+08
4E+08
3E+08
2E+08
2E+08
2E+08
1E+08
5E+07
0
210 200 190 180 170 160 150 140 130 120 110 100 90 80 70 60 50 40 30 20 10 0 -10
f1 (ppm)

Sample Name	Position	Instrument Name	User Name
Inj Vol	InjPosition	SampleType	IRM Calibration Status
Data Filename	ACQ Method	Comment	Acquired Time

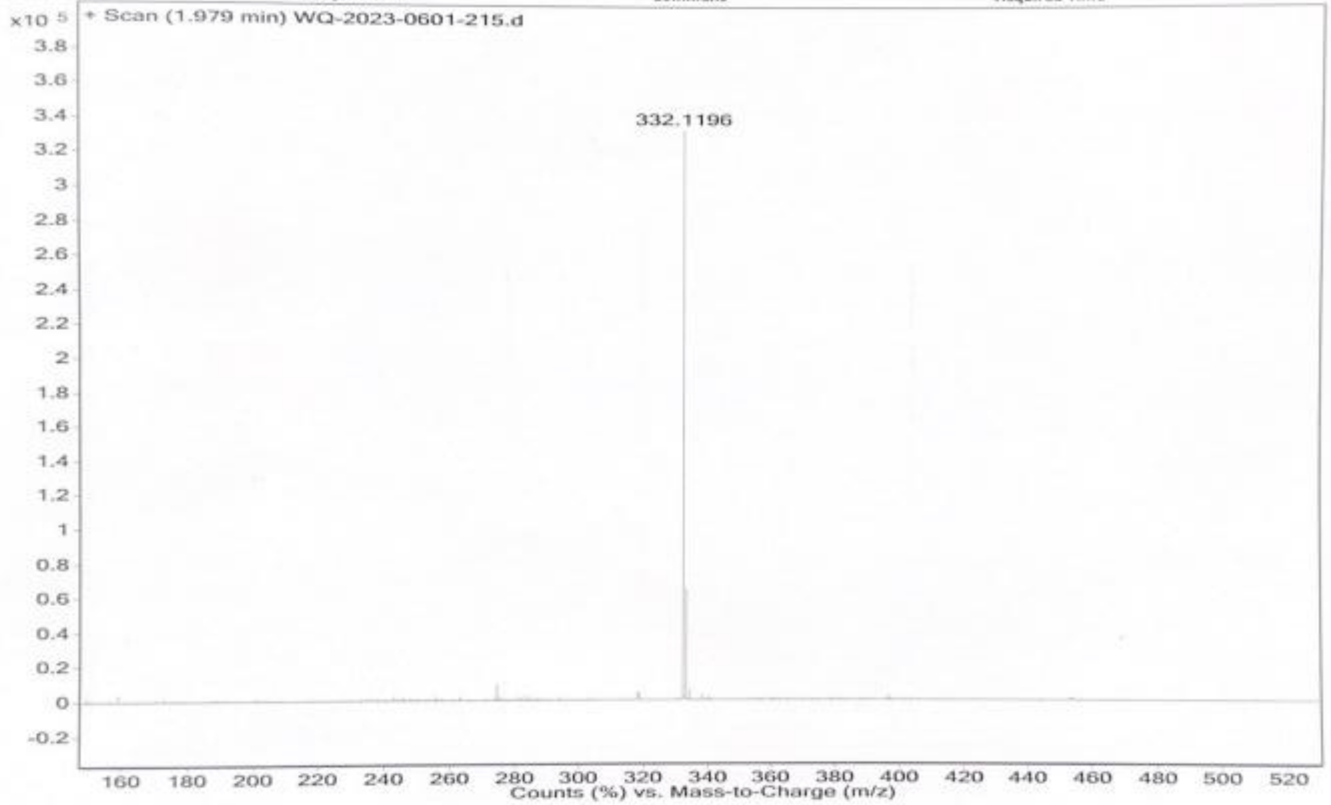

3-(3-(2-chlorophenyl)-1H-pyrrolo[2,3-b]pyridin-5-yl)benzamide （**21**）

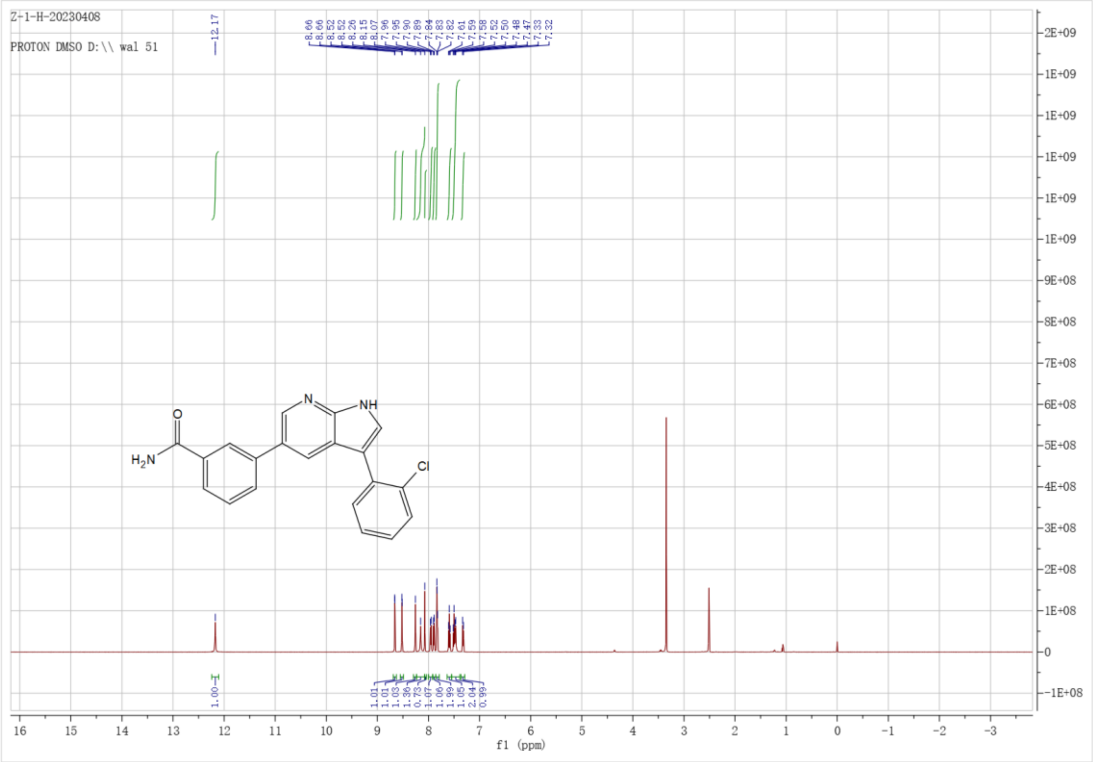

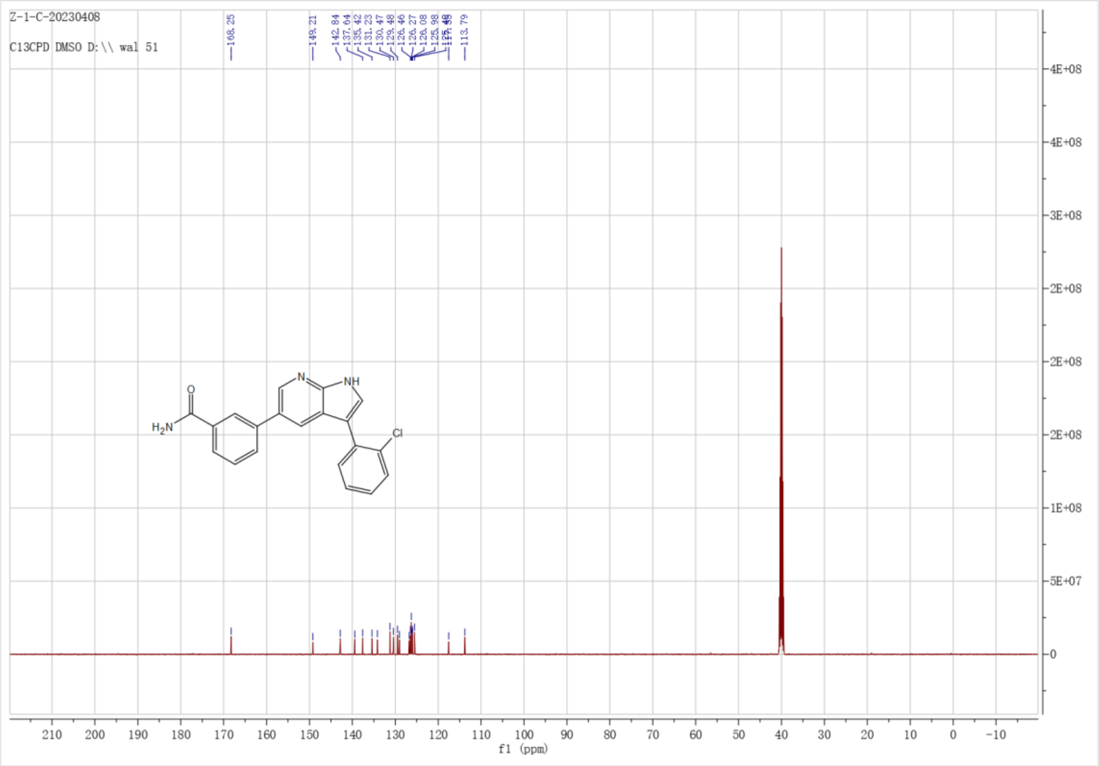

Sample Name	Position	Instrument Name	User Name
Inj Vol	InjPosition	SampleType	IRM Calibration Status
Data Filename	ACQ Method	Comment	Acquired Time

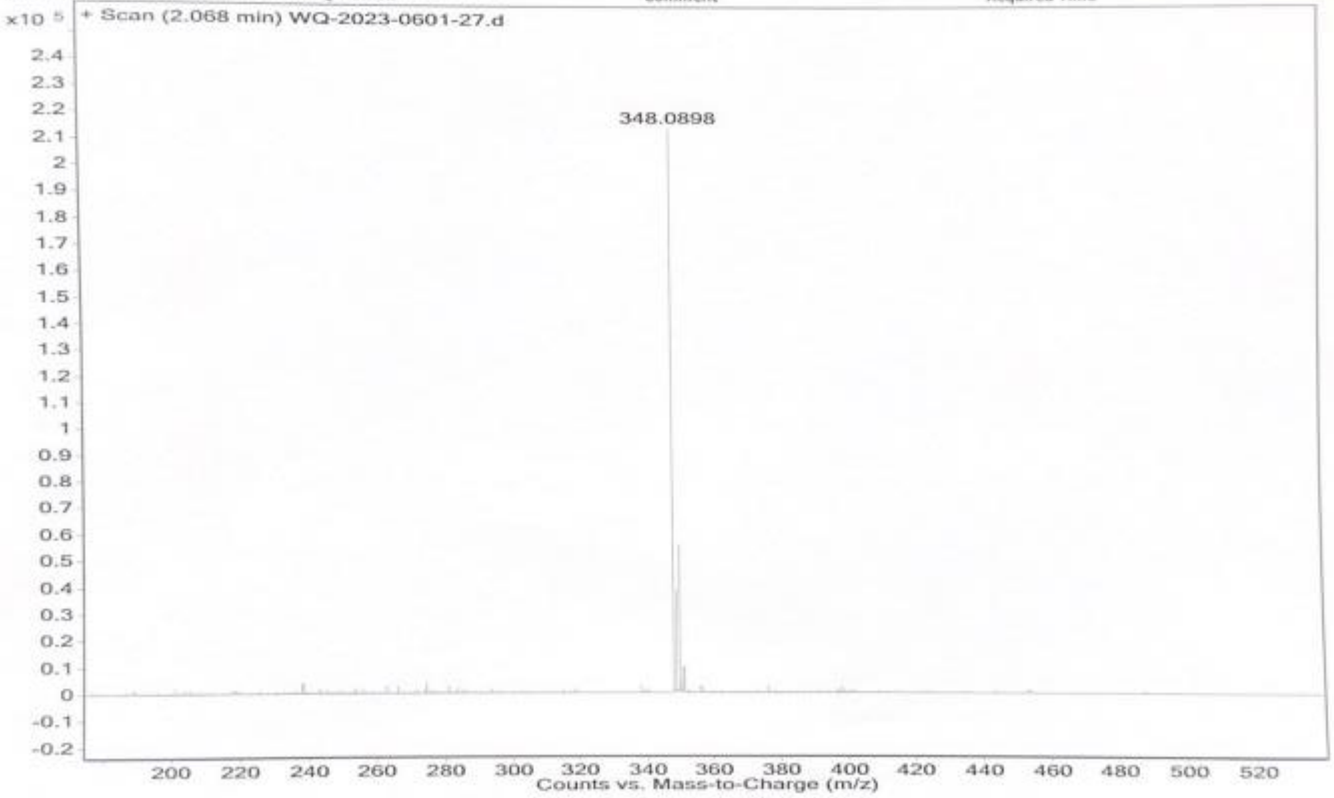

3-(3-(2-hydroxyphenyl)-1H-pyrrolo[2,3-b]pyridin-5-yl)benzamide (**22**)

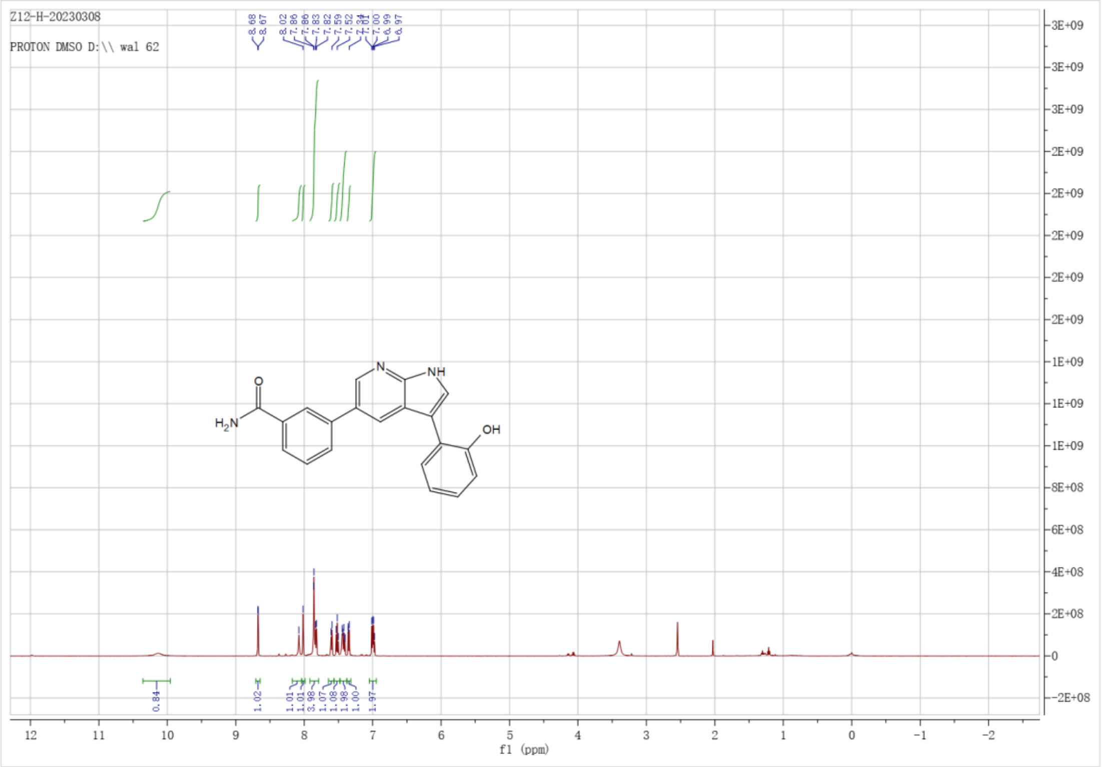

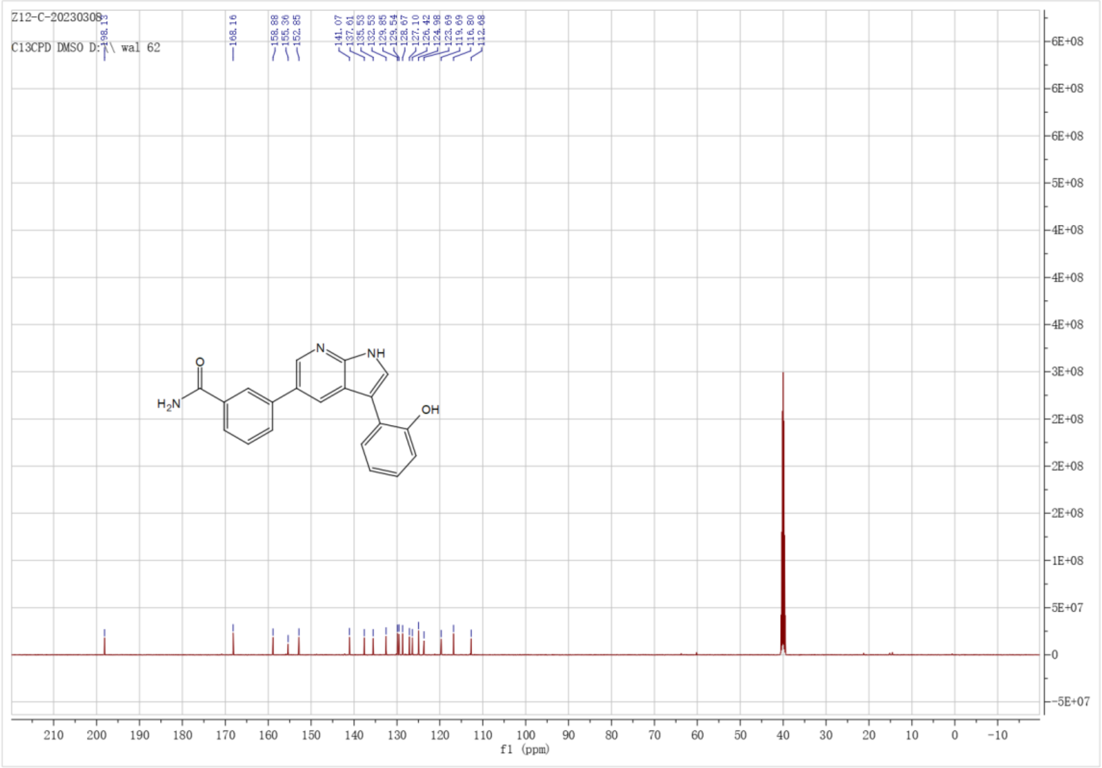

Sample Name	Position	Instrument Name	User Name
Inj Vol	InjPosition	SampleType	IRM Calibration Status
Data Filename	ACQ Method	Comment	Acquired Time

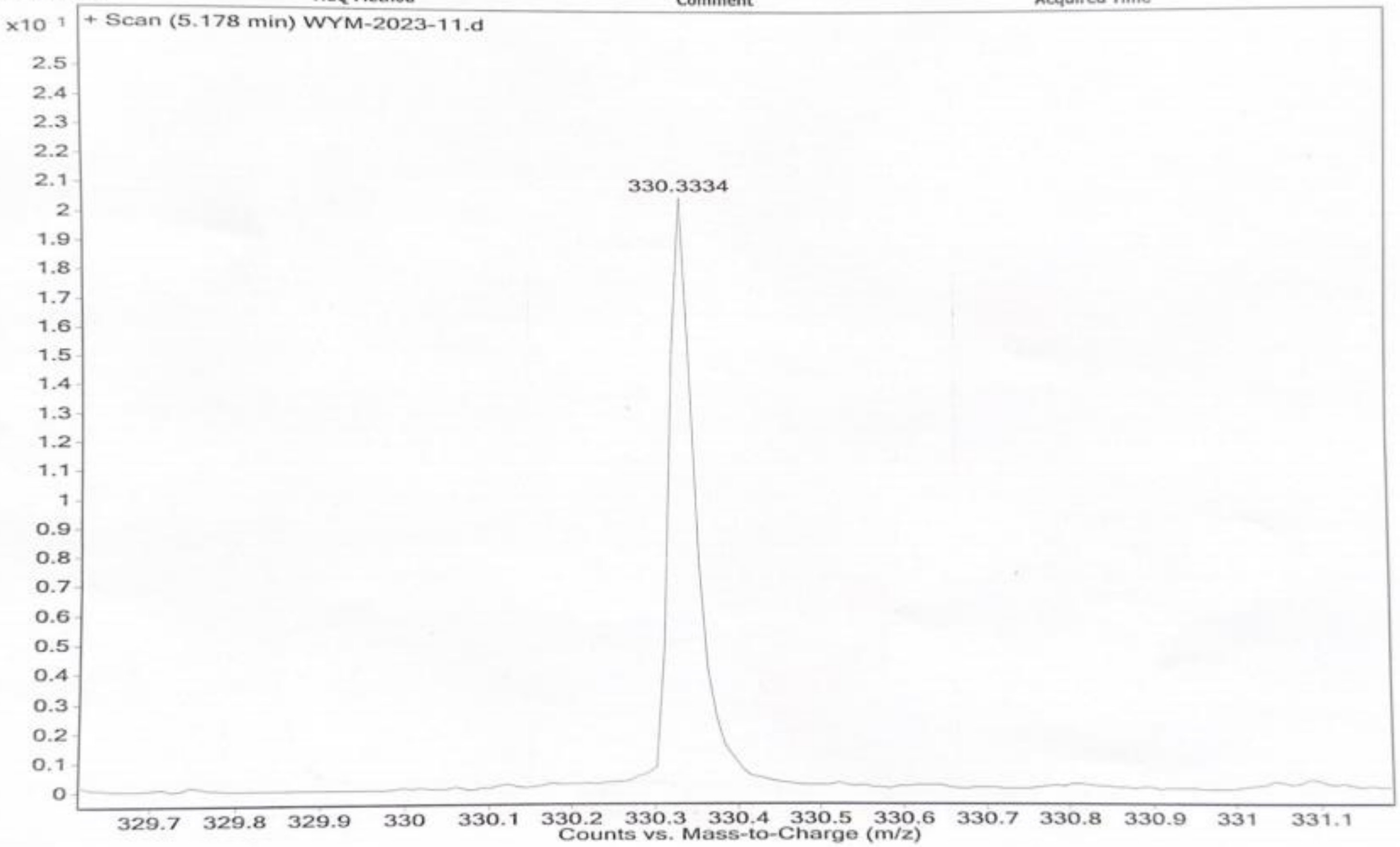

3-(3-(o-tolyl)-1H-pyrrolo[2,3-b]pyridin-5-yl)benzamide (**23**)

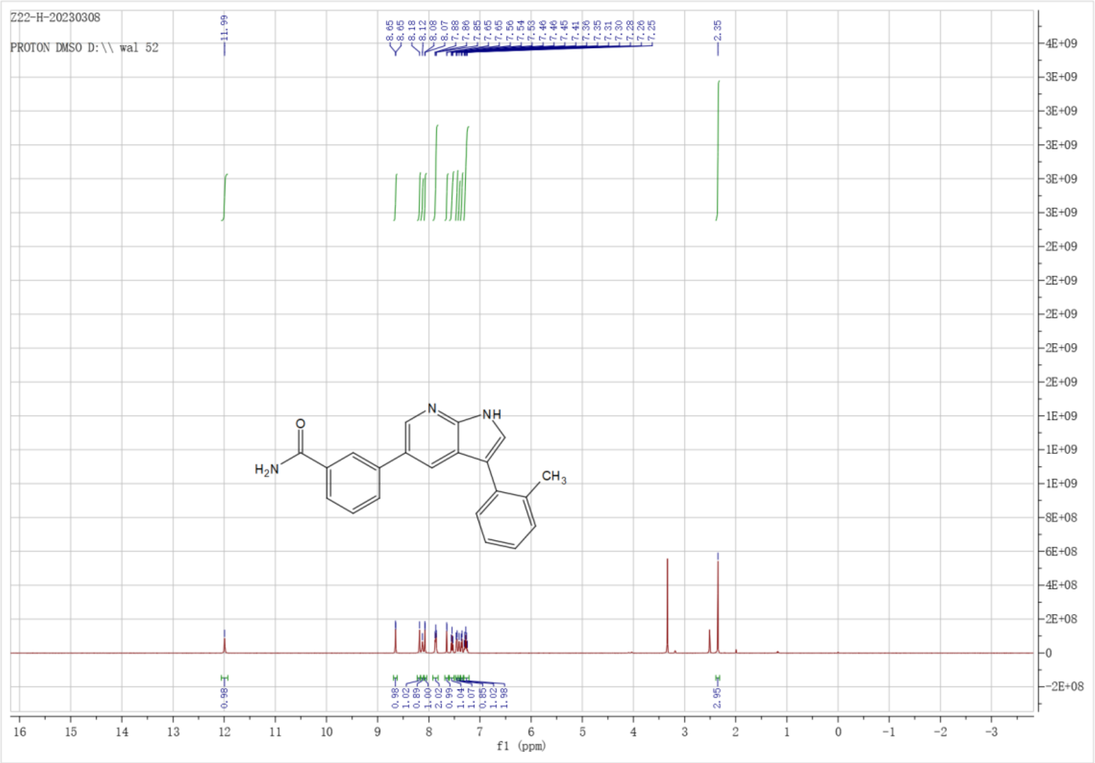

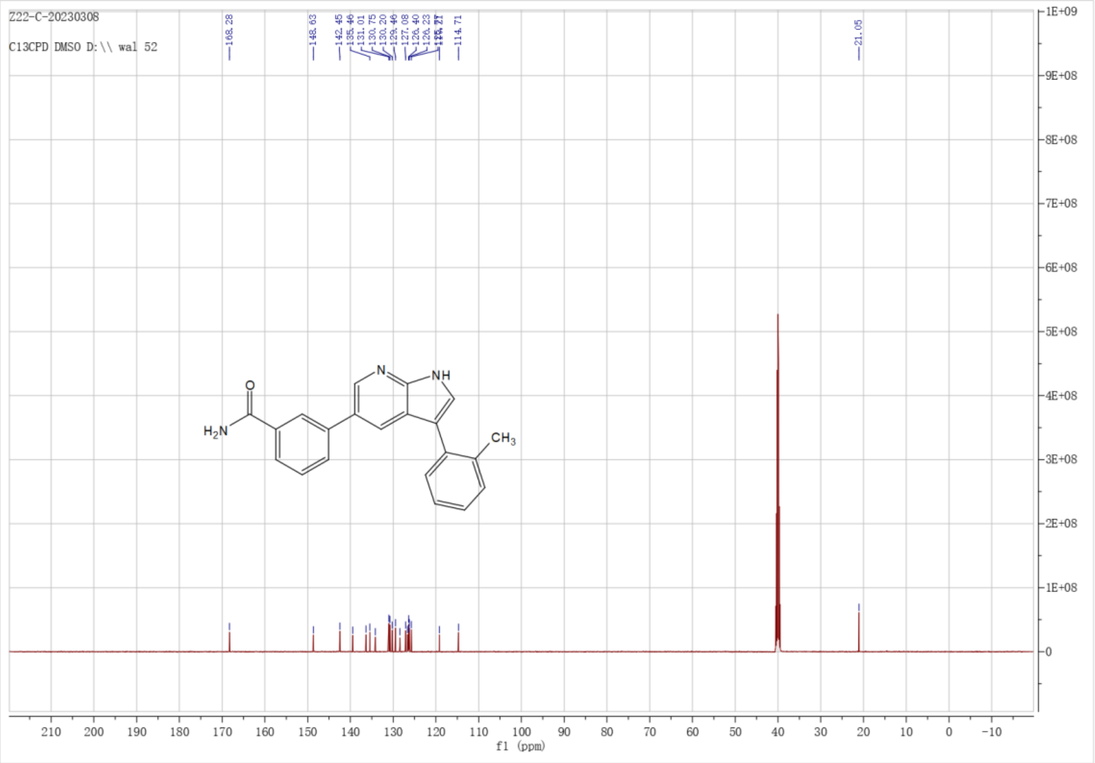

Sample Name	Position	Instrument Name	User Name
Inj Vol	InjPosition	SampleType	IRM Calibration Status
Data Filename	ACQ Method	Comment	Acquired Time

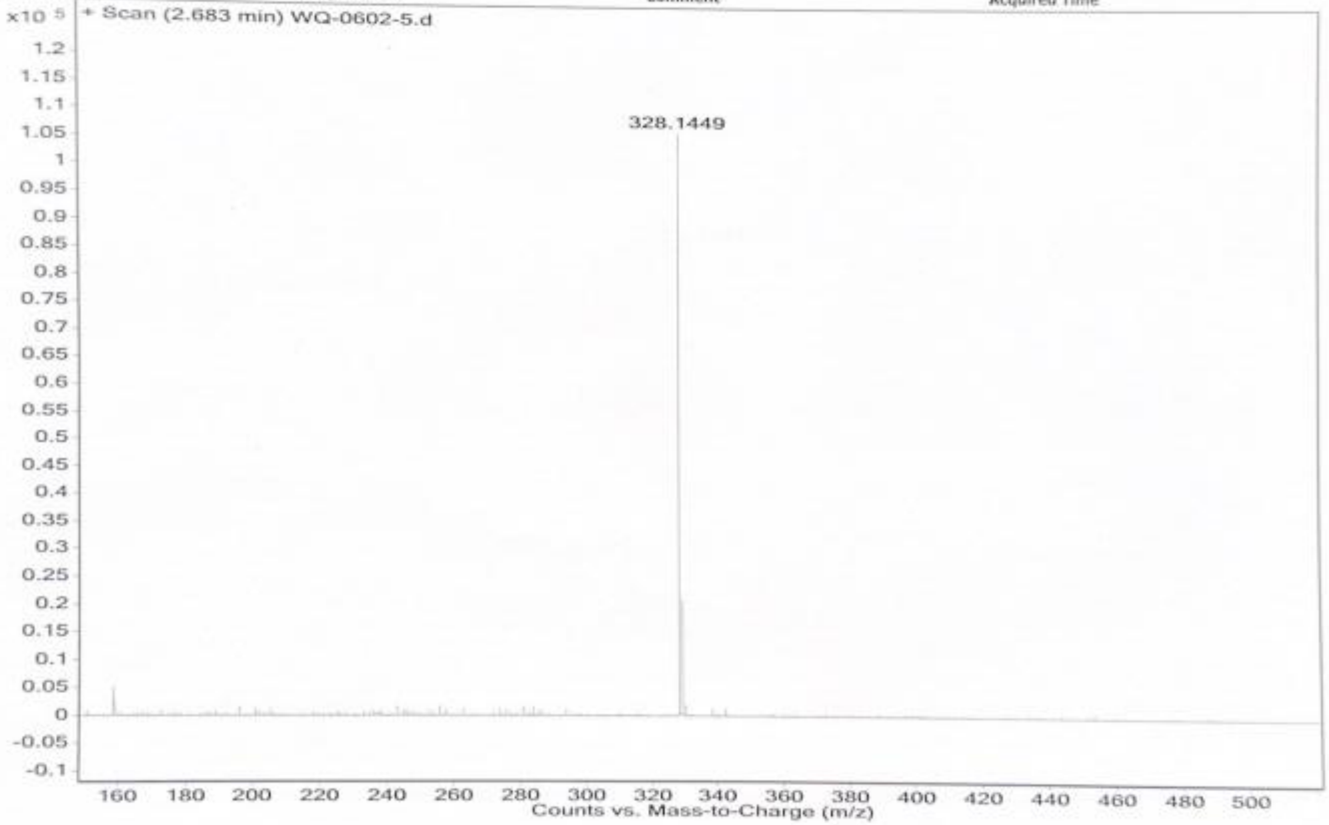

3-(3-(2-ethylphenyl)-1H-pyrrolo[2,3-b]pyridin-5-yl)benzamide (**24**)

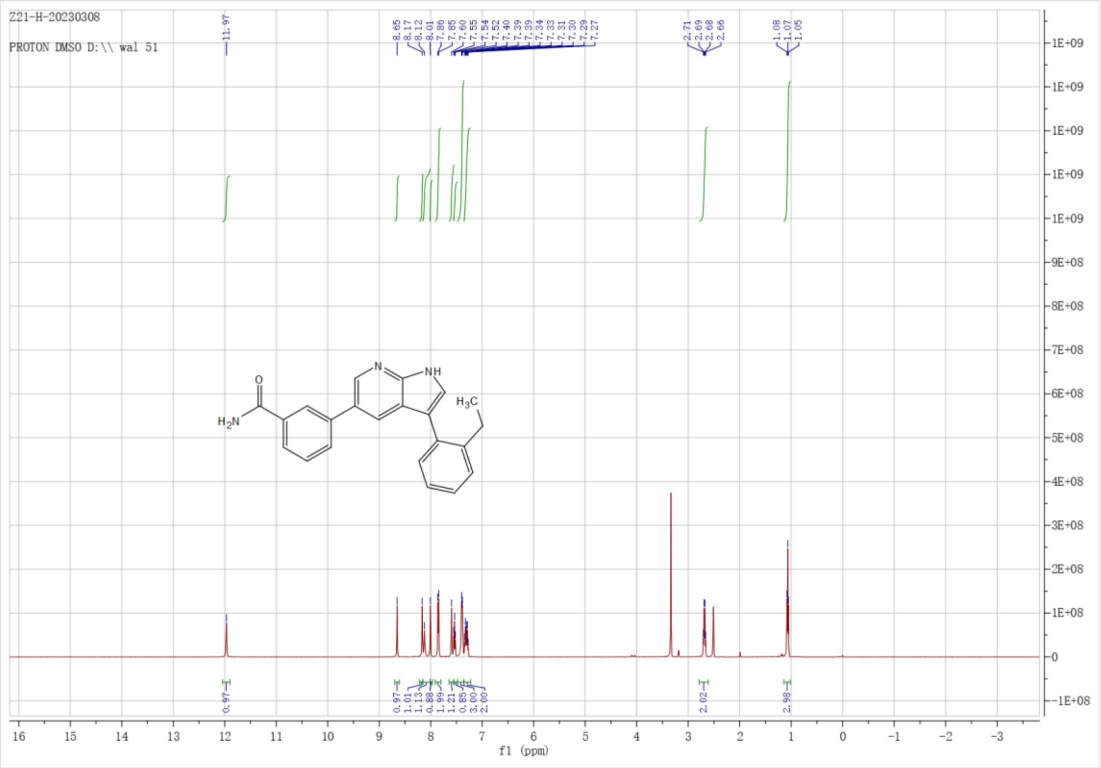

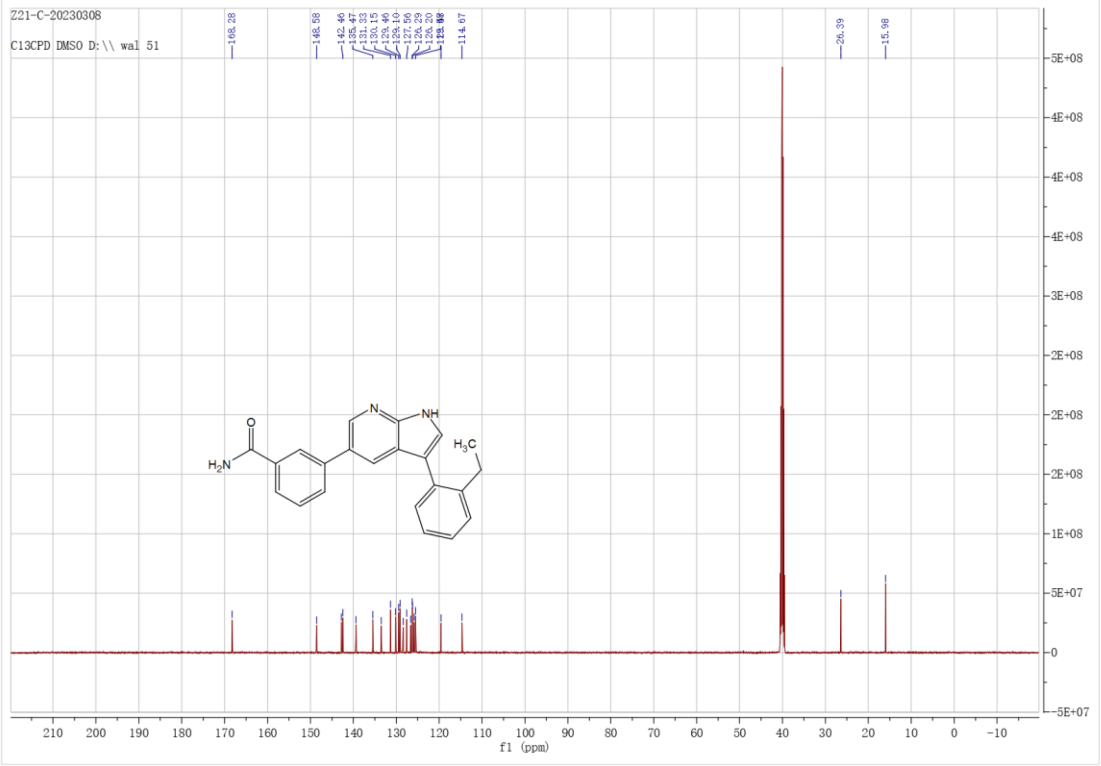

Sample Name	Position	Instrument Name	User Name
Inj Vol	InjPosition	SampleType	IRM Calibration Status
Data Filename	ACQ Method	Comment	Acquired Time

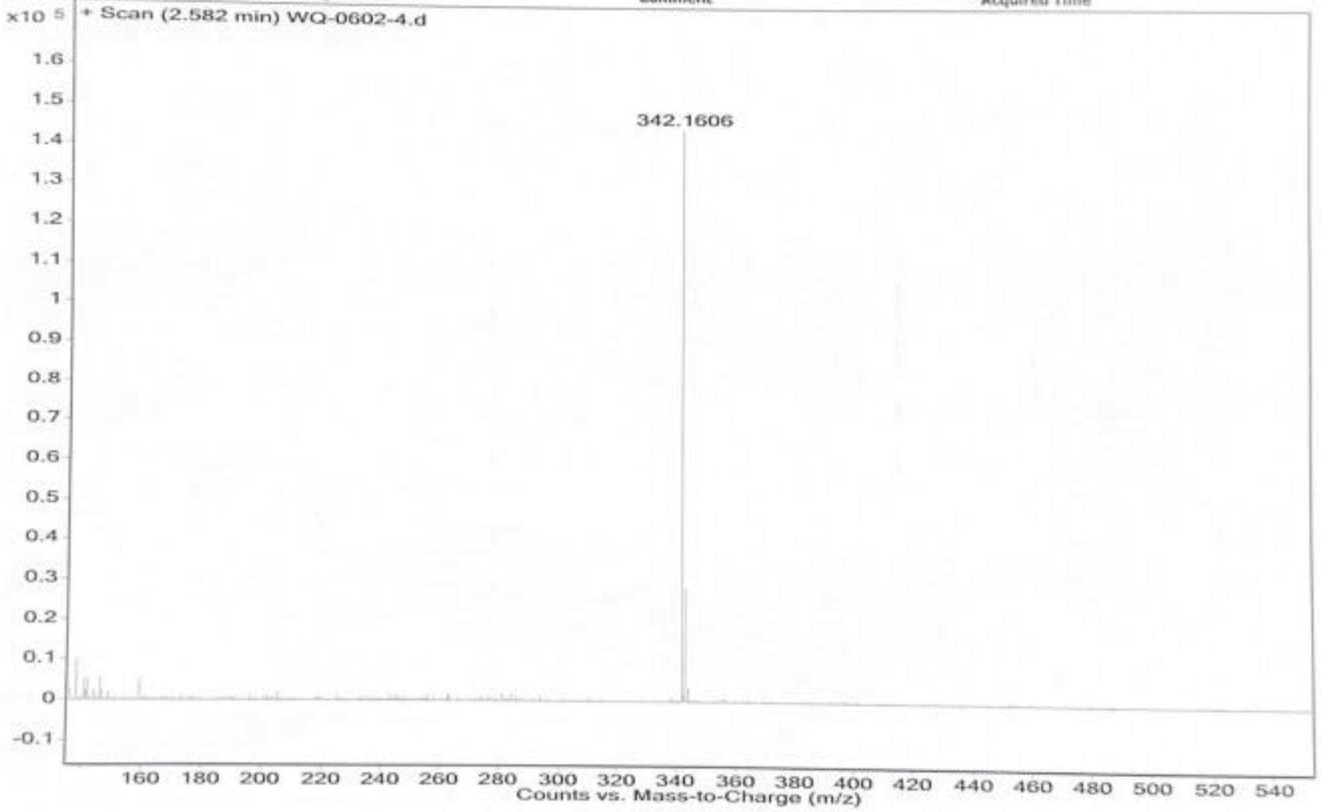

3-(3-(2-methoxyphenyl)-1H-pyrrolo[2,3-b]pyridin-5-yl)benzamide (**25**)

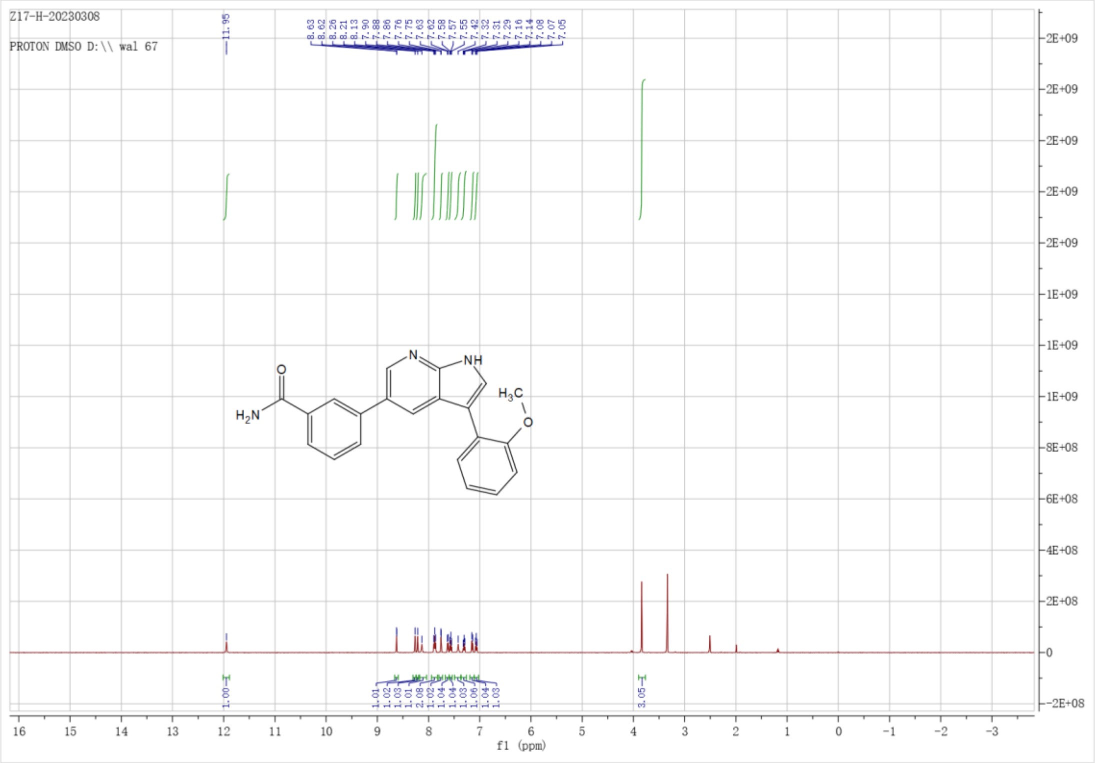

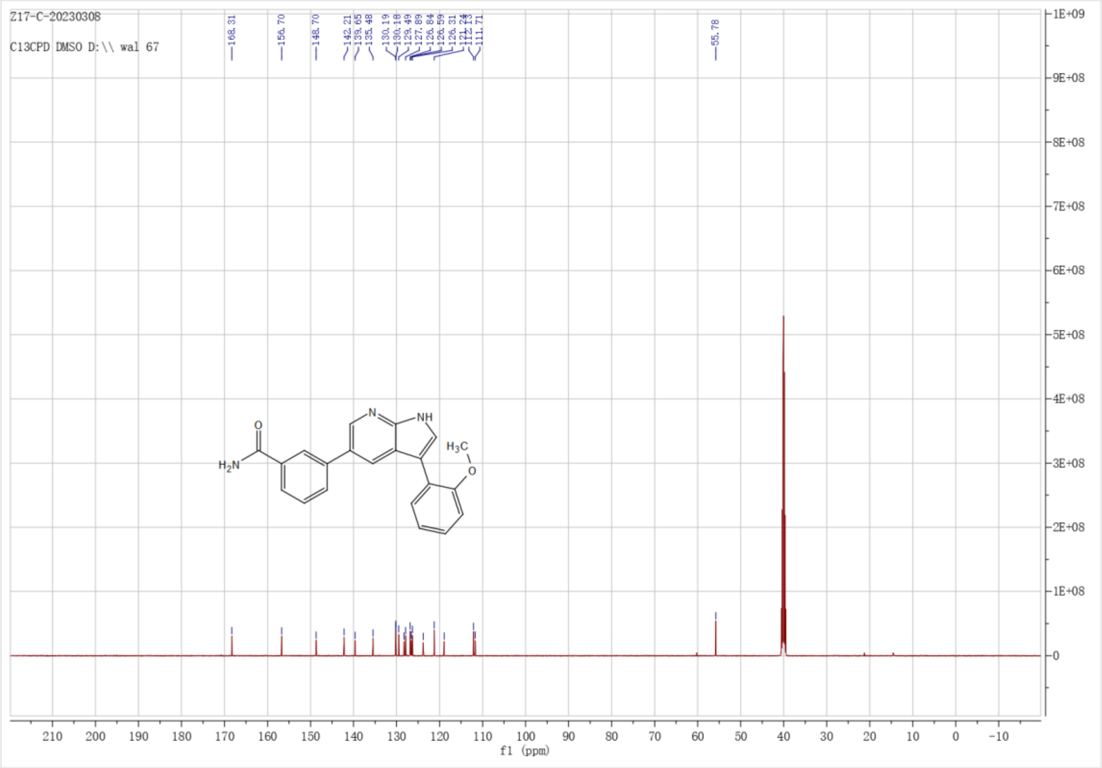

Sample Name	Position	Instrument Name	User Name
Inj Vol	InjPosition	SampleType	IRM Calibration Status
Data Filename	ACQ Method	Comment	Acquired Time

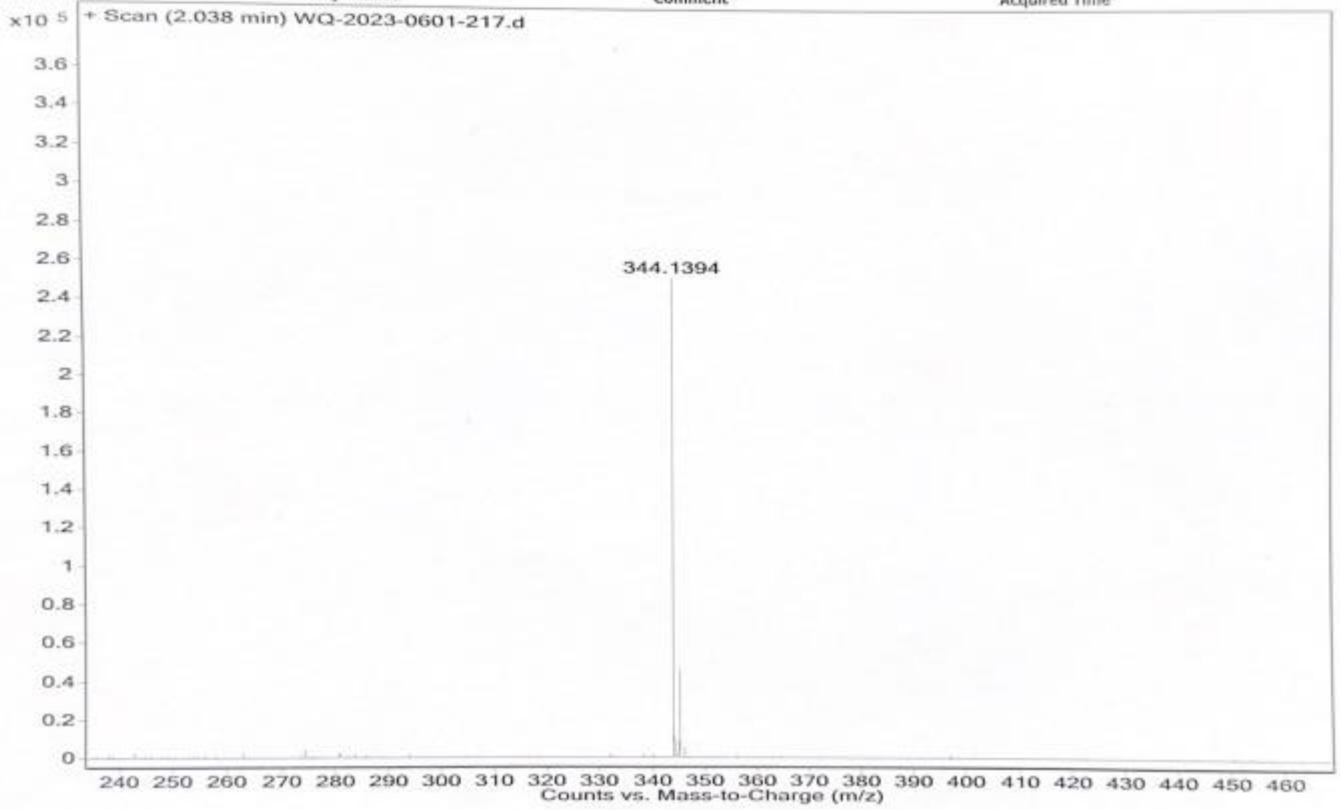

3-(3-(2-isopropylphenyl)-1H-pyrrolo[2,3-b]pyridin-5-yl)benzamide (**26**)

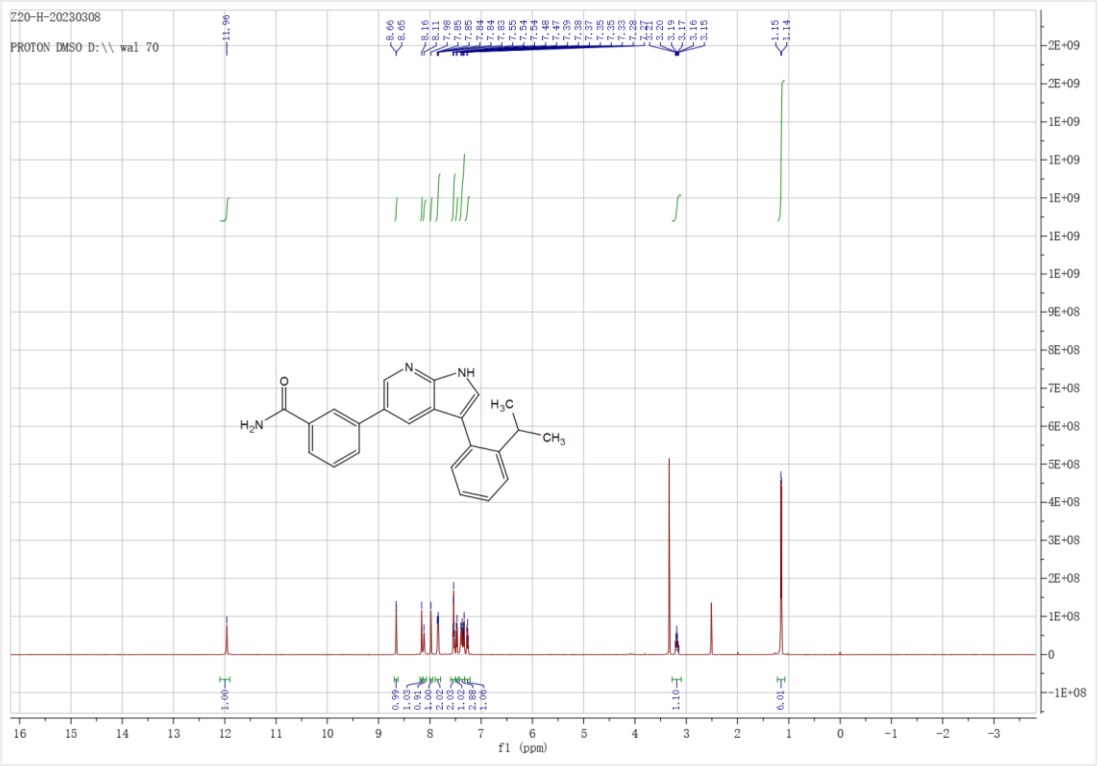

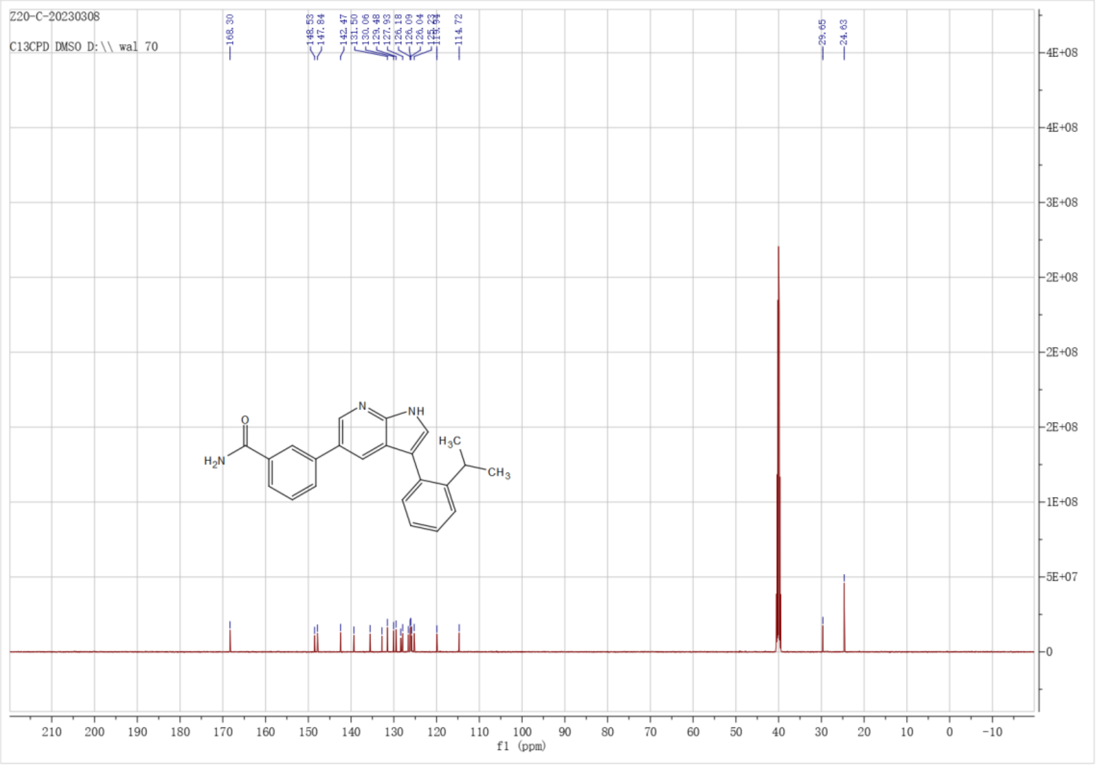

Sample Name	Position	Instrument Name	User Name
Inj Vol	InjPosition	SampleType	IRM Calibration Status
Data Filename	ACQ Method	Comment	Acquired Time

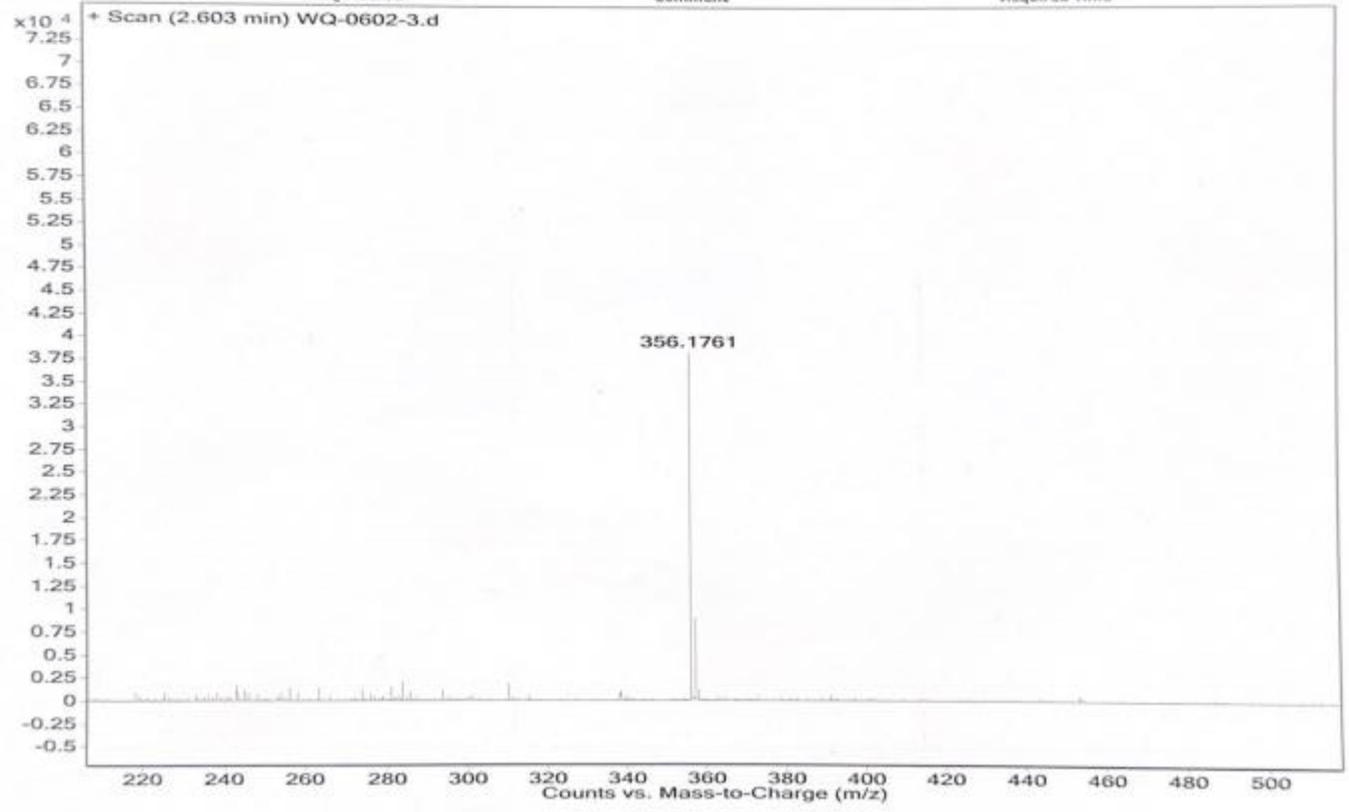

3-(3-(2-(trifluoromethyl)phenyl)-1H-pyrrolo[2,3-b]pyridin-5-yl)benzamide (**27**)

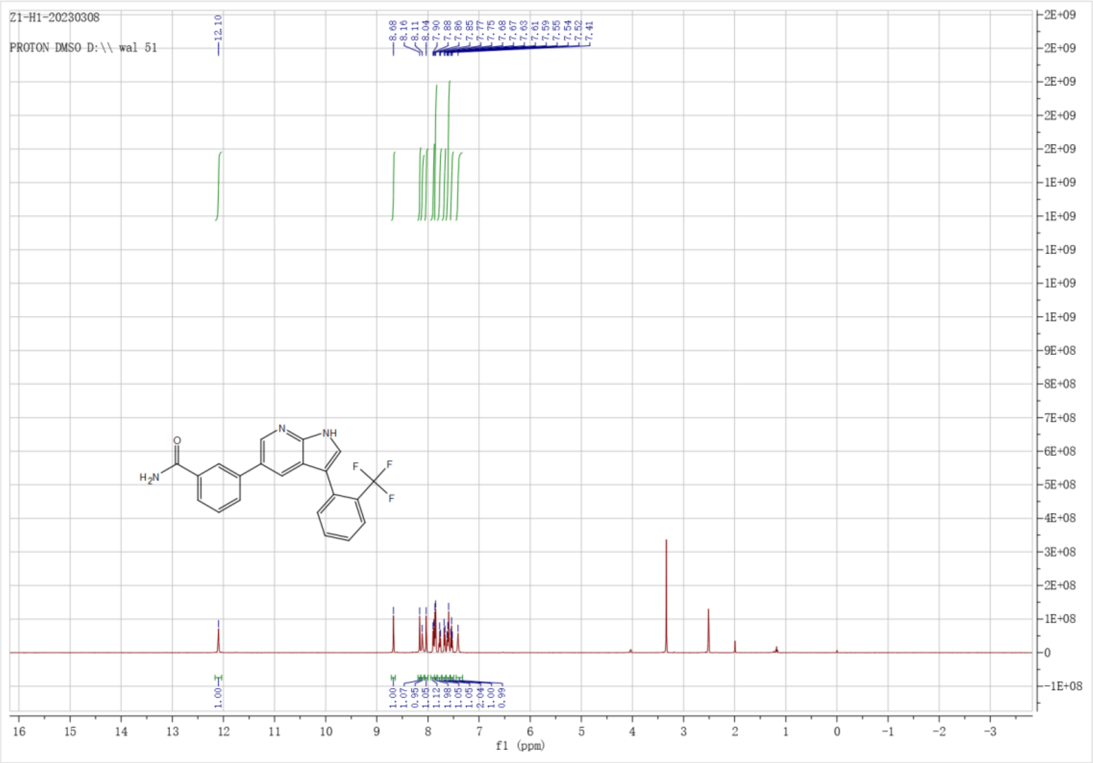

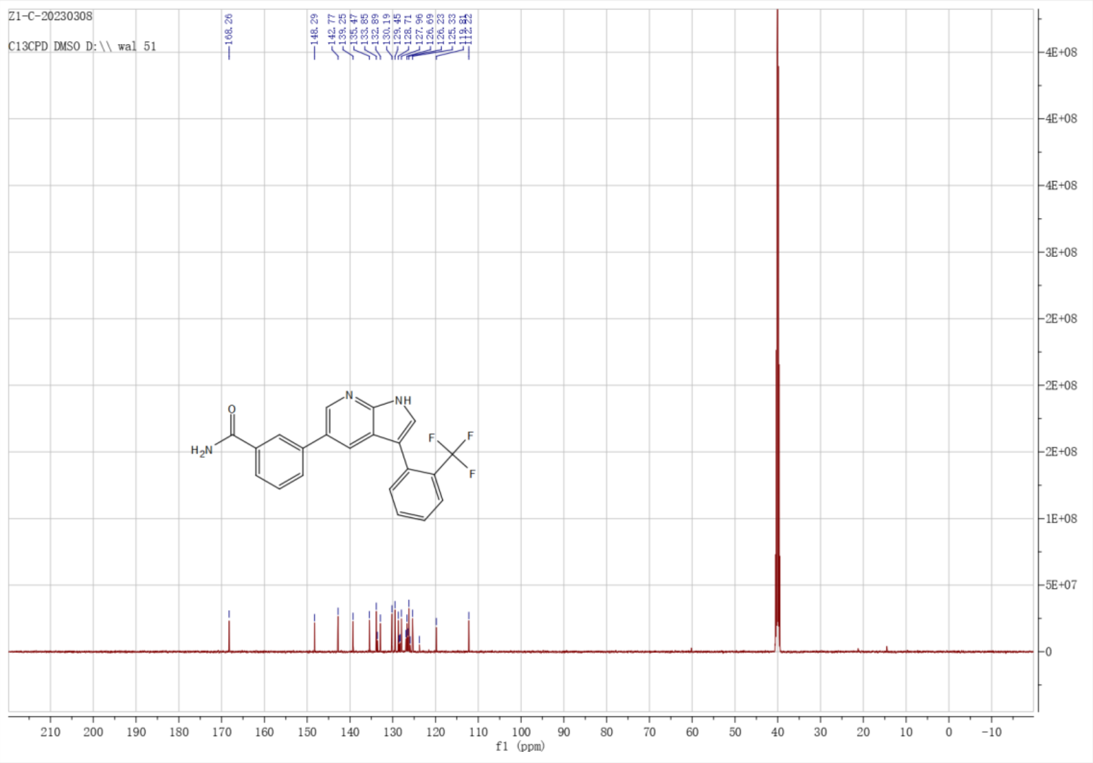

Sample Name	Position	Instrument Name	User Name
Inj Vol	InjPosition	SampleType	IRM Calibration Status
Data Filename	ACQ Method	Comment	Acquired Time

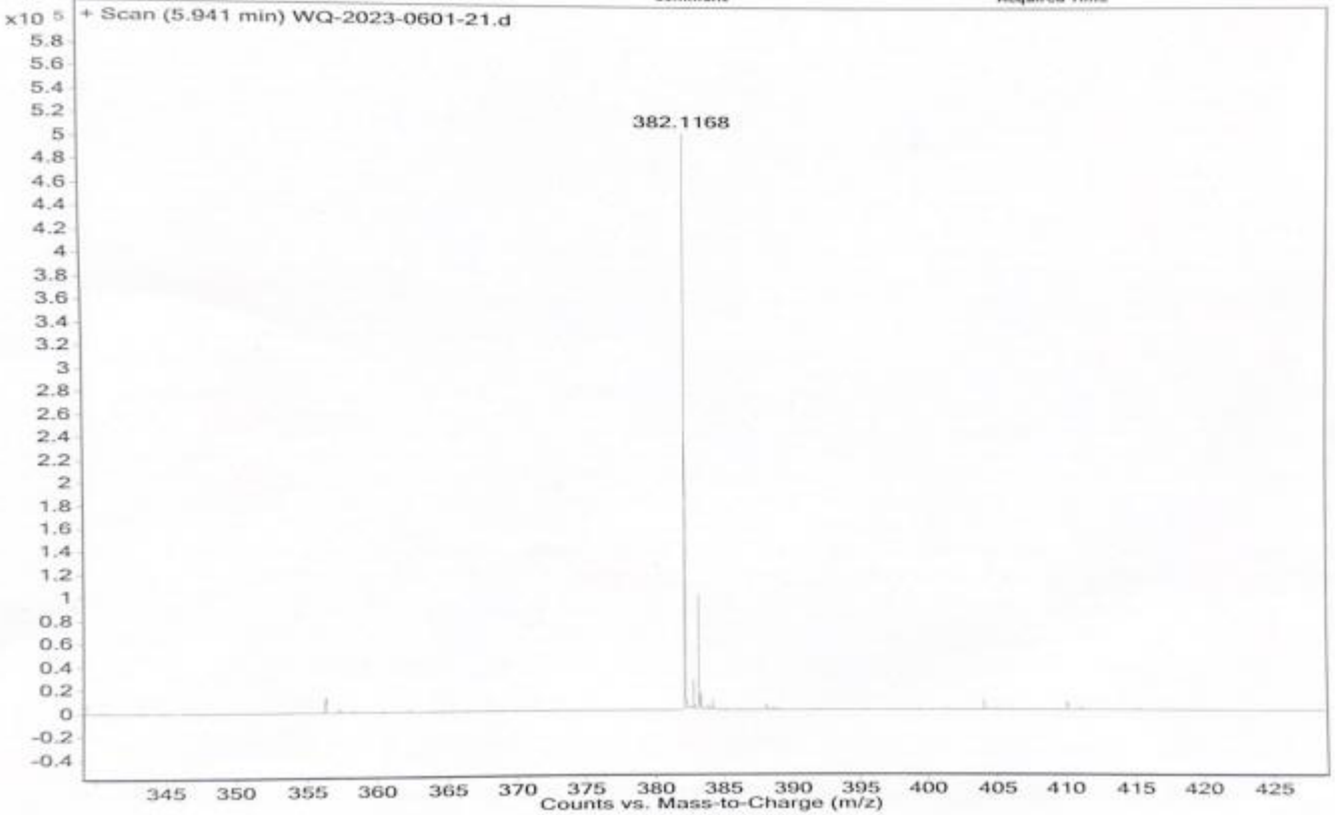

3-(3-(2-(trifluoromethoxy)phenyl)-1H-pyrrolo[2,3-b]pyridin-5-yl)benzamide (**28**)

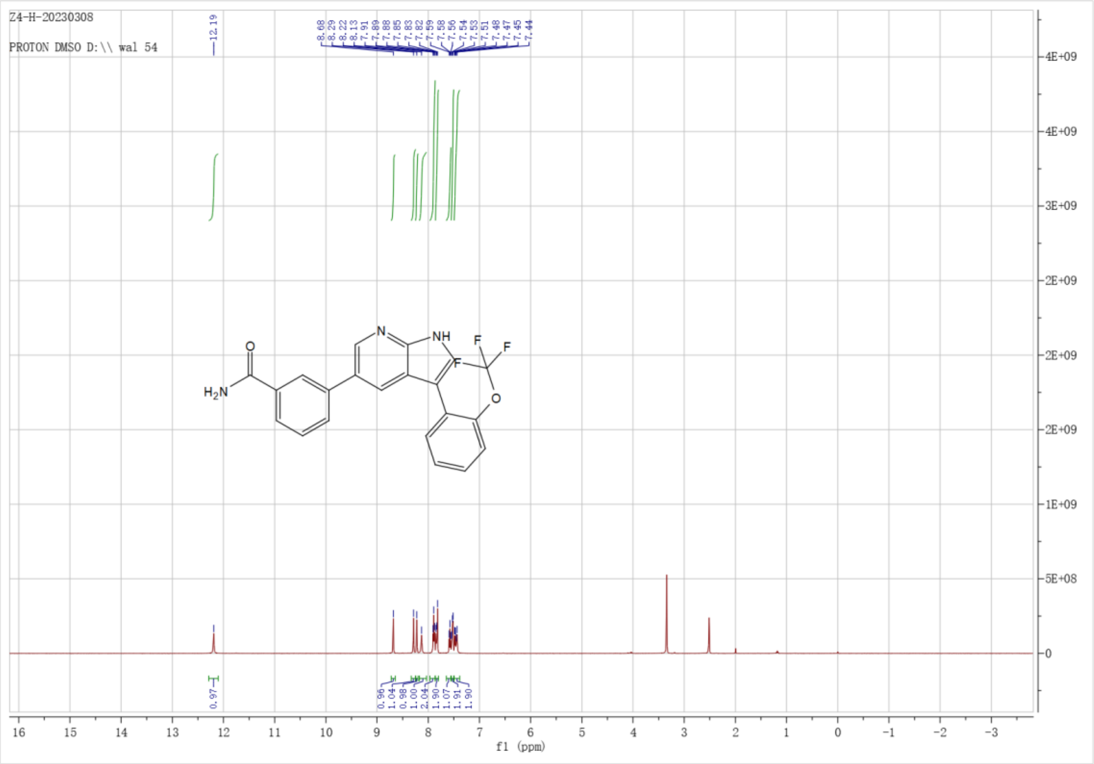

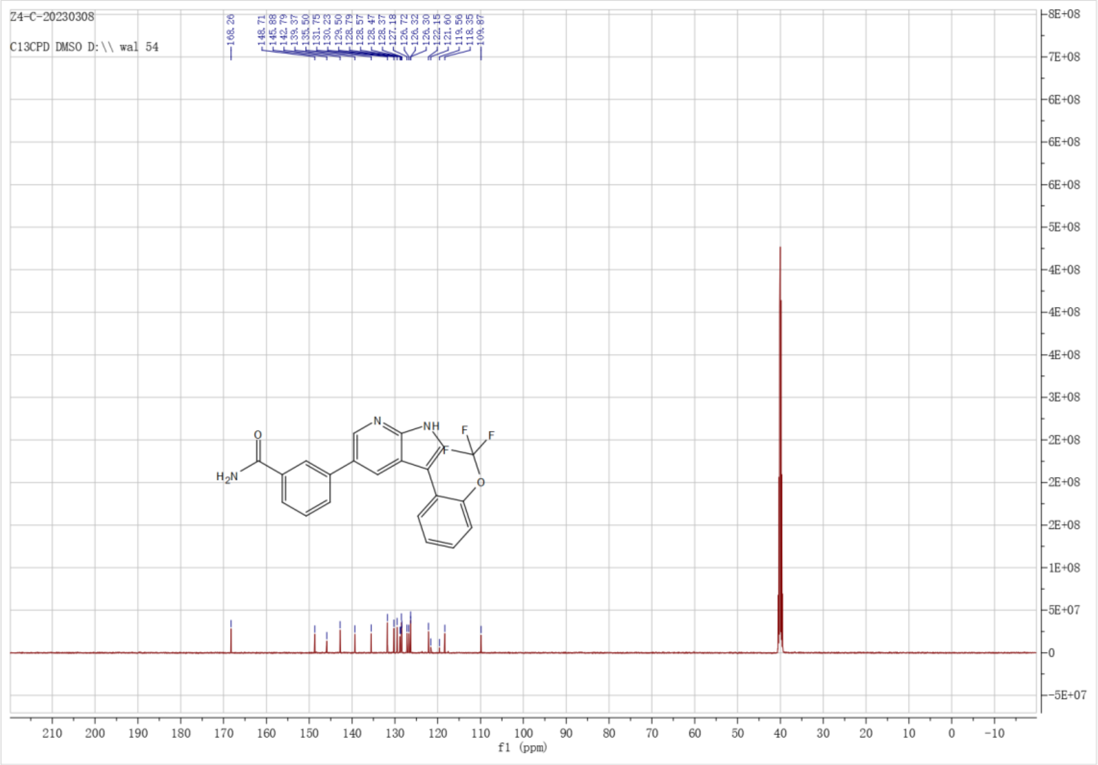

Sample Name	Position	Instrument Name	User Name
Inj Vol	InjPosition	SampleType	IRM Calibration Status
Data Filename	ACQ Method	Comment	Acquired Time

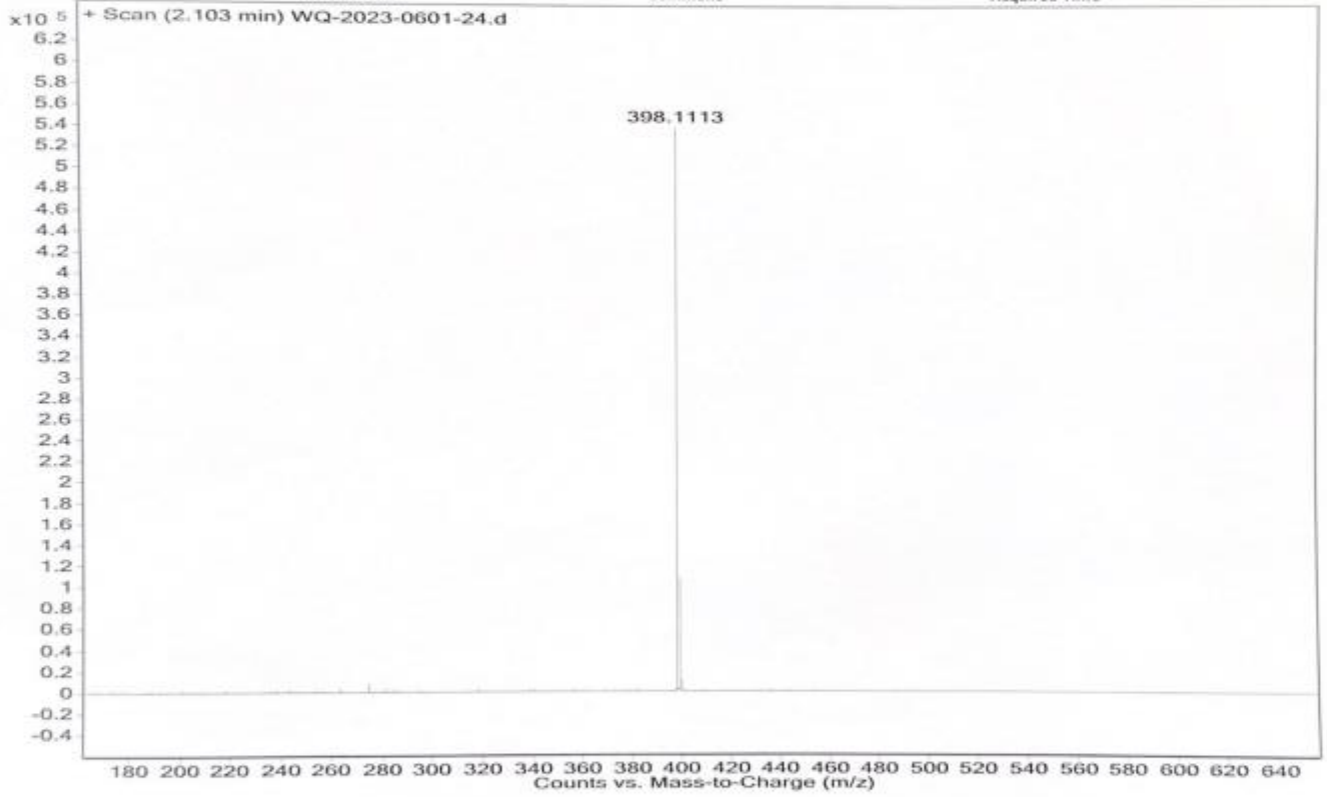

3-(3-(3-fluorophenyl)-1H-pyrrolo[2,3-b]pyridin-5-yl)benzamide (**29**)

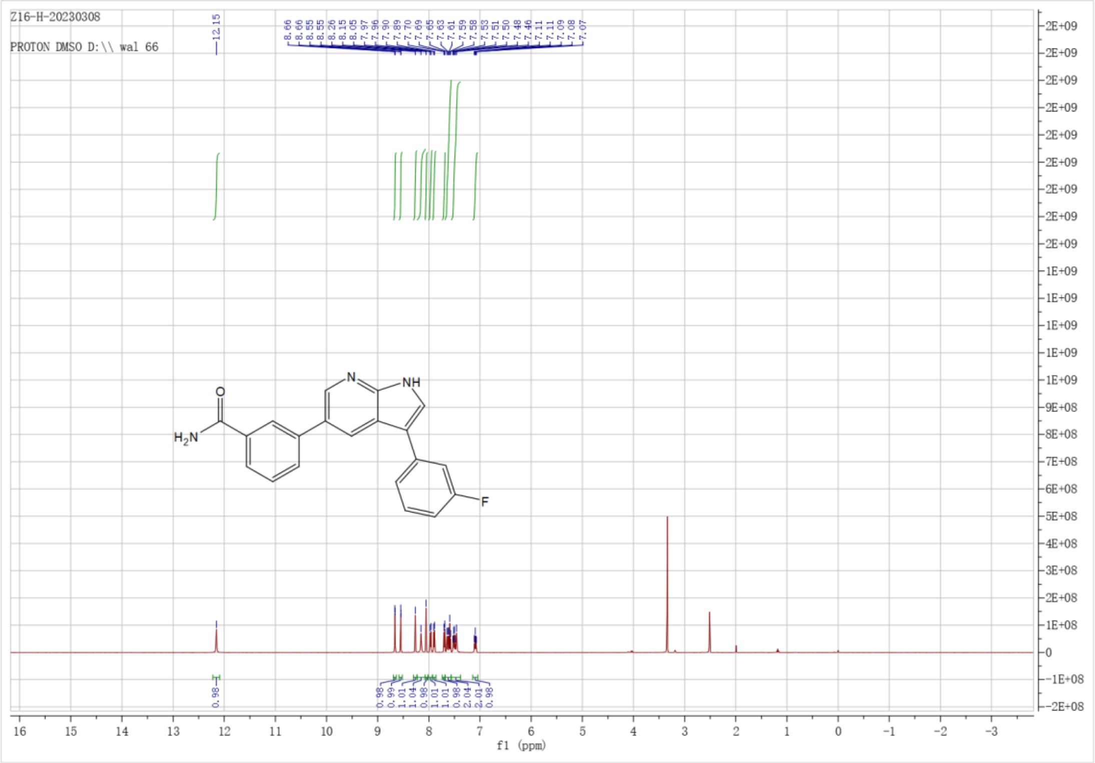

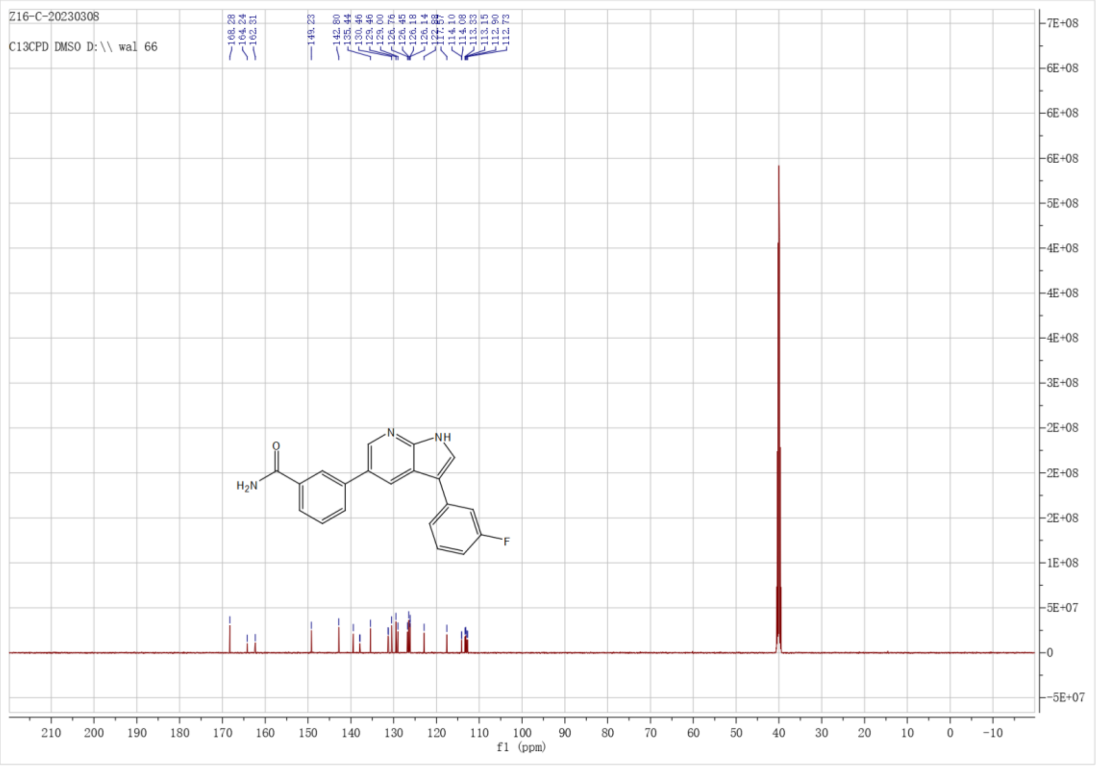

Sample Name	Position	Instrument Name	User Name
Inj Vol	InjPosition	SampleType	IRM Calibration Status
Data Filename	ACQ Method	Comment	Acquired Time

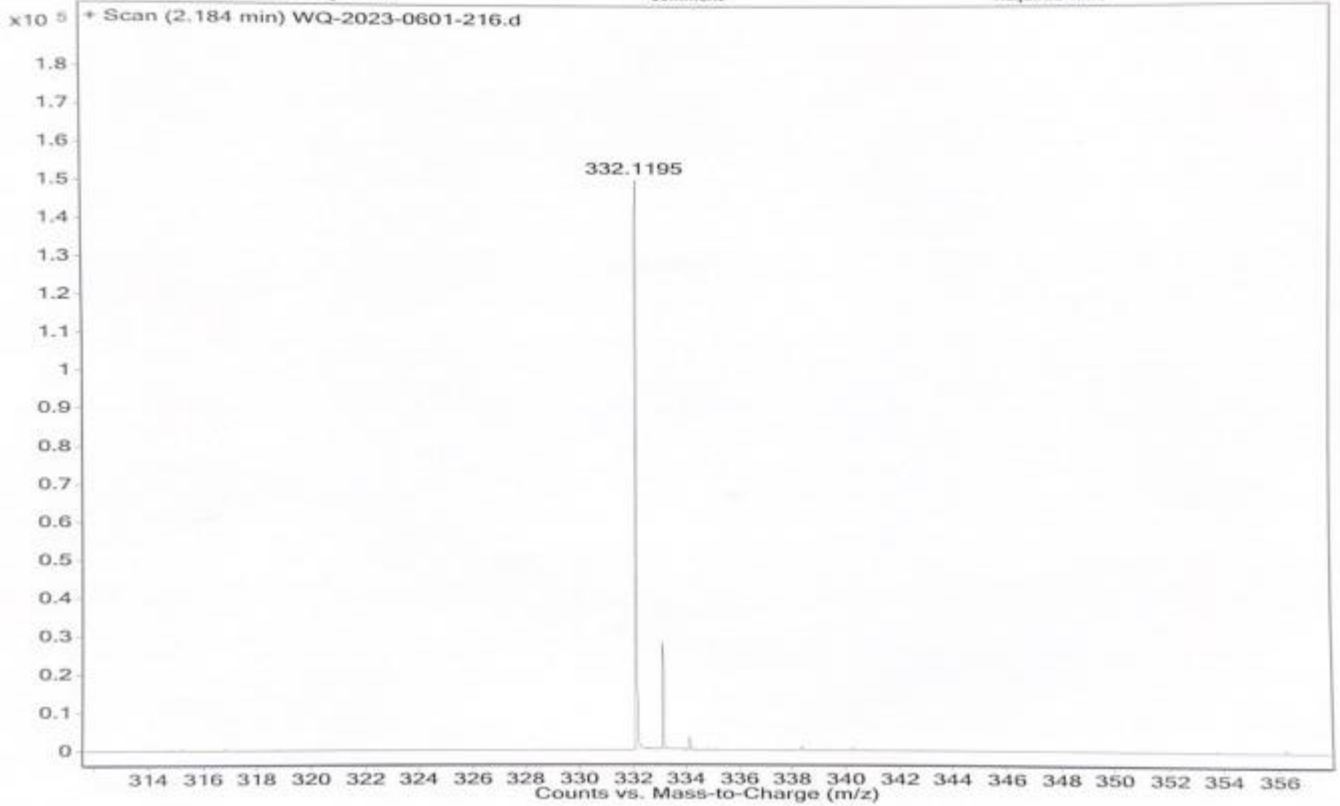

3-(3-(3-chlorophenyl)-1H-pyrrolo[2,3-b]pyridin-5-yl)benzamide （**30**）

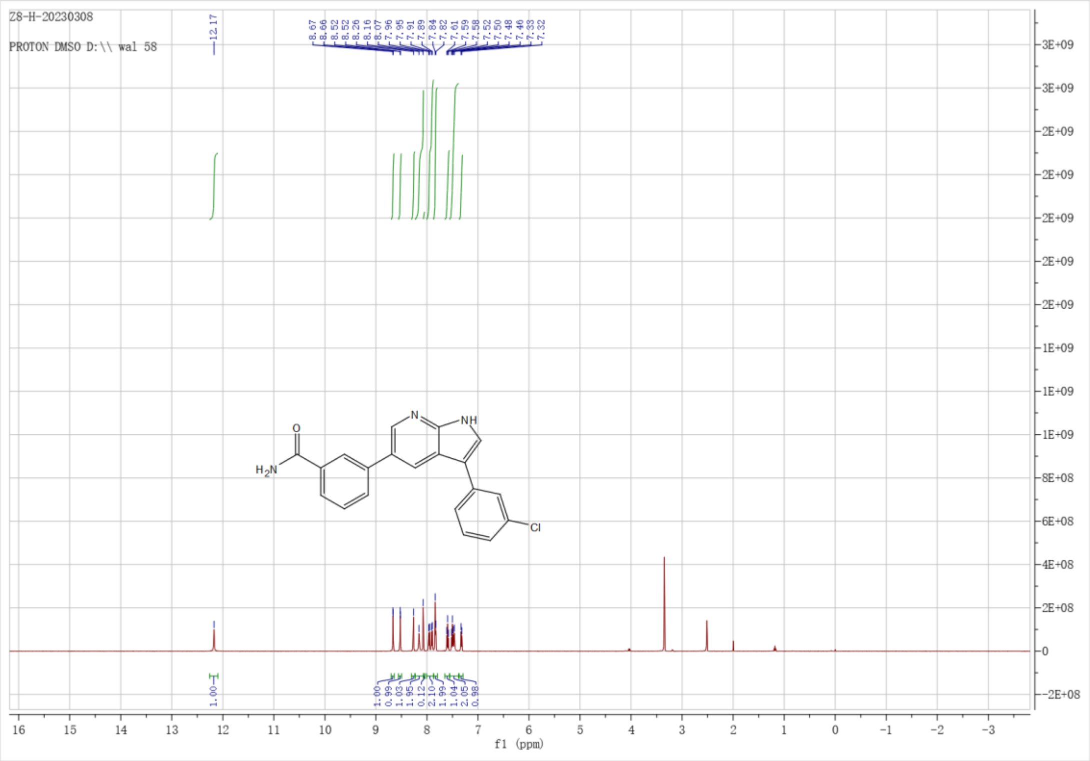

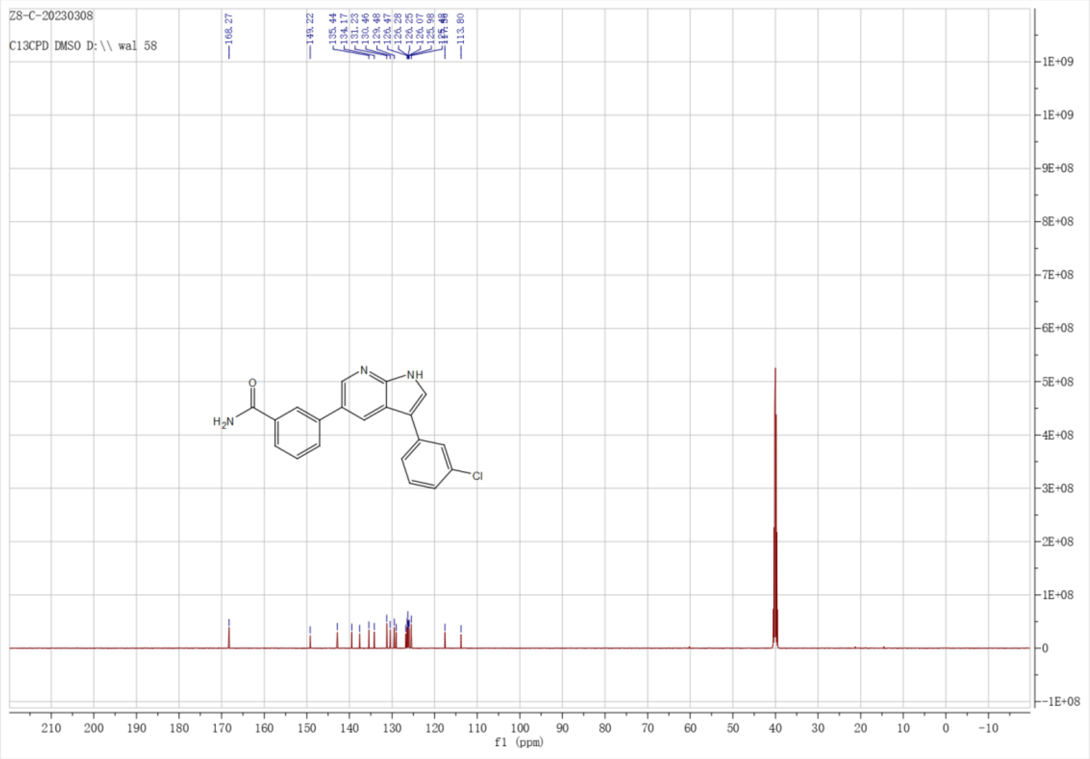

Sample Name	Position	Instrument Name	User Name
Inj Vol	InjPosition	SampleType	IRM Calibration Status
Data Filename	ACQ Method	Comment	Acquired Time

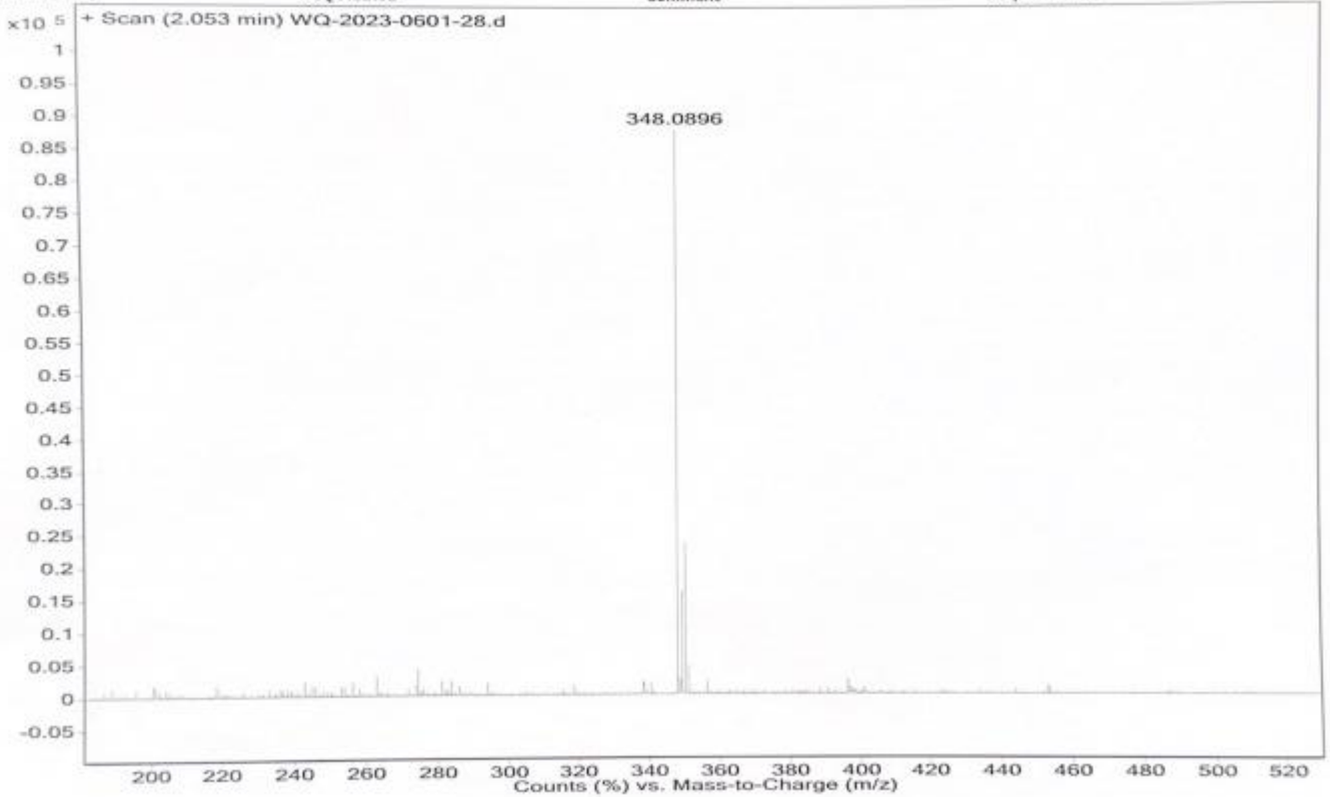

3-(3-(3-hydroxyphenyl)-1H-pyrrolo[2,3-b]pyridin-5-yl)benzamide (**31**)

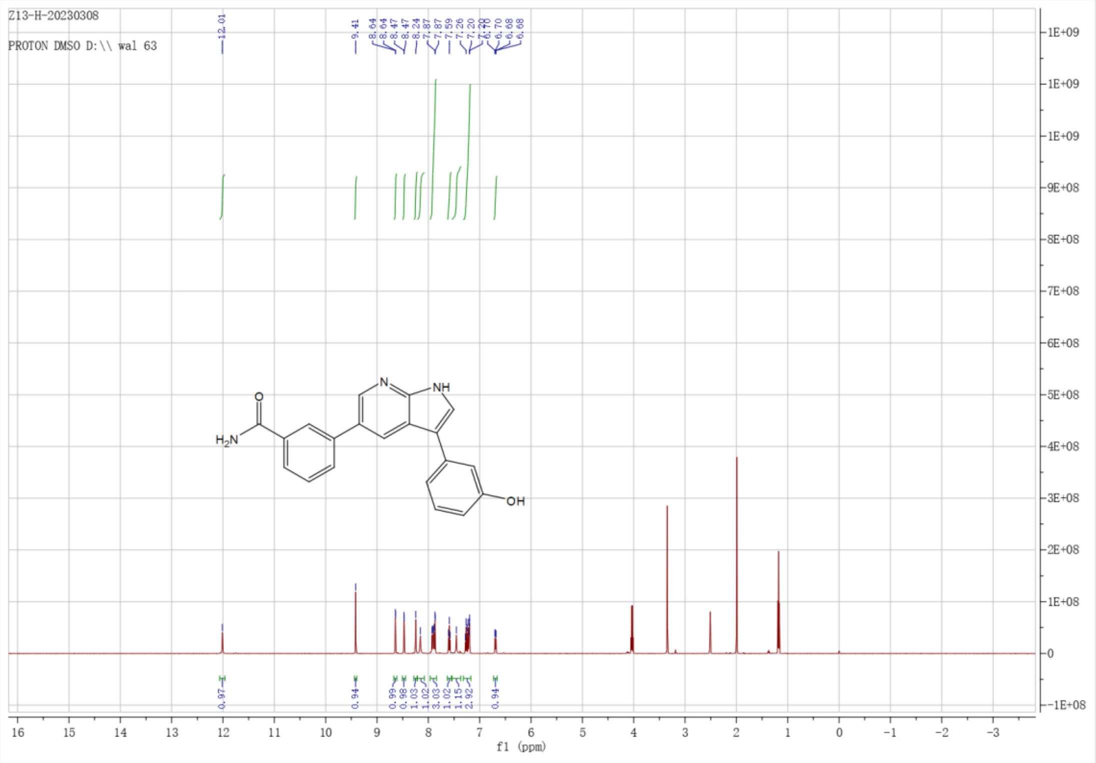

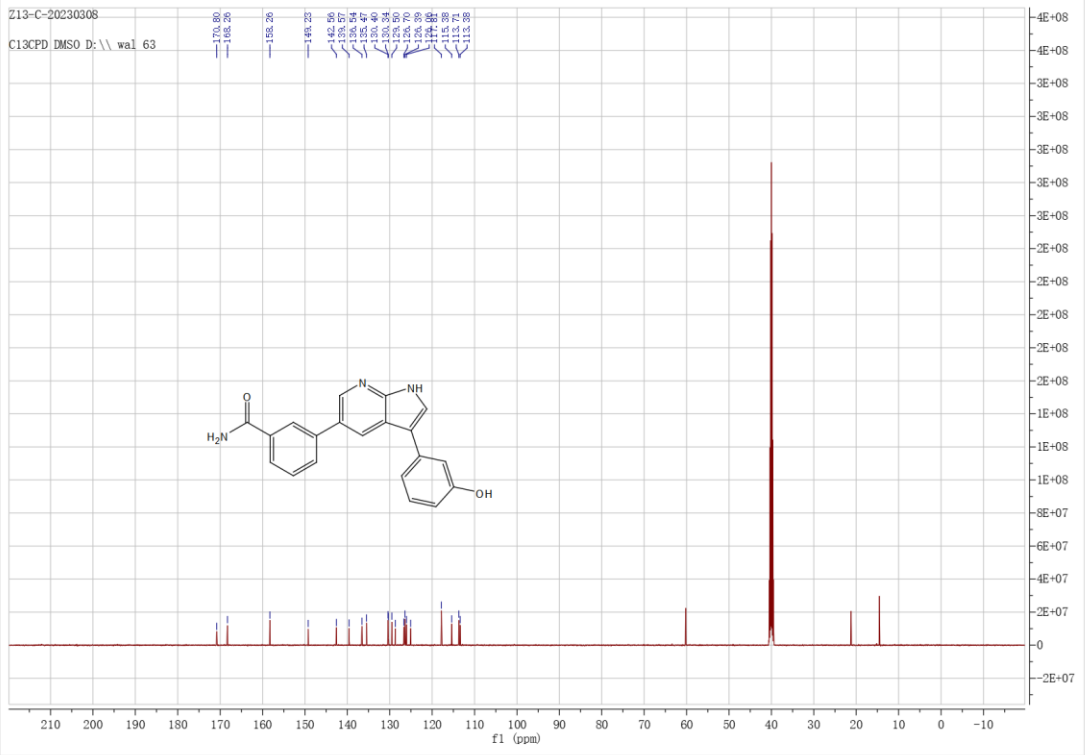

Sample Name	Position	Instrument Name	User Name
Inj Vol	InjPosition	SampleType	IRM Calibration Status
Data Filename	ACQ Method	Comment	Acquired Time

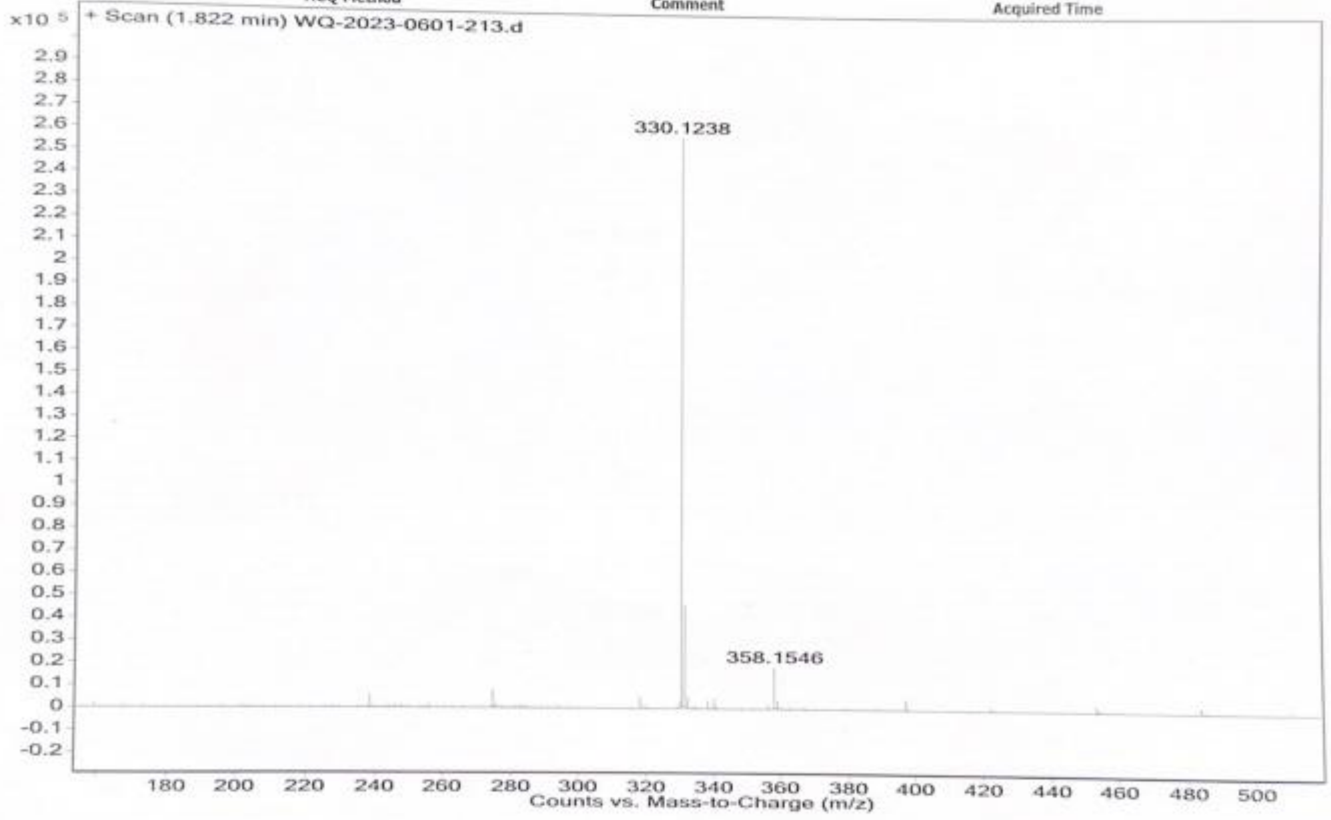

3-(3-(3-methoxyphenyl)-1H-pyrrolo[2,3-b]pyridin-5-yl)benzamide (**32**)

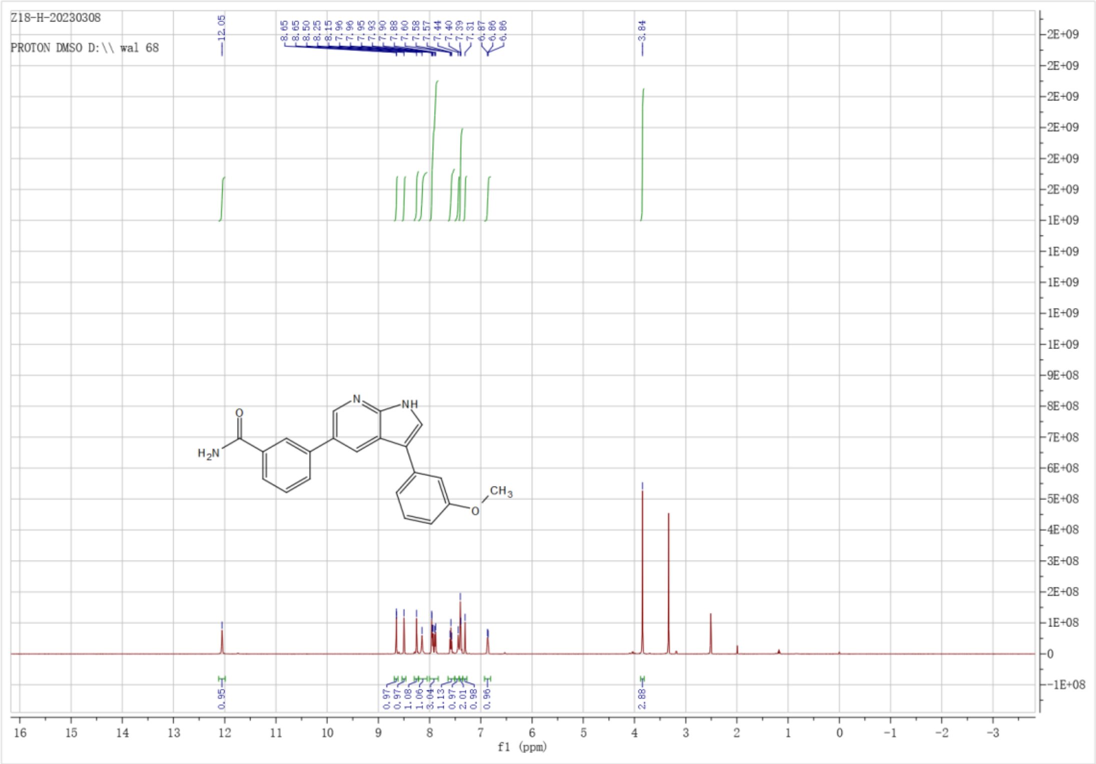

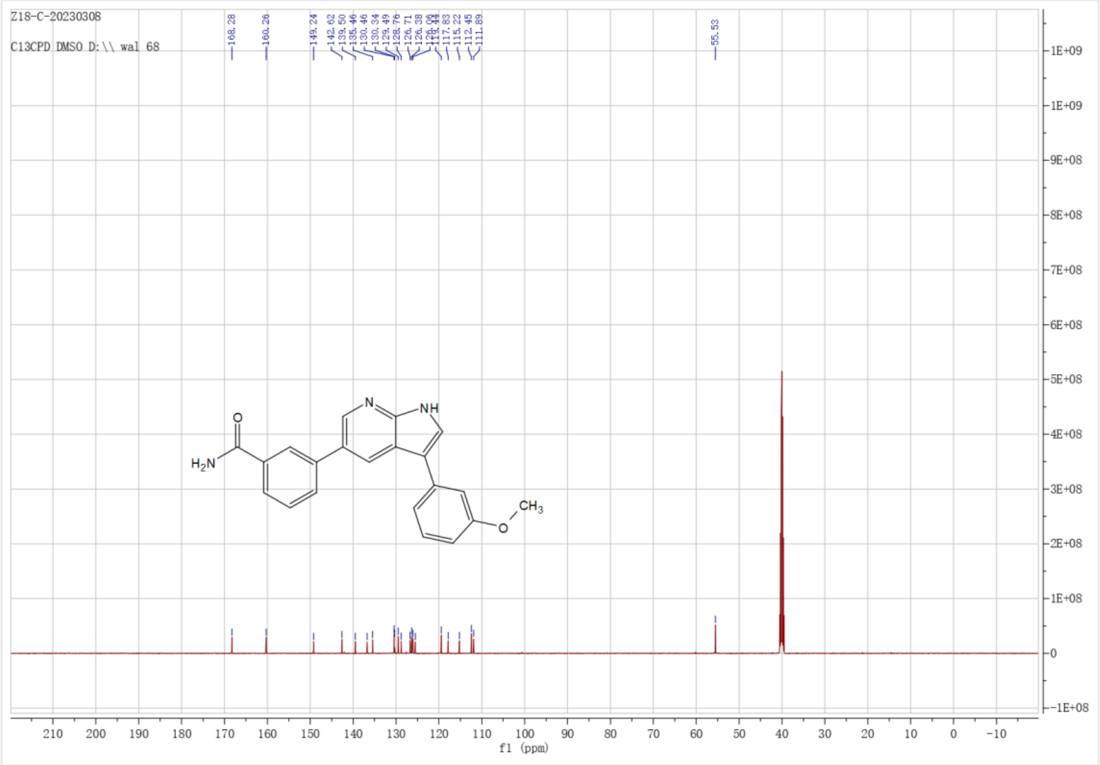

Sample Name	Position	Instrument Name	User Name
Inj Vol	InjPosition	SampleType	IRM Calibration Status
Data Filename	ACQ Method	Comment	Acquired Time

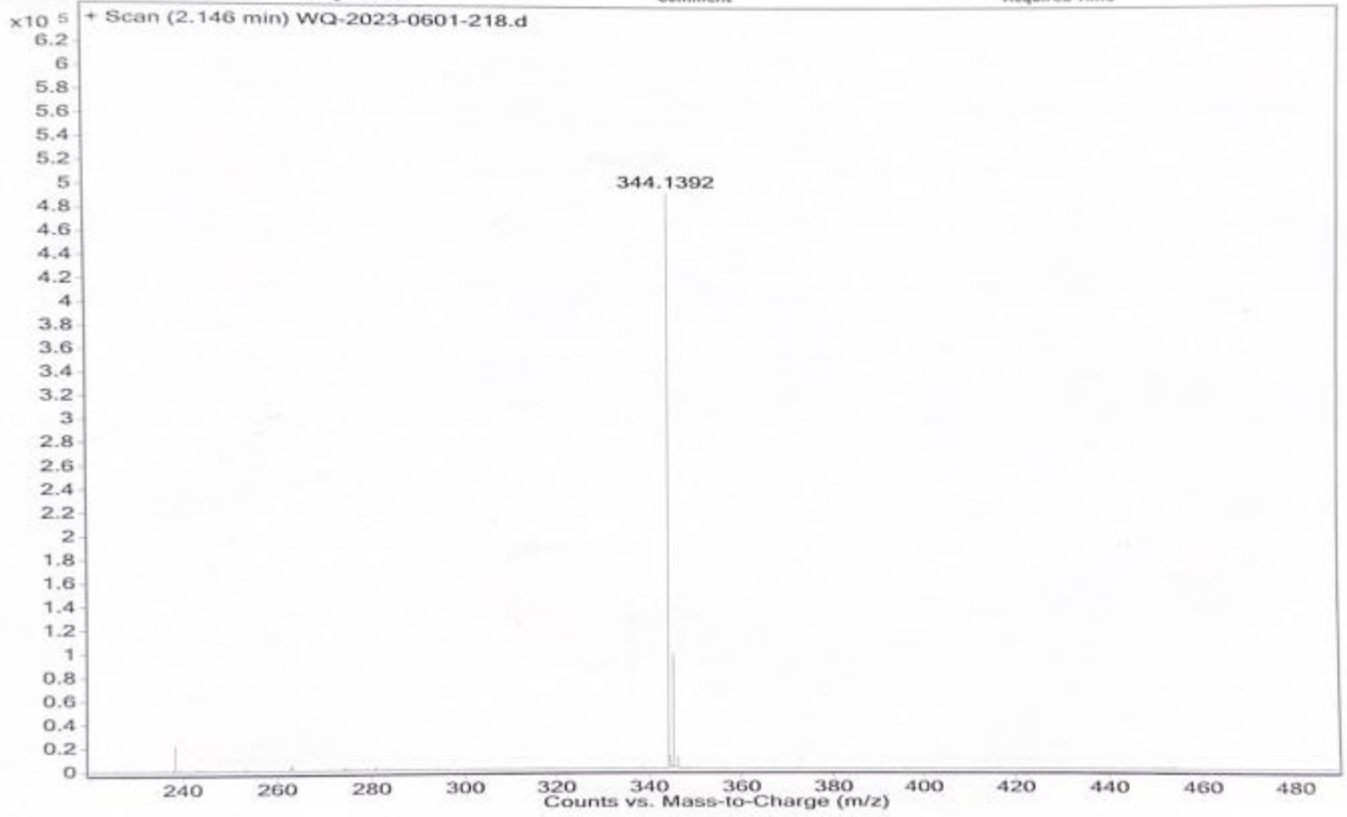

3-(3-(3-(trifluoromethyl)phenyl)-1H-pyrrolo[2,3-b]pyridin-5-yl)benzamide (**33**)

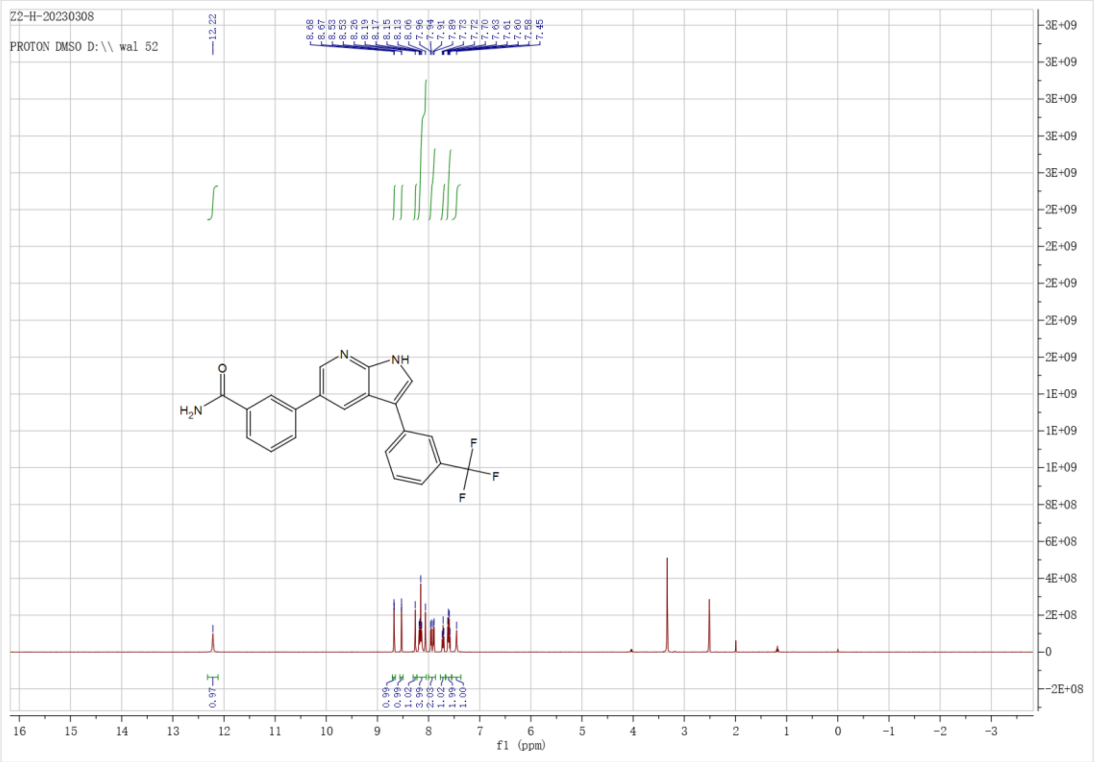

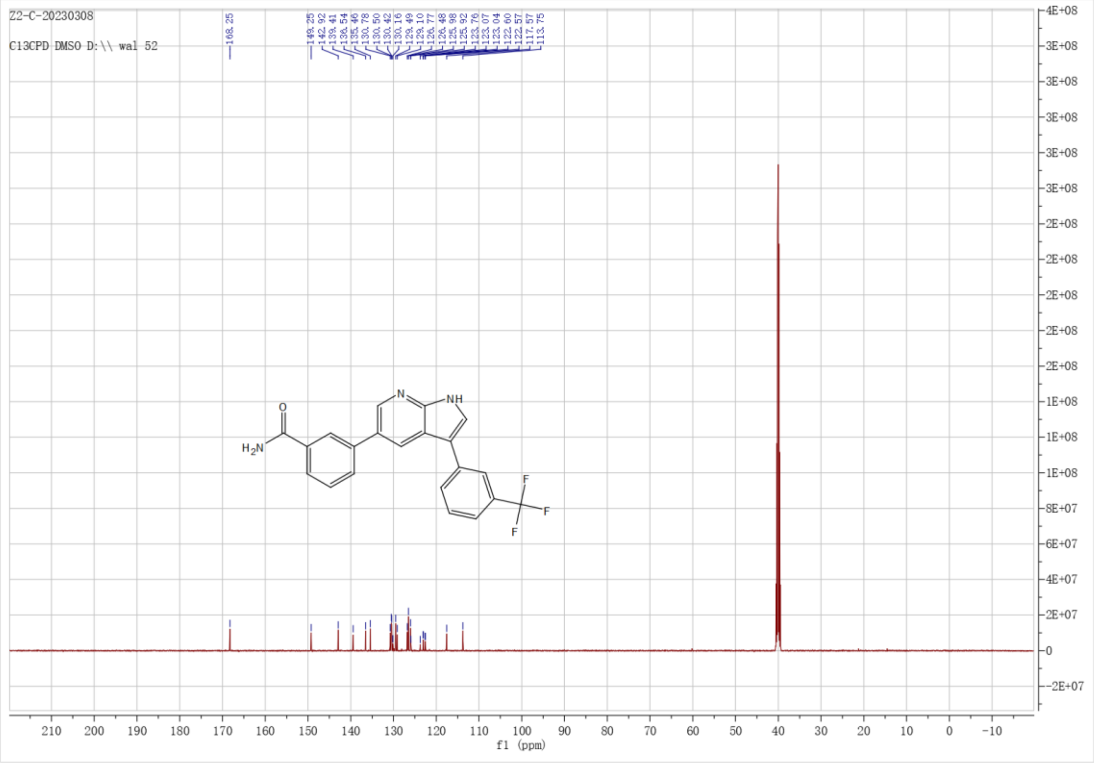

Sample Name	Position	Instrument Name	User Name
Inj Vol	InjPosition	SampleType	IRM Calibration Status
Data Filename	ACQ Method	Comment	Acquired Time

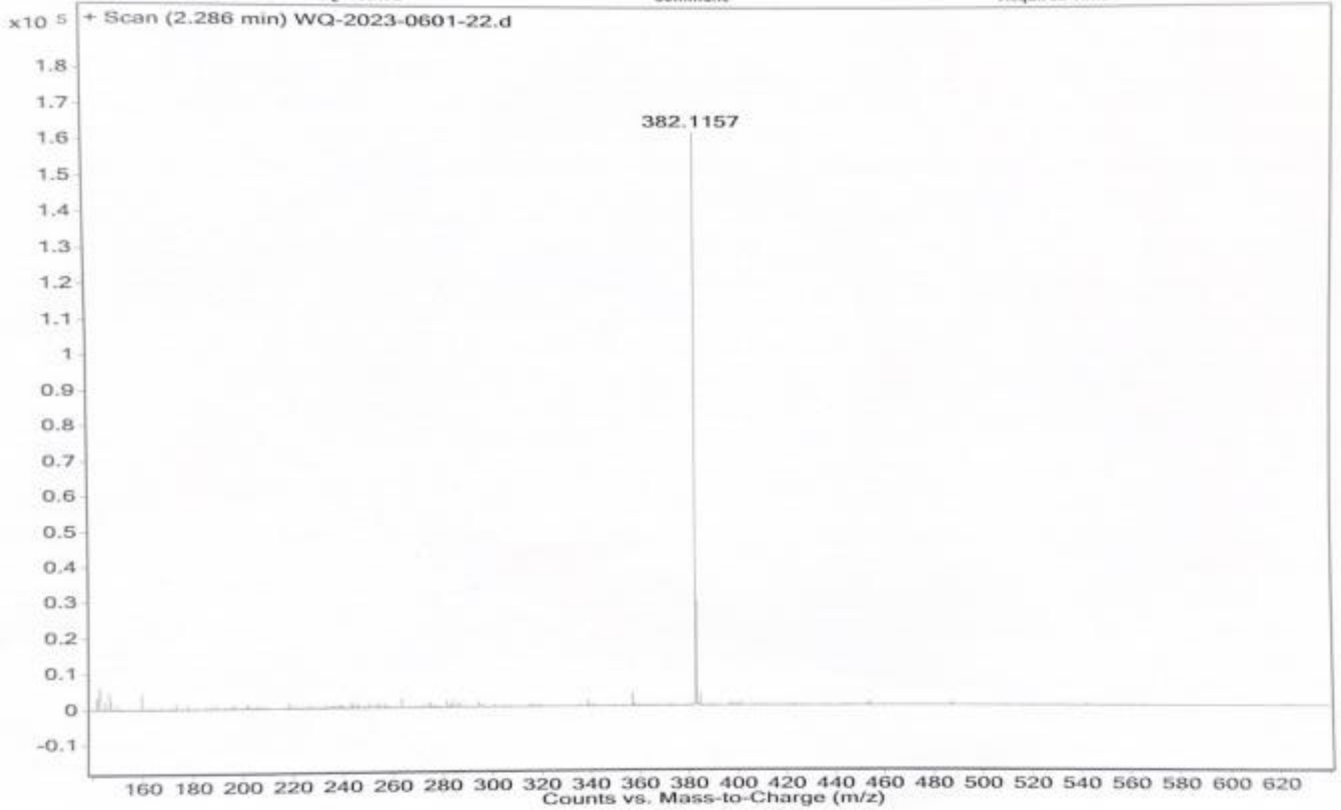

3-(3-(3-(trifluoromethoxy)phenyl)-1H-pyrrolo[2,3-b]pyridin-5-yl)benzamide (**34**)

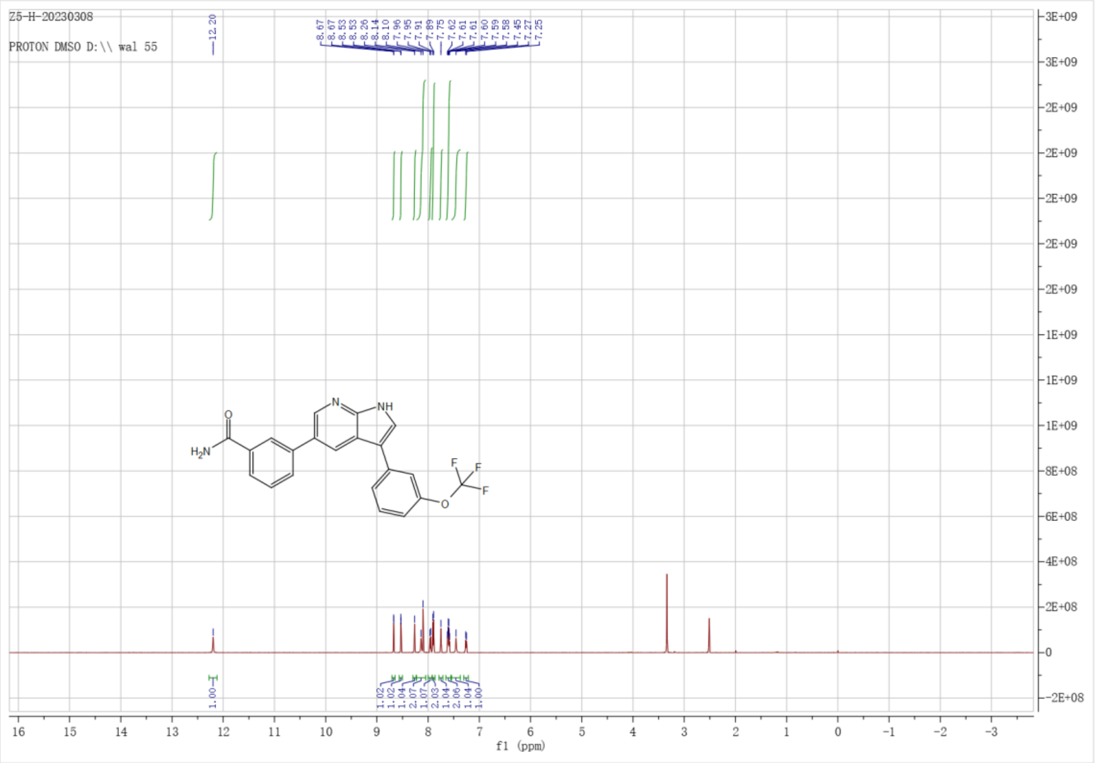

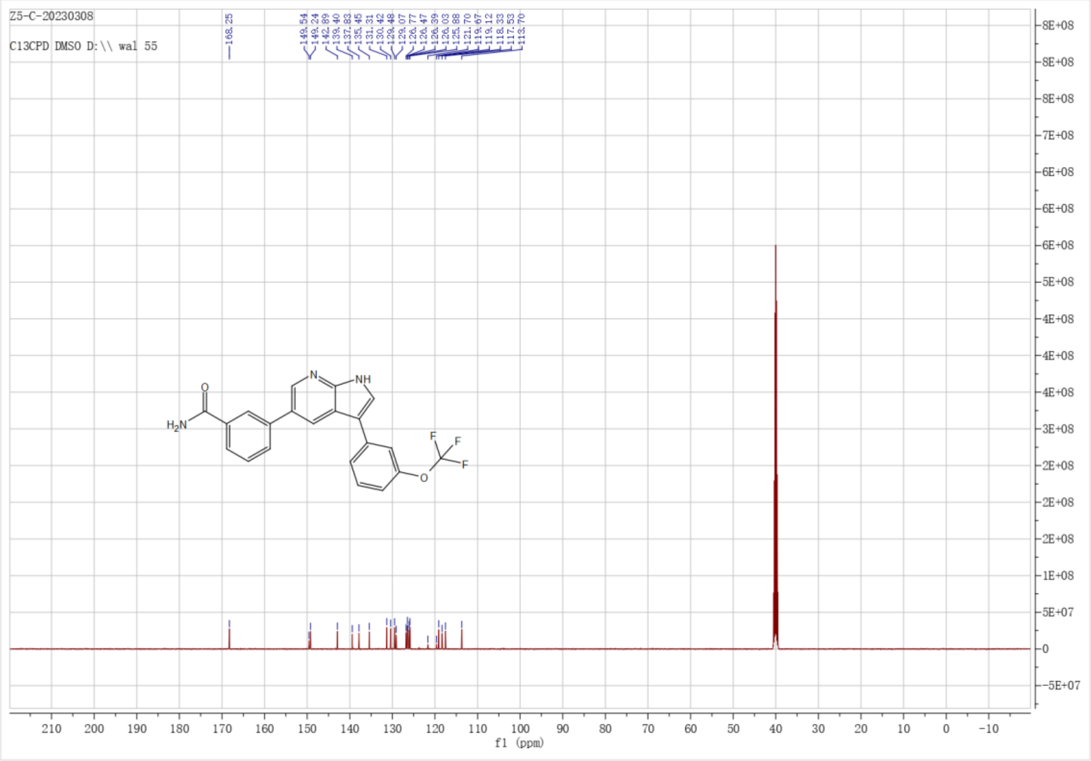

Sample Name		Position		Instrument Name		User Name	
Inj Vol		InjPosition		SampleType		IRM Calibration Status	
Data Filename		ACQ Method		Comment		Acquired Time	

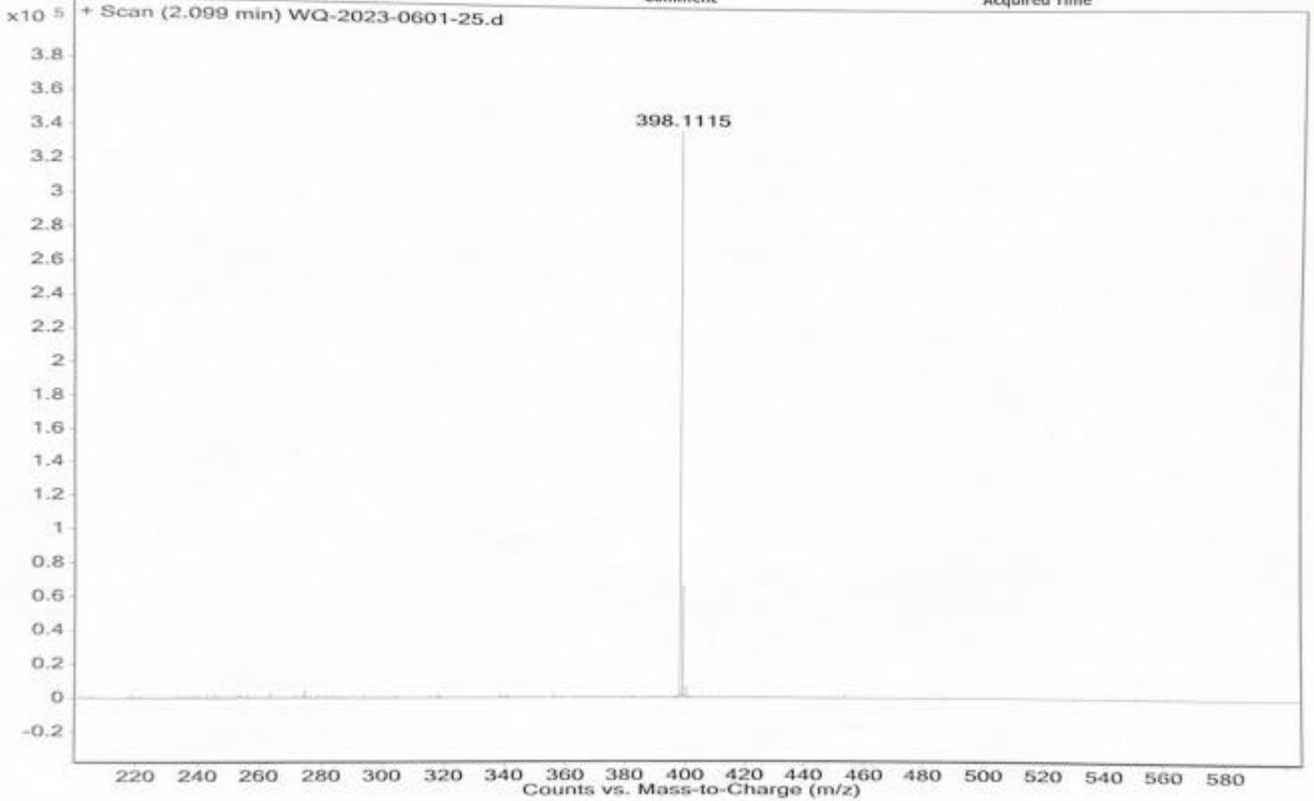

3-(3-(4-fluorophenyl)-1H-pyrrolo[2,3-b]pyridin-5-yl)benzamide (**35**)

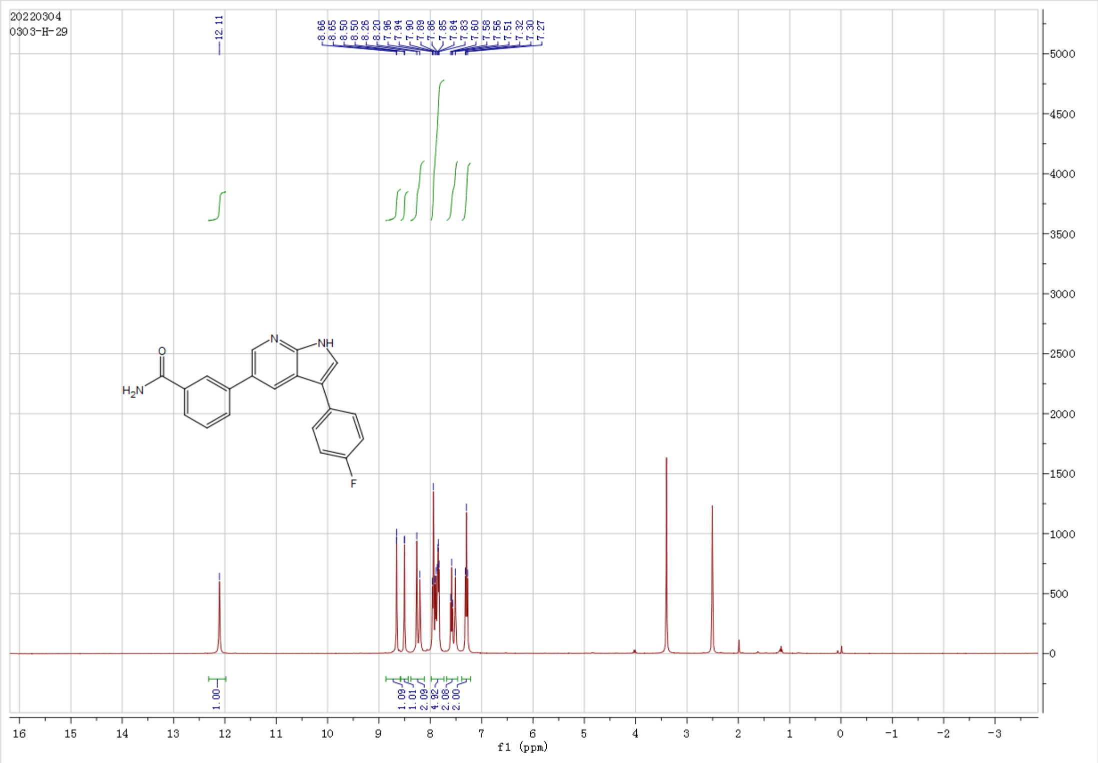

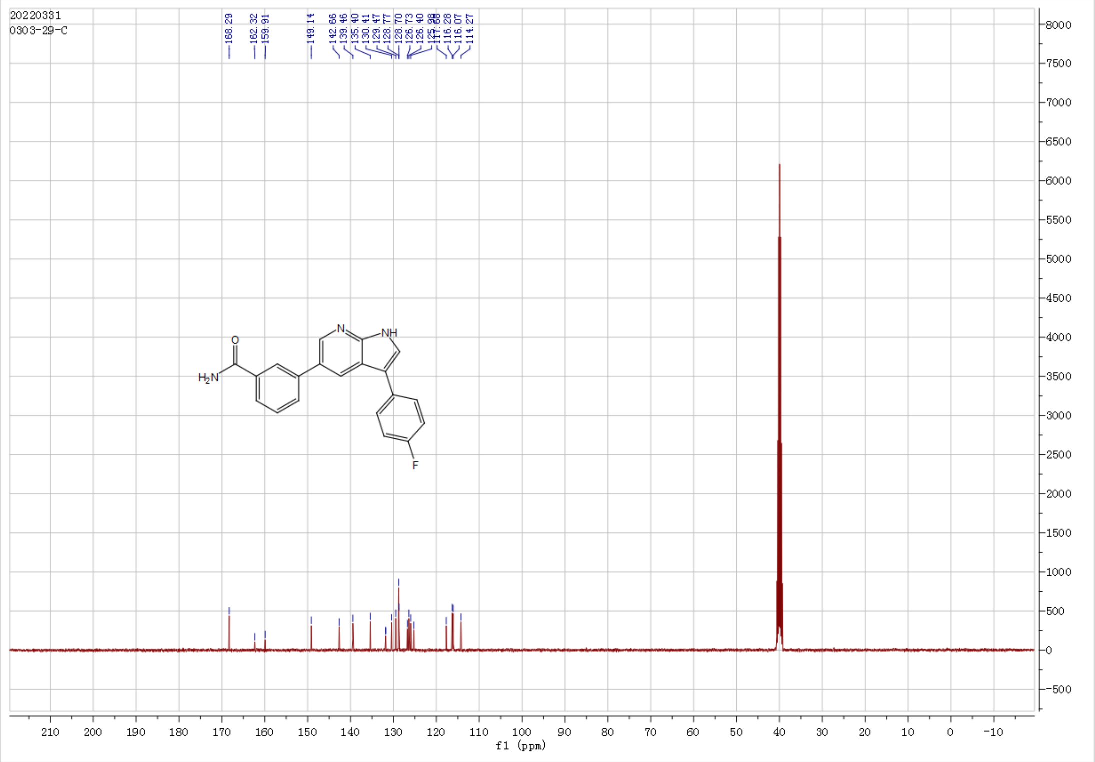

**Sample Name**	ZXX-2022-0301-137	**Position**	Vial 37	**Instrument Name**	Instrument 1	**User Name**	
**Inj Vol**	2	**InjPosition**		**SampleType**	Sample	**IRM Calibration Status**	All Ions Missed
**Data Filename**	ZXX-2022-0301-137.d	**ACQ Method**	2020LXSpo.m	**Comment**		**Acquired Time**	3/1/2022 3:42:52 PM

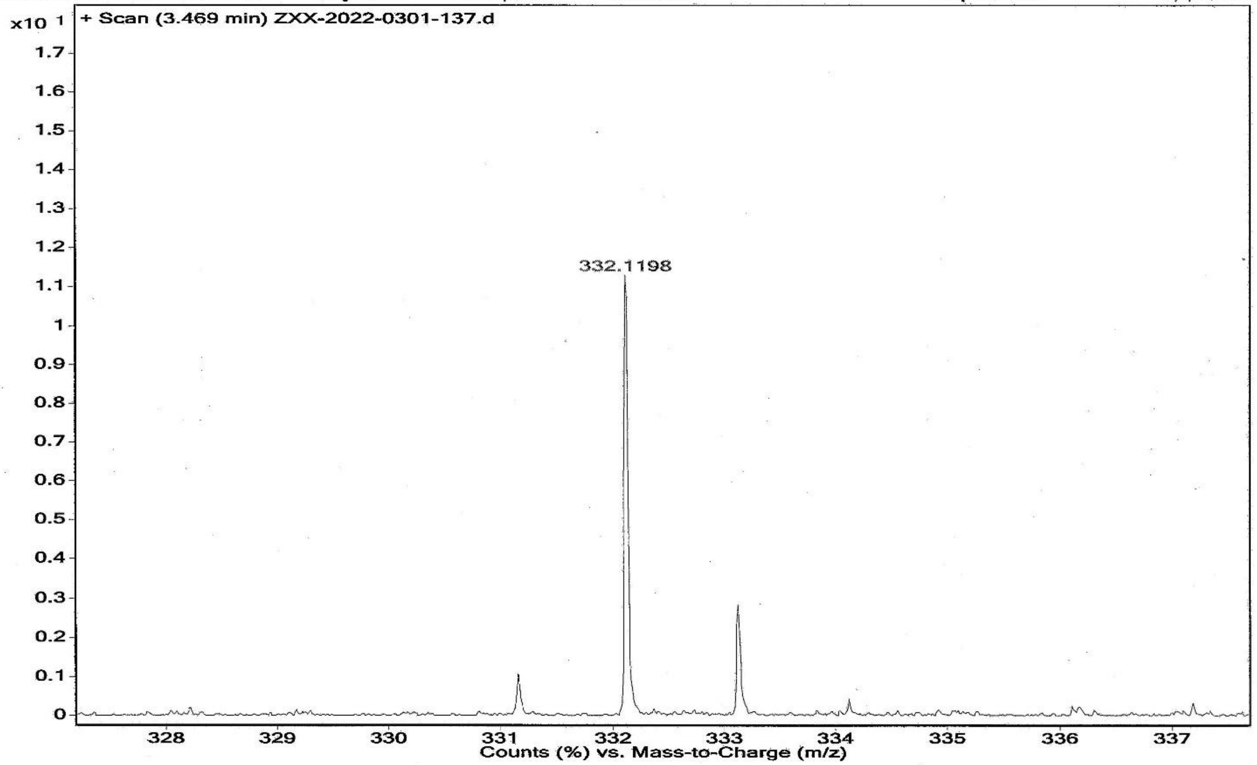

1025-1

3-(3-(4-chlorophenyl)-1H-pyrrolo[2,3-b]pyridin-5-yl)benzamide (**36**)

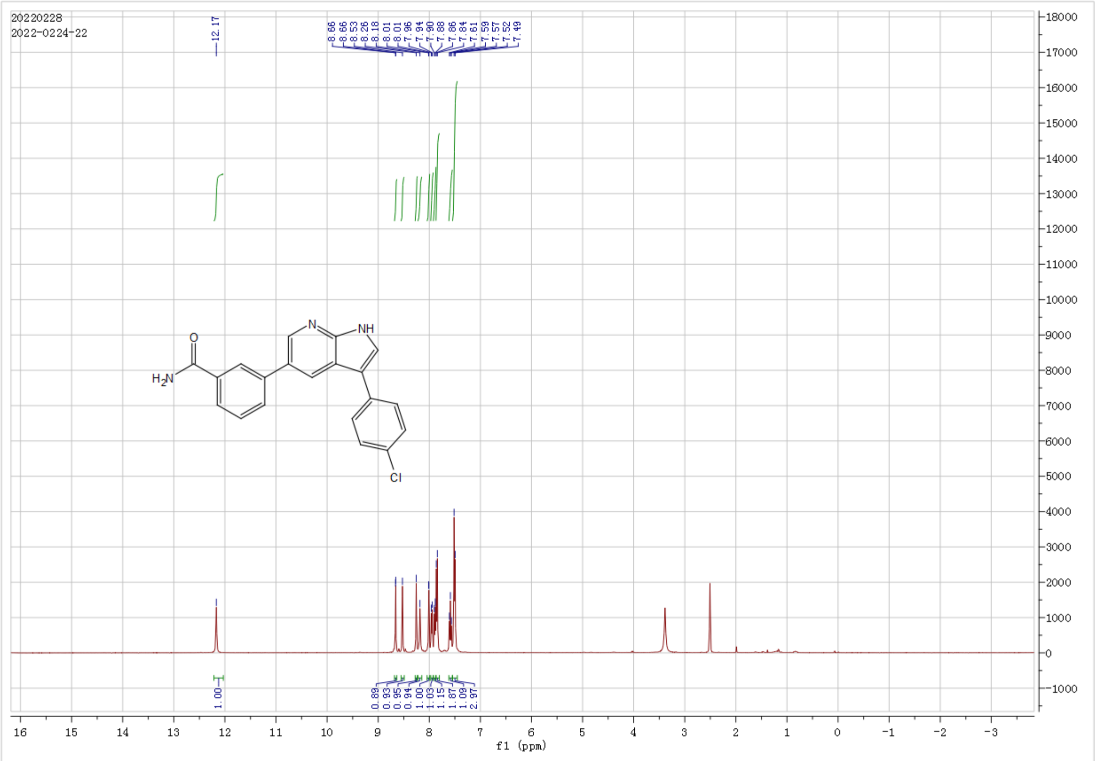

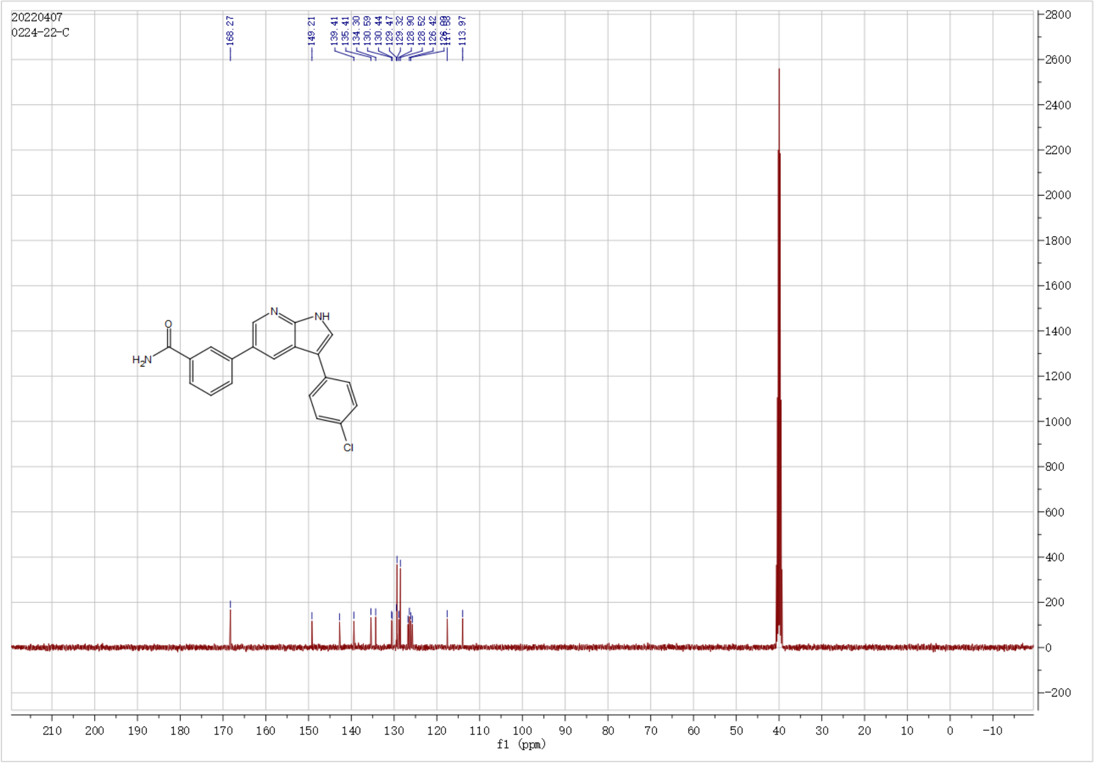

**Sample Name**	ZXX-2022-0224-122	**Position**	Vial 22	**Instrument Name**	Instrument 1	**User Name**	
**Inj Vol**	2	**InjPosition**		**SampleType**	Sample	**IRM Calibration Status**	All Ions Missed
**Data Filename**	ZXX-2022-0224-122.d	**ACQ Method**	2020LXSpo.m	**Comment**		**Acquired Time**	2/25/2022 12:38:31 AM

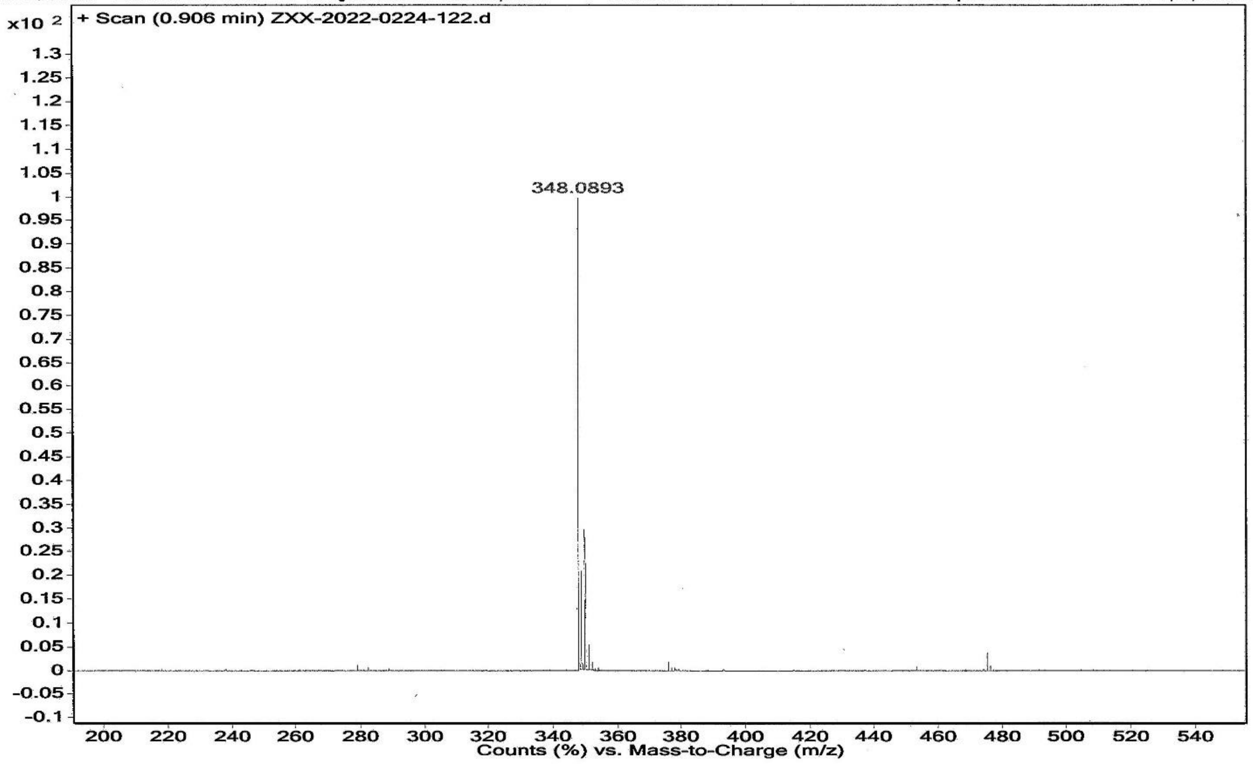

0928-1

3-(3-(4-hydroxyphenyl)-1H-pyrrolo[2,3-b]pyridin-5-yl)benzamide (**37**)

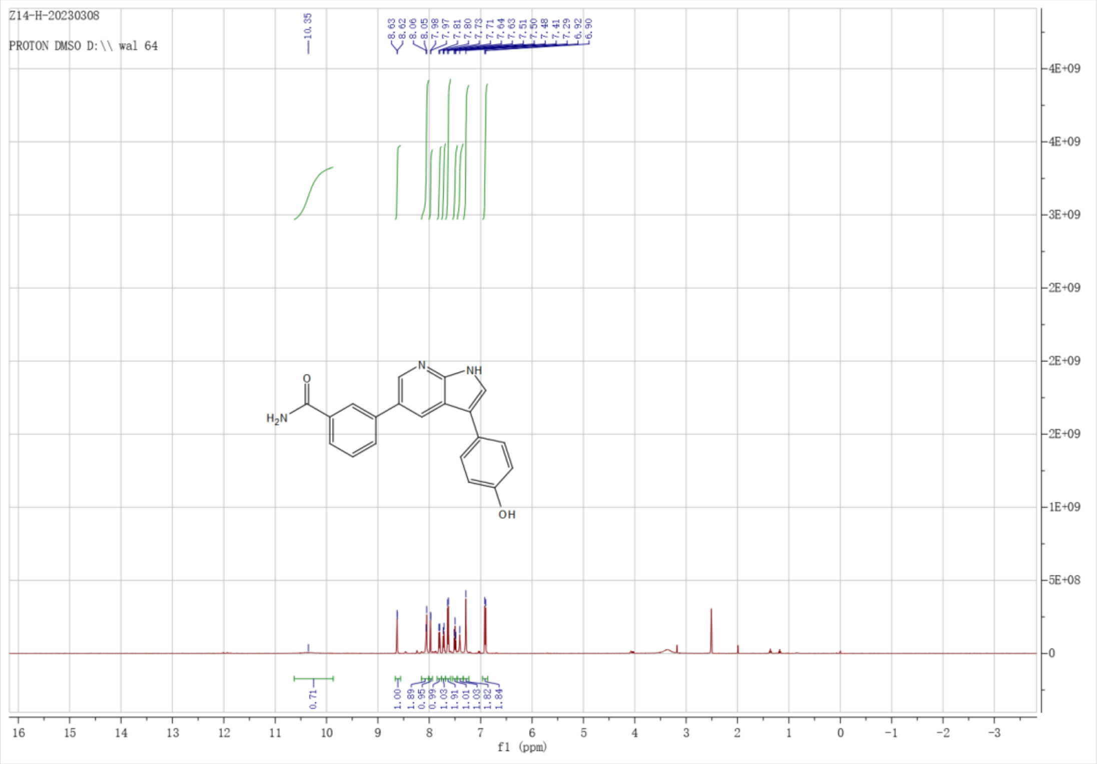

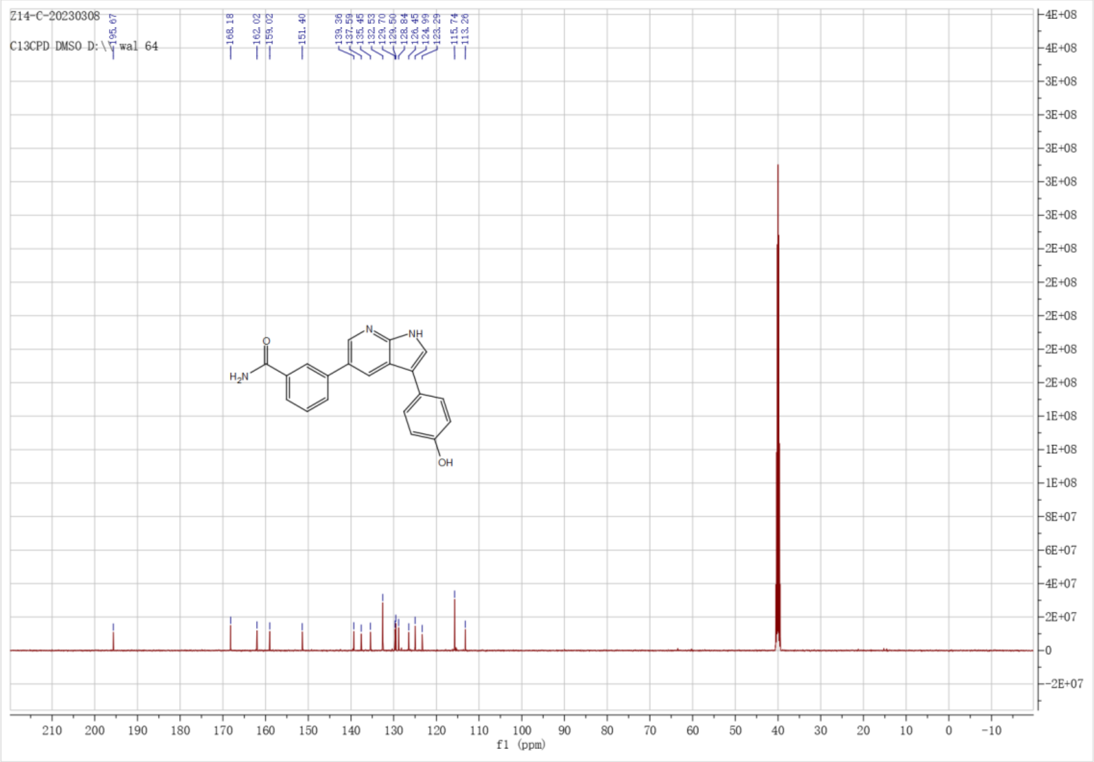

Sample Name	Position	Instrument Name	User Name
Inj Vol	InjPosition	SampleType	IRM Calibration Status
Data Filename	ACQ Method	Comment	Acquired Time

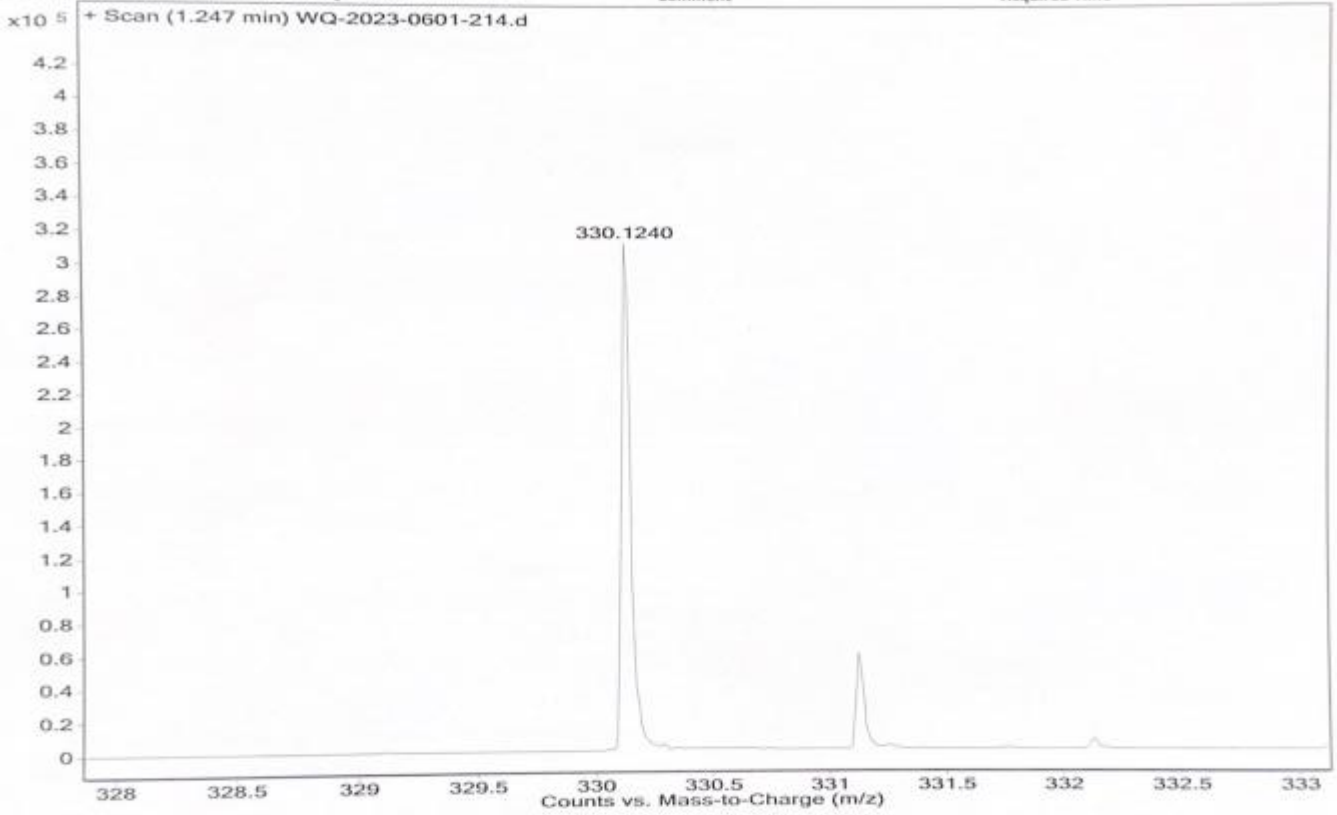

3-(3-(4-methoxyphenyl)-1H-pyrrolo[2,3-b]pyridin-5-yl)benzamide (**38**)

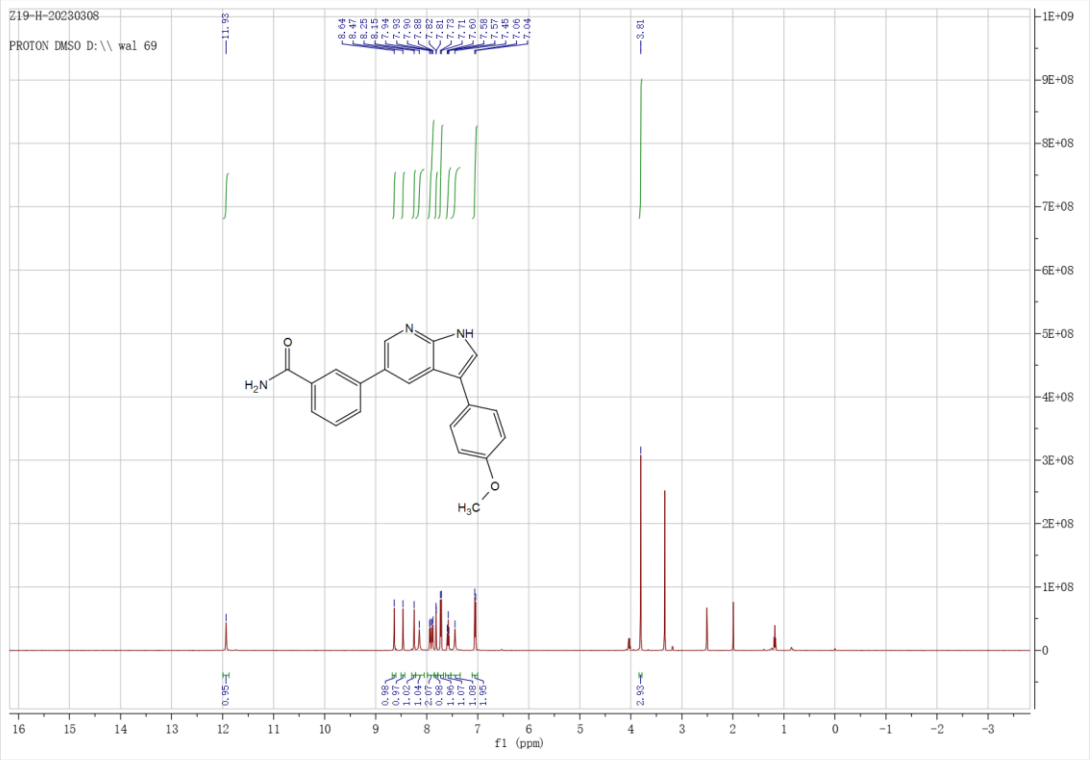

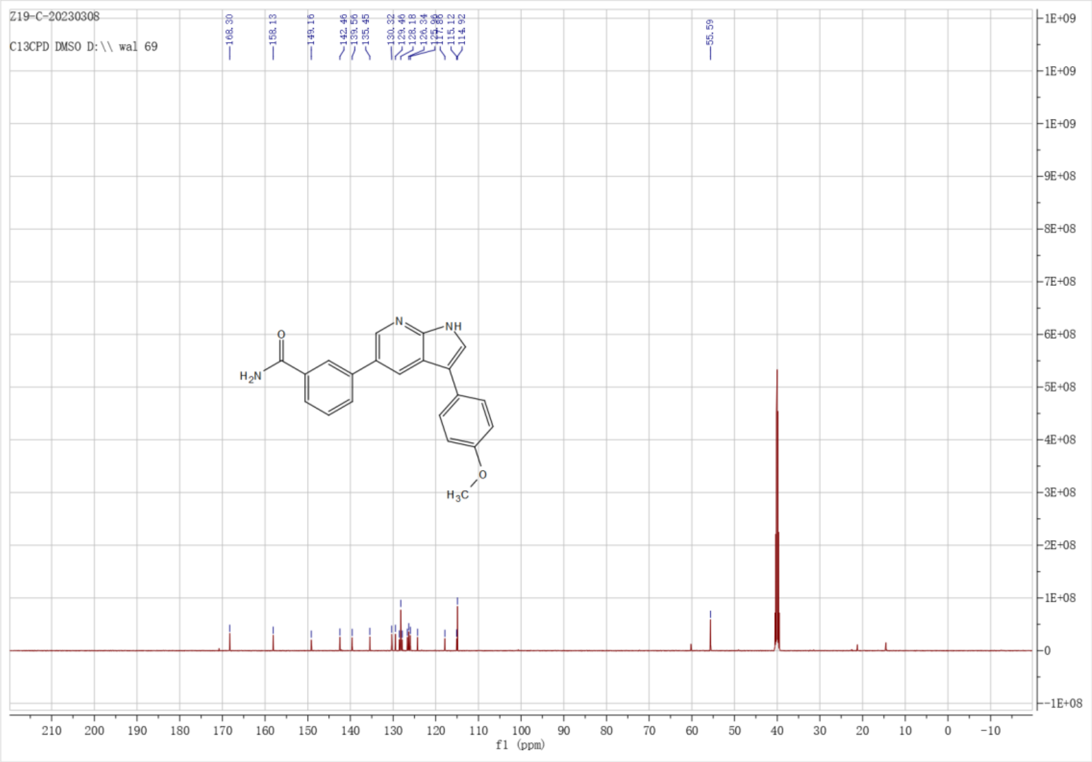

Sample Name	Position	Instrument Name	User Name
Inj Vol	InjPosition	SampleType	IRM Calibration Status
Data Filename	ACQ Method	Comment	Acquired Time

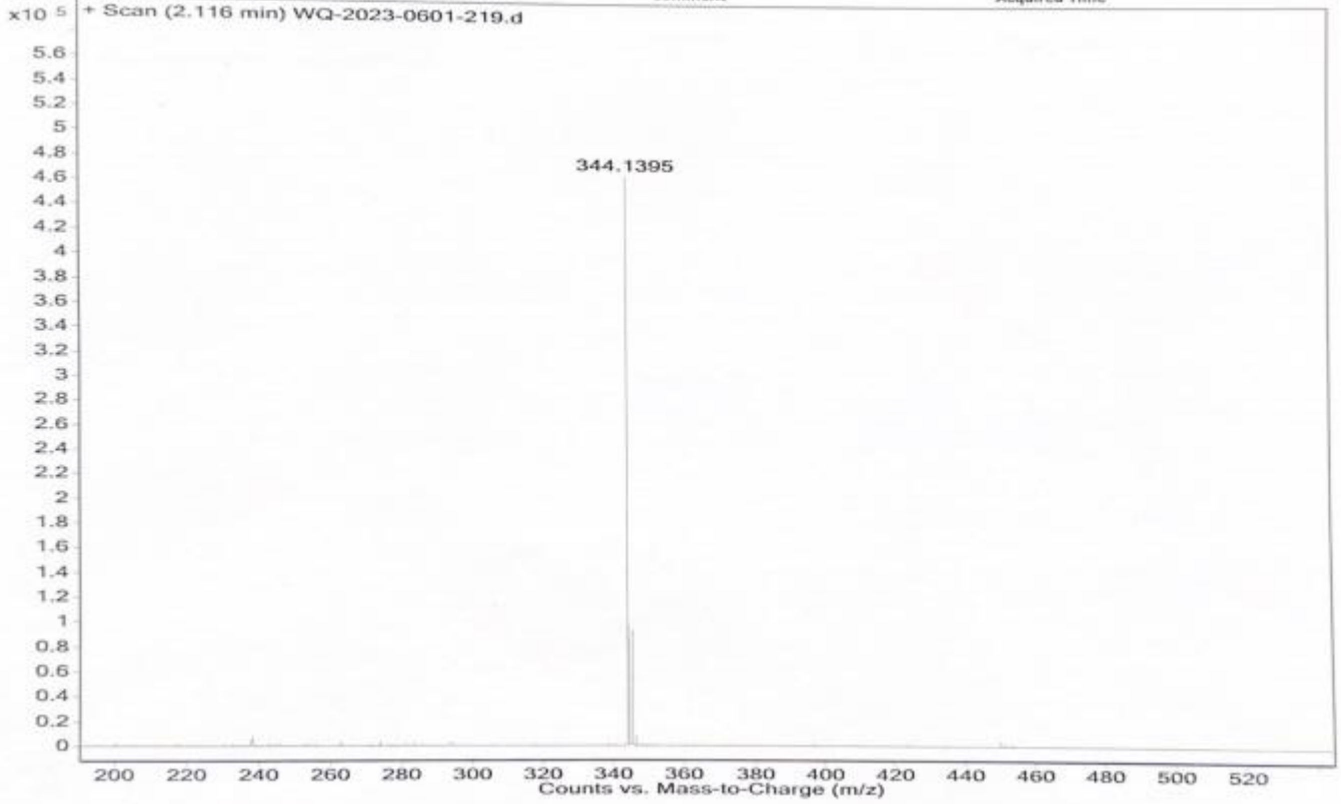

3-(3-(4-(trifluoromethyl)phenyl)-1H-pyrrolo[2,3-b]pyridin-5-yl)benzamide (**39**)

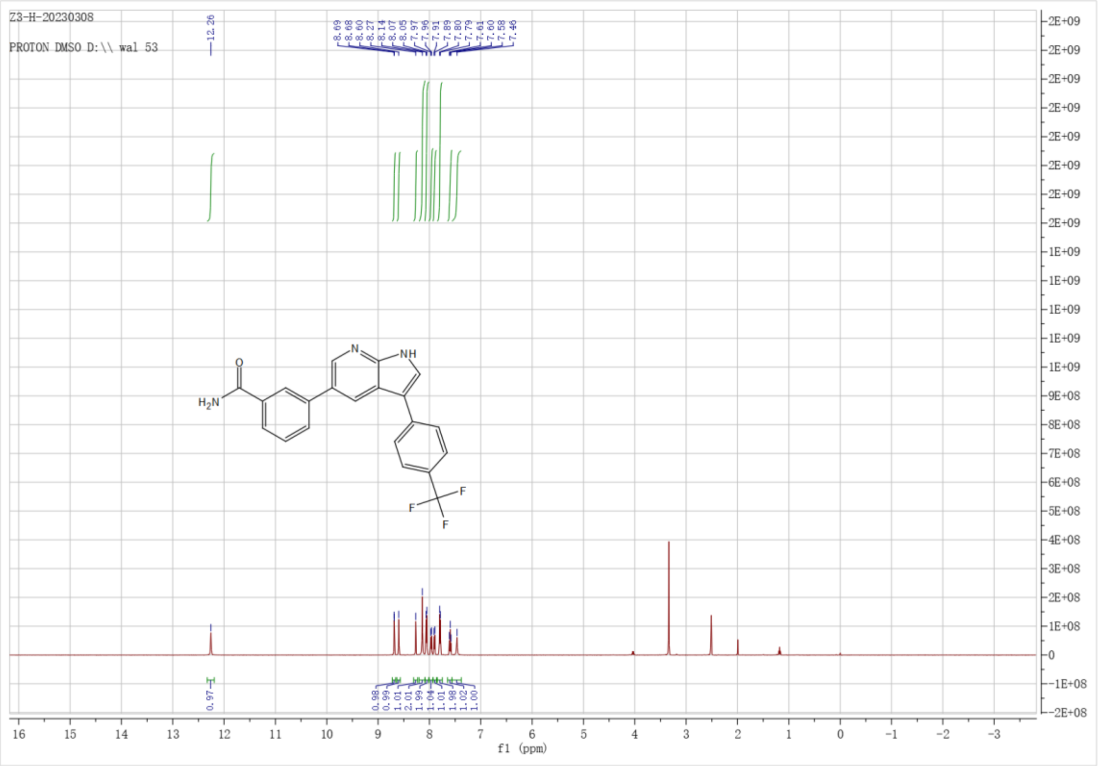

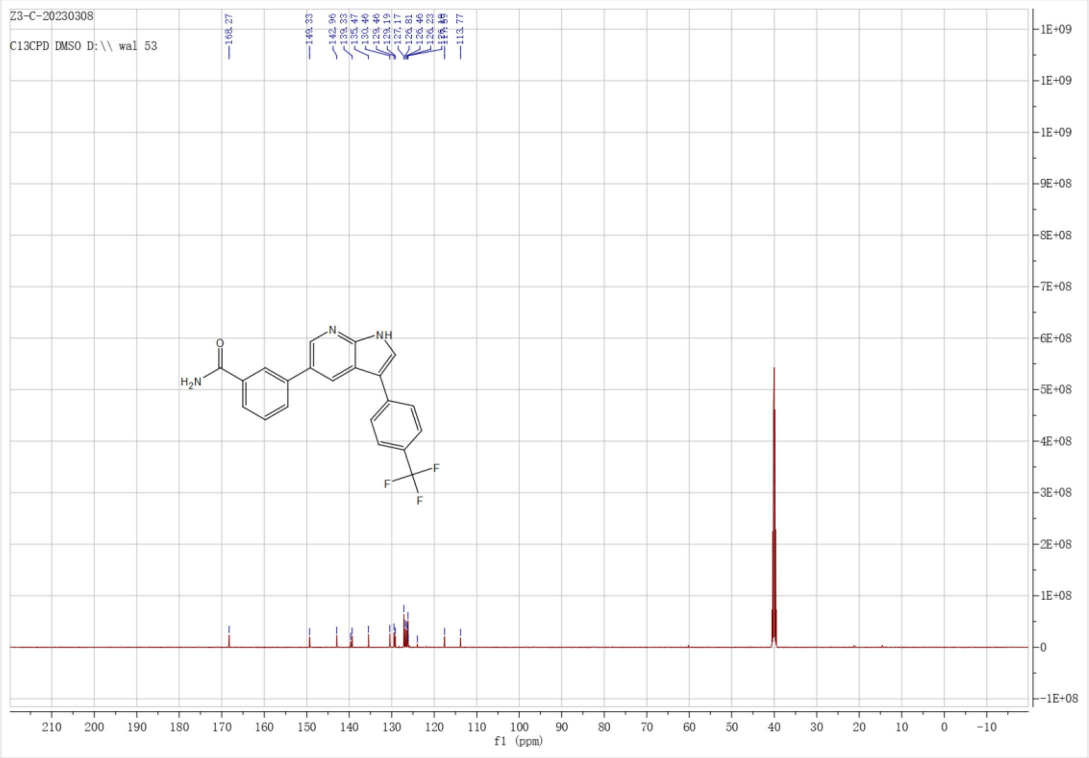

Sample Name	Position	Instrument Name	User Name
Inj Vol	InjPosition	SampleType	IRM Calibration Status
Data Filename	ACQ Method	Comment	Acquired Time

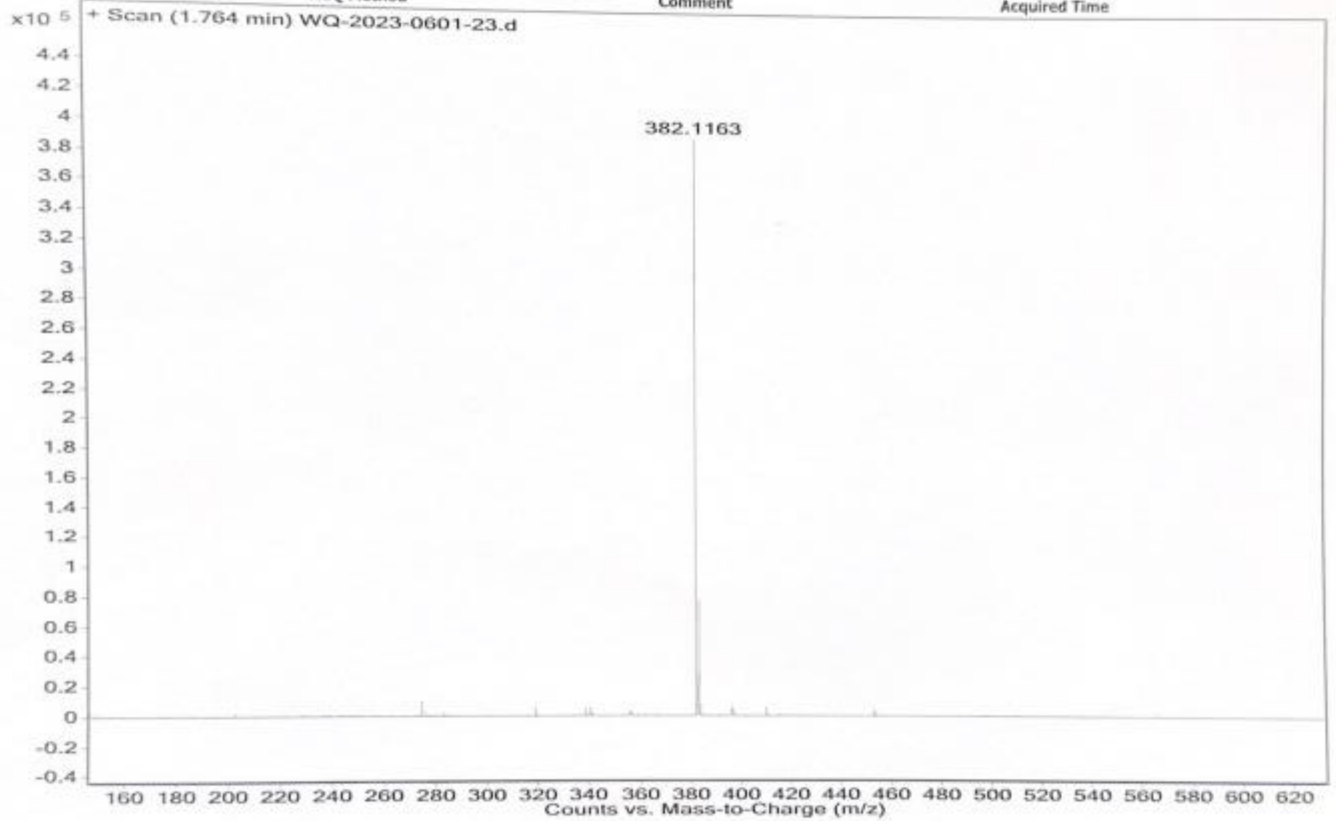

3-(3-(4-(trifluoromethoxy)phenyl)-1H-pyrrolo[2,3-b]pyridin-5-yl)benzamide (**40**)

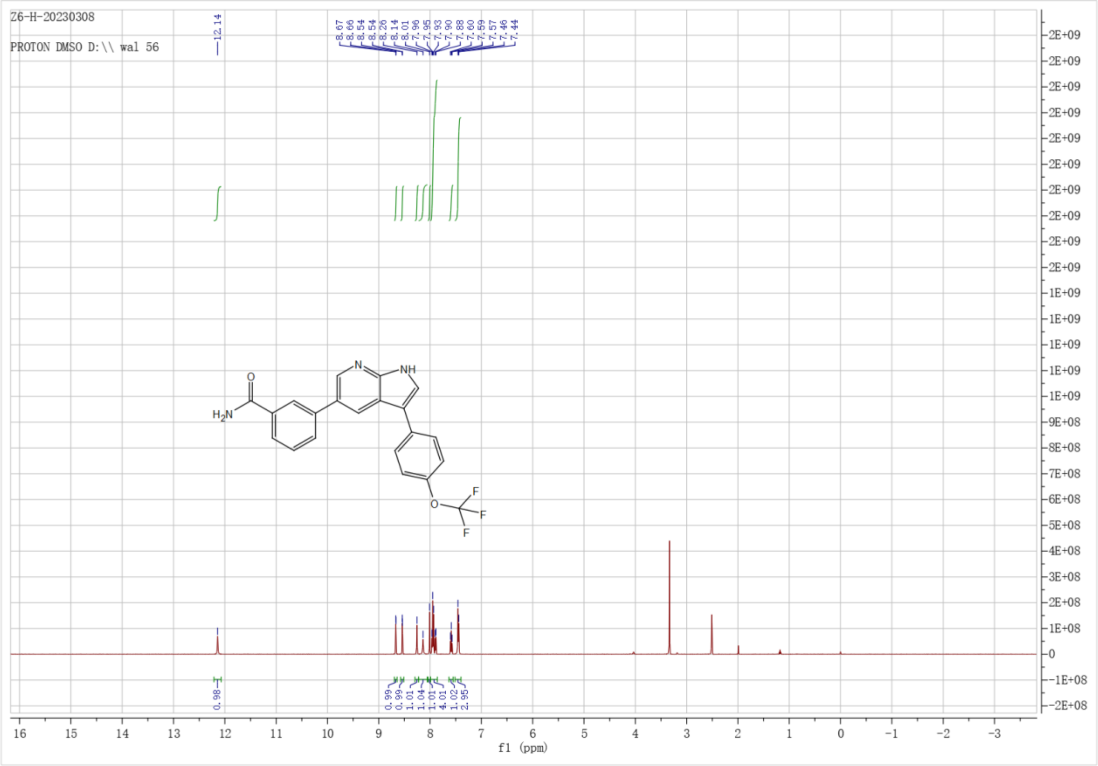

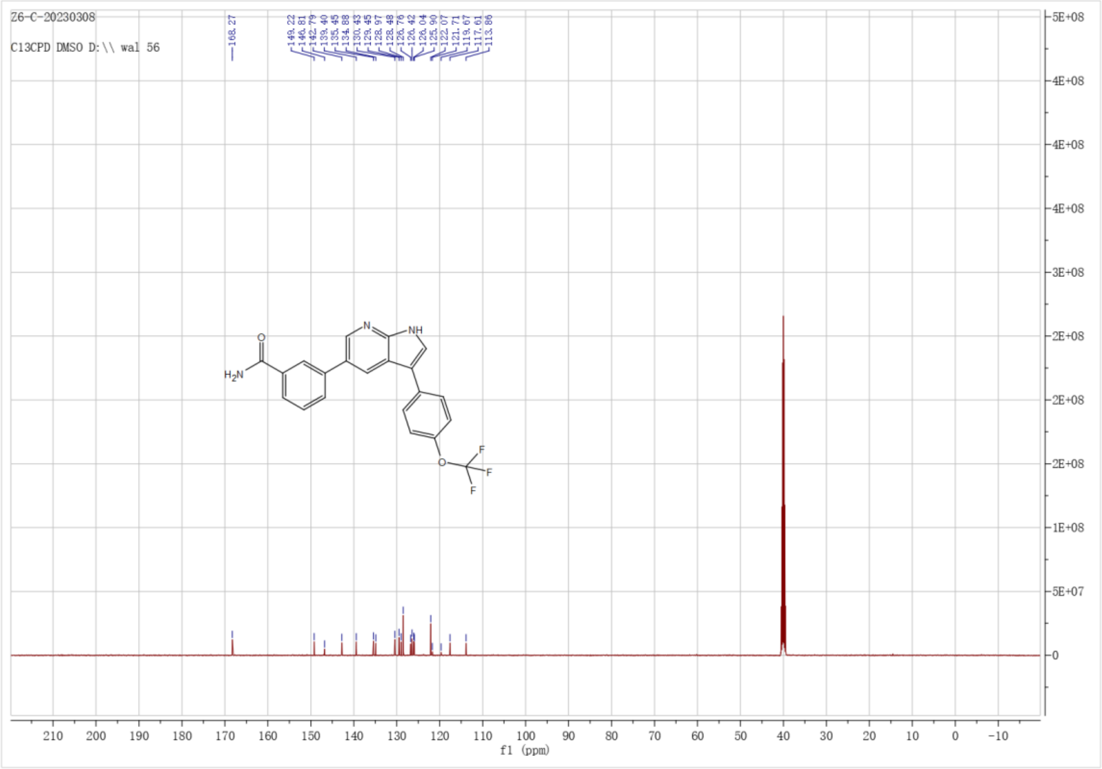

Sample Name	Position	Instrument Name	User Name
Inj Vol	InjPosition	SampleType	IRM Calibration Status
Data Filename	ACQ Method	Comment	Acquired Time

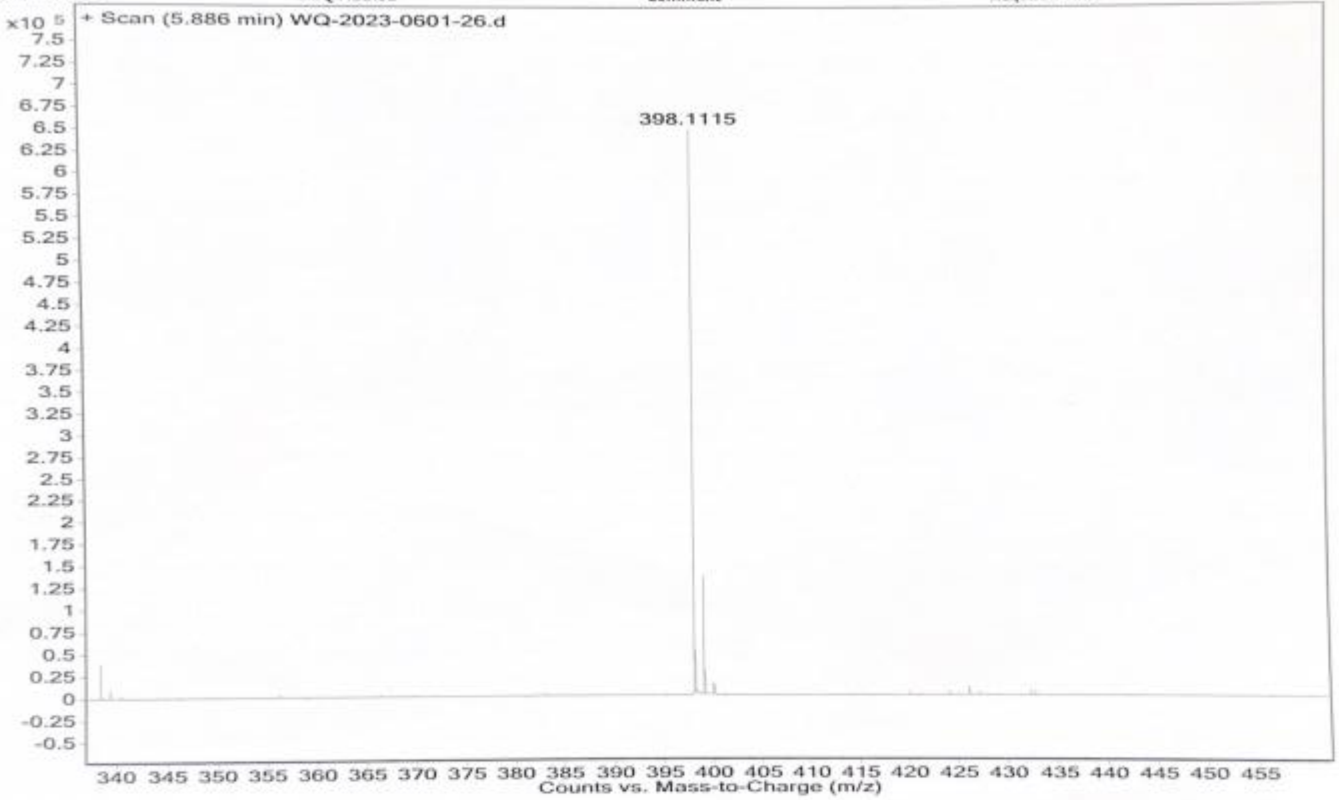

Supplement: Supplemental Material [file IENZ_A_2305852_SM8817.pdf]
